# Temperature-Dependent, Site-Specific Rate Coefficients for the Reaction of OH (OD) with Methyl Formate Isotopologues via Experimental and Theoretical Studies

**DOI:** 10.1021/acs.jpca.4c02524

**Published:** 2024-06-17

**Authors:** Niamh C. K. Robertson, Lavinia Onel, Mark A. Blitz, Robin Shannon, Daniel Stone, Paul W. Seakins, Struan H. Robertson, Christian Kühn, Tobias M. Pazdera, Matthias Olzmann

**Affiliations:** † School of Chemistry, University of Leeds, Leeds LS2 9JT, U.K.; ‡ National Centre for Atmospheric Science, University of Leeds, Leeds LS2 9JT, U.K.; § Dassault Systèmes, 22 Cambridge Science Park, Cambridge CB4 0FJ, U.K.; ∥ Institut für Physikalische Chemie, Karlsruher Institut für Technologie (KIT), 76131 Karlsruhe, Germany

## Abstract

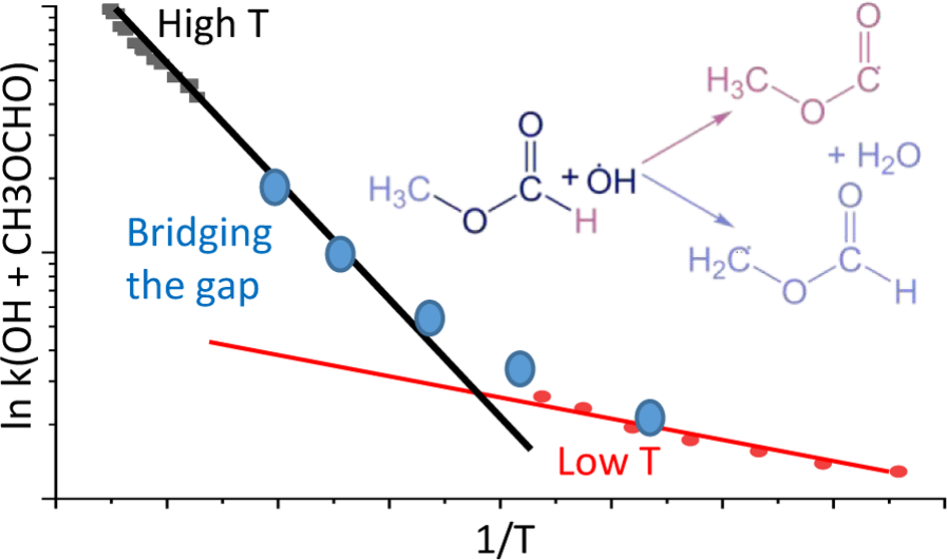

Methyl esters are an important component of combustion and atmospheric systems. Reaction with the OH radical plays an important role in the removal of the simplest methyl ester, methyl formate (MF, CH_3_OCHO). In this paper, the overall rate coefficients for the reactions of OH and OD with MF isotopologues, studied under pseudo-first-order conditions, are reported using two different laser flash photolysis systems with the decay of OH monitored by laser-induced fluorescence. The room-temperature rate coefficient for OH + MF, (1.95 ± 0.34) × 10^–13^ cm^3^ molecule^–1^ s^–1^, is in good agreement with the literature. The rate coefficient exhibits curved Arrhenius behavior, and our results bridge the gap between previous low-temperature and shock tube studies. In combination with the literature, the rate coefficient for the reaction of OH with MF between 230 and 1400 K can be parametrized as *k*_OH+MF_ = (3.2 × 10^–13^) × (T/300 K)^2.3^ × exp(−141.4 K/*T*) cm^3^ molecule^–1^ s^–1^ with an overall estimated uncertainty of ∼30%. The reactions of OD with MF isotopologues show a small enhancement (inverse secondary isotope effect) compared to the respective OH reactions. The reaction of OH/OD with MF shows a normal primary isotope effect, a decrease in the rate coefficient when MF is partially or fully deuterated. Experimental studies have been supported by *ab initio* calculations at the CCSD(T)-F12/aug-cc-pVTZ//M06-2*X*/6-31+G** level of theory. The calculated, zero-point-corrected, barrier heights for abstraction at the methyl and formate sites are 1.3 and 6.0 kJ mol^–1^, respectively, and the *ab initio* predictions of kinetic isotope effects are in agreement with experiment. Fitting the experimental isotopologue data refines these barriers to 0.9 ± 0.6 and 4.1 ± 0.9 kJ mol^–1^. The branching ratio is approximately 50:50 at 300 K. Between 300 and 500 K, abstraction via the higher-energy, higher-entropy formate transition state becomes more favored (60:40). However, experiment and calculations suggest that as the temperature increases further, with higher energy, less constrained conformers of the methyl transition state become more significant. The implications of the experimental and theoretical results for the mechanisms of MF atmospheric oxidation and low-temperature combustion are discussed.

## 1 Introduction

Esters can be released via industrial processes, for example, as solvents or in plasticizers, and as components of biofuels, particularly biodiesels composed of fatty acid methyl esters.^1,2^ As biofuel consumption is expected to increase, so is the release of esters via incomplete combustion and fugitive emissions. Recently, methyl formate has been proposed as an efficient hydrogen carrier.^3^ Esters are also formed *in situ* following the atmospheric oxidation of ethers, for example, methyl formate (MF), CH_3_OCHO, is formed with a 90 ± 8% yield from dimethyl ether,^4^ another proposed biofuel. As the simplest ester, MF can be used as a proxy to understand ester functionality.^5^ This work considers conditions relevant to low-temperature combustion chemistry and the atmospheric oxidation of esters.

The atmospheric removal of MF is primarily driven by its reaction with the OH radical. There have been several studies of the overall kinetics of reaction R1 in the literature^6−9^ with room-temperature rate coefficients being in good agreement, varying from 1.73 to 2.27 × 10^–13^ cm^3^ molecule^–1^ s^–1^.
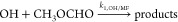
R1

There are fewer temperature-dependent studies. Le Calvé et al.^6^ suggest a relatively flat temperature dependence over the range of their measurements (230–372 K) and a simple extrapolation of the Le Calvé et al. data is in poor agreement with the high-temperature (876–1371 K) shock tube data of Lam et al.^10^ At the other extreme of temperature, Jimenez et al.^11^ report a marked acceleration of the overall rate coefficient at low temperatures, consistent with the reaction of OH with other oxygenated species.^12^ Studies above 372 K are limited to the shock tube data of Lam et al.

Site-specific rate coefficients are the rate coefficient for a reaction site within a molecule, with the sum of all site-specific rate coefficients giving the total rate coefficient.^13^ Site-specific rate coefficients give the ratio of reaction products and therefore determine a molecule’s oxidation pathway.^14^ The oxidation of MF has two reaction sites: hydrogen abstraction from the methyl (R1_CH_3__) or the formate group (R1_CHO_).
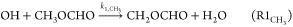

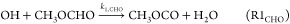


Abstraction at the methyl site leads, under atmospheric conditions, to the major products formic acid (HC(O)OH) and formic acid anhydride,^15^ molecules which can both contribute to aerosol growth/formation, while abstraction from the formate group leads to carbon dioxide and formaldehyde. Currently, there are significant uncertainties in the atmospheric budgets of organic acids.^16^ Accurate determination of branching ratios for the reaction of OH with MF helps to constrain the rates of formic acid formation in atmospheric models.

As mentioned above, MF is a model fuel for understanding biodiesel combustion. Under low-temperature combustion conditions, CH_3_OCO, formed in reaction R1_CHO_, is expected to decompose rapidly, removing any potential for RO_2_/QOOH chemistry (see, e.g., ref (2)) from this pathway and providing a source of methyl radicals
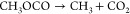
R2CH_2_OCHO, formed in reaction R1_CH_3__, is significantly more stable than CH_3_OCO and can undergo RO_2_/QOOH chemistry
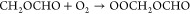
R3a
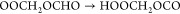
R4However, the resulting QOOH radical, HOOCH_2_OCO, is very unstable, dissociating to HO + HCHO + CO_2_ before further O_2_ addition. Thus, the above discussions show that first-generation products formed following OH abstraction under both atmospheric and low-temperature combustion conditions will differ depending on the OH abstraction site.

There are limited data on the branching ratio for OH attack on MF; an end-product study by Wallington et al.^17^ measured an approximate 50:50 branching ratio at room temperature. In contrast, a modeling study by Dooley et al.^5^ suggests that abstraction at the methyl site dominates ∼6:1 at room temperature, falling to ∼3:1 at 1000 K.

Additionally, there have been a number of theoretical studies on Reaction 1.^9,18−20^ With the exception of the early study by Good et al.,^9^ calculation predicts a lower barrier for abstraction at the methyl site, although there are wide variations in the absolute barrier height. Another aspect of theoretical study has been the potential of the CH_3_OCO radical, formed following R1_CHO_, to undergo chemically activated decomposition,^21^ a process that may have an impact on combustion.^22^

In this work, site-specific data on the reaction of OH with MF were obtained through the temperature-dependent kinetics of OH and OD with the deuterated isotopologues CH_3_OCHO (MF), CH_3_OCDO (MF-d1), CD_3_OCHO (MF-d3), and CD_3_OCDO (MF-d4). Measurements were taken using pulsed laser photolysis (PLP) and probing OH/OD loss by pulsed laser-induced fluorescence (PLIF) between 294 and ∼600 K using two experimental apparatus in Leeds (pressures between 6 and 130 mbar), and Karlsruhe (pressures of 2, 5, and 10 bar), bridging the gap between low- and high-temperature measurements by Le Calvé et al.^6^ (233–372 K) and Lam et al.^10^ (880–1344 K). Subsequent addition of oxygen can lead to OH production either via a chemically activated process at low pressures or, at higher temperatures, via a thermal reaction. Addition of oxygen to form peroxy radicals is in competition with radical dissociation. Studies on the regeneration of OH/OD in the reaction of OH/OD with CH_3_OCDO, in the presence of oxygen, provide complementary data on the branching ratios for R1.

The experiments were supported by theoretical calculations. An *ab initio* surface for reaction 1 was computed at the CCSD(T)-F12/aug-cc-pVTZ//M06-2*X*/6-31+G** level and associated frequencies used to fit the kinetic data to extract branching ratios using the master equation program MESMER.^23^ Companion papers to this work will explore the fates of the CH_2_OCHO and CH_3_OCO radicals, looking at the R + O_2_ reaction and OH recycling from RO_2_ production.

## 2 Methodology

### 2.1 Experimental, University of Leeds

Laser flash photolysis has been used, with laser-induced fluorescence (LIF) to monitor the decay of OH and OD. The apparatus has been described in detail in some previous publications.^24−26^ Methyl formate and a bath gas, typically Ar, were flowed through calibrated mass-flow controllers into a mixing manifold before being flowed into the stainless steel reaction cell. The reaction cell was heated with a ceramic oven, and temperatures were monitored via calibrated thermocouples located close to the reaction zone. Pressures in the cell were recorded with a capacitance manometer.

Hydrogen peroxide (H_2_O_2_) and *tert*-butyl peroxide (tBuOOH/(CH_3_)_3_COOH) were used as OH precursors. Deuterated equivalents were made by adding D_2_O to form D_2_O_2_ and (CH_3_)_3_COOD. H_2_O_2_ was introduced via a pressurized bubbler, whereas tBuOOH was flowed through a calibrated mass-flow controller. CH_3_OCHO (Sigma-Aldrich, 99%), CH_3_OCDO (Sigma-Aldrich, 99%), CD_3_OCHO (Sigma-Aldrich, 99%), and CD_3_OCDO (QMX Laboratories, 99%) were purified via several freeze–thaw pump cycles and stored as diluted mixtures in argon in glass bulbs.

**Figure 1:**
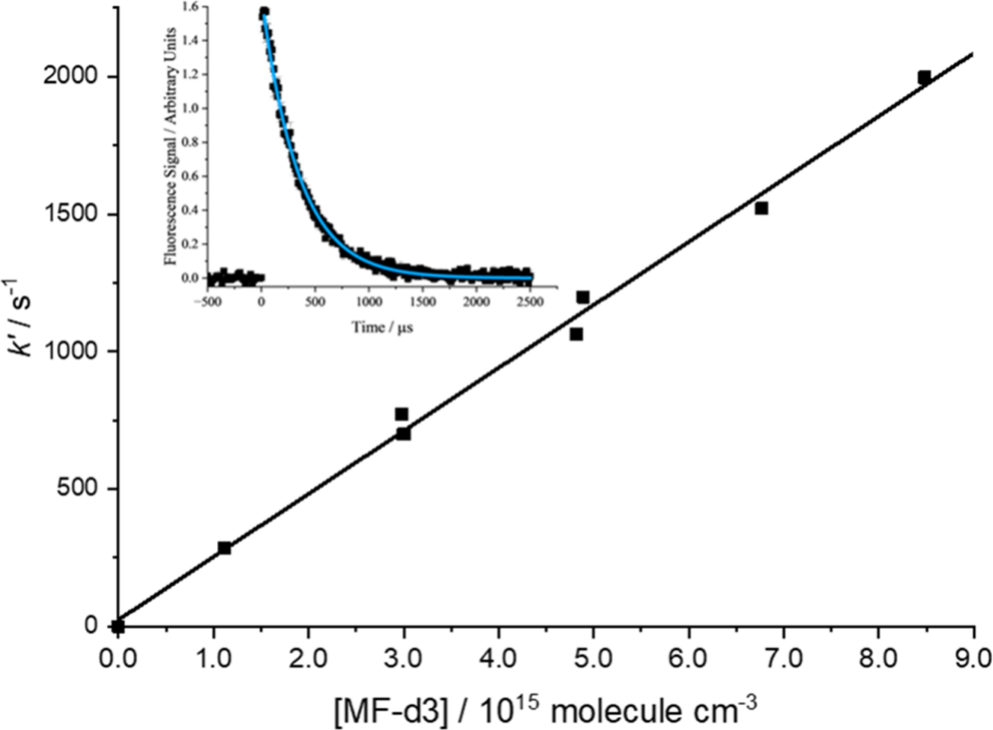
Bimolecular plot for the overall reaction of OH with MF-d3 at 393 K and 77 mbar of argon. The weighted linear fit to the data gives *k*_1_ = (2.34 ± 0.08) × 10^–13^ cm^3^ molecule^–1^ s^–1^ where the error is statistical at the 1σ level. The inset shows a typical exponential OH decay and fit (eq E1) with [CD_3_OCHO] = 6.77 × 10^15^ molecules cm^–3^.

OH and OD radicals were generated via the photolysis of the respective precursor by the fourth harmonic of a pulsed Nd:YAG laser (Quantel Q-smart 850 at 10 Hz) at 266 nm. Radicals were subsequently excited by a Nd:YAG pumped dye laser at 282 (Spectra-Physics, Quanta-Ray, PDL-3, Rhodamine 6G blended with Pyromethane 580 dye) (*A*^2^∑^+^(*v* = 1) ← *X*^2^Π(*v* = 0)) or 308 nm (*A*^2^∑^+^(*v* = 0) ← *X*^2^Π(*v* = 0)) (Sirah, Cobra Stretch, DCM special dye). Fluorescence was detected at ∼308 nm (OH detection at 307.3 nm and OD detection at 307.25 nm) through an interference filter (Barr Associates, Inc., 308.5 ± 5 nm). Off-resonance detection used a photomultiplier tube (PMT) (EMI 9813), whereas on-resonance fluorescence used a channel photomultiplier (CPM, PerkinElmer C1943P). Temporal profiles of decay were monitored by altering the delay, using a digital delay generator (BNC DG535), between the photolysis and probe laser from 0 to 10 μs, scans were typically taken five times. A typical decay profile is shown in the inset to Figure 1.

Reactions were performed at pressures greater than 66 mbar such that chemically activated OH recycling from any trace oxygen was eliminated, and single exponential decays were observed up to ∼490 K. While no oxygen was added, there was a background oxygen concentration of approximately 1 × 10^14^ molecules cm^–3^. This led to biexponential decays above 490 K due to thermal decomposition of the OOCH_2_OCHO peroxy radical.

Pseudo-first-order conditions, with [MF] typically 1–12 × 10^15^ ≫ [OH] < 10^12^, were used such that the decay of the OH LIF signal, *S*_OH_, below 490 K was given by
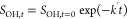
E1where *S*_OH,t_ is the time-dependent LIF signal, *S*_OH,t=0_ is the initial signal after the photolysis laser pulse, *t* is the time between the photolysis and probe pulses, and *k*′ is given by
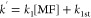
E2where *k*_1st_ represents the sum of the first-order loss processes such as diffusion (approximated to first order) and reaction with the constant concentration of OH precursor. A bimolecular plot, as shown in Figure 1 for *k′ vs* [MF-d3], gives the overall rate coefficient, *k*_1_, as the gradient and *k*_first_ as the intercept.

Biexponential decays above ∼490 K were analyzed using E3 (27)
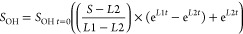
E3with
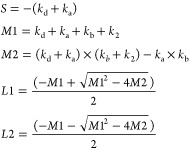
where *k*_d_ represents the loss of OH via diffusion and its reaction with the precursor, *k*_a_ describes the pseudo-first-order rate coefficient for the reaction of OH with methyl formate, *k*_b_ is the rate coefficient for the thermal decomposition of RO_2_, and *k*_2_ is the rate coefficient for the total loss of RO_2_. An example biexponential decay and biexponential plot using *k*_a_ are shown in the SI (Figure S1).

### 2.2 Experimental, KIT

The experimental setup at KIT was similar to that used at the University of Leeds. Its general features have been described earlier,^28−30^ and the details of the specific configuration used in the present work were identical to those in recent publications.^31,32^ In brief, the apparatus consisted of a heatable slow-flow reactor made of stainless steel with a T-shaped arrangement of three quartz windows. The photolysis laser and the fluorescence excitation laser were propagated antiparallel through the cell via two opposite parallel windows, and the fluorescence was monitored perpendicular to the laser axes through the third window.

In contrast to the Leeds experiments, HNO_3_ (synthesized from H_2_SO_4_ and KNO_3_)^31,32^ was used as the OH precursor, and He served as the bath gas. The OH radicals were produced by pulsed photolysis of the precursor with a KrF-excimer laser (Lambda Physik, Compex102) at 248 nm and detected by laser-induced fluorescence excited at 282 nm with a frequency-doubled (BBO crystal) dye laser (Lambda Physik ScanMate2E, Coumarin 153 in ethanol). The dye laser was pumped by an XeCl excimer laser (Lambda Physik, Compex102) at 308 nm. The fluorescence after passing through a monochromator (Carl Zeiss, MQ4III, 308 ± 4 nm) was monitored with a photomultiplier tube (Hamamatsu, R22A). The time delay between the pulses of the photolysis and fluorescence excitation laser was set with a delay generator (Stanford, DG535); the repetition rate was 10 Hz.

**Figure 2:**
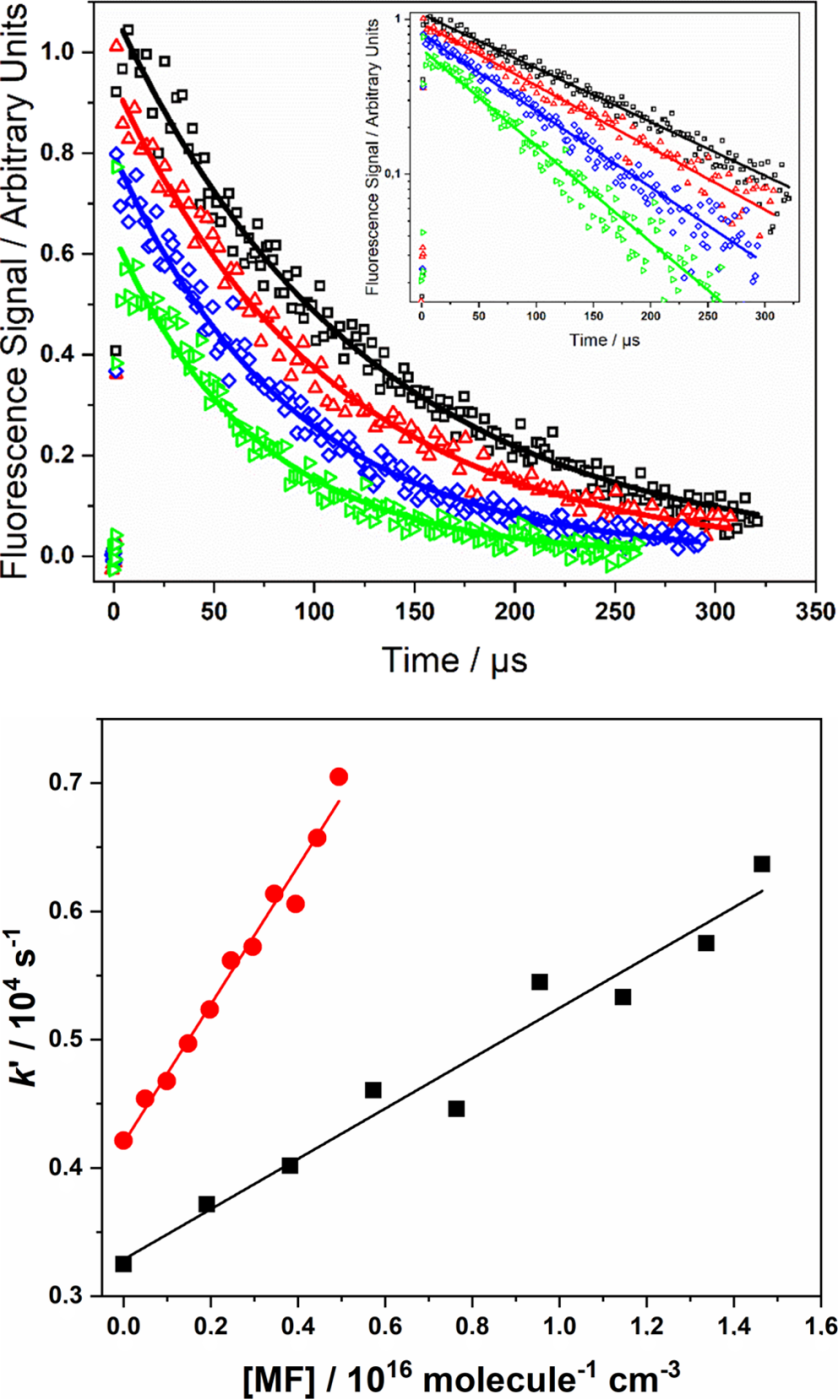
Top: Exponential OH decay profiles at *T* = 455 K and *p* = 5 bar for different initial MF concentrations of (top-down) 1.41 × 10^15^ cm^–3^, 4.23 × 10^15^ cm^–3^, 8.46 × 10^15^ cm^–3^, and 1.41 × 10^16^ cm^–3^. Bottom: Bimolecular plots at *p* = 2 bar and (black) *T* = 295 K and (red) *T* = 520 K giving *k*_1_ = (1.96 ± 0.31) × 10^–13^ cm^3^ s^–1^ and *k*_1_ = (5.39 ± 0.48) × 10^–13^ cm^3^ s^–1^, respectively.

The gases were stored in stainless steel cylinders, and flows were regulated with mass-flow controllers to avoid accumulation of reaction products in the cell. Pseudo-first-order conditions, with [MF] typically in the range 5 × 10^14^ to 5 × 10^16^ cm^–3^ ≫ [OH] < 3 × 10^13^ cm^–3^, were used. The estimated ratios of the initial concentrations of [MF]_0_/[OH]_0_ were always between 150 and 2000, and only monoexponential decays of the OH LIF signals were observed. Typical results are illustrated in Figure 2. The notable intercept in the bimolecular plots is essentially due to the use of HNO_3_ as the OH precursor. As was already discussed in Bänsch and Olzmann,^31^ the reaction of OH with NO_2_ impurities formed in the HNO_3_ storage cylinder is mainly responsible for this effect. We tried to minimize influences on the rate coefficients (the slope) by carefully keeping the HNO_3_ and hence the NO_2_ concentration constants within one series of measurements for differing MF concentrations. We also performed measurements with both increasing and decreasing MF concentrations and obtained, within the error margin, identical results; that is, no kind of hysteresis was observed. Moreover, we always freshly synthesized HNO_3_ and never used HNO_3_/He mixtures older than 7 days.

The purities of the chemicals used were as follows: CH_3_OCHO (Sigma-Aldrich, ≥99.0%), CH_3_OCDO (CDN Isotopes, 98.6%, 99.4%-d1), CD_3_OCHO (CDN Isotopes, 99.6%, 99.9%-d3), CD_3_OCDO (CDN Isotopes, 99.7%, 99.8%-d4), He (Air Liquide, >99.999%), H_2_SO_4_ (Roth, 98%), KNO_3_ (Roth, ≥99%).

### 2.3 Theoretical Calculations

To complement the experimental measurements, statistical rate theory calculations have been performed in the form of RRKM/ME (Rice, Ramsperger, Kassel, Marcus, Master Equation) calculations. For these calculations, the potential energy surface of the OH + MF reaction was mapped out at the CCSD(T)-F12/aug-cc-pVTZ//M06-2*X*/6-31+G** level of theory. In this case, the CCSD(T) energies were obtained using Molpro,^33^ while the DFT geometry optimizations and frequency calculations were performed in Gaussian.^34^ All DFT calculations utilized the “ultrafine” integration grid in Gaussian. For key species, the reactants, and the transition states, we have employed higher-level calculations based on the ANL schemes of Klippenstein and co-workers.^35^ For these calculations, the structures and obtained harmonic frequencies were reoptimized at the CCSD(T)-F12/cc-pVDZ-F12 level of theory. High-level single-point energies were obtained at these optimized structures using the following expression
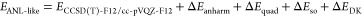
E4where
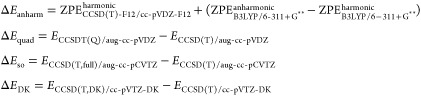


For anharmonic corrections to the zero-point vibrational energies, Δ*E*_anharm_, ZPEs were obtained using vibrational perturbation theory (VPT)^36^ as implemented in Gaussian.^34^ For these VPT calculations, the B3LYP/6-311+G** level of theory was used since previous work^35^ has indicated that there can be numerical instabilities at the M06-2X level.^37^ Higher-order excitations were corrected by taking the difference between CCSDT(Q)/aug-cc-pVDZ and CCSD(T)/aug-cc-pVDZ calculation using the MRCC code.^38^ Δ*E*_so_ and Δ*E*_DK_ refer to corrections for spin–orbit coupling and relativistic effects, respectively, and these calculations were performed in Molpro with DK specifying the inclusion of Douglas Kroll one-electron integrals.^39^ Compared with the original ANL approaches, our calculations are slightly less accurate. The main source of error is that we were unable to use basis sets quite as large as those in the ANL0 and ANL1 schemes, and we have made more extensive use of the explicitly correlated F12 methods to mitigate this.

For all of the species considered in this work, only the lowest-energy conformer was considered explicitly. Given the conformational complexity of the species involved in this reaction system, multidimensional torsional potentials were included and were utilized by the coupled classical rotors method implemented in MESMER. To generate these coupled potentials, 100 (two coupled rotors) or 1000 (three coupled rotors) constrained geometry optimizations were performed for a given species corresponding to 36° steps in each torsional coordinate for the coupled rotors. For each constrained optimization, the coupled torsional coordinates were held rigid. These coupled torsional potentials were fit with a multidimensional Fourier series using the ChemDyME package^40^ and the resulting potentials were read by the coupled rotor methods in MESMER to generate fully coupled torsional density of states. Full details of the Fourier series fitting are given in the Supporting Information.

The highest-level “ANL-like” calculations and multidimensional rotor calculations were only performed for the two transition states as, for the temperatures and pressures considered here, the kinetics of the system were found to be insensitive to the prereaction complexes in that a change in the well depth by ±4 kJ mol^–1^ had a negligible impact on the calculated rate coefficients. For methyl formate, both torsions were considered to be separable. For the formate group abstraction transition state (TS_CHO_), two coupled rotors were considered with the methyl rotor considered separable with a 1-D hindered rotor treatment, while for the methyl abstraction transition state (TS_CH3_), all three hindered rotors were considered coupled. All constrained optimizations for the hindered rotor calculations were performed at the M06-2*X*/6-31+G** level.

To consider tunneling a Wentzel–Kramers–Brillouin (WKB) approach,^41^ as implemented in MESMER, was used. The 1-D tunneling potentials were obtained from intrinsic reaction coordinate (IRC) calculations at the M06-2*X*/6-31+G** level of theory, and these potentials were corrected for ZPE effects using projected frequency calculations along the IRC. These tunneling potentials were scaled to the high-level energy differences between the transition states and their corresponding prereaction complex, and this scaling was maintained whenever transition state energies were altered in our master equation calculations such that the tunneling potential in MESMER was always consistent with the ZPE-corrected energy difference between prereaction complex and transition state.

In this work, MESMER was used to solve the energy grained master equation (EGME) for the OH + MF system. As a starting point, rovibrational densities of states were obtained under the rigid rotor harmonic oscillator approximation. Any rotatable bonds were additionally considered explicitly as 1-D or coupled hindered rotors, as described above. These hindered rotor motions were projected out of the molecular Hessian using the methodology of Sharma, Raman, and Green.^42^ We coupled the internal modes of a given species to the other internal modes of that species using the coupled classical rotors approach^43^ recently implemented in MESMER. This coupling considers how the vibrational modes of a species change upon rotation about an internal bond and this treatment negates the need for a multiconfigurational-type approach which approximates such coupling by considering different conformers of a species as distinct.^44^ Currently, our coupled rotor approach neglects coupling between internal rotor modes and other normal modes, and this is something we intend to add in the future. For the MESMER simulations, a grain size of 50 cm^–1^ was used and the energy span was truncated at 25 kJ mol^–1^ above the highest barrier in the system.

**Figure 3:**
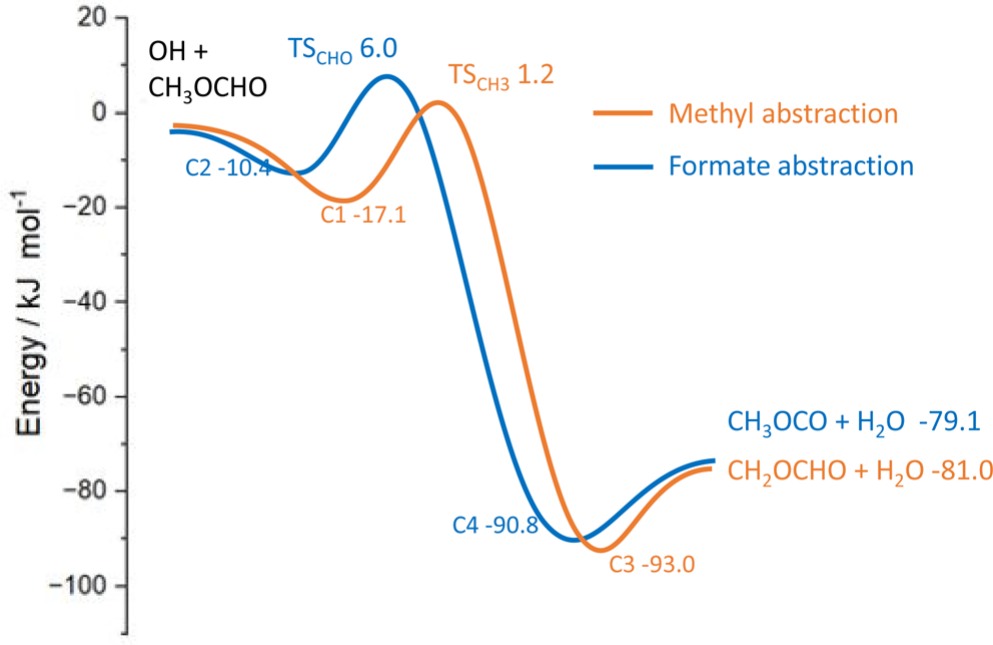
Zero-point energy-corrected potential energy surface for Reaction 1 calculated at the CCSD(T)-F12/aug-cc-pVTZ//M06-2*X*/6-31+G** level of theory. The barrier energies are from higher-level “ANL0-like” calculations.

Figure 3 shows the potential energy surface for Reaction 1 and the calculated effective barrier heights for the various isotopic combinations, as shown in Table 1. We estimate an error in our transition state energies of about approximately 1–2 kJ mol^–1^; however, given the extensive experimental data gathered in this work, we have refined these barriers even further by fitting them to the experimental data. For the fitting of the MESMER simulations to the experimental data for the isotopically different systems, the built-in Levenberg–Marquardt algorithm in MESMER could not be used as this could only deal with one isotopic system at a time. Instead, a bespoke Python script was used to simultaneously fit the TS energies in the MESMER input to the experimental data for all isotopic variants. This script ensures that the relative TS energies for the different isotopic substitutions remained constant as constrained by the difference in ZPEs from the CCSD(T)-F12 harmonic frequencies and the B3LYP anharmonic corrections. This Python script also uses a Levenberg–Marquardt approach and can be found in the Supporting Information.

**Table 1:** Calculated *Ab Initio* and Fitted Barriers for Reaction of OH/OD at the Methyl and Formate Sites of Methyl Formate

isotopologue combination	*ab initio* ZPE-corrected energy barrier at TS_CH3_/kJ mol^–1^	*ab initio* ZPE-corrected energy barrier at TS_CHO_/kJ mol^–1^
OH + CH_3_OCHO (MF) Fit to experimental data	0.91 ± 0.58 Leeds + KIT	4.06 ± 0.86 Leeds + KIT
	1.28	6.00
	1.22	10.1
	7.21	5.48
	7.19	9.59
	–0.75	4.52
	–0.82	8.58
	5.26	4.01
	5.24	8.11

## 3 Results and Discussion

**Figure 4:**
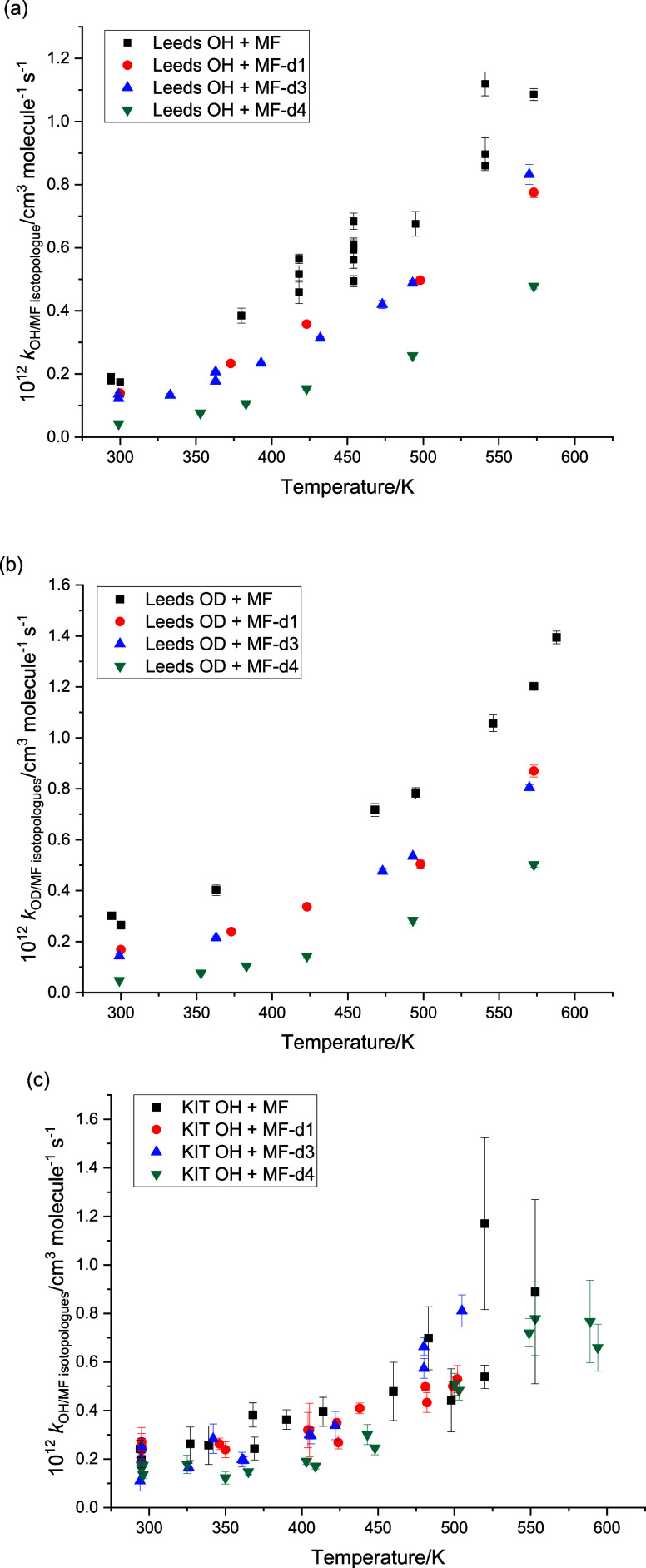
Temperature-dependent rate coefficients. (a) Leeds: OH+MF, MF-d1,3,4, and (b) Leeds: OD+MF, MF-d1,3,4, and (c) KIT: OH+MF, MF-d1,3,4.

Figure 4a-c shows the rate coefficient data for the reactions of OH/OD with the various isotopologues studies at Leeds and KIT. Tabulations of data (Tables S1–8) and a summary figure (Figure S2) of intercomparison of Leeds and KIT data can be found in the SI. In general, there is good agreement between the two studies, and for most analyses, both data sets have been used. An exception appears to be the KIT data for OH + MF-d4, where, especially at lower temperatures, there is little difference from the partially deuterated isotopologues in contrast to the Leeds data and expected effects from increased isotopic substitution (cf. Figure 4a,c and see Figure S2, bottom right). Therefore, in our analyses of the barrier heights and site-specific rate coefficients (section 3.2), the KIT data for OH + MF-d4 have been omitted.

### 3.1 Overall Kinetics of the OH/OD + MF Reactions

The room-temperature rate coefficient for the reaction of OH with CH_3_OCHO, *k*_OH/MF,298 K_, is in good agreement with most previous literature as shown in Table 2. The rate coefficient is low, and therefore, especially if low concentrations of methyl formate have been used, the pseudo-first-order rate coefficient will be small and OH traces can be susceptible to secondary, radical–radical chemistry. Although the absorption cross section at 248 nm is low, there is still some methyl formate photolysis at this laser wavelength.^45^ Preliminary studies at Leeds using a 248 nm excimer laser indicated some secondary chemistry in agreement with the work of Le Calvé et al.;^6^ experiments at Leeds therefore switched to 266 nm photolysis and only report data using this photolysis wavelength. At KIT, photolysis was performed at 248 nm, but no indications for the relevance of side reactions from MF photolysis products were found under these experimental conditions of significantly higher pressure. All decay curves were monoexponential, and variations of initial concentrations and laser fluences did not have any systematic effect on the rate coefficients obtained. Wallington et al.,^8^ who utilized water photolysis at ∼160 nm as the OH source, may have been additionally influenced by secondary chemistry, potentially explaining their higher value, which is, however, still within experimental error of the more recent studies. The flash photolysis work is in good agreement with the flowtube studies of Good et al.^9^ and Szilagyi et al.^7^ suggesting an absence of any significant systematic errors.

**Table 2:** Room-Temperature Rate Coefficients for the Reaction of OH with MF

reference	technique	10^13^ *k*_OH/MF,298 K_/cm^3^ molecule^–1^ s^–1^
Wallington et al.^8^	FP (∼160 nm) and RF detection of OH	2.27 ± 0.34
Le Clavé et al.^6^	LFP (248 or 351 nm) and LIF detection of OH	1.73 ± 0.21
Good et al.^9^	flowtube with RF detection of OH	1.77 ± 0.28
Szilagyi et al.^7^	flowtube with RF detection of OH	1.83 ± 0.33
JPL evaluation^46^	Review	1.8 ± 0.3
this work (Leeds)	LFP (266 nm) with LIF excitation at 282 or 308 nm and detection at 308 nm	1.81 ± 0.27^a^ (0.09)^b^
this work (KIT)	LFP (248 nm) with LIF excitation/detection at 282/308 nm	2.08 ± 0.62^a^ (0.18)^b^

a Total estimated error from the statistical analysis and from estimated systematic uncertainties.

b Statistical error at the 2σ level.

The weighted averaged overall rate coefficient, *k*_OH/MF,298 K_, determined in this study, (1.95 ± 0.34) × 10^–13^ cm^3^ molecule^–1^ s^–1^, is comparable to the value of 2.0 × 10^–13^ cm^3^ molecule^–1^ s^–1^ used in the master chemical mechanism (MCM)^47^ and hence widely used in tropospheric modeling studies.

**Figure 5:**
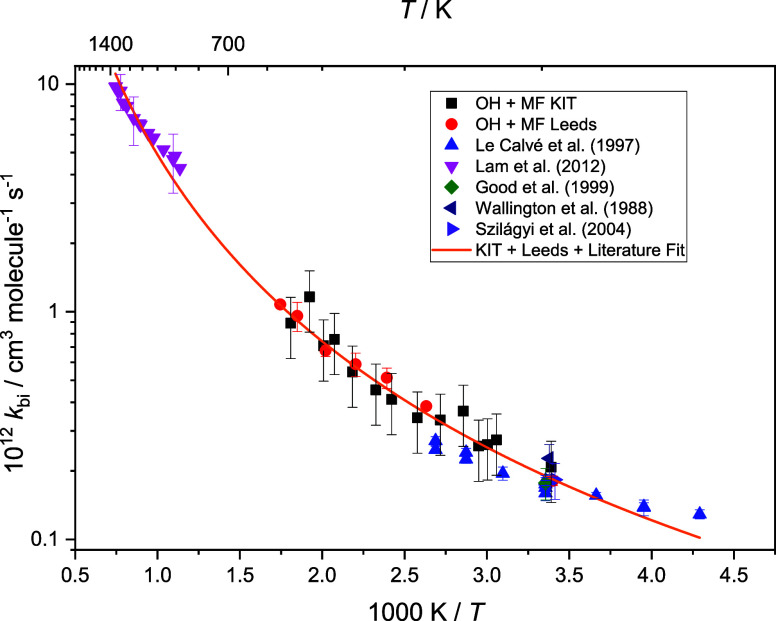
Arrhenius plot of R1_OH/MF_ including literature data; extended Arrhenius fit from eq E5.

Figure 5 shows an Arrhenius plot of literature data for R1_OH/MF_ and the results from this work. If the data sets of Le Calve et al., this work, and Lam et al. are individually analyzed with a simple Arrhenius expression, then there is a clear increase in the activation energies (3.64 ± 0.31, 8.78 ± 0.42, and 16.8 ± 0.7 kJ mol^–1^). Our data confirms curvature of the Arrhenius data for R1_OH/MF_ and *k*_OH/MF_ can be parametrized over the temperature range 233–1300 K as
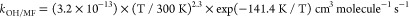
E5

The fits at the lowest temperature are not perfect; the lower-temperature data of Le Calve^6^ hint at the potential upturn in the rate coefficient at low temperatures. Further studies, ideally bridging toward the temperature range used by Jimenez et al.^11^ (64–22 K), would help to define the parametrization of R1 from 22 to 1371 K that will need to be more complex than eq E5.

**Figure 6:**
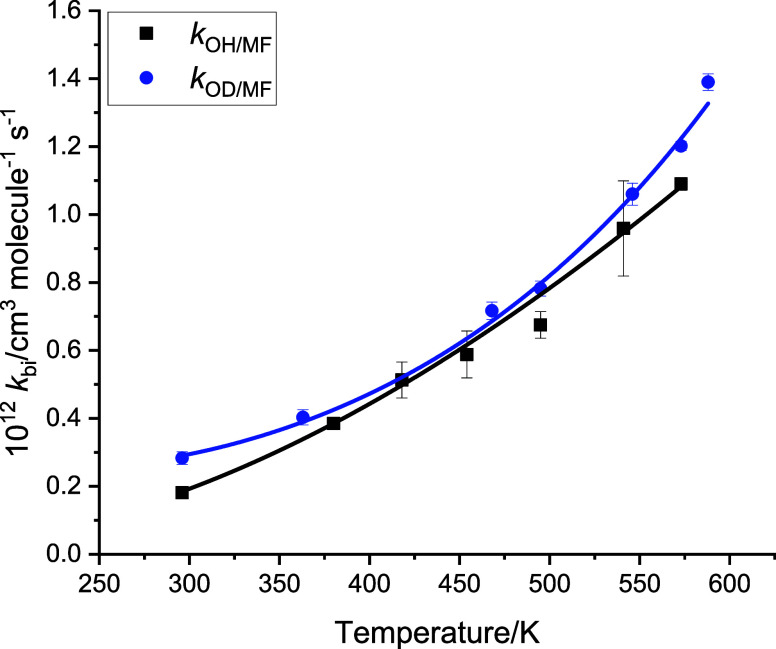
Temperature dependence for the reaction of OH and OD with MF from the University of Leeds. Data were recorded at pressures of 7–128 mbar with no significant pressure dependence observed. The solid lines are three-parameter fits to the data: *k*_OH/MF_ = 1.04 × 10^–12^ × (*T*/300)^1.43^ × exp(−504/*T*), *k*_OD/MF_ = 4.35 × 10^–15^ × (*T*/300)^5.30^ × exp(−1270/*T*) cm^3^ molecule^–1^ s^–1^.

Figure 6 shows the temperature dependence of the reaction of OH and OD with normal methyl formate.
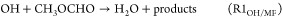

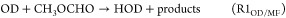


The data are also tabulated in the SI (Table S1). The results indicate a small enhancement of *k*_OD/MF_ over *k*_OH/MF_, i.e., an inverse secondary kinetic isotope effect (KIE) that is reproduced by the theoretical calculations that show a small reduction (1.5–2.0 kJ mol^–1^) in the activation barriers for *k*_OD/MF_. Calculated barrier heights relative to the entrance channel and including ZPE corrections are shown in Table 1 for all isotopologues. The implications of the secondary KIE on the structure of the transition states are discussed in Section 3.3. To the best of our knowledge, there have been no previous studies of reaction R1_OD/MF_.

### 3.2 Reactions of OH and OD with the Isotopologues MF-d1, MF-d3, and MF-d4 and Branching Ratios

**Figure 7:**
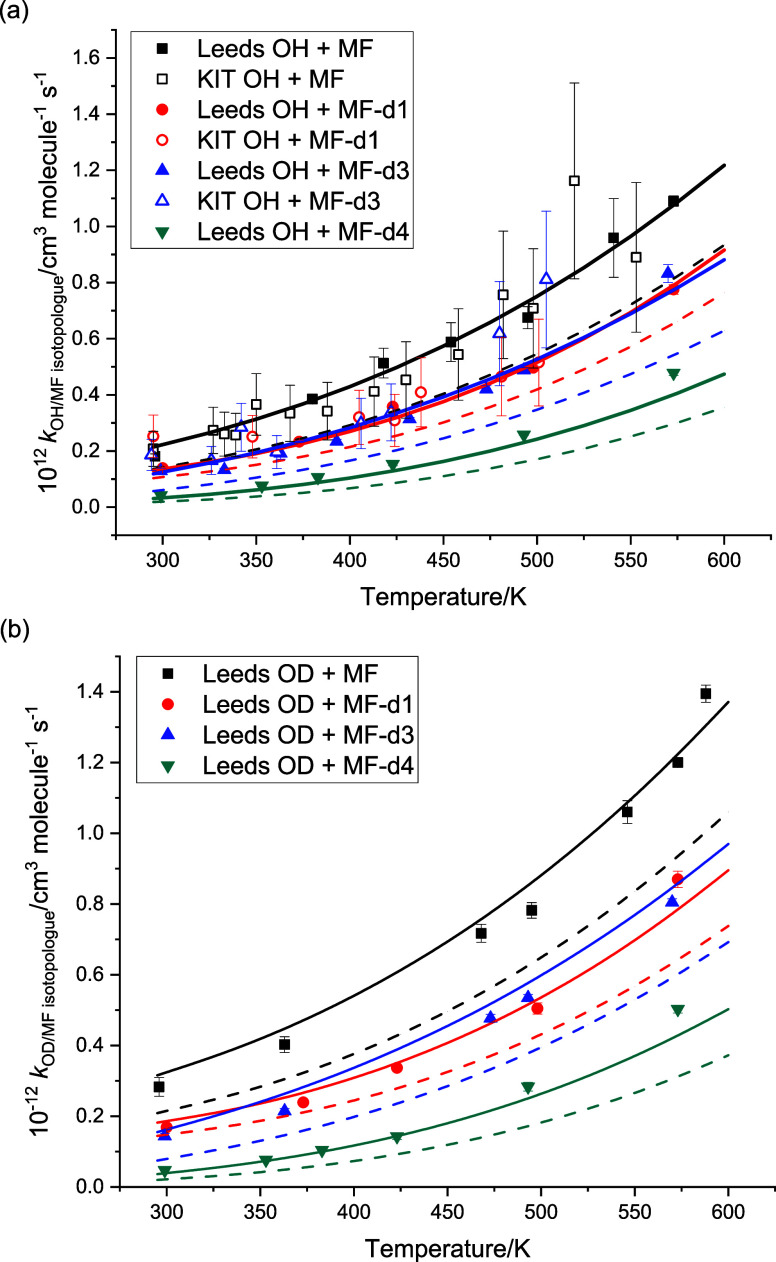
Summary of data for OH/OD + methyl formate isotopologues. The dashed lines are the *ab initio* calculations, and the solid lines are the adjusted fits, floating the barrier heights of the abstraction reactions. (a) Leeds and KIT data on OH + methyl formate isotopologues. (b) Leeds data on OD + methyl formate isotopologues.

Figure 7a,b shows summaries of the kinetic data on the reactions of OH and OD with the four isotopologues of MF. The slight positive secondary isotope effect for OD over OH is maintained across the four isotopologues. Increasing deuteration of methyl formate results in a decrease in the rate coefficient, i.e., a normal primary kinetic isotope effect.

Figure 7a,b also shows the results of the *ab initio* calculations and the fits to the data. The dotted lines represent the ab initio calculations with no adjustment of the barriers. The calculated barriers are given in Table 1, and the effective barrier heights increase sensibly with deuteration of methyl formate. The full lines represent the fits to the experimental data, where barrier heights for all OH and OD reactions have been simultaneously fitted. Note that as explained above, the relative differences in the barrier heights, due to the change in ZPE, have been maintained. The final fitted barriers are also given in Table 1. The change in barrier heights is small, <2 kJ mol^–1^, and on the order of the expected uncertainty in the original calculations. It should be noted that while the overall fit has been improved by varying barrier height, the variation in barrier height may be compensating for systematic errors in other parameters. The quoted errors on the fitted barrier heights are model-dependent and do not incorporate correlations between, e.g., the fitted barriers and the treatment of densities of states. The resulting branching ratios, *f*_CH_3__ and *f*_CHO_ (where, for example, ), are tabulated in Table 3.

**Table 3:** Branching Ratios for Abstraction at Methyl, *f*_CH3_, and Formate, *f*_CHO_, Sites in the Reaction of OH with MF

temperature/K	fraction of methyl abstraction, *f*_CH_3__	fraction of formate abstraction, *f*_CHO_
295	0.49	0.51
350	0.43	0.57
400	0.40	0.60
450	0.38	0.62
500	0.38	0.62
550	0.38	0.62
600	0.39	0.61

### 3.3 The PES for OH + MF and Discussion of the Mechanism of Reaction

The stationary points for the OH + MF system are shown in Figure 3. All energy barriers quoted here include ZPE. The relative stationary point energies at the CCSD(T)-F12 level are in good agreement with previous work from Wu et al.^20^ and Lins et al.^18^ (see Table 4), which would be expected based upon the similar levels of theory. Although our highest-level barrier heights are a little lower than the values from Wu et al. and Lins et al., the agreement is well within the combined theoretical uncertainties. Interestingly, both the energies presented here and the energies obtained by Wu et al. and Lins et al. are significantly lower than the barrier heights from the work of Tan et al.^19^ of 14.6 and 16.7 kJ mol^–1^ for TS_CH3_ and TS_CHO_, respectively. Tan et al. used multireference methods in their study that covered a range of radical + MF systems. However, for OH + MF, multireference methods are not necessary and the consideration of dynamical correlation via multireference methods in the Tan et al. work means that static correlation is treated more approximately than in the CCSD(T) or higher-level energies of this and other works. Our barriers are also lower than the work of Good et al.^9^ although the values from Good and co-workers are from earlier calculations and, consequently, at a lower level of theory which would not be expected to give quantitative accuracy. For the rest of the discussion presented herein, we will consider the system with “ANL-like” energies for the two TS.

**Table 4:** Comparison of Barrier Heights for Abstraction at the Methyl and Formate Sites from This Work and the Literature

reference	method	barrier height TS_CH3_/kJ mol^–1^	barrier height TS_CHO_/kJ mol^–1^
this work	CCSD(T)-F12/aug-cc-pVTZ//M06-2*X*/6-31+G**	4.9	6.9
this work	ANL-like	1.2	6.0
this work	fit to experimental data	0.91 ± 0.58	4.06 ± 0.86
Lins et al.^18^	M05-2X/ma-TZVP	7.4	12.6
Wu et al.^20^	M06-2X/ma-TZVP	3.3	5.9
Tan et al.^19^	MRSDCI+DS(MRACPF)/cc-pV∞Z//B3LYP/cc-pVTZ	14.6	16.7
Good et al.^9^	G2	16.7	10.0

**Figure 8:**
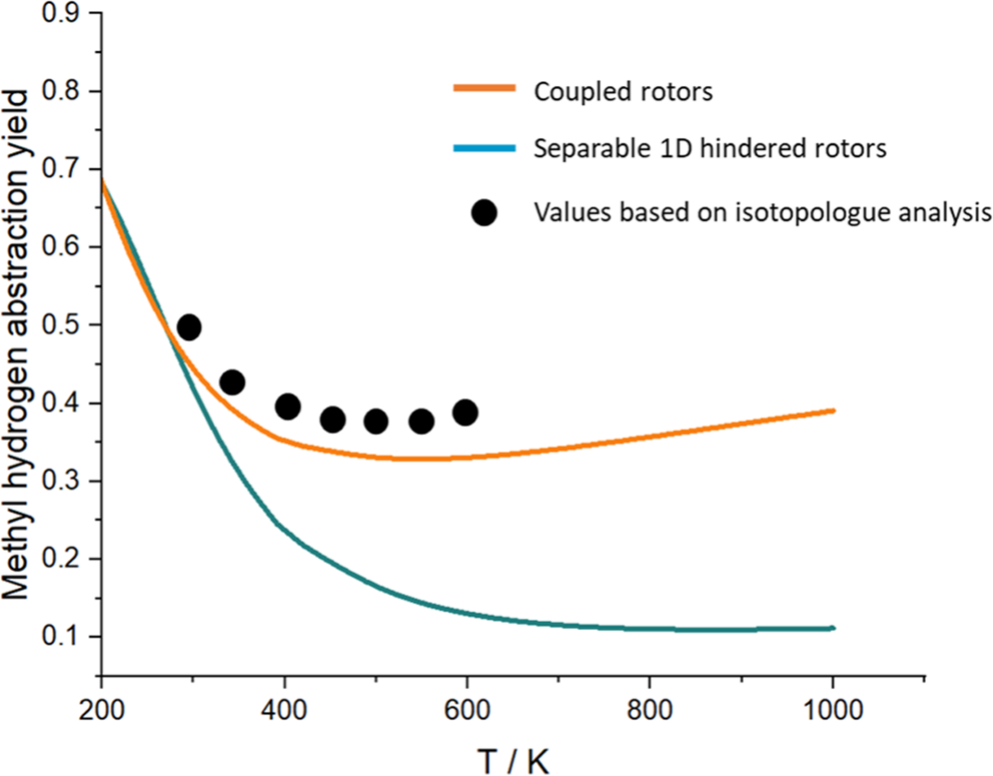
Calculated yield of methyl abstraction as a function of the temperature. The points are the isotopologue analysis results from Table 3.

**Figure 9:**
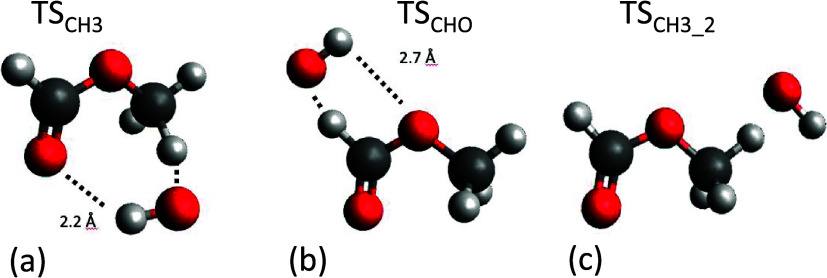
Structures of the two abstraction transition states at the M06-2*X*/6-31+G** level of theory and the second lowest-energy conformer for TS_CH3_2_. Also indicated are the longer H – O hydrogen bond lengths in Å for TS_CH_3__ and TS_CHO_.

From the *ab initio* calculations and master equation fits to the experimental data, we can start to examine the contributions from different reaction channels. Figure 8 shows the methyl abstraction yield as a function of temperature for the undeuterated system. Abstraction at the methyl site is favored at low temperatures due to the smaller activation barrier for this channel, where the TS is stabilized by the formation of a ring structure as shown in Figure 9a. However, the stabilization of TS_CH3_ comes with an entropic cost. Abstraction at the formate site (Figure 9b) has a higher barrier but is also entropically favored due to the potential for methyl rotation. One would therefore expect abstraction at the formate site to become increasingly favored at higher temperatures.

To understand the differing entropic penalties (*A* factors) of the two TS, it is worth examining the structures (Figure 9 and torsional potentials in the SI) in more detail. It can be seen that TS_CH3_ forms a pseudo-ring structure via the formation of two hydrogen bonds, one at each end of the methyl formate backbone. On the other hand, for TS_CHO_, the second H bond is much longer range (2.7 vs 2.2 Å) and consequently, the OH and CH_3_ moieties can rotate with less hindrance. Considering these lowest-energy conformations only, it can readily be rationalized that the lower-energy, methyl transition state has a higher entropic penalty to reaction causing the observed increase in the formate channel yield as temperature increases.

The structures of the TS are consistent with the significant secondary isotope effect reported in Section 3.1. The secondary isotope effect suggests that the OH/OD bond is not a simple spectator moiety in the transition state, where the ZPE of OH/OD would be essentially the same in the reactant and TS so that there is no secondary isotope effect, but rather that OH/OD is involved in the TS structure. Upon examination of the vibrational frequencies of the OH and OD variants of each TS, it is found that, in addition to the OH/OD stretching mode, there are small but significant differences in many of the lower frequency modes that correspond to larger-amplitude bending motions which incorporate the OH/OD moiety. These small variations cumulatively give the 1–2 kJ mol^–1^ difference we observe between the OH and OD barrier heights.

Figure 8 shows that the calculated branching ratios level off at approximately 400 K. These observations are consistent with previous calculations performed using a multiconformer approach^20^ wherein the different conformers of the saddle points were treated explicitly and are also supported by experimental determinations of the branching ratio which we present in a companion paper. Interestingly, this leveling off is not predicted unless the fully coupled torsional surface is considered (see Figure 8) and the leveling off in the branching ratios is related to a higher-entropy methyl abstraction saddle point conformation which becomes available at higher temperatures. This conformation is accessed by rotation in two torsional coordinates and thus cannot be treated properly with a 1-D hindered rotor model. However, the 2-D and 3-D torsional potential energy surfaces used here cover the full conformational space of each saddle point and negate the need to use a multiconformer approximation. The second conformer of the methyl transition state is also shown in Figure 9c, and it can be seen that this is a far more flexible structure with only one hydrogen bond, in contrast to the more rigid, ring-like structure of TS_CH3_.

Previous work of Wu et al.^20^ and Lins et al.^18^ utilized multistructural or multiconformer-type approaches that have become popular in recent years. Such approaches are approximate in their treatment of hindered rotation but offer a computationally efficient way of capturing the coupling between different torsional degrees of freedom and also the coupling between torsions and other normal modes. With the increased accuracy in barrier heights afforded by ANL0-type approaches, recently Klippenstein^48^ has demonstrated that a coupling scheme with explicit torsional potentials is the way to achieve comparable accuracy in the densities of states. We followed this type of approach. Our coupling scheme currently couples only the torsional degrees of freedom, which we believe to be the largest contribution to the coupling between conformers. Qualitatively the branching ratios here agree with the trends of Wu et al.^20^ and Lins et al.^18^

**Figure 10:**
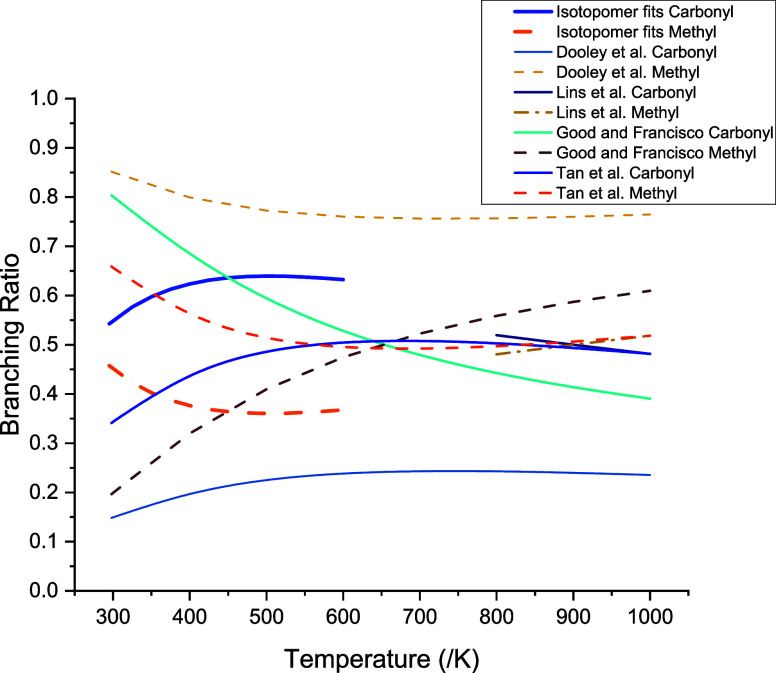
Branching ratios for the abstraction by OH at carbonyl (solid lines) and methyl (dashed lines) sites of methyl formate.

Figure 10 summarizes experimental, theoretical, and modeled values of the branching ratios as a function of temperature.

## 4 Implications

### 4.1 Atmospheric Oxidation

The initiation of the atmospheric oxidation of methyl formate is dominated by the reaction with OH, proceeding via hydrogen abstraction from the methyl (R1_CH_3__) or carbonyl group (R1_CHO_). We will first consider carbonyl abstraction forming CH_3_OCO. Subsequent O_2_ addition forms CH_3_OC(O)OO (R3b). In the presence of NOx, this reacts with NO to form the alkoxy radical CH_3_OC(O)O (R5b), the sole fate of which is decomposition to CO_2_ and CH_3_O (R6) (in turn giving HCHO + HO_2_ (R7)), as identified in previous product studies.^17,49^ Wallington et al.^17^ also observed minor production of CH_3_OC(O)O_2_NO_2_ from the reaction of CH_3_OC(O)OO with NO_2_.
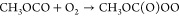
R3b
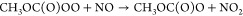
R5b
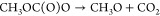
R6
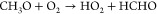
R7

The master chemical mechanism (MCM)^47^ is a comprehensive model for hydrocarbon oxidation, extensively used in the community. The MCM has CH_3_OCO being formed from reaction R1 with a yield of 0.45, slightly less than that in this work (0.51) with all CH_3_OCO undergoing decomposition to CH_3_ + CO_2_ (R2). Under high NOx conditions, this disparity in mechanism for CH_3_OCO will have limited effect as the first-generation products will still be HCHO + CO_2_ + HO_2_ and one conversion of NO to NO_2_ via the following chemistry
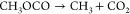
R2
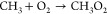
R8
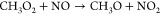
R9
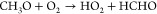
R7However, under low-NOx conditions, prompt decomposition of CH_3_OCO ignores the radical–radical chemistry that CH_3_OC(O)OO could participate in. For example, reaction with HO_2_ could regenerate OH in processes analogous to the reaction of CH_3_C(O)O_2_ + HO_2_ ^50^
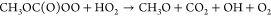
R10

Abstraction at the methyl site produces some slightly more complex chemistry. The OCH_2_OCHO radical formed following oxygen addition and subsequent reaction with NO undergoes α-ester rearrangement, whereby a hydrogen atom is transferred to the carbonyl oxygen in a five-membered transition state, to form formic acid (R11) in competition with hydrogen abstraction by O_2_ to form formic acid anhydride (R12).^49^
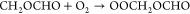
R3a
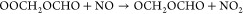
R5a
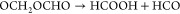
R11
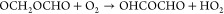
R12

Wallington et al.^17^ used Cl atom chemistry to initiate the oxidation; they found the sum of formic acid and formic acid anhydride to be consistent with the 45% branching ratio of methyl abstraction by Cl, suggesting that these are the major products following CH_2_OCHO formation.

Tyndall et al.^49^ determined the branching ratio for the reaction of OH with methyl formate occurred approximately 50:50 at each site of attack, ranging from 60:40 to 40:60. A branching ratio of 54% abstraction at the carbonyl group from this work agrees well with Tyndall et al. Assuming methyl formate is emitted in environments containing NO, which is highly probable if combustion is the source, 54% of the methyl formate reaction with OH will become formaldehyde and carbon dioxide (R1_CHO_, and CH_3_OCO oxidation), 36% will form formic acid anhydride (R1_CH_3__ and reaction of OCH_2_OCHO with O_2_), and 18% will form formic acid (R1_CH_3__ and OCH_2_OCHO α-ester rearrangement).^17^

There are significant uncertainties in the budgets of HCOOH production and loss in the atmosphere.^16^ Determinations of branching yields for the reaction of OH with methyl formate help constrain the budget for HCOOH production from methyl formate, a source of secondary HCOOH production and one that might increase in importance if methyl formate finds use a hydrogen carrier.^3^

### 4.2 Low-Temperature Combustion

Abstraction at the methyl position under low-temperature combustion (LTC) conditions leads to CH_2_OCHO, and subsequently, there will be a competition between O_2_ addition and decomposition to formaldehyde and HCO.
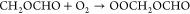
R3a
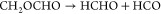
R13

OOCH_2_OCHO can undergo RO_2_ – QOOH chemistry (R14), but the QOOH radical formed, HOOCH_2_OCO, is very susceptible to decomposition (R15), analogous to other QOOH radicals with an ether functionality,^51^ rather than further O_2_ addition and classical low-temperature chain branching.
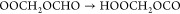
R14
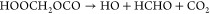
R15

Abstraction at the carbonyl group produces CH_3_OCO and under typical LTC conditions dissociation to CH_3_ + CO_2_ will be the dominant channel and CH_3_OCO will not take part in any RO_2_ – QOOH chemistry to any significant extent.

The limited formation of any significant quantities of the chain branching precursors O_2_QOOH in low-temperature, methyl formate combustion means that there is no significant negative temperature coefficient (NTC) behavior for methyl formate. Minwegen et al.^21^ performed some sensitivity analysis on their model of methyl formate LTC. Abstraction at the methyl site promotes reaction, primarily because the decomposition of HCO, formed in reaction R13, leads to H atoms and chain branching via the H + O_2_ reaction. Conversely, abstraction at the carbonyl site decreases the reactivity. Clearly, the branching ratio of reaction R1 is a key parameter in modeling. Figure 10 shows the wide variation in reported branching ratios in the LTC region. Our value of the branching ratio for methyl abstraction at 1000 K, 0.40, based on the validated PES from the OH + methyl formate isotopologues and OH yield studies from 300–600 K, to be presented in subsequent paper, is significantly lower than the predictions of Dooley et al. (0.76) and slightly below the values of Lins et al. (0.52) and Tan et al. (0.52).

### 4.3 Chemically Activated Decomposition of CH_3_OCO

The role of chemical activation is central to unimolecular processes,^52^ but has more recently been considered in prototypical bimolecular abstraction reactions where the reaction exothermicity may be sufficient to cause fragmentation of one of the products, e.g., refs (22,53,54). A key problem is partitioning the overall reaction exothermicity between the products. A variety of approaches have been taken ranging from purely statistical,^55^ to a modified prior distribution,^53^ to molecular dynamics simulations.^56^

The CH_3_OCO radical, formed from reaction R1 is an ideal candidate for chemically activated decomposition, referred to as “hot β-scission”^21^ and, as discussed above, the MCM assumes 100% CH_3_OCO decomposition under atmospheric conditions. In the presence of oxygen, OH radicals can be recycled via both abstraction channels, e.g., for formate abstraction:
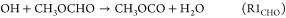

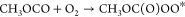
R3c
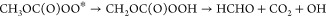
R16
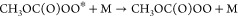
R17

The competition between R16 and R17 means that recycling decreases with pressure, but the yield should be unity if extrapolated to zero pressure. Chemically activated decomposition of CH_3_OCO would reduce the yield of recycled OH to below unity at zero pressure as OH is not recycled from CH_3_ + O_2_.

Our analysis, presented in future work but discussed here to bring together all aspects of reaction R1, provides no evidence for any chemically activated decomposition of CH_3_OCO at room temperature. At higher temperatures, our Stern–Volmer intercepts for OH recycling in the presence of excess O_2_ do increase above unity, but thermal decomposition of CH_3_OCO is rapid and we cannot differentiate between thermally and chemically activated CH_3_OCO decomposition. Minwegen et al.^21^ have considered the role of hot β-scission of CH_3_OCO in their combined experimental and theoretical study of low-temperature MF combustion. They predict a significant role for the chemically activated process; however, as the products are the same as the rapid thermal decomposition, the impact of considering hot β-scission is limited for this system.

## 5 Summary

Overall kinetics and branching ratios for the reaction of OH/OD with various methyl formate isotopologues were determined with two different laser flash photolysis systems. The overall rate coefficient demonstrates significant curvature as a function of temperature, and now previous low-temperature and shock tube studies are reconciled. The overall rate coefficient can be parametrized as *k*_1_ (*T*) = (3.2 × 10^–13^) × (*T*/300 K)^2.3^ × exp(−141.4 K/*T*) cm^3^ molecule^–1^ s^–1^. The overall estimated uncertainty in the rate coefficient is 30%.

The rate coefficients of the various isotopologues have been analyzed to determine the branching ratios for OH abstraction. The results indicate the importance of considering multiple conformers of the transition state, and we have described how this is implicitly treated within MESMER.

The fitted barriers for abstraction at the methyl and carbonyl sites are in good agreement with the high-level *ab initio* calculations. However, because the barriers are similar in magnitude, slight variations in barrier heights significantly alter predicted branching ratios demonstrating the importance of experimental measurements, even when high-quality calculations are available.

The implications of the branching ratios of reaction R1 on atmospheric and combustion chemistry are important, as is the case for many other OH abstraction reactions. Structure–activity relationships (SAR) are useful tools to predict site-specific reactivity; however, the performance of SAR for oxygenated species is often poor. This work and further site-specific studies are important in providing data for more reliable SAR construction.

Finally, abstraction from the carbonyl site generates the relatively weakly bound CH_3_OCO radical, which has been proposed to undergo chemically activated decomposition to CH_3_ + CO_2_. No evidence of this process was observed at room temperature. At higher temperatures, chemically activated decomposition may play a role, but thermal decomposition is already fast.

## Supporting Information Available

Example of biexponential analysis of OH decays, tabulations of experimental data, information on multidimensional torsional potentials, XYZ coordinates of calculated molecules, ANLO energy contributions, python script for simultaneous MESMER fits, and an example MESMER input file (PDF)
