# Supplementary material for: Temperature-Dependent, Site-Specific Rate Coefficients for the Reaction of OH (OD) with Methyl Formate Isotopologues via Experimental and Theoretical Studies
Source: J Phys Chem A. 2024 Jun 17;128(25):5028–40. doi: 10.1021/acs.jpca.4c02524 (PMC11215782; doi:10.1021/acs.jpca.4c02524)
Supplement: Supplementary file 1 — jp4c02524_si_001.pdf [file jp4c02524_si_001.pdf]

Temperature Dependent, Site-Specific Rate Coefficients for the Reaction of OH (OD) with Methyl Formate Isotopologues via Experimental and Theoretical Studies

Niamh C. K. Robertson[1], Lavinia Onel[1], Mark A. Blitz[1,2], Robin Shannon[1], Daniel Stone[1] and Paul W. Seakins[1]*
Struan H. Robertson[3]
Christian Kühn[4], Tobias M. Pazdera[4], Matthias Olzmann[4]*
1 - School of Chemistry, University of Leeds, Leeds, LS2 9JT, UK
2 - National Centre for Atmospheric Science, University of Leeds, Leeds, LS2 9JT, UK
3 - Dassault Systèmes, 22 Cambridge Science Park, Cambridge, CB4 0FJ, UK
4 - Institut für Physikalische Chemie, Karlsruher Institut für Technologie (KIT), 76131 Karlsruhe, Germany

Supplementary Information

Contents

1. Example of biexponential analysis
2. Tabulations of experimental data and intercomparisons
3. Multidimensional torsional potentials
4. XYZ coordinates.
5. ANLO energy contributions
6. Python script for simultaneous MESMER fits
7. Example MESMER input

1. Example of Biexponential Analysis

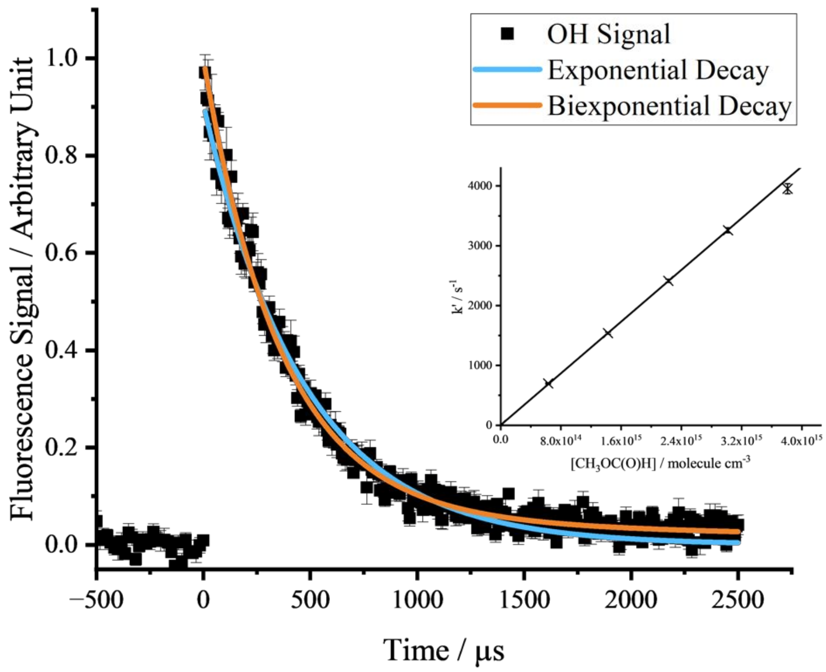

Figure S1. Biexponential decay following the OH initiated oxidation of 2.23 × 10^{15} molecule cm^{-3} methyl formate at 80 Torr and 573 K. The inset shows the corresponding bimolecular plot.

2. Tabulation of data and intercomparison of Leeds and KIT data

Table S1 – Rate coefficients for the reaction of OH and OD with CH_3OCHO (Leeds)

Temperature / K	Pressure / Torr	10^{13} k_{OH} / cm^3 $molecule^{-1}$ s^{-1}	10^{13} k_{OD} / cm^3 $molecule^{-1}$ s^{-1}
294	90	1.79 ± 0.06	
294	90	1.91 ± 0.07	
294	30		3.01 ± 0.13
300	85	1.74 ± 0.04	2.64 ± 0.08
363	25		4.03 ± 0.22
380	10.5	3.84 ± 0.07	
380	15	3.85 ± 0.24	
418	20	5.17 ± 0.26	
418	10	4.59 ± 0.37	
418	6	5.65 ± 0.15	
454	15.5	6.08 ± 0.17	
454	10.5	6.84 ± 0.26	
454	5	5.93 ± 0.38	
454	25	4.94 ± 0.18	
454	20	5.62 ± 0.28	
468	25		7.17 ± 0.25
495	50	6.75 ± 0.39	7.82 ± 0.22
541	17	11.20 ± 0.38	
541	24	8.96 ± 0.52	
541	50	8.60 ± 0.07	
546	30		10.60 ± 0.32
573	80	10.90 ± 0.19	10.20 ± 0.13
588	30		13.90 ± 0.25

Table S2 – Rate coefficients for the reaction of OH and OD with CH_3OCDO (Leeds)

Temperature / K	Pressure / Torr	10^{13} k_{OH} / cm^3 $molecule^{-1}$ s^{-1}	10^{13} k_{OD} / cm^3 $molecule^{-1}$ s^{-1}
300	85	1.39 ± 0.04	1.68 ± 0.02
373	92	2.33 ± 0.06	2.39 ± 0.03
423	88	3.58 ± 0.05	3.37 ± 0.03
498	85	4.96 ± 0.09	5.05 ± 0.16
573	91	7.75 ± 0.17	8.70 ± 0.23

Table S3 – Rate coefficients for the reaction of OH and OD with CD_3OCHO (Leeds)

Temperature / K	Pressure / Torr	10^{13} k_{OH} / cm^3 $molecule^{-1}$ s^{-1}	10^{13} k_{OD} / cm^3 $molecule^{-1}$ s^{-1}
299	86	1.36 ± 0.05	
299	97	1.23 ± 0.05	1.44 ± 0.08
333	86	1.33 ± 0.05	
363	88	2.07 ± 0.06	
363	92	1.77 ± 0.05	2.15 ± 0.07
393	87	2.34 ± 0.08	
432	76	3.14 ± 0.05	
473	92	4.20 ± 0.13	4.77 ± 0.11
493	86	4.88 ± 0.09	5.35 ± 0.09
570	90	8.32 ± 0.32	8.05 ± 0.10

Table S4 – Rate coefficients for the reaction of OH and OD with CD_3OCDO (Leeds)

Temperature / K	Pressure / Torr	10^{14} k_{OH} / cm^3 $molecule^{-1}$ s^{-1}	10^{14} k_{OD} / cm^3 $molecule^{-1}$ s^{-1}
299	79	4.21 ± 0.03	4.73 ± 0.05
353	80	7.64 ± 0.35	7.66 ± 0.51
383	76	10.60 ± 0.17	10.40 ± 0.14
423	80	15.30 ± 0.58	14.30 ± 0.89
493	80	25.80 ± 0.18	28.30 ± 1.23
573	76	47.80 ± 0.89	50.30 ± 1.09

Table S5 – Rate coefficients for the reaction of OH with CH_3OCHO from single measurement series (KIT). The error margin of the rate coefficients represents two standard deviations as obtained from the corresponding bimolecular plot.

***T* / K**	***p* / bar**	**10^{13} *k* / cm^3 s^{-1}**
294 ± 1	2	2.42 ± 0.36
295 ± 1	2	1.96 ± 0.31
327 ± 3	2	2.63 ± 0.69
339 ± 3	2	2.57 ± 0.79
368 ± 1	2	3.82 ± 0.50
369 ± 1	2	2.43 ± 0.47
390 ± 1	2	3.63 ± 0.40
414 ± 1	2	3.95 ± 0.60
460 ± 2	2	4.79 ± 1.20
483 ± 1	2	6.98 ± 1.30
498 ± 1	2	4.42 ± 1.29
520 ± 1	2	11.73 ± 3.54
520 ± 1	2	5.39 ± 0.48
553 ± 1	2	8.90 ± 3.83
294 ± 1	5	2.23 ± 0.66
296 ± 1	5	1.74 ± 0.53
326 ± 1	5	2.84 ± 0.40
333 ± 1	5	2.69 ± 0.31
348 ± 4	5	4.71 ± 2.06
354 ± 1	5	2.04 ± 1.14
366 ± 1	5	3.79 ± 0.39
386 ± 2	5	2.65 ± 0.77
388 ± 1	5	3.98 ± 0.31
413 ± 1	5	4.28 ± 0.90
455 ± 1	5	4.98 ± 0.45
459 ± 1	5	7.08 ± 1.25
482 ± 1	5	8.15 ± 2.97
499 ± 1	5	7.65 ± 0.97
519 ± 1	5	14.67 ± 2.95
523 ± 1	5	14.70 ± 3.17
295 ± 1	10	2.45 ± 0.56
296 ± 1	10	2.14 ± 0.43
296 ± 1	10	1.61 ± 0.31
333 ± 1	10	2.52 ± 0.62
347 ± 2	10	4.50 ± 1.12
351 ± 2	10	3.39 ± 0.54
430 ± 2	10	4.54 ± 0.56
457 ± 4	10	4.90 ± 0.74
498 ± 2	10	9.18 ± 1.97

Table S6 – Rate coefficients for the reaction of OH with CH_3OCDO from single measurement series (KIT). The error margin of the rate coefficients represents two standard deviations as obtained from the corresponding bimolecular plot.

***T* / K**	***p* / bar**	**10^{13} *k* / $cm^3\ s^{-1}$**
295 ± 1	2	2.55 ± 0.30
295 ± 1	2	2.71 ± 0.59
295 ± 1	2	2.47 ± 0.58
295 ± 1	2	2.36 ± 0.34
346 ± 3	2	2.63 ± 0.23
350 ± 3	2	2.39 ± 0.33
404 ± 2	2	3.20 ± 0.73
405 ± 1	2	3.20 ± 1.13
423 ± 2	2	3.50 ± 0.15
424 ± 1	2	2.68 ± 0.26
438 ± 3	2	4.09 ± 0.22
481 ± 2	2	4.98 ± 0.15
482 ± 1	2	4.33 ± 0.41
499 ± 3	2	5.00 ± 0.41
502 ± 2	2	5.30 ± 0.56

Table S7 – Rate coefficients for the reaction of OH with CD_3OCHO from single measurement series (KIT). The error margin of the rate coefficients represents two standard deviations as obtained from the corresponding bimolecular plot.

***T* / K**	***p* / bar**	**10^{13} *k* / $cm^3\ s^{-1}$**
294 ± 1	2	1.10 ± 0.41
295 ± 1	2	1.97 ± 0.34
295 ± 1	2	2.51 ± 0.35
326 ± 2	2	1.66 ± 0.16
342 ± 3	2	2.84 ± 0.61
361 ± 2	2	1.98 ± 0.30
362 ± 2	2	1.95 ± 0.14
405 ± 3	2	3.00 ± 0.17
406 ± 1	2	2.96 ± 0.33
422 ± 5	2	3.38 ± 0.58
480 ± 3	2	6.63 ± 0.36
480 ± 1	2	5.74 ± 0.41
505 ± 4	2	8.11 ± 0.66

Table S8 – Rate coefficients for the reaction of OH with CD_3OCDO from single measurement series (KIT). The error margin of the rate coefficients represents two standard deviations as obtained from the corresponding bimolecular plot.

***T* / K**	***p* / bar**	**10^{13} *k* / cm^3 s^{-1}**
295 ± 1	2	1.61 ± 0.16
295 ± 1	2	1.75 ± 0.24
296 ± 1	2	1.35 ± 0.14
296 ± 1	2	1.74 ± 0.38
296 ± 1	2	1.67 ± 0.44
325 ± 4	2	1.78 ± 0.38
350 ± 2	2	1.23 ± 0.26
365 ± 4	2	1.48 ± 0.11
403 ± 3	2	1.91 ± 0.13
409 ± 5	2	1.71 ± 0.12
443 ± 4	2	3.01 ± 0.41
448 ± 1	2	2.46 ± 0.28
500 ± 4	2	5.08 ± 0.45
503 ± 3	2	4.83 ± 0.40
549 ± 4	2	7.20 ± 0.58
553 ± 2	2	7.79 ± 1.52
589 ± 6	2	7.67 ± 1.71
594 ± 2	2	6.59 ± 0.97

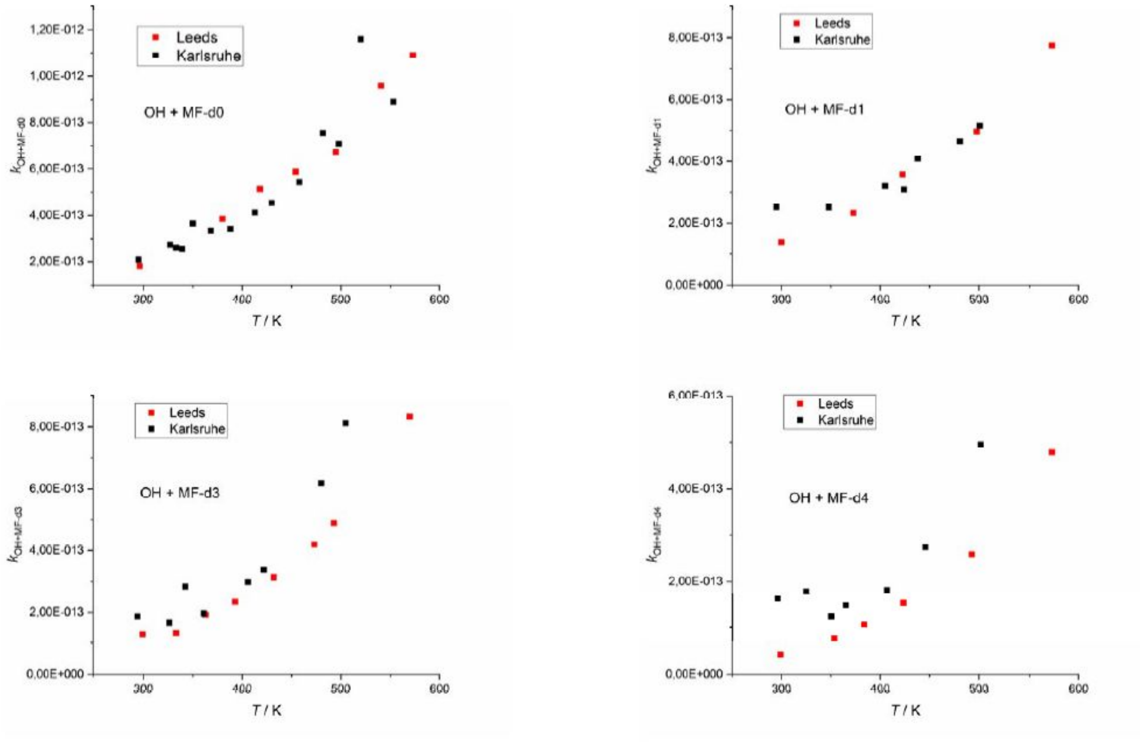

Figure S2. Summary intercomparison of Leeds and KIT data.

3. Multi-dimensional torsional potentials

The coupled torsional potentials used in this work are fit to multi-dimensional Fourier series. The 2D form is:

$$f(x,y) = \frac{1}{4}a_{00} + \frac{1}{2}\sum_{m=1}^{\infty}\left(a_{0m}\cos(my) + b_{0m}\sin(my)\right)$$
$$+ \frac{1}{2}\sum_{n=1}^{\infty}\left(a_{n0}\cos(nx) + b_{n0}\sin(nx)\right)$$
$$+ \sum_{m=1}^{\infty}\sum_{n=1}^{\infty}\left(a_{nm}\cos(nx)\cos(my) + b_{nm}\cos(nx)\sin(my)\right.$$
$$\left. + c_{nm}\sin(nx)\cos(my) + d_{nm}\sin(nx)\sin(my)\right)$$

Where:

$$a_{nm} = \frac{1}{\pi^2}\int_{-\pi}^{\pi}\int_{-\pi}^{\pi} f(x,y)\cos(nx)\cos(my)\,dx\,dy$$

$$b_{nm} = \frac{1}{\pi^2}\int_{-\pi}^{\pi}\int_{-\pi}^{\pi} f(x,y)\cos(nx)\sin(my)\,dx\,dy$$

$$c_{nm} = \frac{1}{\pi^2}\int_{-\pi}^{\pi}\int_{-\pi}^{\pi} f(x,y)\sin(nx)\cos(my)\,dx\,dy$$

$$d_{nm} = \frac{1}{\pi^2}\int_{-\pi}^{\pi}\int_{-\pi}^{\pi} f(x,y)\sin(nx)\sin(my)\,dx\,dy$$

The 3D version of this potential follows the same series but for brevity is not shown in full here. All coefficients were obtained via numerical integration in python (ChemDyME code) over the discrete points on the *ab initio* potentials.

The torsional potentials are shown below. Figure S3 shows the coupled torsional potential for two dihedral angles TS_{ald} along with the structure of the TS with atom labelling to identify the dihedral angles.

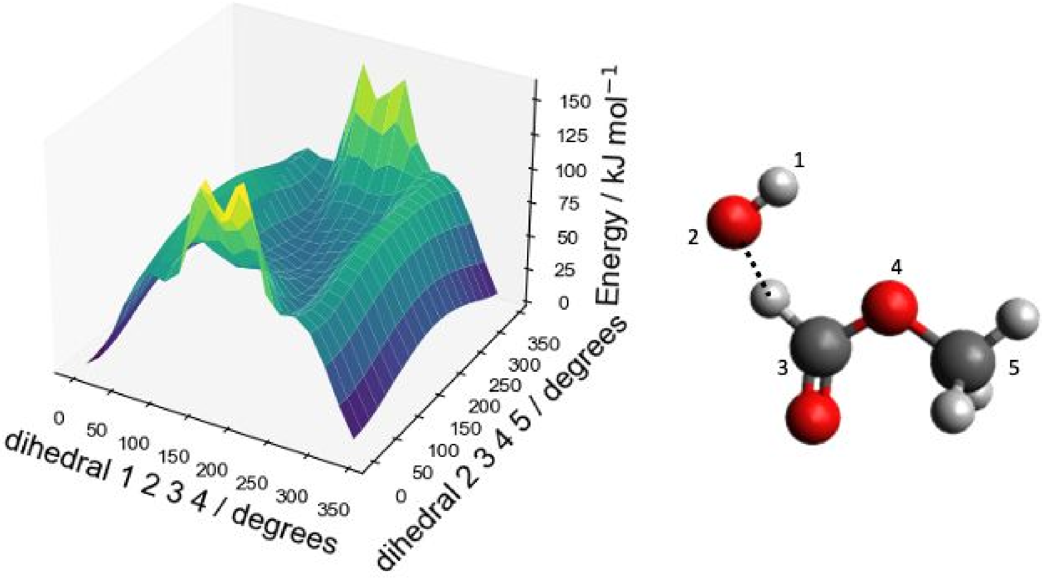

Figure S3. Torsional potential for the two coupled rotors in TS_{ald}. The atom numbering identifies the torsional angles corresponding to each rotor.

For TS_{methyl} there are three coupled rotors and Figures S4 - 6 show potentials for two of these dihedrals with the third held constant at its value in the optimised TS structure.

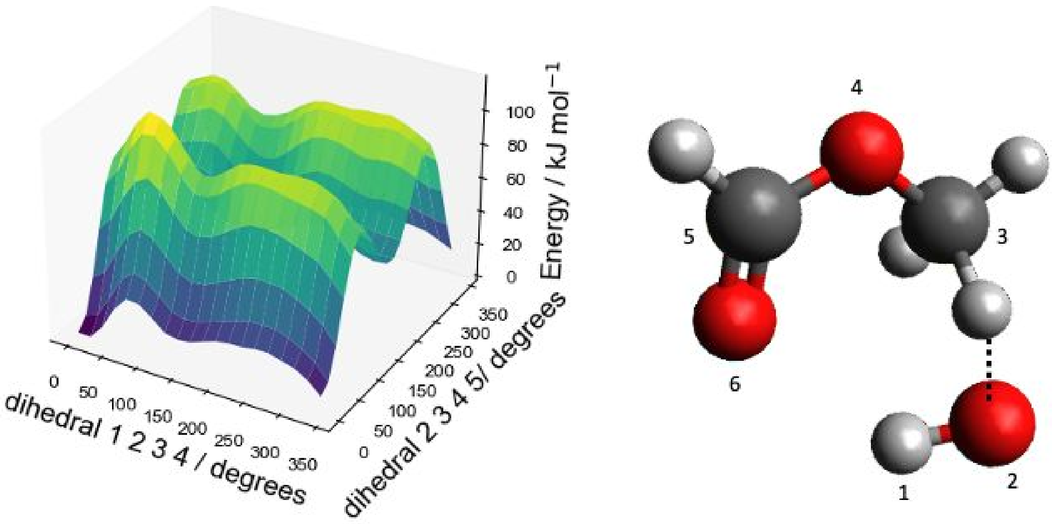

Figure S4. Torsional potential for two coupled rotors in TS_{methyl}. The atom numbering identifies the torsional angles corresponding to each rotor. The rotor corresponding to the dihedral angle formed by atoms 3 4 5 6 is held at its value in the minimised saddle point.

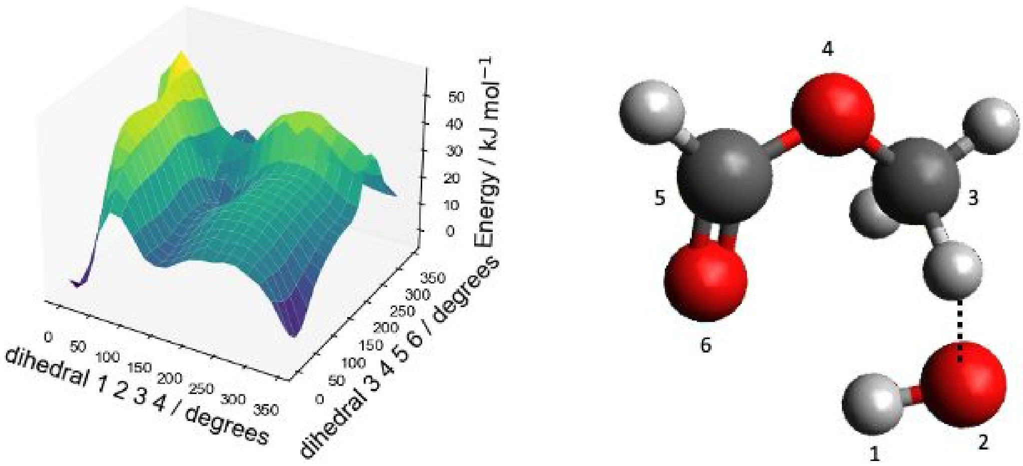

Figure S5. Torsional potential for two coupled rotors in TS_{methyl}. The atom numbering identifies the torsional angles corresponding to each rotor. The rotor corresponding to the dihedral angle formed by atoms 2 3 4 5 is held at its value in the minimised saddle point.

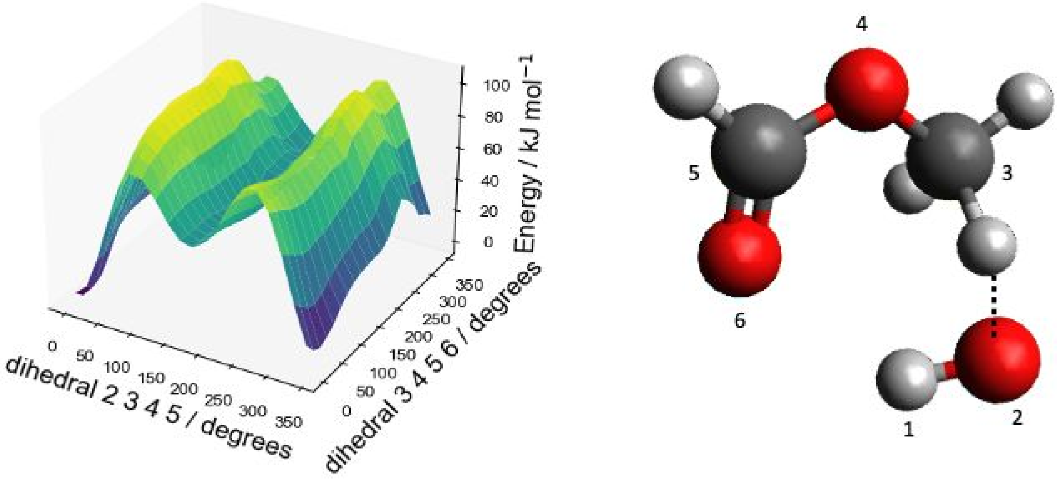

Figure S6. Torsional potential for two coupled rotors in TS_{methyl}. The atom numbering identifies the torsional angles corresponding to each rotor. The rotor corresponding to the dihedral angle formed by atoms 1 2 3 4 is held at its value in the minimised saddle point.

4. XYZ coordinates

Methylformate

8

C 0.877472 0.038966 -0.042438
O 0.891320 1.443191 -0.347097
C 0.945703 2.236724 0.728602
O 0.981478 1.863736 1.870791
H 0.009502 -0.204764 0.567169
H 1.781599 -0.237446 0.496623
H 0.830043 -0.466856 -1.001129
H 0.952534 3.284372 0.410751

TSmethyl
10
C 0.5977 -0.6858 0.5630
O -0.7808 -0.8839 0.3276
H 1.0490 -1.6591 0.7210
H 0.7810 0.0286 1.3621
H 1.1108 -0.2155 -0.4268
C -1.4837 0.2355 0.0932
H -2.5375 -0.0166 -0.0516
O -1.0248 1.3461 0.0457
O 1.4453 0.5617 -1.4320

H 0.8431 1.2888 -1.2023

TSald
10

C 1.9103 -0.6740 -0.0190
O 0.4676 -0.7227 0.0272
H 2.2453 0.3611 -0.0246
H 2.2450 -1.1858 0.8768
H 2.2688 -1.1794 -0.9133
C -0.1300 -0.1330 -1.0060
H -1.3033 -0.2328 -0.8643
O 0.3907 0.4118 -1.9327
O -2.5420 -0.5515 -0.3465
H -2.2343 -1.0110 0.4507

5. ANLO-like energy contributions

	OH	MF	Combined Reactants	TS_{meth}	TS_{ald}
$E_{CCSD(T)-F12/cc-pVQZ-F12}$	-75.673921	-228.83375	-304.50767	-304.50392	-304.50275
$E_{CCSD(T,full)/aug-cc-pCVTZ}$	-75.702611	-228.9614	-304.66401	-304.66026	-304.65918
$E_{CCSD(T)/aug-cc-pCVTZ}$	-75.586826	-228.55408	-304.1409	-304.13731	-304.13638
ΔE_{so}	-0.1157853	-0.4073248	-0.5231101	-0.5229484	-0.5227977
$E_{CCSDT(Q)/aug-cc-pVDZ}$	-75.559719	-228.49339	-304.05311	-304.04745	-304.04646
$E_{CCSD(T)/aug-cc-pVDZ}$	-75.559277	-228.49082	-304.0501	-304.04386	-304.0427
ΔE_{quad}	-0.0004416	-0.002563	-0.0030045	-0.003589	-0.0037588
$E_{CCSD(T,DK)/cc-pVTZ-DK}$	-75.689735	-228.85953	-304.54926	-304.54494	-304.54371
$E_{CCSD(T)/cc-pVTZ-DK}$	-75.633067	-228.71574	-304.34881	-304.3446	-304.34333
ΔE_{DK}	0.05666738	0.14379011	0.20045749	0.20034099	0.20038531
$ZPE^{anharmonic}_{B3LYP/6-311+G^{**}}$	0.008613	6.31E-02	0.07176	6.99E-02	6.97E-02
$ZPE^{harmonic}_{B3LYP/6-311+G^{**}}$	0.176	2.198	2.374	4.719	2.361
ΔE_{anharm}	0.00854597	0.06230983	0.07085579	0.06813963	0.06875174
$E_{ANL-like}$	-75.724935	-229.03754	-304.76247	-304.76198	-304.76017

Table S9 – Contributions to the ANL-like energies for the two abstraction transition states. All energies are in Hartee

6. Python input for simultaneous MESMER fits

```
from MESMER_API.src.Main import MESMER_API
import numpy as np
from scipy.optimize import least_squares as lm
from subprocess import Popen, PIPE
```

```
def get_chi( values, inputs, params):
    mol_dict = dict(zip(params,values))
    chi =[]
    for inp in inputs:
        inp.modify_in_place(mol_dict)
        #run mesmer
        p = Popen(['/home/home02/chmrsh/Mes7/mesmer-code/bin/mesmer','temp.xml'],
stdout=PIPE, stderr=PIPE )
        stdout, stderr = p.communicate()
        out = stderr.decode("utf-8")
        out = MESMER_API()
        out.parse_me_xml('mesmer_out.xml')
        chi += out.get_chi_sq()
    return chi

def sumarise( values, inputs, params):
    mol_dict = dict(zip(params,values))
    results =[]
    for i,inp in enumerate(inputs):
        inp.modify_in_place(mol_dict)
        #run mesmer
        p = Popen(['/home/home02/chmrsh/Mes7/mesmer-code/bin/mesmer','temp.xml'],
stdout=PIPE, stderr=PIPE )
        stdout, stderr = p.communicate()
        out = stderr.decode("utf-8")
        out = MESMER_API()
        out.parse_me_xml('mesmer_out.xml')
        eigs = out.get_eigen_vs_expt()
        for i,sp in enumerate(out.species_profile_list):
            list1 = sp.get_species('CH_post_comp')
            list2 = sp.get_species('CH3_post_comp')
            branch1 = list1[-1]
            branch2 = list2[-1]
```

```
            results.append([eigs[0][i],eigs[1][i],eigs[2][i],eigs[3][i],branch1,branch2])
    return results

me = MESMER_API()
me.parse_me_xml('NonDeut.xml')
me2 = MESMER_API()
me2.parse_me_xml('1D.xml')
me3 = MESMER_API()
me3.parse_me_xml('3D.xml')
out = MESMER_API()
me4 = MESMER_API()
me4.parse_me_xml('4D.xml')
me5 = MESMER_API()
me5.parse_me_xml('ODNon.xml')
me6 = MESMER_API()
me6.parse_me_xml('OD1D.xml')
me7 = MESMER_API()
me7.parse_me_xml('OD3D.xml')
me8 = MESMER_API()
me8.parse_me_xml('OD4D.xml')
me9 = MESMER_API()
me9.parse_me_xml('NonDeutKIT.xml')
me10 = MESMER_API()
me10.parse_me_xml('1DKIT.xml')
me11 = MESMER_API()
me11.parse_me_xml('3DKIT.xml')


inputs = [me,me2,me3,me4,me5,me6,me7,me8,me9,me10,me11]
params = ['TS_COC=O_CO[C]=O', 'TS_COC=O_[CH2]OC=O']
values = [-1.98,-1.66]

sol=lm(get_chi, values, method='lm', diff_step=0.05, ftol=1e-06, args=(inputs,params))
print('variables = ' + str(sol.x))
```

```
print('chi values = ' + str(sol.fun))


for i,inp in enumerate(inputs):
    res = sumarise(sol.x,[inp],params)
    with open(str(i)+'fittingAllWithKIT_2', 'w') as fp:
        fp.write('Temp\texp\terr\tcal\tAld\tMeth\n')
        for item in res:
            fp.write(str(item[0])+'\t'+str(item[1])+'\t'+ str(item[2])+'\t'+ str(item[3])+'\t'+ str(item[4])+'\t'+ str(item[5])+"\n")
```

7. Example MESMER input xml file


```
<?xml version="1.0" encoding="utf-8"?>
<?xml-stylesheet type='text/xsl' href='../../mesmer1.xsl'?>
<me:mesmer xmlns="http://www.xml-cml.org/schema"
xmlns:me="http://www.chem.leeds.ac.uk/mesmer"
xmlns:xsi="http://www.w3.org/2001/XMLSchema-instance">
<title> Glyoxal</title>
<moleculeList>
  <molecule id="N2">
    <atomArray>
      <atom id="a1" elementType="N" />
      <atom id="a2" elementType="N" />
    </atomArray>
    <bondArray>
      <bond atomRefs2="a2 a1" order="3" />
    </bondArray>
    <propertyList>
      <property dictRef="me:epsilon">
        <scalar>82</scalar>
      </property>
      <property dictRef="me:sigma">
        <scalar>3.74</scalar>
      </property>
      <property dictRef="me:MW">
        <scalar units="amu">28.0</scalar>
      </property>
    </propertyList>
  </molecule>
  <molecule spinMultiplicity="2" id="CH_pre_comp">
    <atomArray>
      <atom id="a1" elementType="C" x3="1.398423" y3="-0.305443" z3="0.603972" />
      <atom id="a2" elementType="O" x3="0.659724" y3="-0.576933" z3="1.801438" />
```

```
      <atom id="a3" elementType="H" x3="1.410977" y3="0.776264" z3="0.445273" />
      <atom id="a4" elementType="H" x3="2.453772" y3="-0.572018" z3="0.746166" />
      <atom id="a5" elementType="H" x3="1.048452" y3="-0.916206" z3="-0.232457" />
      <atom id="a6" elementType="C" x3="-0.702434" y3="-0.484829" z3="1.772164" />
      <atom id="a7" elementType="H" x3="-1.038622" y3="-0.583752" z3="2.815566" />
      <atom id="a8" elementType="O" x3="-1.499101" y3="-0.361628" z3="0.873733" />
      <atom id="a9" elementType="O" spinMultiplicity="2" x3="-0.142439" y3="0.973403"
z3="-1.605430" />
      <atom id="a10" elementType="H" x3="0.750486" y3="0.754002" z3="-1.416029" />
    </atomArray>
    <bondArray>
      <bond atomRefs2="a3 a1" order="1" />
      <bond atomRefs2="a9 a10" order="1" />
      <bond atomRefs2="a8 a6" order="2" />
      <bond atomRefs2="a1 a5" order="1" />
      <bond atomRefs2="a1 a2" order="1" id="bond12" />
      <bond atomRefs2="a1 a4" order="1" />
      <bond atomRefs2="a6 a2" order="1" />
      <bond atomRefs2="a6 a7" order="1" />
      <bond atomRefs2="a9 a3" order="1" />
    </bondArray>
    <propertyList>
      <property dictRef="me:ZPE">
        <scalar units="kJ/mol">-10.25856098</scalar>
      </property>
      <property dictRef="me:vibFreqs">
        <array units="cm-1">65.738 103.43 183.8473 252.6934 297.2885 332.8636 359.1348
456.8625 791.2725 980.0732 1062.468 1183.3367 1211.4586 1310.4063 1409.3403
1485.5761 1506.7865 1523.9127 1836.2717 3093.4648 3162.1209 3168.1798 3215.9393
3683.2901</array>
      </property>
      <property dictRef="me:spinMultiplicity">
        <scalar units="cm-1">2</scalar>
      </property>
      <property dictRef="me:sigma" default="true">
        <scalar>5.0</scalar>
      </property>
      <property dictRef="me:epsilon" default="true">
        <scalar>50.0</scalar>
      </property>
      <property dictRef="me:frequenciesScaleFactor" default="true">
        <scalar>0.9522</scalar>
      </property>

      <property dictRef="me:symmetryNumber" default="true">
        <scalar>1</scalar>
      </property>
    </propertyList>
    <me:DOSCMethod default="true" name="ClassicalRotors" />
    <me:DistributionCalcMethod default="true" name="Boltzmann" />
```

<me:energyTransferModel name="ExponentialDown" default="true" />
<me:deltaEDown default="NEEDS TO BE CHECKED**">130.0</me:deltaEDown>
</molecule>
<molecule spinMultiplicity="2" id="[O]OCOC=O">
<atomArray>
<atom id="a1" elementType="C" x3="0.929497" y3="-0.023818" z3="0.171512"/>
<atom id="a2" elementType="O" x3="0.845715" y3="1.210230" z3="-0.494566"/>
<atom id="a3" elementType="H" x3="0.524808" y3="-0.776148" z3="-
0.502192"/>
<atom id="a4" elementType="H" x3="0.446795" y3="0.014410" z3="1.148302"/>
<atom id="a5" elementType="C" x3="0.926396" y3="2.313669" z3="0.287988"/>
<atom id="a6" elementType="O" x3="1.035345" y3="2.301946" z3="1.479590"/>
<atom id="a7" elementType="H" x3="0.867081" y3="3.208990" z3="-0.341885"/>
<atom id="a8" elementType="O" x3="2.298044" y3="-0.339340" z3="0.442677"/>
<atom id="a9" elementType="O" x3="2.903138" y3="-0.749039" z3="-
0.642546"/>
</atomArray>
<bondArray>
<bond atomRefs2="a3 a1" order="1" />
<bond atomRefs2="a2 a1" order="1" id="bond21" />
<bond atomRefs2="a2 a5" order="1" id="bond25" />
<bond atomRefs2="a1 a8" order="1" id="bond18" />
<bond atomRefs2="a1 a4" order="1" />
<bond atomRefs2="a8 a9" order="1" />
<bond atomRefs2="a7 a5" order="1" />
<bond atomRefs2="a5 a6" order="2" />
</bondArray>
<propertyList>
<property dictRef="me:ZPE">
<scalar units="kJ/mol">-210.34</scalar>
</property>
<property dictRef="me:vibFreqs">
<array units="cm-1">53.9147 114.9468 246.3547 331.6236 396.6581 628.638 773.2053
971.7418 1025.0109 1056.2848 1162.8852 1197.0561 1271.7399 1322.1332 1409.2136
1431.1997 1495.2944 1889.6374 3148.9178 3170.035 3232.7224</array>
</property>
<property dictRef="me:spinMultiplicity">
<scalar units="cm-1">2</scalar>
</property>
<property dictRef="me:hessian">
<matrix matrixType="squareSymmetricLT" rows="27" units="Hartree/Bohr2">
0.64759945 -0.02993682 0.46806405 0.00817150 0.00836654 0.60997378 -0.08210618 -
0.03320940 -0.00034029 0.53527191 -0.00048651 -0.22065562 0.01273595 0.12229135
0.45770109 -0.00283212 0.00768630 -0.06522183 0.05772739 -0.01255266 0.08798345 -
0.33778569 -0.01429660 -0.03136668 -0.00557707 -0.00340511 -0.00057125 0.35811974
0.00119599 -0.04601049 -0.00040637 -0.04413610 -0.01554671 -0.00412584 0.01840465
0.06218405 -0.03246299 -0.00225080 -0.05761452 0.00280469 0.00083482 0.00045699
0.03411962 0.00115601 0.05153612 -0.07782705 0.03023986 -0.07806766 0.00026304 -
0.00642164 0.00914281 -0.00516936 0.01062157 -0.02830543 0.07431240 0.02519928 -
0.07784686 0.07564715 0.00040820 -0.03102967 0.03136361 0.00048012 0.00247254 -

0.00264585 -0.02756513 0.09757797 -0.07381901 0.08463715 -0.27708497 0.00305864 0.00287044 0.00560248 0.00073422 -0.00106286 0.00009244 0.08081017 -0.09306483 0.28961561 -0.12024166 0.06514045 0.10383042 0.00365133 -0.00769665 -0.00940368 -0.01087328 0.01404749 0.02406167 0.00535111 -0.00484067 -0.00851517 0.11935141 0.05791453 -0.10480414 -0.09023247 0.00622517 -0.03543555 -0.02489273 -0.00042495 0.00311438 0.00257806 -0.00542564 0.00809461 0.00772932 -0.06488837 0.12863562 0.10070913 -0.09748617 -0.21045424 -0.00470254 0.00145609 0.00700741 -0.00315343 0.00375809 0.00479558 0.01490722 -0.01298080 -0.01855406 -0.10949016 0.10473203 0.21663397 -0.02987756 -0.04178543 -0.00013155 -0.33379647 -0.05760047 -0.03446011 0.00145743 0.00283415 -0.00031019 0.00410200 0.00915268 -0.00340357 0.00379875 0.01003015 0.00248139 1.03063081 -0.04877805 -0.02158523 -0.00552117 -0.03444927 -0.12545527 -0.00079988 0.00030210 -0.00752766 0.00066827 -0.00283946 0.00014098 0.00087279 -0.00290276 -0.00045444 -0.00137764 -0.24101450 0.71820562 -0.00000064 -0.00441921 -0.00036794 -0.03639873 -0.00399863 -0.07468164 0.00026826 0.00126317 -0.00011398 0.00274711 0.00341820 0.00053164 -0.00142905 -0.00048516 0.00121899 0.14314934 -0.07697414 0.23251761 0.00430271 0.01846654 -0.00029286 -0.09524521 0.02363183 -0.02048183 0.00009746 -0.00064021 -0.00004665 -0.00072435 -0.00215449 0.00071020 -0.00061301 -0.00242899 -0.00049301 -0.55699378 0.34447441 -0.09736139 0.64561804 -0.00527792 -0.00499302 -0.00029640 0.02142218 0.01977086 0.00411017 -0.00069667 0.00223020 -0.00037011 0.00136688 0.00152741 -0.00165601 0.00115498 0.00171149 0.00153791 0.33956215 -0.36911376 0.07286035 -0.40545977 0.42134481 0.00167648 0.00289344 -0.00400294 -0.02038871 0.00445536 0.02771585 -0.00000421 -0.00024786 0.00066026 -0.00055852 -0.00088431 0.00012422 0.00028071 -0.00017399 -0.00018485 -0.09736401 0.07406479 -0.10351901 0.12266382 -0.09099958 0.06089605 -0.00328778 0.00441825 -0.00145836 -0.02528947 -0.06820134 0.00063201 -0.00030399 -0.00196551 0.00011256 -0.00035230 -0.00070191 0.00033781 -0.00047495 -0.00096946 -0.00018959 -0.09544096 0.03557409 -0.01146361 0.00111328 0.03488051 -0.00581119 0.51557592 0.00203894 0.00643820 -0.00007322 -0.03585540 -0.03906067 -0.00140045 -0.00022368 -0.00015752 -0.00001541 0.00005523 -0.00085995 -0.00033891 -0.00001412 -0.00082003 0.00037894 0.03609227 -0.15625868 0.01597260 0.03350236 -0.07854051 0.01289918 0.08331607 0.35970650 -0.00128438 0.00036806 0.00353131 -0.00229170 -0.00661481 0.00965172 -0.00003789 -0.00032852 0.00015134 -0.00061507 -0.00064463 -0.00034092 0.00057824 0.00051507 -0.00043729 -0.01111262 0.01545540 -0.05349799 -0.00573427 0.01310228 0.01860415 0.08193594 -0.01343657 0.03535869 -0.00077623 0.00096314 -0.00034452 0.00282813 -0.00211146 0.00024676 0.00003475 -0.00036202 0.00002673 0.00004451 0.00002192 0.00008672 0.00005030 -0.00003244 -0.00006902 -0.02388022 -0.05036656 0.00048869 0.00244487 0.01304765 -0.00049438 -0.39153975 -0.11891167 -0.06143826 0.41079364 -0.00186945 0.00139310 -0.00022001 -0.00269673 -0.01028845 0.00061147 -0.00013985 -0.00075878 0.00004502 -0.00003168 -0.00007702 0.00001293 -0.00000035 -0.00004196 -0.00001845 -0.05727100 -0.03795155 -0.00763718 -0.00939169 0.00606250 -0.00200703 -0.08635068 -0.09044734 -0.00841629 0.15775143 0.13210950 -0.00015797 0.00020469 0.00124135 0.00053126 0.00081344 0.00148558 0.00001135 -0.00000581 0.00003578 -0.00006065 -0.00020854 0.00001355 0.00008701 0.00022987 -0.00002551 0.00115132 -0.00638843 -0.00208768 0.00103599 0.00171140 -0.00029372 -0.06409558 -0.01398616 -0.01302101 0.06149728 0.01762954 0.01265165</matrix>
</property>
<property dictRef="me:sigma" default="true">
<scalar>5.0</scalar>
</property>

<property dictRef="me:epsilon" default="true">
<scalar>50.0</scalar>
</property>
<property dictRef="me:vibFreqsFromHessian">
<scalar calculated="20220120_074051" units="cm-1">53.918 114.946 246.368 331.633 396.658 628.639 773.201 971.738 1025 1056.28 1162.88 1197.04 1271.73 1322.13 1409.21 1431.2 1495.29 1889.63 3148.91 3170.02 3232.71 </scalar>
</property>
<property dictRef="me:frequenciesScaleFactor" default="true">
<scalar>0.9522</scalar>
</property>
<property dictRef="me:symmetryNumber" default="true">
<scalar>1</scalar>
</property>
</propertyList>
<me:DOSCMethod xsi:type="me:ClassicalCoupledRotors">
<me:MCPoints>10000</me:MCPoints>
<me:RotorArray>
<me:Rotor>
<bondRef>bond21</bondRef>
<me:HinderedRotorPotential format="numerical" units="kJ/mol" expansionSize="10" UseSineTerms="yes">
<me:PotentialPoint angle=" 0 " potential=" 0 "/>
<me:PotentialPoint angle=" 10 " potential=" 0.918925 "/>
<me:PotentialPoint angle=" 20 " potential=" 3.93825 "/>
<me:PotentialPoint angle=" 30 " potential=" 9.057975 "/>
<me:PotentialPoint angle=" 40 " potential=" 16.041805 "/>
<me:PotentialPoint angle=" 50 " potential=" 22.710575 "/>
<me:PotentialPoint angle=" 60 " potential=" 28.67046 "/>
<me:PotentialPoint angle=" 70 " potential=" 33.370105 "/>
<me:PotentialPoint angle=" 80 " potential=" 36.205645 "/>
<me:PotentialPoint angle=" 90 " potential=" 36.730745 "/>
<me:PotentialPoint angle=" 100 " potential=" 34.735365 "/>
<me:PotentialPoint angle=" 110 " potential=" 29.956955 "/>
<me:PotentialPoint angle=" 120 " potential=" 23.41946 "/>
<me:PotentialPoint angle=" 130 " potential=" 15.989295 "/>
<me:PotentialPoint angle=" 140 " potential=" 9.793115 "/>
<me:PotentialPoint angle=" 150 " potential=" 6.14367 "/>
<me:PotentialPoint angle=" 160 " potential=" 3.491915 "/>
<me:PotentialPoint angle=" 170 " potential=" 3.806975 "/>
<me:PotentialPoint angle=" 180 " potential=" 5.14598 "/>
<me:PotentialPoint angle=" 190 " potential=" 6.72128 "/>
<me:PotentialPoint angle=" 200 " potential=" 8.29658 "/>
<me:PotentialPoint angle=" 210 " potential=" 9.583075 "/>
<me:PotentialPoint angle=" 220 " potential=" 10.475745 "/>
<me:PotentialPoint angle=" 230 " potential=" 10.843315 "/>
<me:PotentialPoint angle=" 240 " potential=" 10.81706 "/>
<me:PotentialPoint angle=" 250 " potential=" 8.29658 "/>
<me:PotentialPoint angle=" 260 " potential=" 9.583075 "/>
<me:PotentialPoint angle=" 270 " potential=" 10.475745 "/>

<me:PotentialPoint angle=" 280 " potential=" 10.843315 "/>
<me:PotentialPoint angle=" 290 " potential=" 10.81706 "/>
<me:PotentialPoint angle=" 300 " potential=" 10.60702 "/>
<me:PotentialPoint angle=" 310 " potential=" 9.845625 "/>
<me:PotentialPoint angle=" 320 " potential=" 8.61164 "/>
<me:PotentialPoint angle=" 330 " potential=" 7.062595 "/>
<me:PotentialPoint angle=" 340 " potential=" 5.40853 "/>
<me:PotentialPoint angle=" 350 " potential=" 3.964505 "/>
<me:PotentialPoint angle=" 360 " potential=" 0 "/>
</me:HinderedRotorPotential>

</me:Rotor>
<me:Rotor>
<bondRef>bond25</bondRef>
<me:HinderedRotorPotential format="numerical" units="kJ/mol" expansionSize="10" UseSineTerms="yes">
<me:PotentialPoint angle=" 0 " potential=" 0 "/>
<me:PotentialPoint angle=" 10 " potential=" 1.36526 "/>
<me:PotentialPoint angle=" 20 " potential=" 5.40853 "/>
<me:PotentialPoint angle=" 30 " potential=" 12.02479 "/>
<me:PotentialPoint angle=" 40 " potential=" 20.557665 "/>
<me:PotentialPoint angle=" 50 " potential=" 30.272015 "/>
<me:PotentialPoint angle=" 60 " potential=" 39.85509 "/>
<me:PotentialPoint angle=" 70 " potential=" 47.7841 "/>
<me:PotentialPoint angle=" 80 " potential=" 52.483745 "/>
<me:PotentialPoint angle=" 90 " potential=" 54.059045 "/>
<me:PotentialPoint angle=" 100 " potential=" 52.87757 "/>
<me:PotentialPoint angle=" 110 " potential=" 49.228125 "/>
<me:PotentialPoint angle=" 120 " potential=" 43.977125 "/>
<me:PotentialPoint angle=" 130 " potential=" 38.06975 "/>
<me:PotentialPoint angle=" 140 " potential=" 32.29365 "/>
<me:PotentialPoint angle=" 150 " potential=" 27.25269 "/>
<me:PotentialPoint angle=" 160 " potential=" 23.445715 "/>
<me:PotentialPoint angle=" 170 " potential=" 21.10902 "/>
<me:PotentialPoint angle=" 180 " potential=" 20.4789 "/>
<me:PotentialPoint angle=" 190 " potential=" 21.58161 "/>
<me:PotentialPoint angle=" 200 " potential=" 24.31213 "/>
<me:PotentialPoint angle=" 210 " potential=" 28.434165 "/>
<me:PotentialPoint angle=" 220 " potential=" 33.34385 "/>
<me:PotentialPoint angle=" 230 " potential=" 38.463575 "/>
<me:PotentialPoint angle=" 240 " potential=" 43.0582 "/>
<me:PotentialPoint angle=" 250 " potential=" 46.392585 "/>
<me:PotentialPoint angle=" 260 " potential=" 47.915375 "/>
<me:PotentialPoint angle=" 270 " potential=" 47.285255 "/>
<me:PotentialPoint angle=" 280 " potential=" 44.31844 "/>
<me:PotentialPoint angle=" 290 " potential=" 39.27748 "/>
<me:PotentialPoint angle=" 300 " potential=" 32.60871 "/>
<me:PotentialPoint angle=" 310 " potential=" 25.04727 "/>
<me:PotentialPoint angle=" 320 " potential=" 17.43332 "/>
<me:PotentialPoint angle=" 330 " potential=" 10.528255 "/>

```
<me:PotentialPoint angle=" 340 " potential=" 5.014705 "/>
<me:PotentialPoint angle=" 350 " potential=" 1.36526 "/>
<me:PotentialPoint angle=" 360 " potential=" 0 "/>
</me:HinderedRotorPotential>

</me:Rotor>
<me:Rotor>
<bondRef>bond18</bondRef>
<me:periodicity>3</me:periodicity>
<me:HinderedRotorPotential format="numerical" units="kJ/mol" expansionSize="10"
UseSineTerms="yes">
<me:PotentialPoint angle=" 0 " potential=" 0 "/>
<me:PotentialPoint angle=" 10 " potential=" 0.57761 "/>
<me:PotentialPoint angle=" 20 " potential=" 2.31044 "/>
<me:PotentialPoint angle=" 30 " potential=" 4.752155 "/>
<me:PotentialPoint angle=" 40 " potential=" 7.56144 "/>
<me:PotentialPoint angle=" 50 " potential=" 9.50431 "/>
<me:PotentialPoint angle=" 60 " potential=" 10.108175 "/>
<me:PotentialPoint angle=" 70 " potential=" 10.02941 "/>
<me:PotentialPoint angle=" 80 " potential=" 9.4518 "/>
<me:PotentialPoint angle=" 90 " potential=" 8.060285 "/>
<me:PotentialPoint angle=" 100 " potential=" 6.695025 "/>
<me:PotentialPoint angle=" 110 " potential=" 4.7259 "/>
<me:PotentialPoint angle=" 120 " potential=" 3.1506 "/>
<me:PotentialPoint angle=" 130 " potential=" 1.916615 "/>
<me:PotentialPoint angle=" 140 " potential=" 1.68032 "/>
<me:PotentialPoint angle=" 150 " potential=" 2.441715 "/>
<me:PotentialPoint angle=" 160 " potential=" 3.649445 "/>
<me:PotentialPoint angle=" 170 " potential=" 5.329765 "/>
<me:PotentialPoint angle=" 180 " potential=" 6.169925 "/>
<me:PotentialPoint angle=" 190 " potential=" 5.644825 "/>
<me:PotentialPoint angle=" 200 " potential=" 5.30351 "/>
<me:PotentialPoint angle=" 210 " potential=" 5.172235 "/>
<me:PotentialPoint angle=" 220 " potential=" 4.77841 "/>
<me:PotentialPoint angle=" 230 " potential=" 4.41084 "/>
<me:PotentialPoint angle=" 240 " potential=" 3.859485 "/>
<me:PotentialPoint angle=" 250 " potential=" 3.46566 "/>
<me:PotentialPoint angle=" 260 " potential=" 3.51817 "/>
<me:PotentialPoint angle=" 270 " potential=" 3.859485 "/>
<me:PotentialPoint angle=" 280 " potential=" 4.384585 "/>
<me:PotentialPoint angle=" 290 " potential=" 4.83092 "/>
<me:PotentialPoint angle=" 300 " potential=" 5.04096 "/>
<me:PotentialPoint angle=" 310 " potential=" 4.77841 "/>
<me:PotentialPoint angle=" 320 " potential=" 3.964505 "/>
<me:PotentialPoint angle=" 330 " potential=" 2.99307 "/>
<me:PotentialPoint angle=" 340 " potential=" 1.601555 "/>
<me:PotentialPoint angle=" 350 " potential=" 0.603865 "/>
<me:PotentialPoint angle=" 360 " potential=" 0 "/>
</me:HinderedRotorPotential>
```

</me:Rotor>

</me:RotorArray>
</me:DOSCMethod>

<me:energyTransferModel xsi:type="me:ExponentialDown">
<me:deltaEDown >57</me:deltaEDown>
<me:deltaEDownTExponent referenceTemperature="298"
>0.4</me:deltaEDownTExponent>
</me:energyTransferModel>
</molecule>

<molecule spinMultiplicity="2" id="[CH2]OC=O">
<atomArray>
<atom id="a1" elementType="C" spinMultiplicity="2" x3="1.142410" y3="-0.043633"
z3="0.097038" />
<atom id="a2" elementType="H" x3="2.210766" y3="-0.191102" z3="0.032099" />
<atom id="a3" elementType="H" x3="0.656323" y3="0.841156" z3="-0.297560" />
<atom id="a4" elementType="O" x3="0.574664" y3="-0.365984" z3="1.329850" />
<atom id="a5" elementType="C" x3="0.928000" y3="-1.561004" z3="1.877678" />
<atom id="a6" elementType="O" x3="0.559565" y3="-1.931300" z3="2.986197" />
<atom id="a7" elementType="H" x3="1.571180" y3="-2.170765" z3="1.225483" />
</atomArray>
<bondArray>
<bond atomRefs2="a7 a5" order="1" />
<bond atomRefs2="a5 a4" order="1" id="bond54" />
<bond atomRefs2="a5 a6" order="2" />
<bond atomRefs2="a4 a1" order="1" id="bond41" />
<bond atomRefs2="a1 a3" order="1" />
<bond atomRefs2="a1 a2" order="1" />
</bondArray>
<propertyList>
<property dictRef="me:ZPE">
<scalar units="kJ/mol">-79.07411606</scalar>
</property>
<property dictRef="me:vibFreqs">
<array units="cm-1">294.7414 350.6503 361.1965 385.928 770.2518 1028.5275
1048.6918 1186.3673 1284.5562 1410.7167 1462.0424 1861.0154 3150.4263 3226.9493
3381.4734</array>
</property>
<property dictRef="me:spinMultiplicity">
<scalar units="cm-1">2</scalar>
</property>
<property dictRef="me:hessian">
<matrix matrixType="squareSymmetricLT" rows="21"
units="Hartree/Bohr2">0.71454381 -0.07409462 0.49436686 0.03207826 0.21793558
0.13230981 -0.17394200 0.15541841 0.03843933 0.16906753 0.14623788 -0.23197404 -
0.07356571 -0.15364786 0.24662311 0.02622902 -0.06068205 -0.02559254 -0.03565895
0.07658002 0.03082940 -0.37296041 0.00666945 -0.00259982 -0.00404615 -0.00447740
0.00049831 0.38462261 0.01464757 -0.04385608 -0.01963609 0.01688116 0.00653943

0.00222390 0.00166689 0.04535828 0.01614992 -0.01965662 -0.01197827 0.00384470
0.00350118 0.00031631 0.00339425 0.02194968 0.01638454 -0.13212268 -0.06331201 -
0.05177326 0.01068498 0.00587473 0.00676655 -0.00944216 -0.03621719 -0.02334646
0.50774255 -0.03817917 -0.21177386 -0.11209356 -0.01639881 -0.02192800 -0.01913470 -
0.00284621 -0.00527304 -0.00257831 0.05959208 0.32982736 -0.04100871 -0.12784901 -
0.08972310 -0.00539061 -0.00776658 -0.00521333 -0.00122919 -0.00221558 -0.00546810
0.09224124 0.17152274 0.15132986 -0.03769031 -0.03710958 -0.02331089 0.00310260
0.00705237 0.00266681 0.00217283 0.00478251 0.00093525 -0.25765762 -0.01720641 -
0.03396166 0.71819242 -0.04767350 -0.00706783 -0.01623432 -0.00315104 -0.00256665
0.00034646 -0.00074506 -0.00659083 -0.00379695 -0.01218125 -0.10056923 -0.02327514 -
0.19526674 0.82846946 -0.03361937 -0.01187602 -0.00830583 -0.00110448 -0.00079630
0.00033331 0.00028791 -0.00371698 0.00014751 -0.03138245 -0.02405495 -0.08013377 -
0.04455543 0.34529065 0.38047022 0.00620754 0.00912777 0.00561786 -0.00464749 -
0.00079398 -0.00030387 0.00002353 -0.00044671 0.00004358 -0.09459135 0.04745855
0.01271993 -0.36029389 0.31398262 0.14314531 0.44469126 -0.00161497 -0.00305542
0.00369266 0.00044145 0.00316592 0.00056387 -0.00035065 0.00368849 0.00078550
0.04971750 0.00933433 -0.00419349 0.30107634 -0.480749423 -0.19757490 -0.37656643
0.48813205 0.00056578 0.00178850 -0.00090875 -0.00011754 0.00206813 -0.00056157 -
0.00022726 0.00198797 0.00054078 0.01396349 -0.00394833 0.02540980 0.13572325 -
0.19624568 -0.16608411 -0.16406341 0.21700262 0.13228154 -0.00403594 0.00330057
0.00154853 -0.00021946 -0.00024573 -0.00019788 -0.00037025 -0.00131423 -0.00102124 -
0.02461371 -0.03242002 -0.02337098 -0.06782603 -0.05496504 -0.03277149 0.00861041
0.02729676 0.01415568 0.08845497 0.00067681 0.00336036 -0.00009856 0.00045668
0.00014023 0.00010251 0.00008298 0.00013374 -0.00020448 -0.00347387 0.00038244 -
0.00622293 -0.06332848 -0.23088069 -0.10727150 0.00723819 -0.02047115 -0.02265321
0.05834769 0.24733506 -0.00039490 0.00033962 0.00419868 -0.00001245 -0.00002073 -
0.00011159 -0.00012420 -0.00059288 0.00005724 -0.00646911 -0.00971289 0.00379864 -
0.03749733 -0.10608503 -0.12642734 0.00284061 -0.02027626 0.00932232 0.04165739
0.13634817 0.10916205</matrix>
</property>
<property dictRef="me:sigma" default="true">
<scalar>5.0</scalar>
</property>
<property dictRef="me:epsilon" default="true">
<scalar>50.0</scalar>
</property>
<property dictRef="me:vibFreqsFromHessian">
<scalar calculated="20220120_074051" units="cm-1">20.8268 67.1193 296.499
360.825 380.568 723.701 951.027 1028.52 1225.33 1362.31 1454.86 1802.08 3013.76
3224.07 3310.79 </scalar>
</property>
<property dictRef="me:frequenciesScaleFactor" default="true">
<scalar>0.9522</scalar>
</property>
<property dictRef="me:symmetryNumber" default="true">
<scalar>1</scalar>
</property>
</propertyList>
<me:DOSCMethod xsi:type="me:ClassicalCoupledRotors">
<me:MCPoints>10000</me:MCPoints>

<me:RotorArray>
<me:Rotor>
<bondRef>bond54</bondRef>
<me:HinderedRotorPotential format="numerical" units="kJ/mol" expansionSize="10" UseSineTerms="yes">
<me:PotentialPoint angle="0" potential="0.0" />
<me:PotentialPoint angle="5.0" potential="0.4411325357628544" />
<me:PotentialPoint angle="10.0" potential="1.6080559641126524" />
<me:PotentialPoint angle="15.0" potential="3.4817607491278193" />
<me:PotentialPoint angle="20.0" potential="6.00558879824363" />
<me:PotentialPoint angle="25.0" potential="9.167603418728888" />
<me:PotentialPoint angle="30.0" potential="12.897563330313004" />
<me:PotentialPoint angle="35.0" potential="17.086823892881576" />
<me:PotentialPoint angle="40.0" potential="21.67871721529148" />
<me:PotentialPoint angle="45.0" potential="26.54629329002374" />
<me:PotentialPoint angle="50.0" potential="31.54981551736587" />
<me:PotentialPoint angle="55.0" potential="36.542145727182685" />
<me:PotentialPoint angle="60.0" potential="41.36615687996483" />
<me:PotentialPoint angle="65.0" potential="45.864520932529736" />
<me:PotentialPoint angle="70.0" potential="49.89224202039036" />
<me:PotentialPoint angle="75.0" potential="53.31772633418212" />
<me:PotentialPoint angle="80.0" potential="56.06140359487522" />
<me:PotentialPoint angle="85.0" potential="58.03218536659442" />
<me:PotentialPoint angle="90.0" potential="59.25621054981347" />
<me:PotentialPoint angle="95.0" potential="59.66921115247533" />
<me:PotentialPoint angle="100.0" potential="59.427305868613495" />
<me:PotentialPoint angle="105.0" potential="58.56808075382123" />
<me:PotentialPoint angle="110.0" potential="57.19212410241966" />
<me:PotentialPoint angle="115.0" potential="55.3259643742121" />
<me:PotentialPoint angle="120.0" potential="53.19456610914807" />
<me:PotentialPoint angle="125.0" potential="50.856586953694126" />
<me:PotentialPoint angle="130.0" potential="48.40811355541083" />
<me:PotentialPoint angle="135.0" potential="45.92695382103154" />
<me:PotentialPoint angle="140.0" potential="43.511055374514264" />
<me:PotentialPoint angle="145.0" potential="41.21734756118457" />
<me:PotentialPoint angle="150.0" potential="39.11845526035748" />
<me:PotentialPoint angle="155.0" potential="37.255387803219165" />
<me:PotentialPoint angle="160.0" potential="35.68401412423888" />
<me:PotentialPoint angle="165.0" potential="34.427403212791745" />
<me:PotentialPoint angle="170.0" potential="33.528389329151686" />
<me:PotentialPoint angle="175.0" potential="32.991697019361695" />
<me:PotentialPoint angle="180.0" potential="32.84589486811625" />
<me:PotentialPoint angle="185.0" potential="33.0740204789669" />
<me:PotentialPoint angle="190.0" potential="33.69228581447692" />
<me:PotentialPoint angle="195.0" potential="34.66345570430082" />
<me:PotentialPoint angle="200.0" potential="35.98797383072145" />
<me:PotentialPoint angle="205.0" potential="37.61656763741865" />
<me:PotentialPoint angle="210.0" potential="39.529341618279716" />
<me:PotentialPoint angle="215.0" potential="41.66657055948712" />
<me:PotentialPoint angle="220.0" potential="43.9872340773951" />

<me:PotentialPoint angle="225.0" potential="46.42051965382508" />
<me:PotentialPoint angle="230.0" potential="48.90117769311079" />
<me:PotentialPoint angle="235.0" potential="51.34446233179084" />
<me:PotentialPoint angle="240.0" potential="53.659729885316935" />
<me:PotentialPoint angle="245.0" potential="55.747695068662566" />
<me:PotentialPoint angle="250.0" potential="57.47493910341877" />
<me:PotentialPoint angle="255.0" potential="58.77958567703779" />
<me:PotentialPoint angle="260.0" potential="59.550649602424585" />
<me:PotentialPoint angle="265.0" potential="59.635429633320115" />
<me:PotentialPoint angle="270.0" potential="59.04438672594271" />
<me:PotentialPoint angle="275.0" potential="57.70017750363254" />
<me:PotentialPoint angle="280.0" potential="55.542520414938465" />
<me:PotentialPoint angle="285.0" potential="52.66969128464698" />
<me:PotentialPoint angle="290.0" potential="49.11765654894104" />
<me:PotentialPoint angle="295.0" potential="44.99346011003207" />
<me:PotentialPoint angle="300.0" potential="40.393060744073374" />
<me:PotentialPoint angle="305.0" potential="35.5182570878027" />
<me:PotentialPoint angle="310.0" potential="30.508792381001744" />
<me:PotentialPoint angle="315.0" potential="25.521489104546998" />
<me:PotentialPoint angle="320.0" potential="20.70276154847079" />
<me:PotentialPoint angle="325.0" potential="16.18484193664487" />
<me:PotentialPoint angle="330.0" potential="12.079012515208778" />
<me:PotentialPoint angle="335.0" potential="8.472157700359608" />
<me:PotentialPoint angle="340.0" potential="5.450974403058788" />
<me:PotentialPoint angle="345.0" potential="3.0329721796430764" />
<me:PotentialPoint angle="350.0" potential="1.3134543477616207" />
<me:PotentialPoint angle="355.0" potential="0.3071953332927231" />
<me:PotentialPoint angle="360.0" potential="-0.0013055186483654387" />
</me:HinderedRotorPotential>

</me:Rotor>
<me:Rotor>
<bondRef>bond41</bondRef>
<me:periodicity>2</me:periodicity>
<me:HinderedRotorPotential format="numerical" units="kJ/mol" expansionSize="10" UseSineTerms="yes">
<me:PotentialPoint angle="0" potential="0.0" />
<me:PotentialPoint angle="5.0" potential="0.1292479424494682" />
<me:PotentialPoint angle="10.0" potential="0.5103478357006197" />
<me:PotentialPoint angle="15.0" potential="1.1210384418059054" />
<me:PotentialPoint angle="20.0" potential="1.983590897024204" />
<me:PotentialPoint angle="25.0" potential="3.0759037935247044" />
<me:PotentialPoint angle="30.0" potential="4.370547763771805" />
<me:PotentialPoint angle="35.0" potential="5.862966790398696" />
<me:PotentialPoint angle="40.0" potential="7.517762673544095" />
<me:PotentialPoint angle="45.0" potential="9.295482665675063" />
<me:PotentialPoint angle="50.0" potential="11.148483855753431" />
<me:PotentialPoint angle="55.0" potential="13.020259650271878" />
<me:PotentialPoint angle="60.0" potential="14.84573786524583" />
<me:PotentialPoint angle="65.0" potential="16.537936942644542" />

<me:PotentialPoint angle="70.0" potential="18.02249543554549" />
<me:PotentialPoint angle="75.0" potential="19.22036446303233" />
<me:PotentialPoint angle="80.0" potential="20.06781492344739" />
<me:PotentialPoint angle="85.0" potential="20.51056569591616" />
<me:PotentialPoint angle="90.0" potential="20.536512036575004" />
<me:PotentialPoint angle="95.0" potential="20.143970285707745" />
<me:PotentialPoint angle="100.0" potential="19.376770897942976" />
<me:PotentialPoint angle="105.0" potential="18.29201991147405" />
<me:PotentialPoint angle="110.0" potential="16.92037139010932" />
<me:PotentialPoint angle="115.0" potential="15.368316819887545" />
<me:PotentialPoint angle="120.0" potential="13.688766751446085" />
<me:PotentialPoint angle="125.0" potential="11.930588027520164" />
<me:PotentialPoint angle="130.0" potential="10.170859795437378" />
<me:PotentialPoint angle="135.0" potential="8.450695720191339" />
<me:PotentialPoint angle="140.0" potential="6.817480234214986" />
<me:PotentialPoint angle="145.0" potential="5.308072959735696" />
<me:PotentialPoint angle="150.0" potential="3.953120383486697" />
<me:PotentialPoint angle="155.0" potential="2.7757212924740284" />
<me:PotentialPoint angle="160.0" potential="1.7931839196672812" />
<me:PotentialPoint angle="165.0" potential="1.0177387089718744" />
<me:PotentialPoint angle="170.0" potential="0.458372601458932" />
<me:PotentialPoint angle="175.0" potential="0.11968182172231541" />
<me:PotentialPoint angle="180.0" potential="0.006413411853567238" />
<me:PotentialPoint angle="185.0" potential="0.11890306497076199" />
<me:PotentialPoint angle="190.0" potential="0.4555644696668281" />
<me:PotentialPoint angle="195.0" potential="1.0142561281100577" />
<me:PotentialPoint angle="200.0" potential="1.7889111390251815" />
<me:PotentialPoint angle="205.0" potential="2.772118633542557" />
<me:PotentialPoint angle="210.0" potential="3.9491990836354303" />
<me:PotentialPoint angle="215.0" potential="5.303714707372358" />
<me:PotentialPoint angle="220.0" potential="6.811364204498736" />
<me:PotentialPoint angle="225.0" potential="8.442469260602538" />
<me:PotentialPoint angle="230.0" potential="10.159378364922382" />
<me:PotentialPoint angle="235.0" potential="11.920534076339912" />
<me:PotentialPoint angle="240.0" potential="13.669378160642689" />
<me:PotentialPoint angle="245.0" potential="15.355875800915893" />
<me:PotentialPoint angle="250.0" potential="16.917045541623878" />
<me:PotentialPoint angle="255.0" potential="18.273140986871816" />
<me:PotentialPoint angle="260.0" potential="19.368738973006604" />
<me:PotentialPoint angle="265.0" potential="20.151351123334045" />
<me:PotentialPoint angle="270.0" potential="20.53131958263169" />
<me:PotentialPoint angle="275.0" potential="20.51383787766662" />
<me:PotentialPoint angle="280.0" potential="20.06994407811491" />
<me:PotentialPoint angle="285.0" potential="19.227732337975667" />
<me:PotentialPoint angle="290.0" potential="18.03045863278885" />
<me:PotentialPoint angle="295.0" potential="16.546269071060614" />
<me:PotentialPoint angle="300.0" potential="14.86058942374764" />
<me:PotentialPoint angle="305.0" potential="13.033191943751106" />
<me:PotentialPoint angle="310.0" potential="11.16180952092132" />
<me:PotentialPoint angle="315.0" potential="9.30790882435517" />

<me:PotentialPoint angle="320.0" potential="7.527983710445539" />
<me:PotentialPoint angle="325.0" potential="5.871453993623564" />
<me:PotentialPoint angle="330.0" potential="4.378255022461822" />
<me:PotentialPoint angle="335.0" potential="3.078182085153861" />
<me:PotentialPoint angle="340.0" potential="1.9924186810367484" />
<me:PotentialPoint angle="345.0" potential="1.136378590888218" />
<me:PotentialPoint angle="350.0" potential="0.5032112047352885" />
<me:PotentialPoint angle="355.0" potential="0.12968219210891674" />
<me:PotentialPoint angle="360.0" potential="0.010090187996970717" />
</me:HinderedRotorPotential>

</me:Rotor>
</me:RotorArray>
</me:DOSCMethod>
<me:DistributionCalcMethod default="true" name="Boltzmann" />
<me:energyTransferModel xsi:type="me:ExponentialDown">
<me:deltaEDown units="cm-1" >50</me:deltaEDown>
<me:deltaEDownTExponent referenceTemperature="298"
>1</me:deltaEDownTExponent>
</me:energyTransferModel>
</molecule>
<molecule spinMultiplicity="2" id="TS_COC=O_CO[C]=O">
<atomArray>
<atom id="a1" elementType="C" x3="1.910317" y3="-0.673993" z3="-
0.019049"/>
<atom id="a2" elementType="O" x3="0.467577" y3="-0.722668"
z3="0.027163"/>
<atom id="a3" elementType="H" x3="2.244991" y3="-1.185812"
z3="0.876812"/>
<atom id="a4" elementType="H" x3="2.245304" y3="0.361086" z3="-
0.024552"/>
<atom id="a5" elementType="H" x3="2.268801" y3="-1.179378" z3="-
0.913327"/>
<atom id="a6" elementType="C" x3="-0.130043" y3="-0.133002" z3="-
1.006043"/>
<atom id="a7" elementType="H" x3="-1.303313" y3="-0.232818" z3="-
0.864274"/>
<atom id="a8" elementType="O" x3="0.390662" y3="0.411795" z3="-
1.932681"/>
<atom id="a9" elementType="O" x3="-2.542037" y3="-0.551538" z3="-
0.346453"/>
<atom id="a10" elementType="H" x3="-2.234319" y3="-1.011003"
z3="0.450714"/>
</atomArray>
<bondArray>
<bond atomRefs2="a8 a6" order="2" />
<bond atomRefs2="a6 a7" order="1" />
<bond atomRefs2="a6 a2" order="1" id="bond62" />
<bond atomRefs2="a4 a1" order="1" />
<bond atomRefs2="a3 a1" order="1" />

<bond atomRefs2="a2 a1" order="1" id="bond21" />
<bond atomRefs2="a1 a5" order="1" />
<bond atomRefs2="a9 a10" order="1" />
<bond atomRefs2="a7 a6" order="1" />
<bond atomRefs2="a9 a7" order="1" id="bond97" />
</bondArray>
<propertyList>
<property dictRef="me:ZPE">
<scalar units="kJ/mol">6</scalar>
</property>
<property dictRef="me:spinMultiplicity">
<scalar units="cm-1">2</scalar>
</property>
<property dictRef="me:imFreqsScale">
<scalar units="cm-1" >1</scalar>
</property>
<property dictRef="me:hessian">
<matrix matrixType="squareSymmetricLT" rows="30" units="Hartree/Bohr2">
0.4307599

-0.0095135 0.6048293

0.0130839 -0.0164785 0.6241418

-0.1857626 -0.0185186 0.0286823 0.4281549

-0.0046094 -0.071843 0.0086893 -0.0376159 0.1625143

0.0048189 0.0084076 -0.0811669 0.074635 -0.1493231
0.3363363

-0.0653709 0.0331393 -0.0581363 -0.0267769 0.0194334
-0.0343871 0.0873657

0.0401574 -0.1169846 0.1112306 -0.0013784 0.0017762
-0.0027197 -0.0420029 0.1164799

-0.0701833 0.1109999 -0.2474332 0.0017218 -0.0022154
0.0038131 0.0741266 -0.1204581 0.2584194

	-0.06724	-0.0647483	-0.000536	-0.0309601	-0.032911
-0.0037617	0.004721	0.0093592	0.0012986	0.08532	
	-0.0742628	-0.3040819	-0.0004542	-0.0028305	0.0071277
0.0005445	-0.0031129	-0.011039	-0.0020872	0.0819937	0.3208473
	0.0003116	0.00086	-0.0524089	0.000676	0.0019258
0.000174	0.0082271	0.0257407	0.0022951	-0.0011338	-0.0009195
0.047186					
	-0.0705043	0.0350469	0.0603239	-0.0317988	0.018899
0.0260523	0.0049938	-0.005703	-0.0074828	0.0053368	-0.0035852
-0.0077267	0.0891016				
	0.0391134	-0.1134851	-0.1066071	0.0000188	0.0030995
0.0045805	-0.0058195	0.0089084	0.0137316	0.0092734	-0.0127931
-0.0262974	-0.0419515	0.1130856			
	0.0689893	-0.1076594	-0.2394588	0.0020678	0.0030601
0.0049014	0.0074135	-0.013731	-0.017974	-0.0007238	0.0018162
0.0012484	-0.0762203	0.1164059	0.2507035		
	-0.0485615	0.0218622	-0.0390213	-0.1502587	0.0280749
-0.050812	-0.0058688	-0.0011209	0.0017892	0.0019265	0.0005778
-0.0021061	0.0018807	0.0015032	-0.0014963	0.5609071	
	0.0171987	0.0028016	0.0035907	0.0451346	-0.1011728
0.0766118	-0.0023433	0.0000914	-0.0000421	-0.0053462	0.0000691
-0.0019566	-0.0036186	0.0014952	0.0006839	0.0981761	0.3493626
	-0.0310208	0.003814	-0.0030121	-0.0803072	0.0762742
-0.190709	0.0039147	0.0000015	0.0001479	0.0072171	0.0018017
0.002106	0.0082806	-0.0026845	0.0007729	-0.1596913	-0.3187257
0.7088962					
	0.0095377	0.001923	-0.003111	-0.0425628	0.0070355
-0.0135585	-0.0016391	-0.0000064	-0.0000297	-0.000951	0.0004334
0.0006658	-0.0009036	-0.0007643	-0.0000346	-0.021539	0.0011716
-0.0018178	0.0047229				
	0.0020599	0.0032482	0.0032908	0.0095753	0.0064137
0.0042704	0.0003412	0.0001371	0.0000345	0.0007053	-0.0002255
0.0003339	-0.0009264	-0.0002651	-0.0004444	0.0199614	-0.0465009
-0.0093988	-0.0242645	0.0323696			
	-0.0033648	0.0032566	-0.0005646	-0.0178066	0.0042029
0.0009795	-0.0006035	0.0000218	0.0001002	0.0007047	-0.0004163

-0.00016	-0.0002504	0.0003132	-0.0001749	-0.0339959	-0.0099003
-0.0360829	0.0411881	-0.0129074	0.0485502		
	0.0007096	0.0011125	-0.0018036	0.0304174	0.0038395
-0.0069086	0.0022004	0.0007091	-0.001296	0.0013817	0.0010052
0.001349	0.0014325	-0.001773	0.0000045	-0.238644	-0.1414722
0.2408488	-0.027854	-0.0199033	0.0333014	0.2216348	
	-0.0086061	-0.0043488	-0.0033523	0.0016642	-0.0069122
0.0569667	0.0002044	0.0006169	-0.0000993	0.0014119	0.0003053
0.0004856	0.0016219	-0.0001352	-0.0002981	-0.1406996	-0.206408
0.2444116	-0.0102166	0.0081325	0.0145508	0.1525428	0.2077427
	0.0149037	-0.003418	0.0000349	-0.0029406	0.0570077
-0.0723878	-0.0002598	-0.0001828	0.0007032	-0.0026983	-0.000241
-0.0005498	-0.0025855	0.0006117	0.0000394	0.2387859	0.2445963
-0.4779067	0.0167906	0.0142949	-0.0075238	-0.2585439	-0.3102003
0.5541321					
	-0.0025845	-0.000429	0.000912	0.0081842	-0.0027125
0.0050402	0.000322	-0.0000004	0.0000355	0.0001719	-0.0002657
-0.000293	0.0001671	0.0004097	0.0000224	-0.1001408	-0.005935
0.0073887	0.0810476	0.0191043	-0.0306308	0.0083713	0.0010429
-0.0018726	0.0759629				
	-0.0009137	-0.0000891	0.000054	0.0023084	-0.0003184
0.0002256	0.0000725	-0.0000173	0.0000632	0.0002081	-0.0001769
-0.0002077	0.0000046	0.0000508	0.000182	-0.0233379	0.0005225
0.0044062	0.0196909	-0.0025581	-0.0016745	0.0019946	0.0004354
-0.0016312	-0.0780538	0.1155371			
	0.0015725	0.0001373	-0.0001971	-0.0036966	-0.0000026
-0.000523	-0.0000676	0.0000267	0.0000212	-0.0001402	-0.0000452
0.0000294	-0.000281	0.0000123	-0.0000417	0.0378427	0.0049538
-0.0038443	-0.0315749	-0.0017271	-0.0016537	-0.003926	-0.0017608
0.0020912	0.1367734	-0.1966516	0.3435048		
	-0.0009836	0.0001254	-0.000394	0.0013633	0.0005665
-0.0011185	0.0000528	-0.0000136	0.0000202	0.0002932	0.000047
0.0000301	0.0002942	-0.0000103	-0.0000224	0.0002985	-0.0029657
0.0051872	0.0001412	-0.0066532	0.0114578	0.0003503	0.0010341
-0.0015796	-0.0715018	0.0780264	-0.1365021	0.0696917	
	-0.0006238	-0.0000466	0.0000366	0.0016421	-0.0006849
0.0004358	0.0000879	0.0000309	0.0000729	0.0000539	-0.000033
0.0000353	0.0002122	0.0000389	-0.0000153	-0.0049972	-0.0002608
0.0000998	0.0049974	-0.0007515	0.0025533	0.0019447	0.0005716
-0.0008374	0.0668395	-0.113386	0.1950571	-0.0701566	0.1145215
	0.0008891	0.0000806	0.0000648	-0.0030319	0.0003811
-0.0014178	-0.0002277	0.0000703	-0.0000928	-0.0002266	0.0000009
0.0000798	-0.0001101	-0.0000663	-0.0000163	0.0087051	0.0001881
-0.000368	-0.008518	0.0022532	-0.0034699	-0.0030256	-0.0007039
0.0013674	-0.1173757	0.1952341	-0.339387	0.1229214	-0.197438
0.3432398					

```
            </matrix>
      </property>
      <property dictRef="me:symmetryNumber" default="true">
        <scalar>1</scalar>
      </property>
    </propertyList>
    <me:DOSCMethod xsi:type="me:ClassicalCoupledRotors">
      <me:MCPoints>50000</me:MCPoints>
      <me:RotorArray>
          <me:Rotor bondRef="bond97" periodicity="1">
          </me:Rotor>
        <me:Rotor bondRef="bond62" periodicity="1">
          </me:Rotor>
                <me:ExpansionSize>
              <property dictRef="size">
                <array >5 5</array>
              </property>
                </me:ExpansionSize>
                <me:FourierSeries>
              <property dictRef="coeficients">
                <array units="kJ/mol">2619.6782 -353.3246 -1841.4319 -227.3149 81.4447 -
83.7345 308.8422 -48.7181 -31.7526 -4.0442 -72.6742 -10.6218 -16.4939 -32.8086 61.1373
66.3234 -22.6730 -12.2543 -15.2312 -41.5705 -8.5792 85.5125 -51.0517 -58.7109
87.7756</array>
              </property>
                </me:FourierSeries>
                <me:FourierSeries>
              <property dictRef="coeficients">
                <array units="kJ/mol">0.0000 22.6431 15.3555 -15.0946 -22.5255 0.0000
47.4771 26.1739 -25.8342 -47.5129 0.0000 43.9558 32.1265 -31.6296 -43.6768 0.0000
48.6088 24.0070 -23.0226 -47.9909 0.0000 42.1702 33.9275 -33.3267 -41.4070</array>
              </property>
                </me:FourierSeries>
                <me:FourierSeries>
              <property dictRef="coeficients">
                <array units="kJ/mol">0.0000 0.0000 0.0000 0.0000 0.0000 -2.9863 2.3819 -
0.0299 -0.1190 2.7749 1.9506 -7.5735 4.0968 4.8423 -7.0702 -7.9842 6.1453 0.9008 0.1374
6.6917 1.9719 -12.5520 8.1880 8.2069 -12.4706</array>
              </property>
                </me:FourierSeries>
                <me:FourierSeries>
              <property dictRef="coeficients">
                <array units="kJ/mol">0.0000 0.0000 0.0000 0.0000 0.0000 0.0000 -221.8357
42.4500 -6.9064 -14.5508 0.0000 107.4392 -28.6237 -19.8713 27.5754 0.0000 -11.0972
1.4855 6.6332 5.2775 0.0000 5.9870 -7.6769 5.0299 2.7567</array>
              </property>
                </me:FourierSeries>
</me:RotorArray>
</me:DOSCMethod>
<me:ExtraDOSCMethod xsi:type="HinderedRotorQM1D">
```

<me:bondRef>bond21</me:bondRef>
<me:periodicity>3</me:periodicity>
<me:HinderedRotorPotential format="numerical" units="kJ/mol" expansionSize="10" UseSineTerms="yes">
<me:PotentialPoint angle=" 0 " potential=" 0 "/>
<me:PotentialPoint angle=" 10 " potential=" 0.341315 "/>
<me:PotentialPoint angle=" 20 " potential=" 1.20773 "/>
<me:PotentialPoint angle=" 30 " potential=" 2.336695 "/>
<me:PotentialPoint angle=" 40 " potential=" 3.41315 "/>
<me:PotentialPoint angle=" 50 " potential=" 4.174545 "/>
<me:PotentialPoint angle=" 60 " potential=" 4.46335 "/>
<me:PotentialPoint angle=" 70 " potential=" 4.174545 "/>
<me:PotentialPoint angle=" 80 " potential=" 3.41315 "/>
<me:PotentialPoint angle=" 90 " potential=" 2.31044 "/>
<me:PotentialPoint angle=" 100 " potential=" 1.15522 "/>
<me:PotentialPoint angle=" 110 " potential=" 0.288805 "/>
<me:PotentialPoint angle=" 120 " potential=" 0 "/>
<me:PotentialPoint angle=" 130 " potential=" 0.393825 "/>
<me:PotentialPoint angle=" 140 " potential=" 1.286495 "/>
<me:PotentialPoint angle=" 150 " potential=" 2.46797 "/>
<me:PotentialPoint angle=" 160 " potential=" 3.544425 "/>
<me:PotentialPoint angle=" 170 " potential=" 4.25331 "/>
<me:PotentialPoint angle=" 180 " potential=" 4.437095 "/>
<me:PotentialPoint angle=" 190 " potential=" 4.09578 "/>
<me:PotentialPoint angle=" 200 " potential=" 3.281875 "/>
<me:PotentialPoint angle=" 210 " potential=" 2.179165 "/>
<me:PotentialPoint angle=" 220 " potential=" 1.076455 "/>
<me:PotentialPoint angle=" 230 " potential=" 0.26255 "/>
<me:PotentialPoint angle=" 240 " potential=" 0 "/>
<me:PotentialPoint angle=" 250 " potential=" 0.393825 "/>
<me:PotentialPoint angle=" 260 " potential=" 1.286495 "/>
<me:PotentialPoint angle=" 270 " potential=" 2.41546 "/>
<me:PotentialPoint angle=" 280 " potential=" 3.491915 "/>
<me:PotentialPoint angle=" 290 " potential=" 4.227055 "/>
<me:PotentialPoint angle=" 300 " potential=" 4.46335 "/>
<me:PotentialPoint angle=" 310 " potential=" 4.14829 "/>
<me:PotentialPoint angle=" 320 " potential=" 3.36064 "/>
<me:PotentialPoint angle=" 330 " potential=" 2.284185 "/>
<me:PotentialPoint angle=" 340 " potential=" 1.15522 "/>
<me:PotentialPoint angle=" 350 " potential=" 0.31506 "/>
<me:PotentialPoint angle=" 360 " potential=" 0 "/>
</me:HinderedRotorPotential>
<me:CalculateInternalRotorInertia phaseDifference="0"/>
</me:ExtraDOSCMethod>
</molecule>
<molecule id="C=O">
<atomArray>
<atom id="a1" elementType="C" x3="0.884908" y3="-0.010370" z3="-0.016378" />
<atom id="a2" elementType="O" x3="0.272592" y3="-1.070812" z3="-0.032340" />
<atom id="a3" elementType="H" x3="0.371698" y3="0.964383" z3="-0.001705" />

<atom id="a4" elementType="H" x3="1.985769" y3="0.032604" z3="-0.015731" />
</atomArray>
<bondArray>
<bond atomRefs2="a2 a1" order="2" />
<bond atomRefs2="a1 a3" order="1" />
<bond atomRefs2="a1 a4" order="1" />
</bondArray>
<propertyList>
<property dictRef="me:ZPE">
<scalar units="kJ/mol">0</scalar>
</property>
<property dictRef="me:vibFreqs">
<array units="cm-1">1209.9329 1273.8433 1544.5622 1879.0709 2979.513
3057.1366</array>
</property>
<property dictRef="me:spinMultiplicity">
<scalar units="cm-1">1</scalar>
</property>
<property dictRef="me:hessian">
<matrix matrixType="squareSymmetricLT" rows="42" units="Hartree/Bohr2">0.72
379528 0.16953201 0.75016322 0.09826020 0.33483327 0.37061037 -0.29362 248 -
0.28135724 -0.16362954 0.29179765 -0.28114757 -0.51395323 -0.2654 0275 0.32491366
0.54606588 -0.16355311 -0.26541591 -0.21135052 0.18896 801 0.30661245 0.19688622 -
0.29108039 0.02012286 0.01167252 -0.0226840 5 -0.02473247 -0.01443242 0.32473576
0.01698528 -0.06264270 -0.0020744 7 -0.04236378 0.00149693 -0.01029969 0.00500702
0.05426960 0.00977864 -0.00192730 -0.06015986 -0.02478595 -0.01032749 0.01314791
0.00300090 0.01974627 0.03171732 -0.13909242 0.09170236 0.05369683 0.02450888 -0.
01903362 -0.01098248 -0.01097131 0.02037147 0.01200641 0.12555485 0.09 463027 -
0.17356729 -0.06735605 -0.00119264 -0.03360957 -0.03089685 -0. 00039742 0.00687617 -
0.00749148 -0.09304021 0.20030068 0.05551428 -0.0 6749006 -0.09909999 -0.00055252 -
0.03088221 0.00131639 -0.00024100 -0. 00737211 0.01529462 -0.05472075 0.10574438
0.08248898</matrix>
</property>
</propertyList>
</molecule>
<molecule spinMultiplicity="2" id="OOCO[C]=O">
<atomArray>
<atom id="a1" elementType="C" x3="-9.150850" y3="-8.904300" z3="12.682390" />
<atom id="a2" elementType="O" x3="-9.968640" y3="-8.525180" z3="11.576130" />
<atom id="a3" elementType="H" x3="-8.877780" y3="-7.986260" z3="13.202410" />
<atom id="a4" elementType="H" x3="-9.698010" y3="-9.608460" z3="13.314940" />
<atom id="a5" elementType="C" x3="-10.940380" y3="-9.378000" z3="11.239620" />
<atom id="a6" elementType="O" x3="-11.669000" y3="-9.290880" z3="10.314910" />
<atom id="a7" elementType="H" x3="-7.995610" y3="-10.666380" z3="10.826500" />
<atom id="a8" elementType="O" x3="-7.952140" y3="-9.447170" z3="12.250890" />
<atom id="a9" elementType="O" x3="-8.248800" y3="-10.751830" z3="11.758390" />
</atomArray>
<bondArray>
<bond atomRefs2="a6 a5" order="2" />
<bond atomRefs2="a7 a9" order="1" />

<bond atomRefs2="a5 a2" order="1" id="bond52" />
<bond atomRefs2="a2 a1" order="1" id="bond21" />
<bond atomRefs2="a9 a8" order="1" id="bond98" />
<bond atomRefs2="a8 a1" order="1" id="bond81" />
<bond atomRefs2="a1 a3" order="1" />
<bond atomRefs2="a1 a4" order="1" />
</bondArray>
<propertyList>
<property dictRef="me:ZPE">
<scalar units="kJ/mol">-229.22</scalar>
</property>
<property dictRef="me:vibFreqs">
<array units="cm-1">77.9686 136.6058 229.4275 260.2708 323.3685 425.4738
541.7552 690.8477 959.0794 1039.7141 1112.7654 1157.0997 1199.6578 1327.7613
1414.7341 1433.608 1484.7952 1962.0863 3127.1979 3211.1534 3818.6002</array>
</property>
<property dictRef="me:spinMultiplicity">
<scalar units="cm-1">2</scalar>
</property>
<property dictRef="me:hessian">
<matrix matrixType="squareSymmetricLT" rows="27"
units="Hartree/Bohr2">0.51621531 0.12260976 0.67120491 -0.05798525 0.04246063
0.54537766 -0.07613960 0.00688150 -0.05761144 0.34699809 -0.00029258 -0.10104803
0.04003666 0.12463596 0.25479245 -0.03027117 0.04576527 -0.17128993 0.19235980 -
0.04330418 0.28263898 -0.06898513 -0.06892126 -0.03008973 -0.01222903 -0.01460923 -
0.01611949 0.07994948 -0.06412455 -0.25838556 -0.11476868 0.00573376 0.01138520
0.00897556 0.05783252 0.27282392 -0.02515699 -0.10169406 -0.10931428 -0.01912033 -
0.02053219 -0.01851439 0.03825227 0.12265221 0.12439949 -0.11339970 -0.09236573
0.08135919 0.00375568 0.01150668 -0.02356072 -0.00036620 -0.00303461 0.00314573
0.13846107 -0.09596572 -0.17769817 0.10489805 -0.00066100 -0.00307008 0.00887101 -
0.01129357 -0.01053360 0.01395235 0.09296372 0.17774118 0.07326602 0.09089385 -
0.13687255 -0.00155773 0.02168500 -0.02800557 -0.00768134 -0.00843072 0.01225246 -
0.08147247 -0.11290562 0.15456631 -0.04656474 -0.00139614 -0.02797506 -0.13315404 -
0.12007431 -0.04202898 0.00272896 -0.00016343 0.00208537 0.00262710 -0.00122853
0.00489940 0.51538682 -0.01084617 0.01171000 -0.01864096 -0.14441662 -0.18444990
0.01300746 0.00142555 0.00010286 0.00233431 0.00586245 0.00089770 0.00502824
0.09001980 0.24585758 -0.03731882 -0.02327453 -0.03072937 -0.02442051 0.03863616 -
0.05161839 -0.00337677 0.00119219 -0.00299016 -0.00061682 -0.00261259 0.00101627
0.48509178 -0.10888994 0.68171434 0.01266275 -0.01316734 0.00843010 -0.07106388 -
0.02031319 -0.05748605 -0.00216790 0.00111291 -0.00198226 -0.00061590 0.00031324 -
0.00116310 -0.34647956 0.04419306 -0.42860720 0.41213084 -0.00104397 -0.00155322
0.00396002 -0.01771899 0.01946440 -0.04248000 -0.00025331 0.00019010 -0.00083486 -
0.00122072 -0.00005694 -0.00115327 0.03734565 -0.06853847 0.09808504 -0.01493230
0.04977334 0.00918506 -0.00518944 0.01546929 -0.05943270 -0.04595975 -0.03114609 -
0.00078568 0.00038287 -0.00070053 -0.00023612 0.00044522 -0.00024267 -0.42218414
0.10400581 -0.60037986 0.48087779 -0.05668412 0.61896959 -0.00132528 0.00083367
0.00403634 0.00041057 -0.00019478 -0.00105298 0.00014827 0.00032953 -0.00023613
0.00038829 0.00019336 -0.00038576 -0.00122779 0.00037709 -0.00009380 0.00020235 -
0.00003509 0.00022320 0.04243607 -0.00516368 -0.00570737 -0.00690338 -0.00012845
0.00050675 0.00175102 0.00198662 -0.00105118 -0.00001839 -0.00025487 -0.00031230

0.00000918 0.00162824 0.00018055 0.00060941 -0.00036562 0.00001706 -0.00040007
0.02630410 0.06656456 0.00272364 -0.00198084 -0.00049025 -0.00155667 -0.00023845 -
0.00123745 -0.00016355 0.00015121 0.00034492 -0.00075348 -0.00009573 0.00005661
0.00160575 0.00071699 0.00114231 -0.00055702 -0.00008899 -0.00100927 -0.13016395 -
0.03413505 0.48381167 -0.23129325 0.00707404 0.06751816 -0.06282481 0.01896658 -
0.02437391 0.00532878 -0.00109125 0.00075612 -0.03341393 0.01499656 0.01416831
0.00789125 0.01397492 0.00659323 -0.00483769 -0.00265112 -0.00600295 0.00023702
0.01386405 0.00194490 0.37983074 0.05487906 -0.09604136 -0.03620945 0.02744639 -
0.00062639 0.00815476 0.02962080 -0.01679481 -0.01554772 -0.01217154 0.00896631
0.00226975 -0.00529073 -0.00580232 -0.00316956 0.00237904 0.00085002 0.00216323 -
0.00988104 -0.01445947 0.00261002 -0.01892500 0.35814147 0.08206645 -0.01604894 -
0.09564175 -0.03281559 0.00468170 0.01492646 0.01356355 -0.00748443 -0.00692929
0.02538355 -0.01306716 -0.00308213 0.00010195 0.00397191 0.00184214 -0.00082524 -
0.00116815 -0.00197587 -0.01608536 -0.05005137 -0.00624191 -0.06922520 0.14039402
0.18031885 0.00882964 0.03845150 0.01231768 0.00424702 0.00037488 0.00253350 -
0.00440721 0.00340513 0.00225622 0.00256359 0.00068194 -0.00007332 -0.00120801 -
0.00059008 0.00274890 0.00016899 0.00050984 -0.00164444 -0.04126948 -0.03787040
0.12692038 -0.06091810 -0.06805699 -0.00216412 0.09199356 -0.00005215 -0.04248119 -
0.01483289 -0.00177254 0.00304561 -0.00074091 0.00421188 0.00226307 -0.00031166 -
0.00128537 0.00406590 0.00260360 -0.00084055 0.00004200 -0.00057618 0.00078020 -
0.00014628 0.00123625 -0.01792685 -0.04573858 0.03306083 -0.04620878 -0.23423346 -
0.06122758 0.06309418 0.31318293 -0.01650893 -0.03093195 -0.01650883 0.00415518
0.00499505 0.00424639 0.00640074 -0.00267021 0.00145179 -0.00324886 0.00051447
0.00031126 -0.00159607 -0.00153382 0.00000272 0.00131299 0.00036433 0.00101542
0.14375842 0.08913865 -0.47637663 0.00862134 -0.10066506 -0.08321650 -0.14289481
0.04078854 0.56907438</matrix>
</property>
<property dictRef="me:sigma" default="true">
<scalar>5.0</scalar>
</property>
<property dictRef="me:epsilon" default="true">
<scalar>50.0</scalar>
</property>
<property dictRef="me:vibFreqsFromHessian">
<scalar calculated="20220120_074051" units="cm-1">77.9683 136.605 229.427 260.27
323.367 425.472 541.753 690.845 959.076 1039.71 1112.76 1157.1 1199.65 1327.76
1414.73 1433.6 1484.79 1962.08 3127.19 3211.14 3818.59 </scalar>
</property>
<property dictRef="me:frequenciesScaleFactor" default="true">
<scalar>0.9522</scalar>
</property>
<property dictRef="me:symmetryNumber" default="true">
<scalar>1</scalar>
</property>
</propertyList>
<me:DOSCMethod xsi:type="me:ClassicalCoupledRotors">
<me:MCPoints>10000</me:MCPoints>
<me:RotorArray>
<me:Rotor>
<bondRef>bond52</bondRef>

<me:HinderedRotorPotential format="numerical" units="kJ/mol" expansionSize="10" UseSineTerms="yes">
<me:PotentialPoint angle="0" potential="0.0" />
<me:PotentialPoint angle="5.0" potential="0.2470854026517092" />
<me:PotentialPoint angle="10.0" potential="0.9649921771130708" />
<me:PotentialPoint angle="15.0" potential="2.0951074466807182" />
<me:PotentialPoint angle="20.0" potential="3.558672793625146" />
<me:PotentialPoint angle="25.0" potential="5.34649596219099" />
<me:PotentialPoint angle="30.0" potential="7.531818312332662" />
<me:PotentialPoint angle="35.0" potential="9.871970157085686" />
<me:PotentialPoint angle="40.0" potential="12.454456190522171" />
<me:PotentialPoint angle="45.0" potential="15.074784877458214" />
<me:PotentialPoint angle="50.0" potential="17.6277550730026" />
<me:PotentialPoint angle="55.0" potential="20.030243603373705" />
<me:PotentialPoint angle="60.0" potential="22.066733798220127" />
<me:PotentialPoint angle="65.0" potential="23.846165759120318" />
<me:PotentialPoint angle="70.0" potential="25.178859403331128" />
<me:PotentialPoint angle="75.0" potential="26.08111746047603" />
<me:PotentialPoint angle="80.0" potential="26.53822454274632" />
<me:PotentialPoint angle="85.0" potential="26.517770623063754" />
<me:PotentialPoint angle="90.0" potential="26.015350744357725" />
<me:PotentialPoint angle="95.0" potential="25.051294452919397" />
<me:PotentialPoint angle="100.0" potential="23.666215818408045" />
<me:PotentialPoint angle="105.0" potential="21.918256439203585" />
<me:PotentialPoint angle="110.0" potential="19.88099653528004" />
<me:PotentialPoint angle="115.0" potential="17.63760447695797" />
<me:PotentialPoint angle="120.0" potential="15.269601863294872" />
<me:PotentialPoint angle="125.0" potential="12.857596046726483" />
<me:PotentialPoint angle="130.0" potential="10.475764664821513" />
<me:PotentialPoint angle="135.0" potential="8.195362437466834" />
<me:PotentialPoint angle="140.0" potential="6.074972458364886" />
<me:PotentialPoint angle="145.0" potential="4.1606351478040775" />
<me:PotentialPoint angle="150.0" potential="2.5010573349890124" />
<me:PotentialPoint angle="155.0" potential="1.1299006811192134" />
<me:PotentialPoint angle="160.0" potential="0.07154114729559904" />
<me:PotentialPoint angle="165.0" potential="-0.6506677877729209" />
<me:PotentialPoint angle="170.0" potential="-1.0038559281785626" />
<me:PotentialPoint angle="175.0" potential="-0.9930226484592216" />
<me:PotentialPoint angle="180.0" potential="-0.590784083504925" />
<me:PotentialPoint angle="185.0" potential="0.19372652296999762" />
<me:PotentialPoint angle="190.0" potential="1.3642534884805493" />
<me:PotentialPoint angle="195.0" potential="2.914008855131865" />
<me:PotentialPoint angle="200.0" potential="4.832440862280562" />
<me:PotentialPoint angle="205.0" potential="7.09305390586138" />
<me:PotentialPoint angle="210.0" potential="9.665370359665069" />
<me:PotentialPoint angle="215.0" potential="12.494559628818275" />
<me:PotentialPoint angle="220.0" potential="12.155416801712164" />
<me:PotentialPoint angle="225.0" potential="13.415991163081415" />
<me:PotentialPoint angle="230.0" potential="14.82163378996161" />
<me:PotentialPoint angle="235.0" potential="16.34944341282462" />

```
      <me:PotentialPoint angle="240.0" potential="17.954374689194577" />
      <me:PotentialPoint angle="245.0" potential="19.572427265398314" />
      <me:PotentialPoint angle="250.0" potential="21.122720659739326" />
      <me:PotentialPoint angle="255.0" potential="20.70767550894268" />
      <me:PotentialPoint angle="260.0" potential="21.8542470124412" />
      <me:PotentialPoint angle="265.0" potential="22.60296108222236" />
      <me:PotentialPoint angle="270.0" potential="22.929207068531362" />
      <me:PotentialPoint angle="275.0" potential="22.793734825068555" />
      <me:PotentialPoint angle="280.0" potential="22.202600659597227" />
      <me:PotentialPoint angle="285.0" potential="21.147522353772732" />
      <me:PotentialPoint angle="290.0" potential="19.666413857587496" />
      <me:PotentialPoint angle="295.0" potential="17.79423666216791" />
      <me:PotentialPoint angle="300.0" potential="15.587434482999518" />
      <me:PotentialPoint angle="305.0" potential="13.119946774249213" />
      <me:PotentialPoint angle="310.0" potential="10.472764957771725" />
      <me:PotentialPoint angle="315.0" potential="7.737728034124776" />
      <me:PotentialPoint angle="320.0" potential="5.002469840171424" />
      <me:PotentialPoint angle="325.0" potential="2.355663790434786" />
      <me:PotentialPoint angle="330.0" potential="-0.11859361438926953" />
      <me:PotentialPoint angle="335.0" potential="-2.3428642698838416" />
      <me:PotentialPoint angle="340.0" potential="-4.248279415494503" />
      <me:PotentialPoint angle="345.0" potential="-5.776116188057299" />
      <me:PotentialPoint angle="350.0" potential="-6.879693776740078" />
      <me:PotentialPoint angle="355.0" potential="-7.522908364089206" />
      <me:PotentialPoint angle="360.0" potential="-7.68326450352668" />
     </me:HinderedRotorPotential>

    </me:Rotor>
    <me:Rotor>
     <bondRef>bond21</bondRef>
     <me:HinderedRotorPotential format="numerical" units="kJ/mol" expansionSize="10"
UseSineTerms="yes">
      <me:PotentialPoint angle="0" potential="0.0" />
      <me:PotentialPoint angle="5.0" potential="0.0012962099011899347" />
      <me:PotentialPoint angle="10.0" potential="0.005343145055839557" />
      <me:PotentialPoint angle="15.0" potential="0.013968497792074226" />
      <me:PotentialPoint angle="20.0" potential="0.02241917063079888" />
      <me:PotentialPoint angle="25.0" potential="0.038726477385543474" />
      <me:PotentialPoint angle="30.0" potential="0.055367196392588906" />
      <me:PotentialPoint angle="35.0" potential="0.07991546335576137" />
      <me:PotentialPoint angle="40.0" potential="0.1008209050394389" />
      <me:PotentialPoint angle="45.0" potential="0.1285689193692128" />
      <me:PotentialPoint angle="50.0" potential="0.1541240606994581 7" />
      <me:PotentialPoint angle="55.0" potential="0.18212733459870376" />
      <me:PotentialPoint angle="60.0" potential="0.20559598950440533" />
      <me:PotentialPoint angle="65.0" potential="0.22989897095187123" />
      <me:PotentialPoint angle="70.0" potential="0.25173457829876367" />
      <me:PotentialPoint angle="75.0" potential="0.2712859651349395" />
      <me:PotentialPoint angle="80.0" potential="0.28402276778262275" />
      <me:PotentialPoint angle="85.0" potential="0.29557530876143334" />
```

<me:PotentialPoint angle="90.0" potential="0.30407934705916495" />
<me:PotentialPoint angle="95.0" potential="0.3057759381086097" />
<me:PotentialPoint angle="100.0" potential="0.30418703852366097" />
<me:PotentialPoint angle="105.0" potential="0.29771455191941887" />
<me:PotentialPoint angle="110.0" potential="0.2832940839104813" />
<me:PotentialPoint angle="115.0" potential="0.26346660672516015" />
<me:PotentialPoint angle="120.0" potential="0.2362907937438458" />
<me:PotentialPoint angle="125.0" potential="0.20155829685749757" />
<me:PotentialPoint angle="130.0" potential="0.1586645044534129" />
<me:PotentialPoint angle="135.0" potential="0.10550452861652973" />
<me:PotentialPoint angle="140.0" potential="0.04599277384692595" />
<me:PotentialPoint angle="145.0" potential="-0.020409860264956222" />
<me:PotentialPoint angle="150.0" potential="-0.09293991594740411" />
<me:PotentialPoint angle="155.0" potential="-0.16890205093103614" />
<me:PotentialPoint angle="160.0" potential="-0.24662444300130495" />
<me:PotentialPoint angle="165.0" potential="-0.32230201872734177" />
<me:PotentialPoint angle="170.0" potential="-0.3937657565769248" />
<me:PotentialPoint angle="175.0" potential="-0.45605683851452516" />
<me:PotentialPoint angle="180.0" potential="-0.5113793069185745" />
<me:PotentialPoint angle="185.0" potential="-0.5484939710570115" />
<me:PotentialPoint angle="190.0" potential="-0.5689487441565348" />
<me:PotentialPoint angle="195.0" potential="-0.5663597361169248" />
<me:PotentialPoint angle="200.0" potential="-0.5421586269303688" />
<me:PotentialPoint angle="205.0" potential="-0.48997702013571587" />
<me:PotentialPoint angle="210.0" potential="-0.4142539167599272" />
<me:PotentialPoint angle="215.0" potential="-0.30860706386114256" />
<me:PotentialPoint angle="220.0" potential="-0.1809798375346338" />
<me:PotentialPoint angle="225.0" potential="-0.026469952290016224" />
<me:PotentialPoint angle="230.0" potential="0.1444891172731761" />
<me:PotentialPoint angle="235.0" potential="0.3314241166112031" />
<me:PotentialPoint angle="240.0" potential="0.5219558454583751" />
<me:PotentialPoint angle="245.0" potential="0.7109478815698461" />
<me:PotentialPoint angle="250.0" potential="0.8862994704923781" />
<me:PotentialPoint angle="255.0" potential="1.0392631914204902" />
<me:PotentialPoint angle="260.0" potential="1.1622094445689062" />
<me:PotentialPoint angle="265.0" potential="1.253081973138251" />
<me:PotentialPoint angle="270.0" potential="1.3084819557155842" />
<me:PotentialPoint angle="275.0" potential="1.3335058822995052" />
<me:PotentialPoint angle="280.0" potential="1.3296904593858918" />
<me:PotentialPoint angle="285.0" potential="1.2907184137244372" />
<me:PotentialPoint angle="290.0" potential="1.2305276850215552" />
<me:PotentialPoint angle="295.0" potential="1.1427306479402741" />
<me:PotentialPoint angle="300.0" potential="1.0385713153170344" />
<me:PotentialPoint angle="305.0" potential="0.9154864355498258" />
<me:PotentialPoint angle="310.0" potential="0.7856738152370174" />
<me:PotentialPoint angle="315.0" potential="0.6494063429611782" />
<me:PotentialPoint angle="320.0" potential="0.5169322881980966" />
<me:PotentialPoint angle="325.0" potential="0.39173149911880284" />
<me:PotentialPoint angle="330.0" potential="0.28105637072408246" />
<me:PotentialPoint angle="335.0" potential="0.18516939855215653" />

```
      <me:PotentialPoint angle="340.0" potential="0.11129334897117799" />
      <me:PotentialPoint angle="345.0" potential="0.05903158962964686" />
      <me:PotentialPoint angle="350.0" potential="0.022820030649018233" />
      <me:PotentialPoint angle="355.0" potential="0.006085008609208798" />
      <me:PotentialPoint angle="360.0" potential="0.0028620450843248693" />
    </me:HinderedRotorPotential>

  </me:Rotor>
  <me:Rotor>
    <bondRef>bond98</bondRef>
    <me:HinderedRotorPotential format="numerical" units="kJ/mol" expansionSize="10"
UseSineTerms="yes">
      <me:PotentialPoint angle="0" potential="0.0" />
      <me:PotentialPoint angle="5.0" potential="0.023805719889393367" />
      <me:PotentialPoint angle="10.0" potential="0.08099479836177866" />
      <me:PotentialPoint angle="15.0" potential="0.2875148085844143" />
      <me:PotentialPoint angle="20.0" potential="0.39200740850624904" />
      <me:PotentialPoint angle="25.0" potential="0.6270109873527736" />
      <me:PotentialPoint angle="30.0" potential="0.9174836067408281" />
      <me:PotentialPoint angle="35.0" potential="1.2408117204878377" />
      <me:PotentialPoint angle="40.0" potential="1.5847901824096151" />
      <me:PotentialPoint angle="45.0" potential="1.9384870317405563" />
      <me:PotentialPoint angle="50.0" potential="2.291286297126055" />
      <me:PotentialPoint angle="55.0" potential="2.635987256911069" />
      <me:PotentialPoint angle="60.0" potential="2.9657262133914273" />
      <me:PotentialPoint angle="65.0" potential="3.2773736744106685" />
      <me:PotentialPoint angle="70.0" potential="3.5670900831110646" />
      <me:PotentialPoint angle="75.0" potential="3.8331516872460996" />
      <me:PotentialPoint angle="80.0" potential="4.072504713345323" />
      <me:PotentialPoint angle="85.0" potential="4.284217903859588" />
      <me:PotentialPoint angle="90.0" potential="4.46674434338549" />
      <me:PotentialPoint angle="95.0" potential="4.618152509808645" />
      <me:PotentialPoint angle="100.0" potential="4.738787658808766" />
      <me:PotentialPoint angle="105.0" potential="4.8274934434366" />
      <me:PotentialPoint angle="110.0" potential="4.8844939654608295" />
      <me:PotentialPoint angle="115.0" potential="4.911745153272082" />
      <me:PotentialPoint angle="120.0" potential="4.910130832120817" />
      <me:PotentialPoint angle="125.0" potential="4.8874388339804655" />
      <me:PotentialPoint angle="130.0" potential="4.850887795545572" />
      <me:PotentialPoint angle="135.0" potential="4.811384326382309" />
      <me:PotentialPoint angle="140.0" potential="4.782635282254331" />
      <me:PotentialPoint angle="145.0" potential="4.7815337556581055" />
      <me:PotentialPoint angle="150.0" potential="4.827353560150992" />
      <me:PotentialPoint angle="155.0" potential="4.939644101968743" />
      <me:PotentialPoint angle="160.0" potential="5.143437391917821" />
      <me:PotentialPoint angle="165.0" potential="5.460976845845858" />
      <me:PotentialPoint angle="170.0" potential="5.914248442074331" />
      <me:PotentialPoint angle="175.0" potential="6.52297376898256" />
      <me:PotentialPoint angle="180.0" potential="7.3031146818617145" />
      <me:PotentialPoint angle="185.0" potential="8.265227156322098" />
```

<me:PotentialPoint angle="190.0" potential="9.412741781433542" />
<me:PotentialPoint angle="195.0" potential="10.740262427586872" />
<me:PotentialPoint angle="200.0" potential="12.232026602728224" />
<me:PotentialPoint angle="205.0" potential="13.860743360473887" />
<me:PotentialPoint angle="210.0" potential="15.586952178902498" />
<me:PotentialPoint angle="215.0" potential="17.359106519150206" />
<me:PotentialPoint angle="220.0" potential="19.114614554050796" />
<me:PotentialPoint angle="225.0" potential="20.786707917922495" />
<me:PotentialPoint angle="230.0" potential="22.29831940585513" />
<me:PotentialPoint angle="235.0" potential="23.61012025199887" />
<me:PotentialPoint angle="240.0" potential="24.714090334864224" />
<me:PotentialPoint angle="245.0" potential="25.578772251906436" />
<me:PotentialPoint angle="250.0" potential="26.166669154898113" />
<me:PotentialPoint angle="255.0" potential="26.45123228104785" />
<me:PotentialPoint angle="260.0" potential="26.416448852675295" />
<me:PotentialPoint angle="265.0" potential="26.051286346507677" />
<me:PotentialPoint angle="270.0" potential="25.35643677068556" />
<me:PotentialPoint angle="275.0" potential="24.343199175278237" />
<me:PotentialPoint angle="280.0" potential="23.033474810808325" />
<me:PotentialPoint angle="285.0" potential="21.459425564606352" />
<me:PotentialPoint angle="290.0" potential="19.662591776907288" />
<me:PotentialPoint angle="295.0" potential="17.686223502385616" />
<me:PotentialPoint angle="300.0" potential="15.598786059897305" />
<me:PotentialPoint angle="305.0" potential="13.455789767508127" />
<me:PotentialPoint angle="310.0" potential="11.319827629808588" />
<me:PotentialPoint angle="315.0" potential="9.25038569226981" />
<me:PotentialPoint angle="320.0" potential="7.305379672404014" />
<me:PotentialPoint angle="325.0" potential="5.534510564203791" />
<me:PotentialPoint angle="330.0" potential="3.9807246454953455" />
<me:PotentialPoint angle="335.0" potential="2.674113846109764" />
<me:PotentialPoint angle="340.0" potential="1.6335886265655826" />
<me:PotentialPoint angle="345.0" potential="0.8649588070767" />
<me:PotentialPoint angle="350.0" potential="0.3585348380917264" />
<me:PotentialPoint angle="355.0" potential="0.0874887026084842" />
<me:PotentialPoint angle="360.0" potential="-0.0014560647159652807" />
</me:HinderedRotorPotential>

</me:Rotor>
<me:Rotor>
<bondRef>bond81</bondRef>
<me:HinderedRotorPotential format="numerical" units="kJ/mol" expansionSize="10" UseSineTerms="yes">
<me:PotentialPoint angle="0" potential="0.0" />
<me:PotentialPoint angle="5.0" potential="0.14535024584885647" />
<me:PotentialPoint angle="10.0" potential="0.31040136252986483" />
<me:PotentialPoint angle="15.0" potential="0.601250383351717" />
<me:PotentialPoint angle="20.0" potential="1.0010326471428674" />
<me:PotentialPoint angle="25.0" potential="1.4434428806342814" />
<me:PotentialPoint angle="30.0" potential="2.0493282960125705" />
<me:PotentialPoint angle="35.0" potential="2.7780055147606597" />

<me:PotentialPoint angle="40.0" potential="3.6250819618656633" />
<me:PotentialPoint angle="45.0" potential="4.549322239892002" />
<me:PotentialPoint angle="50.0" potential="5.537153536834582" />
<me:PotentialPoint angle="55.0" potential="6.473242628330183" />
<me:PotentialPoint angle="60.0" potential="7.397777030403221" />
<me:PotentialPoint angle="65.0" potential="8.212636816814289" />
<me:PotentialPoint angle="70.0" potential="8.932957179326257" />
<me:PotentialPoint angle="75.0" potential="9.491694486973318" />
<me:PotentialPoint angle="80.0" potential="9.908484926603647" />
<me:PotentialPoint angle="85.0" potential="10.137366160842824" />
<me:PotentialPoint angle="90.0" potential="10.208289651774923" />
<me:PotentialPoint angle="95.0" potential="10.108046658865312" />
<me:PotentialPoint angle="100.0" potential="9.85629254288163" />
<me:PotentialPoint angle="105.0" potential="9.435128416654132" />
<me:PotentialPoint angle="110.0" potential="8.904455211609976" />
<me:PotentialPoint angle="115.0" potential="8.25607548614095" />
<me:PotentialPoint angle="120.0" potential="7.543289886549799" />
<me:PotentialPoint angle="125.0" potential="6.761127474390399" />
<me:PotentialPoint angle="130.0" potential="5.978396423221157" />
<me:PotentialPoint angle="135.0" potential="5.182993418141952" />
<me:PotentialPoint angle="140.0" potential="4.4572919214609685" />
<me:PotentialPoint angle="145.0" potential="3.781111604579364" />
<me:PotentialPoint angle="150.0" potential="3.231100774556564" />
<me:PotentialPoint angle="155.0" potential="2.772499339712187" />
<me:PotentialPoint angle="160.0" potential="2.4799112321147145" />
<me:PotentialPoint angle="165.0" potential="2.3102461417586775" />
<me:PotentialPoint angle="170.0" potential="2.3204836770541157" />
<me:PotentialPoint angle="175.0" potential="2.46440528859869" />
<me:PotentialPoint angle="180.0" potential="2.7805476900355868" />
<me:PotentialPoint angle="185.0" potential="3.2579754774200382" />
<me:PotentialPoint angle="190.0" potential="3.8766335255556803" />
<me:PotentialPoint angle="195.0" potential="4.5540441813980195" />
<me:PotentialPoint angle="200.0" potential="5.371497958902629" />
<me:PotentialPoint angle="205.0" potential="6.260892351462356" />
<me:PotentialPoint angle="210.0" potential="7.191344892020469" />
<me:PotentialPoint angle="215.0" potential="8.130114674329986" />
<me:PotentialPoint angle="220.0" potential="9.047617895251836" />
<me:PotentialPoint angle="225.0" potential="9.87622969896788" />
<me:PotentialPoint angle="230.0" potential="10.651347428994791" />
<me:PotentialPoint angle="235.0" potential="11.307081649969467" />
<me:PotentialPoint angle="240.0" potential="11.86632870412793" />
<me:PotentialPoint angle="245.0" potential="12.306828310885038" />
<me:PotentialPoint angle="250.0" potential="12.599560430838384" />
<me:PotentialPoint angle="255.0" potential="12.770443590054636" />
<me:PotentialPoint angle="260.0" potential="12.810458620544521" />
<me:PotentialPoint angle="265.0" potential="12.718784279082575" />
<me:PotentialPoint angle="270.0" potential="12.492424543790317" />
<me:PotentialPoint angle="275.0" potential="12.144420379873312" />
<me:PotentialPoint angle="280.0" potential="11.678507450908267" />
<me:PotentialPoint angle="285.0" potential="11.093844818941001" />

<me:PotentialPoint angle="290.0" potential="10.407075599245994" />
<me:PotentialPoint angle="295.0" potential="9.629324128533" />
<me:PotentialPoint angle="300.0" potential="8.728613893366445" />
<me:PotentialPoint angle="305.0" potential="7.788336077526676" />
<me:PotentialPoint angle="310.0" potential="6.7996835133628775" />
<me:PotentialPoint angle="315.0" potential="5.793435681913528" />
<me:PotentialPoint angle="320.0" potential="4.776791328846151" />
<me:PotentialPoint angle="325.0" potential="3.8016617744247085" />
<me:PotentialPoint angle="330.0" potential="2.891020855937783" />
<me:PotentialPoint angle="335.0" potential="2.0524195779326035" />
<me:PotentialPoint angle="340.0" potential="1.3401772362650852" />
<me:PotentialPoint angle="345.0" potential="0.755302172254476" />
<me:PotentialPoint angle="350.0" potential="0.3356811233776671" />
<me:PotentialPoint angle="355.0" potential="0.07282670731240366" />
<me:PotentialPoint angle="360.0" potential="0.001002127949789883" />
</me:HinderedRotorPotential>

</me:Rotor>
</me:RotorArray>
</me:DOSCMethod>
<me:DOSCMethod default="true" name="ClassicalRotors" />
<me:DistributionCalcMethod default="true" name="Boltzmann" />
<me:energyTransferModel name="ExponentialDown" default="true" />
<me:deltaEDown default="NEEDS TO BE CHECKED**">130.0</me:deltaEDown>
</molecule>
<molecule id="[OH]">
<atomArray>
<atom id="a1" elementType="O" />
<atom id="a2" elementType="H" />
</atomArray>
<bondArray>
<bond atomRefs2="a1 a2" order="1" />
</bondArray>
<propertyList>
<property dictRef="me:ZPE">
<scalar units="kJ/mol">0.0</scalar>
</property>
<property dictRef="me:rotConsts">
<array units="cm-1">19.2438</array>
</property>
<property dictRef="me:symmetryNumber">
<scalar>1</scalar>
</property>
<property dictRef="me:vibFreqs">
<array units="cm-1">3722.0</array>
</property>
<property dictRef="me:MW">
<scalar units="amu">17</scalar>
</property>
<property dictRef="me:spinMultiplicity">

```
      <scalar>2</scalar>
    </property>
    <property dictRef="me:electronicExcitation">
      <array units="cm-1">139.7</array>
    </property>
    <property dictRef="me:frequenciesScaleFactor" default="true">
      <scalar>0.9522</scalar>
    </property>
  </propertyList>
  <me:DOSCMethod xsi:type="ClassicalRotors" />
</molecule>
<molecule spinMultiplicity="2" id="CH3_post_comp">
  <atomArray>
    <atom id="a1" elementType="C" spinMultiplicity="2" x3="1.256283" y3="-0.764253"
z3="0.452634" />
    <atom id="a2" elementType="O" x3="0.499465" y3="-1.389330" z3="1.410607" />
    <atom id="a3" elementType="H" x3="2.277604" y3="-0.553120" z3="0.720275" />
    <atom id="a4" elementType="H" x3="0.896707" y3="-0.895426" z3="-0.560403" />
    <atom id="a5" elementType="H" x3="0.848091" y3="1.190632" z3="-0.948263" />
    <atom id="a6" elementType="C" x3="-0.841324" y3="-1.213353" z3="1.176247" />
    <atom id="a7" elementType="H" x3="-1.383220" y3="-1.916712" z3="1.827744" />
    <atom id="a8" elementType="O" x3="-1.375406" y3="-0.434288" z3="0.407453" />
    <atom id="a9" elementType="O" x3="1.688998" y3="1.284322" z3="-1.431776" />
    <atom id="a10" elementType="H" x3="2.350386" y3="1.292681" z3="-0.722659" />
  </atomArray>
  <bondArray>
    <bond atomRefs2="a5 a9" order="1" />
    <bond atomRefs2="a10 a9" order="1" />
    <bond atomRefs2="a8 a6" order="2" />
    <bond atomRefs2="a6 a7" order="1" />
    <bond atomRefs2="a6 a2" order="1" />
    <bond atomRefs2="a4 a1" order="1" />
    <bond atomRefs2="a1 a2" order="1" id="bond21" />
    <bond atomRefs2="a1 a3" order="1" />
    <bond atomRefs2="a9 a3" order="1" />
  </bondArray>
  <propertyList>
    <property dictRef="me:ZPE">
      <scalar units="kJ/mol">-93.04081505</scalar>
    </property>
    <property dictRef="me:vibFreqs">
      <array units="cm-1">75.0643 128.9619 183.5067 201.6902 275.1827 335.4973
358.2943 381.5825 393.3452 475.4579 776.2456 1026.6066 1047.0655 1189.1587
1302.8367 1417.93 1488.8347 1608.881 1838.118 3166.118 3231.0885 3397.9734
3829.3828 3990.2888</array>
    </property>
    <property dictRef="me:spinMultiplicity">
      <scalar units="cm-1">2</scalar>
    </property>
    <property dictRef="me:sigma" default="true">
```

```
      <scalar>5.0</scalar>
    </property>
    <property dictRef="me:epsilon" default="true">
      <scalar>50.0</scalar>
    </property>
    <property dictRef="me:frequenciesScaleFactor" default="true">
      <scalar>0.9522</scalar>
    </property>
    <property dictRef="me:symmetryNumber" default="true">
      <scalar>1</scalar>
    </property>
  </propertyList>
  <me:DOSCMethod default="true" name="ClassicalRotors" />
  <me:DistributionCalcMethod default="true" name="Boltzmann" />
  <me:energyTransferModel name="ExponentialDown" default="true" />
  <me:deltaEDown default="NEEDS TO BE CHECKED**">130.0</me:deltaEDown>
</molecule>
<molecule id="COC=O">
  <atomArray>
        <atom id="a1" elementType="C" x3="0.877472" y3="0.038966" z3="-
0.042438"/>
        <atom id="a2" elementType="O" x3="0.891320" y3="1.443191" z3="-
0.347097"/>
        <atom id="a3" elementType="C" x3="0.945703" y3="2.236724" z3="0.728602"/>
        <atom id="a4" elementType="O" x3="0.981478" y3="1.863736" z3="1.870791"/>
        <atom id="a5" elementType="H" x3="0.009502" y3="-0.204764"
z3="0.567169"/>
        <atom id="a6" elementType="H" x3="1.781599" y3="-0.237446"
z3="0.496623"/>
        <atom id="a7" elementType="H" x3="0.830043" y3="-0.466856" z3="-
1.001129"/>
        <atom id="a8" elementType="H" x3="0.952534" y3="3.284372" z3="0.410751"/>
  </atomArray>
  <bondArray>
    <bond atomRefs2="a7 a1" order="1" />
    <bond atomRefs2="a2 a1" order="1" id="bond21" />
    <bond atomRefs2="a2 a3" order="1" id="bond23" />
    <bond atomRefs2="a1 a6" order="1" />
    <bond atomRefs2="a1 a5" order="1" />
    <bond atomRefs2="a8 a3" order="1" />
    <bond atomRefs2="a3 a4" order="2" />
  </bondArray>
  <propertyList>
    <property dictRef="me:ZPE">
      <scalar units="kJ/mol">0</scalar>
    </property>

    <property dictRef="me:spinMultiplicity">
      <scalar units="cm-1">1</scalar>
    </property>
```

<property title="Hessian" dictRef="me:hessian">
<matrix matrixType="squareSymmetricLT" rows="24" units="Hartree/Bohr2">
0.5933028

-0.0006935 0.4557282

0.0018571 0.0471298 0.6201665

-0.0692955 -0.0013666 -0.0001821 0.0805492

-0.0002485 -0.1888469 0.0499776 0.0100384 0.4801626

-0.0007357 0.0204094 -0.0991682 0.013337 0.0778459
0.4002213

0.0032726 -0.0020209 -0.0003494 -0.0638071 -0.0059586
-0.0067281 0.1952726

-0.0024494 -0.0545728 -0.0371731 -0.0052623 -0.2058908
-0.0729582 0.0033486 0.7437009

-0.0001506 -0.0260742 0.0125829 -0.007067 -0.0914766
-0.2090166 0.0241398 -0.1745568 0.9277202

-0.0071596 0.0008221 0.0001568 0.029014 -0.0009379
-0.0053162 -0.0738285 0.005823 -0.0181797 0.0303672

0.0000672 0.0007827 -0.0014251 -0.0009279 0.0323563
-0.0269533 0.0054069 -0.1996979 0.206882 -0.0055669 0.1650505

0.0005204 0.0185747 -0.0021431 -0.0052983 -0.026442
-0.1008311 -0.0180366 0.2174278 -0.6626915 0.0242613 -0.2157468
0.7853275

-0.2287505 -0.042985 0.1210827 0.0061784 -0.0034514
-0.0032833 0.0006027 0.0005695 0.0033337 0.0001822 0.0015677
-0.0009976 0.2392623

-0.0352297 -0.0576405 0.0236615 -0.030321 -0.0265857
0.0198232 -0.0008768 0.0021221 0.0084182 -0.0003393 0.0010609
-0.0033696 0.0475702 0.0726507

0.1215631 0.0289797 -0.1348387 0.0027522 0.0074663
-0.0023338 -0.0007192 -0.0021385 0.0001449 -0.0000903 0.0003144

0.0002189 -0.1330678 -0.0365754 0.1400221

-0.2440808 0.0518392 -0.1113254 0.0074291 0.0027917
0.0032306 0.0004273 -0.000676 -0.0030507 0.0002322 -0.001519
0.0008617 -0.0204345 0.0051753 -0.0139732 0.2557459

0.0436886 -0.0608158 0.0247962 0.0305612 -0.027839
0.0172902 0.0015691 0.0020963 0.0084438 0.0001251 0.0011259
-0.0034122 -0.0046326 0.0036062 -0.003905 -0.0568883 0.0764895

-0.1116128 0.0294753 -0.1162949 -0.0032589 0.0073327
-0.0023335 0.0006682 -0.0021083 0.0003428 0.0000661 0.0004494
0.0001521 0.0162022 -0.0048471 0.0096128 0.1223296 -0.0371268
0.1196548
-0.0542639 -0.0054454 -0.0107741 0.000222 -0.0002675
-0.0001942 0.0002114 0.0000081 0.0000589 0.0006278 -0.000035
-0.0000045 0.0033728 0.0126666 0.0231531 0.001158 -0.0130622
-0.0240599 0.0484766
-0.0048832 -0.0969142 -0.1028366 -0.001958 -0.0327126
-0.0341031 0.0001757 -0.0053268 0.0071768 -0.0000201 0.0024943
-0.0009464 0.0013451 0.0049424 0.0056946 -0.0007346 0.0054523
0.0066492 0.006141 0.1232205
-0.0110079 -0.1166255 -0.2767339 0.0005949 0.0087675
0.0110938 -0.0000317 0.0027266 -0.0020782 -0.000031 -0.0021359
0.00098 -0.0030586 -0.0068879 -0.0127633 0.0016893 -0.0059517
-0.0110812 0.011836 0.1197261 0.2902271
0.0069749 -0.0001499 -0.0004656 0.0097098 -0.0019661
-0.00031 -0.0621509 -0.0013614 0.0009155 0.0205647 0.0010068
-0.0013066 -0.0004133 0.0013546 0.0003821 -0.0004771 -0.001361
-0.0003344 0.0001954 -0.000066 0.000009 0.0255966
-0.0002516 0.0022794 -0.0041303 -0.0007638 -0.0306439
-0.0013542 -0.001644 -0.282431 0.061187 0.0000939 -0.0031727
0.0139146 0.0000164 -0.0001562 0.0001638 0.0000118 -0.0001154
0.0001756 -0.0000057 -0.0011559 0.0003808 0.002543 0.3153956

-0.0004336 -0.0018693 -0.0035706 -0.0008777 -0.0334713
0.0023681 0.0010569 0.0687804 -0.0670045 -0.0008672 0.0386153
-0.0210128 -0.0002113 -0.0002228 -0.0000628 0.0002381 -0.0001346
-0.0000528 -0.0000152 -0.0013605 0.0003556 0.00111 -0.0703373
0.0889798
</matrix>
</property>
<property dictRef="me:sigma" default="true">
<scalar>5.0</scalar>
</property>
<property dictRef="me:epsilon" default="true">
<scalar>50.0</scalar>
</property>
<property dictRef="me:frequenciesScaleFactor" default="true">
<scalar>1</scalar>

```
    </property>
    <property dictRef="me:symmetryNumber" default="true">
      <scalar>1</scalar>
    </property>
   </propertyList>
   <me:DOSCMethod xsi:type="me:ClassicalCoupledRotors">
    <me:MCPoints>10000</me:MCPoints>
    <me:RotorArray>
   <me:Rotor bondRef="bond23" periodicity="1">
    <me:HinderedRotorPotential format="numerical" units="kJ/mol" expansionSize="10"
UseSineTerms="yes">
                <me:PotentialPoint  angle="     0     " potential="   0     "/>
                <me:PotentialPoint  angle="     10    " potential="   1.706575      "/>
                <me:PotentialPoint  angle="     20    " potential="   6.695025      "/>
                <me:PotentialPoint  angle="     30    " potential="   14.361485     "/>
                <me:PotentialPoint  angle="     40    " potential="   23.73452      "/>
                <me:PotentialPoint  angle="     50    " potential="   33.422615     "/>
                <me:PotentialPoint  angle="     60    " potential="   42.69063      "/>
                <me:PotentialPoint  angle="     70    " potential="   50.51462      "/>
                <me:PotentialPoint  angle="     80    " potential="   55.896895     "/>
                <me:PotentialPoint  angle="     90    " potential="   58.154825     "/>
                <me:PotentialPoint  angle="     100   " potential="   57.18339      "/>
                <me:PotentialPoint  angle="     110   " potential="   53.638965     "/>
                <me:PotentialPoint  angle="     120   " potential="   48.387965     "/>
                <me:PotentialPoint  angle="     130   " potential="   42.349315     "/>
                <me:PotentialPoint  angle="     140   " potential="   36.415685     "/>
                <me:PotentialPoint  angle="     150   " potential="   31.24345      "/>
                <me:PotentialPoint  angle="     160   " potential="   27.41022      "/>
                <me:PotentialPoint  angle="     170   " potential="   25.231055     "/>
                <me:PotentialPoint  angle="     180   " potential="   24.758465     "/>
                <me:PotentialPoint  angle="     190   " potential="   25.2048       "/>
                <me:PotentialPoint  angle="     200   " potential="   27.383965     "/>
                <me:PotentialPoint  angle="     210   " potential="   31.24345      "/>
                <me:PotentialPoint  angle="     220   " potential="   36.38943      "/>
                <me:PotentialPoint  angle="     230   " potential="   42.32306      "/>
                <me:PotentialPoint  angle="     240   " potential="   48.36171      "/>
                <me:PotentialPoint  angle="     250   " potential="   53.638965     "/>
                <me:PotentialPoint  angle="     260   " potential="   57.18339      "/>
                <me:PotentialPoint  angle="     270   " potential="   58.154825     "/>
                <me:PotentialPoint  angle="     280   " potential="   55.92315      "/>
                <me:PotentialPoint  angle="     290   " potential="   50.540875     "/>
                <me:PotentialPoint  angle="     300   " potential="   42.716885     "/>
                <me:PotentialPoint  angle="     310   " potential="   33.475125     "/>
                <me:PotentialPoint  angle="     320   " potential="   23.760775     "/>
                <me:PotentialPoint  angle="     330   " potential="   14.413995     "/>
                <me:PotentialPoint  angle="     340   " potential="   6.695025      "/>
                <me:PotentialPoint  angle="     350   " potential="   1.706575      "/>
                <me:PotentialPoint  angle="     360   " potential="   0     "/>
    </me:HinderedRotorPotential>
                <me:CalculateInternalRotorInertia phaseDifference ="0.0"/>
```

```
</me:Rotor>
        </me:RotorArray>
</me:DOSCMethod>
<me:ExtraDOSCMethod xsi:type="HinderedRotorQM1D">
<me:bondRef>bond21</me:bondRef>
<me:periodicity>3</me:periodicity>
   <me:HinderedRotorPotential format="numerical" units="kJ/mol" expansionSize="10"
UseSineTerms="yes">
          <me:PotentialPoint angle="  0  " potential="  0  "/>
          <me:PotentialPoint angle="  10  " potential="  0.26255  "/>
          <me:PotentialPoint angle="  20  " potential="  1.47028  "/>
          <me:PotentialPoint angle="  30  " potential="  3.281875  "/>
          <me:PotentialPoint angle="  40  " potential="  5.19849  "/>
          <me:PotentialPoint angle="  50  " potential="  6.695025  "/>
          <me:PotentialPoint angle="  60  " potential="  7.325145  "/>
          <me:PotentialPoint angle="  70  " potential="  6.957575  "/>
          <me:PotentialPoint angle="  80  " potential="  5.67108  "/>
          <me:PotentialPoint angle="  90  " potential="  3.88574  "/>
          <me:PotentialPoint angle="  100  " potential="  2.021635  "/>
          <me:PotentialPoint angle="  110  " potential="  0.603865  "/>
          <me:PotentialPoint angle="  120  " potential="  -0.026255  "/>
          <me:PotentialPoint angle="  130  " potential="  0.31506  "/>
          <me:PotentialPoint angle="  140  " potential="  1.549045  "/>
          <me:PotentialPoint angle="  150  " potential="  3.36064  "/>
          <me:PotentialPoint angle="  160  " potential="  5.277255  "/>
          <me:PotentialPoint angle="  170  " potential="  6.72128  "/>
          <me:PotentialPoint angle="  180  " potential="  7.325145  "/>
          <me:PotentialPoint angle="  190  " potential="  6.905065  "/>
          <me:PotentialPoint angle="  200  " potential="  5.56606  "/>
          <me:PotentialPoint angle="  210  " potential="  3.701955  "/>
          <me:PotentialPoint angle="  220  " potential="  1.864105  "/>
          <me:PotentialPoint angle="  230  " potential="  0.498845  "/>
          <me:PotentialPoint angle="  240  " potential="  -0.026255  "/>
          <me:PotentialPoint angle="  250  " potential="  0.31506  "/>
          <me:PotentialPoint angle="  260  " potential="  1.549045  "/>
          <me:PotentialPoint angle="  270  " potential="  3.36064  "/>
          <me:PotentialPoint angle="  280  " potential="  5.277255  "/>
          <me:PotentialPoint angle="  290  " potential="  6.72128  "/>
          <me:PotentialPoint angle="  300  " potential="  7.325145  "/>
          <me:PotentialPoint angle="  310  " potential="  7.010085  "/>
          <me:PotentialPoint angle="  320  " potential="  5.88112  "/>
          <me:PotentialPoint angle="  330  " potential="  4.14829  "/>
          <me:PotentialPoint angle="  340  " potential="  2.284185  "/>
          <me:PotentialPoint angle="  350  " potential="  0.73514  "/>
          <me:PotentialPoint angle="  360  " potential="  0  "/>
   </me:HinderedRotorPotential>
<me:CalculateInternalRotorInertia phaseDifference="0"/>
</me:ExtraDOSCMethod>

   <me:energyTransferModel name="ExponentialDown" default="true" />
```

<me:deltaEDown default="NEEDS TO BE CHECKED**">130.0</me:deltaEDown>
</molecule>
<molecule spinMultiplicity="2" id="CO[C]=O">
<atomArray>
<atom id="a1" elementType="C" x3="1.182280" y3="-0.163560" z3="0.073040" />
<atom id="a2" elementType="O" x3="2.541860" y3="0.284310" z3="-0.158230" />
<atom id="a3" elementType="C" x3="3.479850" y3="-0.524620" z3="0.279290" />
<atom id="a4" elementType="O" x3="3.387340" y3="-1.569740" z3="0.837720" />
<atom id="a5" elementType="H" x3="1.026150" y3="-1.123260" z3="-0.422220" />
<atom id="a6" elementType="H" x3="0.545510" y3="0.606740" z3="-0.355350" />
<atom id="a7" elementType="H" x3="1.010430" y3="-0.263920" z3="1.146030" />
</atomArray>
<bondArray>
<bond atomRefs2="a5 a1" order="1" />
<bond atomRefs2="a6 a1" order="1" />
<bond atomRefs2="a2 a1" order="1" id="bond21" />
<bond atomRefs2="a2 a3" order="1" id="bond23" />
<bond atomRefs2="a1 a7" order="1" />
<bond atomRefs2="a3 a4" order="2" />
</bondArray>
<propertyList>
<property dictRef="me:ZPE">
<scalar units="kJ/mol">-81.04275808</scalar>
</property>
<property dictRef="me:vibFreqs">
<array units="cm-1">152.2246 288.6369 386.9847 769.352 958.2114 1170.8316
1183.4724 1231.8529 1469.3762 1498.3608 1504.5501 1915.2925 3096.7238 3190.0855
3218.003</array>
</property>
<property dictRef="me:spinMultiplicity">
<scalar units="cm-1">2</scalar>
</property>
<property dictRef="me:hessian">
<matrix matrixType="squareSymmetricLT" rows="21"
units="Hartree/Bohr2">0.45581874 -0.06793469 0.61137775 0.03451753 -0.00466785
0.60535918 -0.15471054 -0.05323493 0.02881507 0.46333366 -0.03479002 -0.09474196
0.01579394 -0.08050171 0.31598038 0.01873345 0.01571970 -0.07206847 0.04410853 -
0.13263391 0.13840941 -0.06980559 0.02526855 -0.01401820 -0.24273927 0.08370617 -
0.04562299 0.40860453 0.02221358 0.00973249 0.00213854 0.10811754 -0.15037702
0.06338723 -0.02303444 0.82003935 -0.01233835 0.00201281 0.01200582 -0.05844051
0.06329219 -0.06682989 0.01369740 -0.41794833 0.26230869 0.00757539 0.00297076 -
0.00161573 0.01646249 0.04541596 -0.02425019 -0.09712499 -0.08669865 0.04613267
0.06901458 -0.01481178 -0.00283319 -0.00285578 0.05543671 -0.07273348 0.04809846 -
0.08636609 -0.67779230 0.35141840 0.03810041 0.75073619 0.00802007 -0.00275209 -
0.00642079 -0.02973059 0.04795694 -0.00917378 0.04611319 0.35148386 -0.20765959 -
0.02021274 -0.39563564 0.22224594 -0.05191417 -0.02006473 -0.01318410 -0.02443803 -
0.03114380 -0.01504400 0.00341916 -0.00862553 0.00464621 0.00097684 0.00342954 -
0.00222748 0.06418450 -0.02802690 -0.26689307 -0.11354907 -0.00996574 -0.00603252 -
0.00110148 0.00263748 -0.00090664 0.00152258 -0.00009221 0.00107094 -0.00090829
0.03674971 0.28359968 -0.01642837 -0.11472841 -0.11357956 0.00198009 0.00861425

0.00523156 -0.00018232 -0.00128623 0.00049791 0.00078367 0.00051030 -0.00014436
0.01496220 0.12256325 0.11245471 -0.13462237 0.11283455 -0.06286691 -0.03281473
0.02164059 -0.01213521 -0.00584338 -0.00330679 0.00171274 0.00174154 0.00056094 -
0.00031759 0.00509118 -0.00291936 0.00251486 0.16093669 0.12457977 -0.20246152
0.08253874 -0.01235227 0.00947431 -0.00508956 -0.00380439 -0.00058574 0.00040010
0.00126410 0.00101775 -0.00024750 0.01534525 -0.01536939 0.01264832 -0.12479426
0.20769904 -0.06936408 0.08251587 -0.09999178 0.00639985 -0.00473533 0.00306262
0.00207923 0.00031527 0.00002022 -0.00063657 -0.00022566 0.00066315 0.01013401 -
0.01118590 0.00748784 0.06981501 -0.08904092 0.09709258 -0.05234145 0.00016050
0.02835233 -0.02509358 -0.00432719 0.03421042 0.00348954 -0.00866571 0.00458984
0.00135416 0.00365027 -0.00164487 0.00268053 0.00161702 -0.00363013 0.00551107 -
0.00023820 -0.01842745 0.06439974 -0.00122995 -0.05418050 0.02060148 -0.00749960 -
0.00156972 0.01161957 0.00159271 -0.00011015 -0.00069775 -0.00096037 0.00053408
0.00010272 0.00430956 0.00453100 -0.02832149 -0.00401566 0.00022556 0.02235668
0.00780331 0.05056973 0.03685975 0.02189996 -0.32530441 0.00686755 0.00171192
0.00136855 -0.00206631 0.00190967 -0.00034316 -0.00020111 -0.00131007 0.00048943
0.00071316 0.00265890 -0.01194811 0.00127710 -0.00120918 -0.00833463 -0.04345014 -
0.02566120 0.34407233</matrix>
</property>
<property dictRef="me:sigma" default="true">
<scalar>5.7</scalar>
</property>
<property dictRef="me:epsilon" default="true">
<scalar>50.0</scalar>
</property>
<property dictRef="me:vibFreqsFromHessian">
<scalar calculated="20220120_074051" units="cm-1">152.224 288.636 386.983
769.349 958.208 1170.83 1183.47 1231.85 1469.37 1498.36 1504.54 1915.29 3096.71
3190.07 3217.99 </scalar>
</property>
<property dictRef="me:frequenciesScaleFactor" default="true">
<scalar>0.9522</scalar>
</property>
<property dictRef="me:symmetryNumber" default="true">
<scalar>1</scalar>
</property>
</propertyList>
<me:DOSCMethod xsi:type="me:ClassicalCoupledRotors">
<me:MCPoints>10000</me:MCPoints>
<me:RotorArray>
<me:Rotor>
<bondRef>bond21</bondRef>
<me:periodicity>3</me:periodicity>
<me:HinderedRotorPotential format="numerical" units="kJ/mol" expansionSize="10"
UseSineTerms="yes">
<me:PotentialPoint angle=" 0 " potential=" 0 "/>
<me:PotentialPoint angle=" 10 " potential=" 0.183785 "/>
<me:PotentialPoint angle=" 20 " potential=" 0.84016 "/>
<me:PotentialPoint angle=" 30 " potential=" 1.73283 "/>
<me:PotentialPoint angle=" 40 " potential=" 2.6255 "/>

<me:PotentialPoint angle=" 50 " potential=" 3.25562 "/>
<me:PotentialPoint angle=" 60 " potential=" 3.544425 "/>
<me:PotentialPoint angle=" 70 " potential=" 3.386895 "/>
<me:PotentialPoint angle=" 80 " potential=" 2.861795 "/>
<me:PotentialPoint angle=" 90 " potential=" 2.021635 "/>
<me:PotentialPoint angle=" 100 " potential=" 1.10271 "/>
<me:PotentialPoint angle=" 110 " potential=" 0.341315 "/>
<me:PotentialPoint angle=" 120 " potential=" 0 "/>
<me:PotentialPoint angle=" 130 " potential=" 0.15753 "/>
<me:PotentialPoint angle=" 140 " potential=" 0.761395 "/>
<me:PotentialPoint angle=" 150 " potential=" 1.654065 "/>
<me:PotentialPoint angle=" 160 " potential=" 2.546735 "/>
<me:PotentialPoint angle=" 170 " potential=" 3.229365 "/>
<me:PotentialPoint angle=" 180 " potential=" 3.544425 "/>
<me:PotentialPoint angle=" 190 " potential=" 3.41315 "/>
<me:PotentialPoint angle=" 200 " potential=" 2.88805 "/>
<me:PotentialPoint angle=" 210 " potential=" 2.04789 "/>
<me:PotentialPoint angle=" 220 " potential=" 1.10271 "/>
<me:PotentialPoint angle=" 230 " potential=" 0.341315 "/>
<me:PotentialPoint angle=" 240 " potential=" 0 "/>
<me:PotentialPoint angle=" 250 " potential=" 0.183785 "/>
<me:PotentialPoint angle=" 260 " potential=" 0.84016 "/>
<me:PotentialPoint angle=" 270 " potential=" 1.759085 "/>
<me:PotentialPoint angle=" 280 " potential=" 2.651755 "/>
<me:PotentialPoint angle=" 290 " potential=" 3.30813 "/>
<me:PotentialPoint angle=" 300 " potential=" 3.544425 "/>
<me:PotentialPoint angle=" 310 " potential=" 3.36064 "/>
<me:PotentialPoint angle=" 320 " potential=" 2.78303 "/>
<me:PotentialPoint angle=" 330 " potential=" 1.94287 "/>
<me:PotentialPoint angle=" 340 " potential=" 1.023945 "/>
<me:PotentialPoint angle=" 350 " potential=" 0.31506 "/>
<me:PotentialPoint angle=" 360 " potential=" 0 "/>
</me:HinderedRotorPotential>

</me:Rotor>
<me:Rotor>
<bondRef>bond23</bondRef>
<me:HinderedRotorPotential format="numerical" units="kJ/mol" expansionSize="10" UseSineTerms="yes">
<me:PotentialPoint angle="0" potential="0.0" />
<me:PotentialPoint angle="5.0" potential="0.07173147649221348" />
<me:PotentialPoint angle="10.0" potential="0.2856398880556986" />
<me:PotentialPoint angle="15.0" potential="0.6403206376818008" />
<me:PotentialPoint angle="20.0" potential="1.1309667080098655" />
<me:PotentialPoint angle="25.0" potential="1.7541874789119045" />
<me:PotentialPoint angle="30.0" potential="2.4915851941325013" />
<me:PotentialPoint angle="35.0" potential="3.3521409180108974" />
<me:PotentialPoint angle="40.0" potential="4.319141944491504" />
<me:PotentialPoint angle="45.0" potential="5.380718576915658" />
<me:PotentialPoint angle="50.0" potential="6.468031420106057" />

<me:PotentialPoint angle="55.0" potential="7.669781129326473" />
<me:PotentialPoint angle="60.0" potential="8.913875930215042" />
<me:PotentialPoint angle="65.0" potential="10.175022597122569" />
<me:PotentialPoint angle="70.0" potential="11.425155393032314" />
<me:PotentialPoint angle="75.0" potential="12.631978156173801" />
<me:PotentialPoint angle="80.0" potential="13.75756393972768" />
<me:PotentialPoint angle="85.0" potential="14.757651190188362" />
<me:PotentialPoint angle="90.0" potential="15.594197767432057" />
<me:PotentialPoint angle="95.0" potential="16.226097026258735" />
<me:PotentialPoint angle="100.0" potential="16.619994657760532" />
<me:PotentialPoint angle="105.0" potential="16.757581524085253" />
<me:PotentialPoint angle="110.0" potential="16.633648359977038" />
<me:PotentialPoint angle="115.0" potential="16.261751002355954" />
<me:PotentialPoint angle="120.0" potential="15.666759884756555" />
<me:PotentialPoint angle="125.0" potential="14.889811969467099" />
<me:PotentialPoint angle="130.0" potential="13.964805808854402" />
<me:PotentialPoint angle="135.0" potential="12.947452989780457" />
<me:PotentialPoint angle="140.0" potential="11.882416647227165" />
<me:PotentialPoint angle="145.0" potential="10.816119077211415" />
<me:PotentialPoint angle="150.0" potential="9.79403357078658" />
<me:PotentialPoint angle="155.0" potential="8.856322916420401" />
<me:PotentialPoint angle="160.0" potential="8.03888230289411" />
<me:PotentialPoint angle="165.0" potential="7.371603348435153" />
<me:PotentialPoint angle="170.0" potential="6.877987520475606" />
<me:PotentialPoint angle="175.0" potential="6.575186243112727" />
<me:PotentialPoint angle="180.0" potential="6.473064282943017" />
<me:PotentialPoint angle="185.0" potential="6.575076507242136" />
<me:PotentialPoint angle="190.0" potential="6.877048331917322" />
<me:PotentialPoint angle="195.0" potential="7.370137134759554" />
<me:PotentialPoint angle="200.0" potential="8.03645434060963" />
<me:PotentialPoint angle="205.0" potential="8.852984203038037" />
<me:PotentialPoint angle="210.0" potential="9.78972074084821" />
<me:PotentialPoint angle="215.0" potential="10.811202428546698" />
<me:PotentialPoint angle="220.0" potential="11.876964193019441" />
<me:PotentialPoint angle="225.0" potential="12.942387621301554" />
<me:PotentialPoint angle="230.0" potential="13.958825461063823" />
<me:PotentialPoint angle="235.0" potential="14.879379529235086" />
<me:PotentialPoint angle="240.0" potential="15.657376123824962" />
<me:PotentialPoint angle="245.0" potential="16.250573376873653" />
<me:PotentialPoint angle="250.0" potential="16.62235895690832" />
<me:PotentialPoint angle="255.0" potential="16.746616893623827" />
<me:PotentialPoint angle="260.0" potential="16.61104867043126" />
<me:PotentialPoint angle="265.0" potential="16.219432338701722" />
<me:PotentialPoint angle="270.0" potential="15.590480351087372" />
<me:PotentialPoint angle="275.0" potential="14.7564458197637" />
<me:PotentialPoint angle="280.0" potential="13.759356442768816" />
<me:PotentialPoint angle="285.0" potential="12.63677881803423" />
<me:PotentialPoint angle="290.0" potential="11.433993432845488" />
<me:PotentialPoint angle="295.0" potential="10.187285621706808" />
<me:PotentialPoint angle="300.0" potential="8.927740331989801" />

```
      <me:PotentialPoint angle="305.0" potential="7.684509742938388" />
      <me:PotentialPoint angle="310.0" potential="6.482987021084489" />
      <me:PotentialPoint angle="315.0" potential="5.343796316212372" />
      <me:PotentialPoint angle="320.0" potential="4.284956327749349" />
      <me:PotentialPoint angle="325.0" potential="3.3219013892620737" />
      <me:PotentialPoint angle="330.0" potential="2.4623584116715764" />
      <me:PotentialPoint angle="335.0" potential="1.722695507287231" />
      <me:PotentialPoint angle="340.0" potential="1.1094717700729602" />
      <me:PotentialPoint angle="345.0" potential="0.6234445614879266" />
      <me:PotentialPoint angle="350.0" potential="0.27827661991756686" />
      <me:PotentialPoint angle="355.0" potential="0.07447232203133601" />
      <me:PotentialPoint angle="360.0" potential="0.013578978372427853" />
     </me:HinderedRotorPotential>

    </me:Rotor>
     </me:RotorArray>

</me:DOSCMethod>
    <me:DOSCMethod default="true" name="ClassicalRotors" />
    <me:DistributionCalcMethod default="true" name="Boltzmann" />
    <me:energyTransferModel xsi:type="me:ExponentialDown">
     <me:deltaEDown derivedFrom="[CH2]OC=O:deltaEDown">150</me:deltaEDown>
     <me:deltaEDownTExponent referenceTemperature="298"
derivedFrom="[CH2]OC=O:deltaEDownTExponent">1</me:deltaEDownTExponent>
    </me:energyTransferModel>
  </molecule>
  <molecule spinMultiplicity="2" id="CH3_pre_comp">
    <atomArray>
     <atom id="a1" elementType="C" x3="1.590476" y3="0.324268" z3="-0.398125" />
     <atom id="a2" elementType="O" x3="1.410924" y3="-1.102541" z3="-0.446415" />
     <atom id="a3" elementType="H" x3="2.651470" y3="0.551026" z3="-0.473258" />
     <atom id="a4" elementType="H" x3="1.286478" y3="0.716993" z3="0.583627" />
     <atom id="a5" elementType="H" x3="0.966721" y3="0.839521" z3="-1.134263" />
     <atom id="a6" elementType="C" x3="1.223931" y3="-1.740118" z3="-1.611418" />
     <atom id="a7" elementType="H" x3="0.710042" y3="-2.699430" z3="-1.431129" />
     <atom id="a8" elementType="O" x3="1.601589" y3="-1.376703" z3="-2.726314" />
     <atom id="a9" elementType="O" spinMultiplicity="2" x3="-0.546744" y3="-0.822373"
z3="1.475768" />
     <atom id="a10" elementType="H" x3="0.309404" y3="-0.980909" z3="1.105418" />
    </atomArray>
    <bondArray>
     <bond atomRefs2="a9 a10" order="1" />
     <bond atomRefs2="a8 a6" order="2" />
     <bond atomRefs2="a5 a1" order="1" />
     <bond atomRefs2="a6 a7" order="1" />
     <bond atomRefs2="a6 a2" order="1" id="bond62" />
     <bond atomRefs2="a1 a2" order="1" id="bond12" />
     <bond atomRefs2="a1 a4" order="1" />
     <bond atomRefs2="a1 a3" order="1" />
     <bond atomRefs2="a10 a6" order="1" />
```

</bondArray>
<propertyList>
<property dictRef="me:ZPE">
<scalar units="kJ/mol">-18.06518522</scalar>
</property>
<property dictRef="me:vibFreqs">
<array units="cm-1">64.8471 124.4542 185.3756 266.3419 333.6176 354.0543
440.0652 474.4843 792.8493 979.9042 1061.5637 1185.5472 1211.7951 1311.2414
1412.1397 1483.9081 1509.5684 1522.5054 1837.7354 3093.2838 3158.5779 3176.41
3221.8885 3674.7478</array>
</property>
<property dictRef="me:spinMultiplicity">
<scalar units="cm-1">2</scalar>
</property>
<!-- <property dictRef="me:hessian">
<matrix matrixType="squareSymmetricLT" rows="30"
units="Hartree/Bohr2">0.46193612 -0.00458265 0.63343569 0.01746490 -0.02001300 0.6
1382130 -0.21224985 -0.02002659 0.02843148 0.48592817 0.00333246 -0.08 157215
0.00736564 -0.11786483 0.37075525 0.01345048 0.00987636 -0.0771 2588 0.03184344 -
0.17030054 0.18736714 -0.06469054 0.05538575 -0.03558 889 -0.02689449 0.03520547 -
0.02014761 0.08686364 0.06787744 -0.245424 21 0.11962411 -0.00007782 0.00214057 -
0.00146972 -0.07251363 0.2554933 9 -0.04393106 0.12124155 -0.12815501 0.00291572 -
0.00523643 0.00299072 0.04380169 -0.12948519 0.12832052 -0.08504572 -0.07860867 -
0.03837679 -0.03342710 -0.02514471 -0.01538377 0.00576478 0.00755294 0.00516114
0.10521488 -0.08764590 -0.24637473 -0.09437171 -0.00060010 0.00719791 0.00443062 -
0.00889026 -0.01749893 -0.01196313 0.09477181 0.25769943 - 0.04247034 -0.09375577 -
0.09779954 0.00221621 0.00655575 0.00300577 0. 00756914 0.01439580 0.00821199
0.04421718 0.09992385 0.09764191 -0.061 14580 0.00414303 0.05174740 -0.02781988
0.00735728 0.03532447 0.003942 41 -0.00225941 -0.00906434 0.00536162 0.00033331 -
0.01186564 0.0776478 2 0.00456527 -0.05345676 -0.01817446 0.00105888 -0.00091588
0.00013236 -0.00675816 0.00437527 0.02550437 0.00646669 -0.00052113 -0.02629757 -
0.00493844 0.05109707 0.06139053 -0.01971797 -0.31385631 0.00546123 0 .00004080
0.00513262 0.00230467 -0.00297172 -0.01189964 0.00195841 0.0 0085685 -0.01030181 -
0.06922021 0.01994773 0.33153414 -0.04017864 0.03 912887 -0.02039329 -0.19953507
0.08807557 -0.04216283 -0.00678858 -0.0 0141932 0.00170393 0.00054103 0.00211069 -
0.00121819 0.00085499 0.0017 4452 -0.00133517 0.84032180 0.03419205 -0.00482239
0.00135638 0.095167 71 -0.22134566 0.08786039 -0.00455290 0.00044518 0.00020648 -
0.0085091 5 0.00095362 -0.00236700 -0.00649026 0.00270312 0.00238942 0.12656730
0.71612208 -0.01716643 0.00056553 0.00376650 -0.04660888 0.08862958 -0 .11773936
0.00353989 0.00000957 -0.00011925 0.00376294 0.00076805 0.00 174826 0.00572251 -
0.00265998 -0.00014533 -0.14720304 -0.34094706 0.43 372290 0.00341777 0.00243929 -
0.00117646 -0.02375266 0.00636547 -0.000 41698 -0.00086590 -0.00003574 0.00014295 -
0.00003438 -0.00017283 0.000 09475 -0.00027543 -0.00008539 0.00001742 -0.31335501 -
0.00998021 0.031 57000 0.34611014 0.00035389 -0.00081556 0.00508891 0.03413827 -
0.00187 264 0.00394599 0.00127847 0.00010593 -0.00007522 0.00088577 -0.0004227 6
0.00006975 -0.00053515 0.00001783 -0.00039797 -0.01728962 -0.0538747 1 -0.00447730
0.01336879 0.05801645 0.00024158 0.00490698 0.00409113 - 0.01830961 0.00687256
0.00769866 -0.00067683 0.00007955 0.00023713 0.0 0092716 -0.00066920 -0.00020959 -
0.00125043 0.00037761 -0.00044241 0.0 3630616 -0.00535713 -0.06436850 -0.04114642 -
0.02139299 0.04444085 -0. 00408175 0.00276450 -0.00242485 0.04094270 -0.00071724 -

0.00068306 0.0 0287019 0.00103244 -0.00082345 0.00191197 0.00030040 0.00178292 0.0017 1821 -0.00190205 -0.00101189 -0.28144594 -0.22025607 0.16243137 -0.011 52598 - 0.03266488 0.02418945 0.26130975 -0.01775243 -0.00036852 -0.002 13835 0.00919141 - 0.07516270 0.06603006 0.00060331 0.00035651 0.000073 62 0.00275215 -0.00027888 0.00048013 0.00276883 -0.00060357 -0.0005248 4 -0.23776935 -0.43762949 0.25624852 - 0.01213036 -0.00153635 0.0151743 0 0.25177018 0.51474321 0.01090286 -0.00440973 - 0.00511576 -0.00694569 0.06707506 -0.01170146 -0.00063180 0.00004393 0.00062009 - 0.00216287 0.00008183 -0.00045024 -0.00172015 0.00077960 0.00038629 0.17352650 0. 25440013 -0.25536087 0.01105383 0.01727478 0.00809228 -0.18497456 -0.3 3408365 0.26447712 -0.00108022 0.00007183 0.00001062 0.00171786 -0.002 44147 0.00140075 0.00009052 -0.00014238 0.00011170 -0.00034906 -0.0005 0025 0.00034772 -0.00039253 - 0.00060735 0.00040093 -0.00149173 0.00604 678 -0.00368482 -0.00029828 -0.00069279 0.00041563 -0.00744546 -0.0064 2034 0.00532586 0.47603743 0.00046188 -0.00080362 0.00003471 -0.001718 45 0.00087276 -0.00011433 -0.00002040 -0.00009513 0.00026110 - 0.000270 02 -0.00090764 0.00038168 -0.00054988 -0.00123216 0.00085404 0.0007776 3 - 0.00165561 0.00078709 -0.00014260 -0.00004835 -0.00016725 -0.001338 32 0.00219722 0.00039945 -0.07426668 0.01962857 -0.00020911 0.00048230 -0.00006733 0.00095738 - 0.00012955 0.00011571 0.00002072 0.00017720 - 0.00003430 0.00027448 0.00046010 - 0.00070883 0.00023517 0.00055337 -0. 00089619 -0.00030926 0.00020722 -0.00025653 0.00010147 -0.00007534 -0. 00025889 0.00204408 0.00093107 0.00146824 -0.01423810 - 0.00032222 0.00 529041 0.00311861 -0.00071536 0.00030587 -0.00490966 0.00583200 - 0.003 22490 -0.00029202 -0.00001451 -0.00001827 0.00006198 0.00029312 -0.000 67374 0.00010858 0.00045604 0.00003409 0.00107713 -0.01218525 0.007636 48 0.00057973 0.00115726 -0.00069669 -0.00425369 0.00698661 -0.0043739 9 -0.46678853 0.07706685 0.01112316 0.47129787 -0.00080201 0.00020225 0.00122777 0.00073152 -0.00009746 - 0.00039121 0.00026235 0.00010142 -0 .00052715 0.00010320 0.00015312 0.00061337 0.00017068 -0.00146378 -0.0 0047636 -0.00192628 -0.00089613 0.00107601 0.00037359 0.00043017 0.000 17557 0.00101104 -0.00171742 -0.00156139 0.07895265 -0.01795603 - 0.002 28415 -0.07887676 0.02124386 0.00032658 0.00082373 0.00044089 0.000038 71 - 0.00087288 0.00025608 -0.00019098 -0.00040354 -0.00017225 -0.00037 788 0.00048274 - 0.00113792 0.00009123 -0.00016304 0.00048863 0.0010852 0 0.00225117 -0.00124780 - 0.00024056 0.00003937 0.00071934 -0.00053001 -0.00219086 -0.00241571 0.00990971 - 0.00211426 -0.00465230 -0.0101120 0 0.00214755 0.00772102</matrix>
</property>-->
<property dictRef="me:sigma" default="true">
<scalar>5.0</scalar>
</property>
<property dictRef="me:epsilon" default="true">
<scalar>50.0</scalar>
</property>
<property dictRef="me:frequenciesScaleFactor" default="true">
<scalar>0.9522</scalar>
</property>
<property dictRef="me:symmetryNumber" default="true">
<scalar>1</scalar>
</property>
</propertyList>

<me:DOSCMethod default="true" name="ClassicalRotors" />
<me:DistributionCalcMethod default="true" name="Boltzmann" />

<me:energyTransferModel name="ExponentialDown" default="true" />
<me:deltaEDown default="NEEDS TO BE CHECKED**">130.0</me:deltaEDown>
</molecule>
<molecule spinMultiplicity="2" id="[CH2]OC(=O)OO">
<atomArray>
<atom id="a1" elementType="C" spinMultiplicity="2" x3="1.020109" y3="-0.225215" z3="0.003287" />
<atom id="a2" elementType="O" x3="0.526137" y3="-0.823270" z3="1.162658" />
<atom id="a3" elementType="H" x3="2.083080" y3="-0.044428" z3="0.076128" />
<atom id="a4" elementType="H" x3="0.526366" y3="-0.526115" z3="-0.909724" />
<atom id="a5" elementType="H" x3="-2.591408" y3="-2.205774" z3="3.376903" />
<atom id="a6" elementType="C" x3="-0.843162" y3="-0.902137" z3="1.273116" />
<atom id="a7" elementType="O" x3="-1.657749" y3="-0.437739" z3="0.484172" />
<atom id="a8" elementType="O" x3="-1.130939" y3="-1.597771" z3="2.423247" />
<atom id="a9" elementType="O" x3="-2.587236" y3="-1.694163" z3="2.548872" />
</atomArray>
<bondArray>
<bond atomRefs2="a5 a9" order="1" />
<bond atomRefs2="a7 a6" order="2" />
<bond atomRefs2="a4 a1" order="1" />
<bond atomRefs2="a9 a8" order="1" id="bond98" />
<bond atomRefs2="a6 a8" order="1" id="bond68" />
<bond atomRefs2="a6 a2" order="1" id="bond62" />
<bond atomRefs2="a1 a2" order="1" id="bond12" />
<bond atomRefs2="a1 a3" order="1" />
</bondArray>
<propertyList>
<property dictRef="me:ZPE">
<scalar units="kJ/mol">-331.3735674</scalar>
</property>
<property dictRef="me:vibFreqs">
<array units="cm-1">136.5564 169.9987 270.6821 273.9378 342.9493 415.221 482.5241 535.0077 728.2992 774.5499 993.083 1070.8474 1198.2706 1260.4344 1401.6551 1464.2378 1522.053 1870.9306 3217.7808 3376.6121 3630.8574</array>
</property>
<property dictRef="me:spinMultiplicity">
<scalar units="cm-1">2</scalar>
</property>
</propertyList>
</molecule>
<molecule spinMultiplicity="2" id="TS_COC=O_[CH2]OC=O">

<atomArray>
<atom id="a1" elementType="C" x3="0.597690" y3="-0.685798" z3="0.562960"/>
<atom id="a2" elementType="O" x3="-0.780795" y3="-0.883852" z3="0.327634"/>
<atom id="a3" elementType="H" x3="1.048996" y3="-1.659111" z3="0.720984"/>
<atom id="a4" elementType="H" x3="0.780963" y3="0.028647" z3="1.362136"/>
<atom id="a5" elementType="H" x3="1.110756" y3="-0.215478" z3="-0.426833"/>
<atom id="a6" elementType="C" x3="-1.483718" y3="0.235530" z3="0.093239"/>

<atom id="a7" elementType="H" x3="-2.537537" y3="-0.016642" z3="-0.051554"/>
<atom id="a8" elementType="O" x3="-1.024827" y3="1.346139" z3="0.045706"/>
<atom id="a9" elementType="O" x3="1.445327" y3="0.561747" z3="-1.431960"/>
<atom id="a10" elementType="H" x3="0.843145" y3="1.288829" z3="-1.202312"/>
</atomArray>
<bondArray>
<bond atomRefs2="a9 a10" order="1" />
<bond atomRefs2="a5 a1" order="1" />
<bond atomRefs2="a7 a6" order="1" />
<bond atomRefs2="a8 a6" order="2" />
<bond atomRefs2="a6 a2" order="1" id="bond62" />
<bond atomRefs2="a2 a1" order="1" id="bond21" />
<bond atomRefs2="a1 a3" order="1" />
<bond atomRefs2="a1 a4" order="1" />
<bond atomRefs2="a5 a1" order="1" />
<bond atomRefs2="a9 a5" order="1" id="bond95" />
</bondArray>
<propertyList>
<property dictRef="me:ZPE">
<scalar units="kJ/mol">2.58</scalar>
</property>
<property dictRef="me:imFreqsScale">
<scalar units="cm-1">1</scalar>
</property>
<property dictRef="me:spinMultiplicity">
<scalar units="cm-1">2</scalar>
</property>
<property dictRef="me:hessian">
<matrix matrixType="squareSymmetricLT" rows="30" units="Hartree/Bohr2">
0.4197694

-0.0867276 0.5920187

0.0863311 0.0901621 0.3920104

-0.1979248 -0.028448 -0.0540393 0.4510669

-0.0242532 -0.1020505 -0.0023897 -0.0658806 0.3967468

-0.0259406 -0.0098691 -0.0650763 0.0823186 -0.0397774 0.0952188

-0.082949 0.0918626 -0.0188538 -0.0287194 0.0357029
-0.0087658 0.1078369

0.1098608 -0.2900073 0.0322235 -0.0064606 0.0101487
-0.001401 -0.1111071 0.2985569

-0.025864 0.0344314 -0.0461972 -0.0020284 0.0048581
-0.0008219 0.0238961 -0.0375963 0.0459781

-0.0511 -0.0132565 -0.0245791 -0.0228805 -0.0211521
-0.0320349 0.0043306 0.0061825 0.0100186 0.0656826

-0.0180254 -0.1659814 -0.1397506 -0.0086446 0.0021357
-0.0022402 -0.0038143 -0.0115818 -0.0205306 0.0293389 0.1744379

-0.0385594 -0.1454125 -0.1930706 -0.002304 -0.000201
0.0013505 0.0014414 0.0060655 0.0067758 0.0370062 0.1500512
0.2037561

-0.018232 0.0094687 -0.0104671 -0.0377672 -0.0066937
0.0191581 0.0024269 0.0035119 -0.003193 0.0007093 -0.0026176
0.0024961 0.0474215

0.0367431 -0.0117637 -0.0371305 -0.0270599 0.0034397
0.0031499 -0.0101725 -0.0075679 0.0234525 0.0023452 -0.001668
-0.0053609 -0.0279019 0.0128458

-0.0354515 -0.0183734 -0.0094862 0.0299473 -0.0085219
0.0037294 0.005717 0.0003711 -0.0119762 0.0102987 0.0147312
-0.0240773 0.0216734 0.0513102 -0.0173037

-0.0561523 0.0375249 -0.0067154 -0.1793886 0.0646016
-0.027625 -0.0052036 -0.0026864 -0.0017498 0.0019483 0.0025125
-0.0007871 0.0029662 0.0072923 -0.0086895 0.7634133

0.0469326 0.0188431 -0.0104213 0.0643034 -0.2039121
0.0275373 -0.0075198 -0.0012553 0.0004606 -0.0091116 -0.0020635
-0.001967 -0.0130379 -0.0137143 0.0217493 0.2057644 0.8748803

-0.0136985 0.0053319 0.0025783 -0.0279998 0.0249468
-0.071026 -0.0004905 -0.000297 0.0005639 0.0003294 0.0018062
0.0002266 0.0026408 0.0011354 -0.001688 0.0708588 -0.0430046

0.209176					
	0.0031197	0.0037146	-0.0011649	-0.0259563	0.0027556
-0.0063571	-0.0010276	-0.0003294	-0.0001105	-0.0003049	-0.0001608
0.0001245	-0.0001461	-0.0004437	0.0004377	-0.2914772	-0.0490122
-0.0320481	0.3212714				
	0.000852	-0.0042636	0.0017692	0.0355092	-0.0003039
0.0071998	0.001439	0.0003009	0.0001447	0.0003253	-0.0000321
0.0001051	0.0002922	0.0003321	-0.0007771	-0.0564201	-0.0599517
-0.0097257	0.0572877	0.0812072			
	-0.0008863	0.0018156	0.0061835	-0.0093359	0.0014977
0.0081499	-0.0003528	-0.0001433	0.0000655	0.0011497	-0.000496
-0.000228	-0.0008896	0.0000638	-0.0002998	-0.0319225	-0.0079267
-0.0660372	0.0420778	0.0031351	0.0320046		
	-0.0034884	-0.0007316	0.003371	0.038273	0.0130293
-0.0005678	0.0027639	0.0017251	0.0000582	0.0014188	0.0003674
0.0015885	0.0024312	0.0018259	-0.0045016	-0.2349083	-0.2360802
0.0000984	-0.0056488	-0.0393363	0.0002146	0.202458	
	-0.0250364	-0.0051081	0.0024542	0.0232609	-0.1039506
0.0170498	0.0014084	0.0007827	-0.0005242	0.0035034	0.0007346
0.0004273	0.0049049	0.0057553	-0.00793	-0.2541225	-0.6193786
0.0200129	-0.0139549	-0.0171849	0.0016233	0.2581432	0.7415112
	0.0053653	0.000906	-0.0083527	-0.0031454	0.0179493
0.0260752	0.000228	0.0003786	0.0008924	-0.0005462	-0.0002271
0.0007847	-0.0009367	-0.0018008	0.0025103	0.0025838	0.0200228
-0.0743737	-0.0026424	-0.0018295	0.0201639	-0.002999	-0.0346476
0.0350223					
	-0.0199148	-0.0141565	0.0300586	0.0095358	-0.0021554
0.0005262	0.0012873	-0.0006908	-0.0019449	0.0005682	0.0014392
-0.0012328	0.0001651	0.0241836	-0.0264334	-0.0010512	0.0080898
-0.0015896	0.0002293	-0.0004205	-0.0004339	-0.0043448	-0.0031825
0.0045174	0.2071654				
	-0.0366679	-0.0288939	0.0587036	0.0106199	-0.0002275
-0.0011698	0.0018497	0.0002143	-0.0037758	0.001307	0.0033042
-0.0029299	0.0225948	0.0188321	-0.0465437	-0.0016571	0.0034383
-0.0009382	0.0001696	0.000016	0.0000493	-0.0004024	0.000072
0.0006346	-0.2291306	0.2859422			
	0.0517563	0.0394583	-0.0806089	-0.0151059	0.0032753
0.0016035	-0.0031178	0.0009483	0.0053613	-0.0016929	-0.0036524
0.0052284	-0.0250612	-0.0489245	0.0601517	0.0038445	-0.0105633
0.0008711	-0.0000808	0.0005897	0.0002862	0.0043494	0.0039537
-0.0024657	-0.0858499	0.0844341	0.0545398		
	0.0068723	0.0007492	-0.0039411	-0.0062389	0.0040457
-0.0007116	-0.0007459	-0.000006	0.0009177	-0.0003725	-0.0003954
0.0002264	0.0000249	-0.0068122	0.007002	-0.0001466	-0.0103287

0.0018992 -0.0000595 0.0004715 0.0003788 0.0010453 0.0050754
-0.0024247 -0.1936403 0.2313169 0.0709582 0.1932612
-0.003678 -0.0027933 0.0043796 0.0028001 -0.0020264
-0.0004794 0.000351 0.0004087 -0.0009205 0.0005178 0.0007145
-0.0007779 0.0094785 -0.006491 -0.0060157 -0.0028097 0.0031139
0.0007322 -0.0000264 -0.00012 0.0003812 0.0014597 -0.0032337
-0.0013863 0.2160236 -0.2826977 -0.0695193 -0.2241164 0.2931249

-0.0030524 0.0015495 0.0020196 0.0016928 -0.0016373
0.0007968 0.0002982 -0.0005492 -0.0006417 0.0000505 0.0003082
-0.0007462 -0.0054208 0.0141049 -0.0015602 0.0002021 0.0041129
-0.0002909 -0.0002362 -0.0006114 -0.0002887 -0.0016117 -0.0024195
-0.0002567 0.0823823 -0.0884642 -0.0449675 -0.0743049 0.0736061
0.0459354
</matrix>
</property>
<property dictRef="me:frequenciesScaleFactor" default="true">
<scalar>1</scalar>
</property>
<property dictRef="me:symmetryNumber" default="true">
<scalar>0.5</scalar>
</property>
</propertyList>
<me:DOSCMethod xsi:type="me:ClassicalCoupledRotors">
<me:MCPoints>500000</me:MCPoints>
<me:RotorArray>
<me:Rotor bondRef="bond95" periodicity="1">
</me:Rotor>
<me:Rotor bondRef="bond62" periodicity="1">
</me:Rotor>
<me:Rotor bondRef="bond21" periodicity="1">
</me:Rotor>
</me:RotorArray>
<me:ExpansionSize>
<property dictRef="size">
<array >8 8 8</array>
</property>
</me:ExpansionSize>
<me:FourierSeries>
<property dictRef="coeficients">
<array units="kJ/mol">4367.6483 -170.4008 415.8602 150.1547
31.4194 -1.9608 -0.5141 0.5584 -712.2584 173.8014
46.9947 37.6333 -1.231 1.3165 -2.1328 -3.9722 -1810.4881
60.6648 23.4284 -79.1161 -0.9725 2.5907 -5.551 -4.58 -
88.7203 20.2553 5.6682 67.0719 -1.6031 -2.6561 1.5029
0.8313 62.0351 -2.1516 -2.1742 36.5347 -0.9289 -
1.0204 -0.9691 -1.4382 4.0769 -2.6132 -0.7738 9.4065 3.1929 -
0.7318 -4.4137 -3.5574 -4.5053 3.6823 0.4373 -1.6923 0.0361 -
1.6528 -0.6341 1.5404 0.661 4.068 -0.9395 -0.0816 -0.4677 -
1.8317 -0.5596 2.8197 -148.5689 42.749 -28.8604 -2.6823 -2.1544

1.0526 0.5012 1.0839 -113.8073 -309.4083 -164.2658 -54.6338 -
5.8122 2.2667 2.0806 2.0204 6.5617 -37.2589 -62.7357 -32.2351 -5.353
1.415 1.0085 1.3547 -1.7396 -1.0413 -5.4271 -7.6981
5.4479 1.0881 4.181 3.9894 0.9559 7.0013 4.694 7.4067 6.5783 5.0829 1.9875
0.6733 0.6917 1.4951 0.6791 0.8844 -1.5501 0.4389 1.6392 0.975 0.5565 -
1.4908 -0.3492 2.9257 -1.1225 2.5779 1.4933 -2.2767 0.648 -0.5959
0.6558 2.8701 -0.4541 1.9252 0.9223 -2.3309 -205.4786 28.6926
-36.8571 -1.1307 -0.8241 1.8677 -0.335 -2.585 -16.0004 -
57.4044 -64.9238 -37.3529 -10.6508 -0.0415 0.2955 0.5753
5.3871 6.7957 -31.3308 -32.9362 -11.1544 -0.0011 3.6715
5.008 -4.5528 -5.3319 -5.8877 -13.0233 -8.8068 -
1.0707 -1.3745 -2.8125 1.5344 2.2 3.4593 2.3738 -0.5831 0.9425 -
0.154 1.463 4.1801 3.6993 4.4354 -1.6048 -3.3491 -1.0677 0.1696
5.873 -2.7569 -4.2486 -1.4268 1.3589 1.0838 1.4306 -0.1792
-1.941 -2.9074 -3.0264 0.5694 -1.6185 3.0563 -0.3858
0.3138 -2.2659 -109.5376 31.3133 -25.7173 -1.0551
0.1031 -0.6093 -1.8035 -0.2588 -15.5035 -22.9536 -
3.0211 1.3915 -1.935 1.2808 0.5962 0.2489 -0.7702 2.913 -2.1709 -7.3789
-2.371 3.8087 0.8445 -3.0695 1.4949 3.2128 4.1805 -4.283 -9.6494 -
4.7864 -3.5761 -4.1461 -2.1422 -2.2431 0.0554 -0.9416 -
3.0298 -1.7724 -2.7359 -0.871 2.3209 2.6346 2.6754 2.7168 4.0715 0.0527 -
1.8927 -0.2172 -1.4916 -0.4503 -2.5599 -3.816 -0.5413 -
0.7058 0.1847 1.7265 0.0735 -0.9213 -2.5568 -4.5912 0.1283 -2.3256
-0.4495 1.8623 19.4213 -1.8216 -2.8207 -6.8832
1.4242 -2.1006 -0.4232 3.4701 0.4421 -7.2261 -5.2467 -
3.3589 2.259 -3.0544 -0.8506 3.9496 0.7773 -3.8293 -5.2551 -
4.4669 0.485 0.2906 -2.7168 -3.3533 0.5316 3.0307 -2.7707 -6.1855
-5.5103 -5.4251 -0.4404 2.1614 -0.9049 2.159 -0.6022
-1.4142 2.4899 -2.0688 0.5644 -0.2648 -2.1261 -2.002 -
0.0514 4.1668 3.9833 3.0313 -1.9318 -4.6934 2.3862 2.3576 1.1501 -1.6919
-3.5545 -0.5923 -2.6466 0.2009 3.4413 -0.7706 -1.2878
0.6231 -6.6316 1.3384 -0.4708 -1.6028 21.047 -19.8189
7.7099 -1.7736 1.7711 3.4331 1.8458 -1.1624 16.6075 14.9987
1.762 -1.4256 -9.6404 -3.4629 0.7763 -1.1056 7.167
0.9753 -5.8074 -3.9371 -9.8359 -2.7114 -1.3417 -
1.2444 -1.1719 -5.3759 -3.1803 2.2448 -0.7209 3.8868 2.1536 -
1.6043 1.0113 3.6915 1.2147 2.6501 2.3672 -0.8979 1.765 -2.3353 -1.9474
-1.1646 -2.1883 -3.9205 -1.6061 -2.334 -0.2164
1.5471 -1.3435 2.6842 2.6239 0.8686 4.2919 -2.0955 -2.2236
2.4151 -1.1678 1.3269 2.8136 -0.1536 3.7723 0.4003 0.5453 0.6472
13.5365 8.0014 6.0038 1.6015 3.4153 1.0222 -0.9215 -0.8738
0.6638 4.8932 1.4848 -3.319 -3.0121 -3.5518 0.8519 0.1516 -3.5604
-0.3099 -5.3814 -4.7541 -5.1381 -6.185 0.6982 4.6779 -
0.0588 2.6979 8.4143 7.9447 5.5621 2.9435 -0.7517 -2.1191 -0.9846 -
1.7223 -0.6169 0.3542 -2.4508 -2.2886 1.1902 -0.9296 1.5999
0.5598 -3.8633 -6.667 -6.8825 -4.9697 0.586 3.2836 1.0894
0.6651 -0.4965 4.8388 3.1705 4.6517 4.8613 -1.7386 -0.0556 -
1.6347 1.6606 3.1178 2.7584 4.2531 2.3795 -1.8227 24.9691 4.5428 -1.5276
0.6704 -2.2983 -1.3678 -0.421 1.2285 -3.8463 -8.0367 -
2.7055 -0.8007 2.2047 2.8867 0.2138 -0.8059 -3.5032 -3.6759

0.5286 4.3033 3.2717 -1.4325 -0.473 0.3602 0.7644 2.3063 3.1315 -0.3391
-1.1158 -3.9346 -2.451 2.0486 -0.4754 -3.9014 -0.4549
0.2791 -0.1072 1.0168 -0.571 2.4679 -0.3994 -1.882 -0.4578
3.0337 3.344 2.1824 -0.0766 -3.7236 2.1215 0.9569 -2.4075 -
0.2835 -3.0812 -1.1135 0.5313 -1.7411 0.5721 1.8121 -1.12 -1.4811
-2.5368 -0.3791 -0.6298 0.9783</array>
</property>
</me:FourierSeries>
<me:FourierSeries>
<property dictRef="coeficients">
<array units="kJ/mol">0 30.0061 -13.9028 -12.5297 -13.1318
-1.5388 1.5366 2.2739 0 -29.2063 -16.7414 -15.028 -
5.9505 -0.6593 -1.5665 0.7254 0 -15.1062 -16.7019 -16.0947
-13.3636 -4.9186 -0.2762 3.4209 0 -3.2148 -0.9341
-4.1588 -1.9648 -2.0868 -3.286 -1.2149 0 -0.5842
-0.6494 -3.6718 -0.5194 -0.5668 -0.1427 0.5 0
-2.216 -5.4216 -3.0975 -6.1091 -3.151 -0.1894 -0.2096
0 2.1949 -0.1315 1.1303 -0.7866 -1.6385 0.7106 -1.4379
0 2.957 0.8269 2.2046 0.8439 -0.6015 2.1237 -0.0671 0 -
2.8515 -0.82 5.944 6.078 1.3542 0.6496 -1.4953 0 37.0075 46.663
26.5069 3.7949 -4.8379 0.4292 -2.4434 0 4.5897 15.0264
14.8425 2.0997 -7.3275 -0.7766 -4.8068 0 2.6497
3.7846 4.6947 -0.2865 -3.3867 0.9767 1.5605 0 0.6713 0.7542
1.5551 -0.7058 -2.0313 -1.0455 -0.8094 0 0.6942
1.7948 -0.412 0.3671 2.6703 0.1141 0.3819 0 -1.183 2.1231 0.793 0.5958
1.3465 1.1529 -0.0034 0 -2.3069 1.7796 -2.0095 1.5459
2.6089 -2.4888 0.6834 0 -24.5641 1.8937 -0.7126 3.4552
2.9421 0.0091 -0.7649 0 -0.8858 8.7904 15.5656 9.114
3.3776 -0.1872 0.3439 0 2.3861 12.6964 18.9921 13.6316
0.768 -4.1156 -2.7852 0 -2.0148 -3.214 2.83 5.8488
6.5531 4.7039 2.2717 0 -1.5476 -3.1176 0.3812 -0.5385
0.4866 -0.2136 -0.2491 0 6.6636 6.7676 3.8894 3.0432 0.4317 -
1.5379 -1.1272 0 -0.9802 -2.7058 -3.0607 -0.6731
1.508 5.1008 0.3393 0 -1.1728 -2.4524 -2.5242 0.0736
1.8361 2.0863 2.2911 0 -13.6146 0.148 -3.6821 -2.6001 -
3.6683 -4.0213 0.0738 0 -6.2692 -9.1309 -6.9947 -2.5442
2.9336 0.7872 4.3246 0 -3.8062 -3.9184 2.2497 8.5708 9.8717
4.7846 5.3601 0 -0.9018 -2.2129 -3.583 1.3168 1.1706 -1.6762
-2.3154 0 1.5529 -1.2297 -2.2588 -0.3999 0.1538 -
1.1283 0.5437 0 -0.1039 0.1312 1.4304 -0.0315 1.9509 3.6718 4.1198 0
2.0435 -2.8054 -0.5312 -2.3779 -1.6244 -3.2253 -
3.7481 0 1.6095 -2.0084 1.3873 -1.3978 -3.8322 0.0341 -4.0344
0 10.551 0.9176 2.9942 -0.9315 -3.5258 -0.0044 -1.7087
0 2.7841 -6.8125 -2.1137 -1.7487 -1.4702 4.9777
0.7921 0 3.3491 -4.6203 -2.3263 -1.744 1.7014 4.5516 0.5734 0
5.091 2.5756 3.5018 0.8763 -3.2794 -3.1018 -5.4996 0 -
0.184 -0.3087 -0.767 2.4619 -1.429 -0.5183 0.635 0 -6.3219 -
4.7335 -2.7093 -2.1272 4.4882 3.5499 2.2155 0 0.0157 1.8068 2.3179 -
1.4173 -0.3667 -7.205 -0.3305 0 -0.7232 3.9361 1.3127 -0.3084
-0.1641 -5.7233 -2.3715 0 5.5964 6.3633 4.3793 1.4812

3.0077 0.7423 3.3101 0 -0.0483 3.0282 -0.0024 -0.823 1.4001 -
3.192 -1.5938 0 1.3182 7.1398 -0.7579 -1.7743 0.181 -9.4088
-5.0027 0 -1.424 0.268 1.1469 3.1193 7.2537 4.7073 2.6454 0 -
1.8419 1.3052 0.9996 0.9912 -0.4824 0.6017 0.3954 0 0.3571 -1.7526 -
0.4317 -1.8133 -3.3719 -3.0155 -7.1578 0 -0.3494 -
2.0607 3.9479 3.3258 2.0239 4.0141 1.9669 0 -0.4861 -4.2288 1.4547
1.7485 0.9051 4.4931 0.9526 0 -2.8039 0.8708 4.5513 1.6839 1.8119
3.7794 3.3047 0 10.0945 7.6645 7.0248 -0.6603 -2.5123
0.3452 -4.7932 0 3.4895 2.3348 3.8529 1.787 -3.4785 -2.2774
-5.226 0 -1.7425 -7.0095 -2.0663 -0.1811 4.0893
7.0169 3.6817 0 1.0232 0.2921 -0.9772 0.1046 0.2763 1.7956 1.1086 0
3.6437 4.7102 1.4008 -0.4321 -6.5631 -6.3374 -4.1964 0
-3.4465 1.9977 -1.3842 2.4244 4.4974 3.527 2.5808 0 -3.0713
0.364 -2.281 0.375 2.2738 2.358 4.4719 0 6.9429 3.3678 1.4138 -2.0353
-1.8422 -1.808 -1.1282 0 3.091 4.1046 0.2993 0.1985 0.6596
1.039 0.7185 0 -0.5196 -0.759 1.7989 0.8024 -0.3794 4.1574
1.405 0 1.8057 1.0272 0.4335 -0.1633 -4.7272 -4.871 -2.9224
0 2.2237 0.3921 0.013 1.5898 0.6759 -0.1594 0.0611 0 -0.5759
-0.9206 -0.8773 1.2392 4.9726 3.4605 5.1709 0 -0.4017
2.914 -1.2883 -2.6414 -3.4634 -2.6391 -1.2294 0
1.6951 1.1388 0.5283 -1.1752 -2.8106 -1.2247 -2.5121</array>
</property>
</me:FourierSeries>
<me:FourierSeries>
<property dictRef="coeficients">
<array units="kJ/mol">0 0 0 0 0 0 0 0
0.6825 -15.299 -8.6941 -7.9424 -4.9318 -1.1596
1.8179 -0.6229 -1.5334 -4.8661 -8.1782 -11.1418 -
16.3286 -6.9855 0.0451 -1.2881 -1.3842 -5.4498 -2.1342
-3.0241 -3.3629 -2.2608 -1.7966 -2.7255 1.4692 -
0.1359 -0.2241 -1.6123 -4.8458 -3.0689 -0.3408 0.3956
0.7088 -3.359 -0.9673 -1.6623 -4.8073 -2.212 -1.2216 -
2.0875 -0.8137 -5.3951 -1.6809 1.0776 -1.0052 0.2376 -0.6841
-1.7607 2.135 2.6677 0.4393 0.1085 -1.1839 1.7453 3.213 3.0205 0
0 0 0 0 0 0 0 -3.3402 31.8496
60.0429 34.0531 5.2335 -3.4498 -0.192 1.0012 -2.425 2.6086
16.5706 11.9599 5.4681 -5.2349 -2.7221 -0.4554 -
1.5289 0.6902 4.2086 -1.0453 1.7516 -4.7744 -2.2091 3.307 -0.7329
4.4225 3.696 0.7704 3.3997 -3.6901 -0.1882 2.0916 -1.0532
1.0475 1.2579 -2.1347 4.1815 0.6778 1.4089 3.8202 -1.0667 1.341 -
1.055 -1.3267 1.3954 -1.9813 -0.8103 2.5255 -0.4391 2.1496
1.1905 -1.1862 2.2659 -1.3359 -2.3326 0.5466 0 0 0
0 0 0 0 0 -2.2398 -0.9488 7.9215 12.6247
8.4586 5.5987 -1.0154 -3.2734 2.5511 2.1958 2.1303 8.1755 9.5127
4.875 2.3597 2.8229 3.8798 2.0878 0.4259 4.3501 4.7506 5.9913 2.5702 1.6253 -
1.838 -4.7603 0.3052 4.3801 3.7243 2.3456 -1.1013 -6.5356 0.0042
1.183 0.4416 1.2624 0.6302 1.903 0.8108 2.0677 1.9328 6.3783 2.3569 0.8689 -
2.0666 -0.8578 2.1234 2.2357 -2.1557 -4.6691 -1.1419 1.3104
3.004 1.7756 -4.307 -4.7523 0 0 0 0 0 0 0
0 6.1095 -1.8284 -14.8676 -13.6539 -9.6411 -3.3995

0.7321 1.6906 3.5553 1.1651 -3.1031 0.4154 5.4143 4.9219 3.1677 1.2611
3.3284 -0.1235 -1.8238 4.2855 2.2869 6.861 0.8737 -2.3572
0.232 -4.9436 -6.451 -6.6311 -4.8702 0.6817 -0.5533 -
1.6895 0.8837 -0.6955 -0.1967 2.7583 -2.3528 1.0981 0.1152 -3.6074
0.4817 0.0059 1.7295 2.8756 1.7517 0.7904 -1.2208 -1.8051 -0.9109
0.2534 -3.9811 -0.9137 -2.1015 -0.4604 1.0452 -0.5118
0 0 0 0 0 0 0 0 4.7868 3.0778 -4.2975
-5.0503 -1.5223 -4.6273 -0.8047 2.9015 -1.2818
1.3682 0.7968 2.0828 6.7861 1.9776 -1.2551 -1.317 -2.8538 0.9703 -
2.0118 -0.435 6.0571 -0.0294 1.0174 -0.4775 0.2729 4.6011 -0.8964 -
9.2831 -0.6247 -2.8908 -0.5457 8.0433 -0.822 1.9483 0.1711 -1.6832
3.3522 -3.2987 0.5612 0.7523 -1.9615 -1.9287 1.3607 0.2633
6.6234 -0.1383 -2.2751 0.7698 -0.4957 4.4618 -3.8241 -
3.7762 -1.0793 -4.642 2.9597 4.5118 0 0 0 0 0 0 0
0 -0.8502 -1.6912 4.3379 7.0946 5.1053 7.7725 1.2158 0.4719
0.8257 -0.42 -1.4752 3.4891 -2.4675 -0.7425 -4.1367 -
2.8041 0.0386 -1.3115 -1.5159 0.3485 -3.5867 0.2968 1.356 -4.1215
2.5632 -0.5131 3.5243 7.0515 3.0552 7.2044 3.5203 -3.1345 1.7177 -
2.1448 -1.7311 0.4314 -3.584 1.2641 1.627 -1.6974 1.3783 -1.4348 -
1.303 0.2443 -5.4749 0.5172 0.6794 -0.0012 0.8242 -1.9073 1.2092
4.7725 0.6689 2.1574 0.6973 -4.1824 0 0 0 0 0 0
0 0 -4.6005 3.2466 11.9809 9.827 5.8413 4.7057 0.8194 -
2.5966 0.125 3.3692 1.1332 -0.3809 -1.8261 -3.8547 -1.1172
3.5017 0.7022 2.6336 -1.1719 -6.366 -2.2676 -4.1174 -1.9764
-0.8279 0.5735 0.8791 0.9993 3.2996 7.1437 3.8103 1.2687 -5.1966 -
0.5598 0.9846 -1.6322 -2.016 1.8439 -0.2182 1.4967 2.2691 -1.965 1.7072 -
3.5451 -5.8359 -4.5231 -4.3443 -1.5128 2.643 0.522 1.0311
5.3877 3.7679 5.0549 3.8741 0.271 -3.1713 0 0 0 0 0
0 0 0 -3.1397 3.4403 2.9805 -2.402 -2.8528 -6.2825
0.7938 -1.7736 -1.8772 -0.1796 3.394 1.2885 1.2581 0.5436
0.3883 -0.0981 0.429 -0.2801 1.2701 0.7324 2.049 1.3584 0.2014 -
0.3401 1.1121 2.881 -1.488 -3.3737 -3.2389 -2.583 -0.6172 0.6944 -
0.6847 -0.2212 -0.0858 0.543 0.3247 0.28 -0.0482 -0.0142 -
2.0201 -1.4387 1.2019 -0.1202 3.6136 1.0092 -1.0813 0.5966 0.6814
3.0725 1.1406 -2.6071 -2.0112 -0.8762 0.1297 3.3464</array>
</property>
</me:FourierSeries>
<me:FourierSeries>
<property dictRef="coeficients">
<array units="kJ/mol">0 0 0 0 0 0 0 0 0
-114.018 -39.8795 -56.5899 1.2702 1.9842 0.6002 -0.6656 0
-21.6241 -27.7607 90.6094 -9.4656 0.9116 4.6962 1.1794 0
-12.4914 8.5425 -60.2859 2.3466 2.1009 1.2356 1.6993 0 -0.8941
7.9144 -38.7021 1.0007 -0.3686 -1.0584 1.5383 0 -1.4994
-1.9695 -11.8158 -2.9019 2.2293 0.3093 2.8101 0 -3.0899
-1.9957 -1.9208 -0.7902 1.9654 0.4956 1.4381 0 1.5684
4.071 1.3091 2.3561 1.1449 -0.1615 1.0764 0 0 0 0 0
0 0 0 0 285.111 189.5079 66.7684 7.1921 -
6.0718 0.4065 0.2934 0 42.7623 66.8048 29.7017 6.2312 -4.4202
-0.9714 -2.5284 0 -0.2287 1.361 7.7603 -2.5941 -

5.0608 -0.901 -4.6478 0 1.4938 -4.946 -3.1204 -3.4992 -3.855
2.7094 -0.5694 0 0.422 -4.1528 -1.0024 -2.2604 -
2.7016 0.4062 -4.7937 0 1.8068 -3.7035 1.5521 -1.7658 -4.8876
0.9399 -2.5472 0 1.6283 -0.5583 3.6993 -0.0563 0.0576
3.036 0.4717 0 0 0 0 0 0 0 0 0
57.2218 65.2914 33.5276 10.4387 -1.9216 2.1815
2.215 0 8.6727 39.1852 30.2329 11.11 0.3018 -3.4802 -
0.6093 0 -0.432 2.2295 15.1467 7.8246 2.4772 -3.2178 0.9506 0 -
1.6493 -6.709 -2.4688 -2.6349 2.6475 -1.6738 0.3821 0 0.0018
4.1437 -0.1835 1.926 0.5581 -1.393 -2.5279 0 2.3226 5.0918
2.186 2.1758 -0.6953 -1.82 -0.2122 0 -3.943 -4.7266 -
3.7341 -3.6198 2.3467 0.9939 -0.2459 0 0 0 0 0 0
0 0 0 27.7293 15.5354 7.1996 -2.7434 -4.7076
-6.885 -4.1174 0 -3.8064 9.4424 7.3402 4.6855 3.6581 1.0439
5.0123 0 -6.2694 -3.7626 3.962 7.288 6.6606 1.4367 4.6346 0
-3.0513 -1.9814 -4.425 -1.8941 0.742 -5.381 -1.4979 0
-0.5331 0.9686 -1.1515 1.8719 2.7408 2.7561 6.1652 0 -1.4329
2.5501 0.2997 1.7927 1.3555 1.3424 3.7572 0 -1.0303 0.1284 -2.7759
-0.9104 -2.9543 -3.2502 -4.7777 0 0 0 0
0 0 0 0 0 10.6242 11.3595 8.8022 -0.9095
-1.8596 -1.4945 -4.5775 0 -0.5303 1.7698 6.5397
3.1923 1.8341 3.8723 2.4233 0 -1.8097 -3.5251 3.16 5.7018
2.9744 6.6921 -0.7654 0 4.8709 1.2233 3.8285 0.8331 -6.2557 -
0.0302 -4.3607 0 3.0013 -7.6983 1.4656 -0.344 -3.4926 5.4987
1.4091 0 -1.0561 -5.4392 -1.2541 0.3233 2.1731 6.9523
1.7442 0 4.0939 4.4939 2.3989 2.9613 -5.9076 -1.8241 -1.6551
0 0 0 0 0 0 0 0 0 -14.8976 -
4.9065 -4.9195 2.0878 7.808 5.3115 6.5017 0 -1.9744 6.108 3.6169
2.0629 2.2469 -7.0719 -1.0163 0 -1.1685 3.906 -0.6948
2.0406 0.8742 -6.0854 -1.8903 0 -1.3719 2.5878 1.7663
1.5662 5.9445 0.6154 4.0724 0 -0.9755 2.396 -2.3555 -0.7053
-1.4237 -4.2612 1.0676 0 -1.6808 3.8301 -2.5782 -
3.2172 3.1376 -5.9182 0.0406 0 -2.157 2.2319 -1.7783 2.8491 6.4634
0.4016 6.3068 0 0 0 0 0 0 0 0 0 -
2.5432 -2.2089 0.4765 6.3936 6.7092 5.9176 3.578 0 0.3142 1.1698 1.6837 -
0.3213 -2.6389 -3.6291 -4.5667 0 1.987 -4.0235 0.7754
0.634 -4.8991 -1.1692 -1.5786 0 -1.8451 -7.3168
0.2364 1.1227 7.293 8.5655 2.3101 0 2.4458 1.0064 2.5751 1.044 -2.3941
0.5559 -2.7425 0 3.8109 2.3236 4.8229 -2.6537 -5.2474 -
7.2615 -6.2964 0 -0.6736 -3.5588 -1.5549 -1.5818
4.2023 3.5037 3.6465 0 0 0 0 0 0 0 0 0
5.0251 2.3166 5.348 0.4528 -4.5508 -3.1049 -2.6412 0 -
1.8375 -1.6116 -1.7799 1.2853 1.6153 4.947 0.3361 0 -0.1651 -
0.2394 -1.2869 -0.5936 -1.0237 3.3502 1.7253 0 1.6099 2.5228 -
1.8299 -1.5355 -3.87 -2.4001 -2.6594 0 0.463 -1.1731
0.8043 2.055 1.44 2.1892 0.507 0 0.2564 -3.8855 0.3044 0.4615
0.7824 3.4685 2.068 0 2.0799 0.3409 1.791 -2.3719 -3.7521 -
2.4476 -3.7763</array>
</property>
</me:FourierSeries>

<me:FourierSeries>
<property dictRef="coeficients">
<array units="kJ/mol">0 0 0 0 0 0 0 0 0
0 0 0 0 0 0 0 0 0 0 0 0
0 0 0 0 0 0 0 0 0 0 0 0
0 0 0 0 0 0 0 0 0 0 0 0
0 0 0 0 0 0 0 0 0 0 0 0
0 0 0 0 0 0 0 -309.0869 2.084 -25.1152
27.791 5.4395 3.3942 0.1118 -2.8798 -30.9446 21.7769 76.9296
45.5836 4.7866 -5.4097 -3.5208 1.3308 -12.9289 10.0578
32.3652 25.0774 8.054 -1.7386 1.2143 3.7679 -1.986 -2.0409
10.4635 13.9375 3.4649 1.3499 -1.8036 -3.175 3.0612 3.9472
4.1998 1.4329 -3.733 -3.0749 -2.4876 0.7384 1.1245 -3.0326 -
4.059 -6.429 -4.5174 -2.551 2.575 5.6347 -1.4706 -0.4129 3.9658
1.5309 6.9579 1.6971 0.7617 -0.0511 -1.346 -0.2404 4.0295 1.2834
5.315 1.7648 1.6604 -1.9029 -137.2342 25.4455 -12.5655
8.9714 4.1606 -1.4547 0.2942 3.1768 -28.3097 -14.8238 31.7671
23.014 10.4207 -1.5065 -1.0628 2.89 -14.6837 -9.7664
12.1371 18.002 17.5917 1.3141 -3.3447 -0.5679 2.559
4.4918 6.3276 11.0232 10.7852 3.0459 0.078 0.5923 -0.1181 -
1.685 0.9033 -1.3727 0.5187 -2.071 -0.4373 0.031 -0.0751 -2.2269
-2.5784 -1.5894 -1.4917 0.5096 -0.3518 -0.2211
2.7073 0.6712 1.9482 -1.044 0.5207 3.7784 2.3536 0.9307 2.7862 -1.0494
1.242 1.9422 -4.1989 2.3662 -0.2837 -1.6135 86.4696 -
4.7581 6.5292 -7.5023 -7.7177 -2.9177 -0.363 0.427 8.8526 -13.401
-14.3891 -2.7826 -2.7118 2.7714 1.2566 -1.5732 5.776 -
11.6708 -8.3223 5.3493 3.9147 7.2343 -2.2726 -6.1387 4.7747
0.9568 -6.3165 -1.8497 0.0287 1.7394 0.9573 0.389 -0.2609 -
0.0123 -1.2003 -2.863 1.9283 -0.2065 2.366 -2.1216 -2.8645
0.8893 0.2155 3.5057 4.9197 1.0454 -2.7651 -5.4429 0.572 3.296 -
0.6375 -5.4213 -1.3871 -5.9247 -2.0615 2.4777 -0.6343
2.855 -2.2433 -3.3045 -1.2136 -0.7271 -2.049 3.1735
68.7369 -23.9792 10.2757 -7.5478 -1.4858 -0.131
0.3081 -1.2628 20.9369 20.8495 -5.6755 -8.7316 -
3.4631 -1.6314 1.4482 2.3707 10.5098 7.4205 -4.2834 -1.6982 -
1.4123 -2.0978 -0.7759 2.4265 -4.4319 -9.4223 -8.7455 -
6.1794 -1.7871 -1.2628 -1.4038 -0.8747 0.2619 3.281 0.99
1.1586 2.652 -0.1101 1.5849 -2.4143 0.8243 3.5577 3.8195 3.9939
1.0491 2.0496 4.8411 2.6075 -1.2478 -3.8074 -1.8758 -0.0496
-0.8328 -4.305 -3.2939 -3.1831 -1.6612 -2.7001 -
1.6686 0.2462 -1.967 0.1869 -0.7402 -2.8291 11.7393 4.1972 4.756
2.43 1.4063 0.3237 -0.5453 -1.834 0.0086 9.3829 2.0663 -4.5126 -
1.4687 -0.4986 -0.0461 0.5677 -0.603 7.075 1.1259 -2.5827 -1.9305
-5.2946 1.7646 5.5762 -3.0524 -1.0381 4.2617 2.3778 2.7579
0.5862 -2.5197 -2.9414 -1.3582 -1.664 -0.2016 -0.7087
-3.0524 0.6214 1.13 -1.4065 4.1349 3.1157 -0.4278 -0.8336
-3.8577 -1.9852 6.222 5.3132 -1.2565 -3.823 -2.2257
3.4596 2.2688 2.1299 2.6818 -2.9625 -0.9335 -2.2008 -0.4393
1.6371 0.6021 0.6488 0.6887 -2.4353 17.5258 14.3719 -0.9673
4.6726 -3.5036 1.2188 1.2849 -0.0815 -8.7714 -18.6666 -

4.9561 0.9524 -0.2705 5.8168 -1.376 -4.5583 -4.9126 -6.857 -0.7038
2.3101 -1.1392 1.2829 -0.0509 -3.6759 3.8435 7.3265 6.7963
4.0086 0.7027 0.5268 0.8556 0.7416 -0.6244 -3.977 -0.1316 -1.083 -
1.4678 2.5784 -0.7702 1.2574 -1.4934 -3.6407 -4.0686 -1.1982
-0.5441 -2.5742 -2.0316 -3.695 1.5836 3.8565 0.4632 -0.1242
2.12 0.1626 2.0456 1.7134 0.1589 5.3847 0.8548 -1.8505 3.4846 -1.4775
0.0966 4.3235 -0.9596 -5.544 -0.5682 -0.2671 0.023 0.5389
2.1153 3.4506 -0.2933 -3.0441 -5.0422 -4.0977 4.7822 -
0.2553 -2.3058 2.9131 0.8666 0.6824 1.6004 0.2444 5.5396 1.2425 -3.0578 -
2.7978 0.2016 3.3482 -2.9479 -5.2176 2.3459 -1.5245 2.0208 5.7752
2.2117 1.5653 1.8925 -0.3414 2.5458 2.3892 -0.968 1.9285 -4.1466 -
7.2913 -0.855 1.9391 0.2318 1.7111 -3.8868 -6.5995 2.5029 1.0383 3.0427
1.79 -4.7566 -1.0512 -1.9624 0.3262 2.5973 2.5911 1.7828
2.2072 -3.724 0.7659 -0.5608 0.1111</array>
</property>
</me:FourierSeries>
<me:FourierSeries>
<property dictRef="coeficients">
<array units="kJ/mol">0 0 0 0 0 0 0 0 0
0 0 0 0 0 0 0 0 0 0 0 0
0 0 0 0 0 0 0 0 0 0 0 0
0 0 0 0 0 0 0 0 0 0 0 0
0 0 0 0 0 0 0 0 0 0 0 0
0 0 0 0 0 0 0 0 -14.2121 13.2131
-7.947 -5.8656 4.5552 2.4919 2.4829 0 238.0269 148.1248
39.5092 -0.9227 -2.5329 -2.2707 1.3529 0 50.2203
67.6029 32.5329 1.1639 -3.6986 -7.2787 -4.0903 0
4.9799 12.0553 4.9392 2.4543 7.1163 7.0848 6.4166 0 0.5538 1.0666 -
1.0691 -3.9691 -1.7832 -0.0733 -0.0828 0 1.5155 2.4048 -
1.2253 -2.438 -7.6781 -6.8095 -9.1866 0 -0.6721 -4.5365
-1.5009 1.6255 1.8815 4.2727 1.6829 0 -2.1912 -2.4311 -
1.0606 0.5845 2.0896 5.8613 3.6343 0 -15.5067 26.0732 18.8242
4.9416 -2.6011 0.9607 -0.5133 0 101.2743 95.8187
46.4086 9.251 -0.699 2.8332 -2.6126 0 18.9412 46.445
39.6289 17.79 4.7768 2.5445 -1.4957 0 2.3618 8.5218 17.9852
9.7759 2.9351 3.2794 0.9671 0 0.3713 -1.9064 -1.6392 -1.535 -
2.0001 -0.1581 -0.7373 0 -3.4506 -1.3579 -0.9737 -
2.2533 -0.3774 -1.9095 -1.7021 0 -0.8694 2.0031 -0.2821
-0.8236 2.9793 -2.7215 3.076 0 -2.2024 3.9365 0.4132
0.0043 2.1424 -1.4534 2.3636 0 13.048 13.2638 5.5238 -2.7396
-2.8799 -2.8899 -0.0667 0 10.1652 20.2057
7.7635 3.6064 4.4414 -1.7925 2.2548 0 -6.7393 10.5589
12.0832 8.2292 7.3191 -1.3818 4.9644 0 -1.2118 4.6959
9.4575 4.8796 -0.3135 -5.4041 -5.2766 0 0.3168 -0.3944
-1.5064 2.9331 1.7868 -0.4231 1.0173 0 -3.3559 -8.2333
-1.3826 0.059 4.0504 7.4114 4.2446 0 1.1761 -0.0835 3.7334 -
3.6635 -2.8148 -3.4281 -3.6139 0 3.9734 -1.9675 4.4214 -
1.7903 -5.3809 -2.5754 -5.4628 0 5.1314 3.3719 5.2245 -1.8768
-1.6259 -0.3403 -1.3191 0 2.5694 6.8467 8.1161 3.9581 -
0.54 2.571 -0.5628 0 -2.1667 5.56 8.723 6.8373 1.7875 -1.6024

-1.9436 0 -2.1028 -5.1395 3.6108 2.2605 1.2066 1.2244 -
2.5635 0 0.2745 -0.3785 1.2843 -0.1677 0.6611 0.5495 -0.6972 0
1.9701 1.4709 1.4849 4.0003 2.1248 1.6451 1.1816 0 -2.3957 0.5203 -
0.8873 0.032 -0.1084 -0.6641 -1.194 0 -2.5199 -2.0084 -
2.7814 -0.2999 0.4059 -2.7432 0.2128 0 -6.8502 -2.3735
0.6775 -1.1912 0.5555 2.0909 0.481 0 5.1583 6.3131 6.0113 3.0302 -
2.1602 0.629 -2.8696 0 1.2292 2.4247 5.3875 4.3131 -1.9313 -0.3038
-1.9577 0 -3.8643 -8.0496 -2.7576 -0.2867
2.4258 3.5642 0.0361 0 1.3431 -1.4154 0.5803 -1.5815 1.4998
1.524 -0.7222 0 4.4536 7.5149 3.5326 2.2692 -3.7524 -2.4592
-1.6373 0 -2.5428 0.4759 -4.5097 2.4644 2.214 2.5853
0.4597 0 -2.3322 0.7306 -3.9576 -1.1014 1.9331 1.3605
2.9949 0 -1.3192 1.0017 -3.1157 -1.8765 1.3154 -0.6672
2.1898 0 -4.0959 4.0028 -4.4931 0.2333 1.8766 -4.3306
1.4464 0 -4.6616 -0.6802 -2.2428 -0.6349 -0.0797
-0.4932 1.2491 0 -0.9497 4.5753 0.8467 2.077 -0.09 -2.7846
1.9781 0 1.3716 0.5135 -0.6741 0.3485 2.3068 -0.1849 0.2668 0
-0.7827 -1.3365 -2.1437 -1.6525 -1.0061 1.9371
0.7292 0 3.1759 -0.5913 -0.6934 -0.8527 -3.0145
2.1308 -0.3679 0 5.2701 0.2496 3.3706 0.7822 -2.2136 3.8342 -
2.6624 0 5.8895 -3.5438 -1.053 -0.8159 -0.3812 0.6822 -1.7603
0 -10.1045 -12.5652 -3.9824 0.1003 -3.293 1.5064 -0.6074
0 -4.2161 -7.7645 -2.8901 -2.726 -3.3407 2.1197 -
2.2228 0 2.6458 6.9009 7.0535 5.4739 -0.3834 1.0855 0.2824 0 -0.248
0.1681 1.7222 -0.8153 1.0778 -0.6457 -0.0935 0 -3.8058
-3.8849 -6.3046 -2.581 2.5035 -0.3088 0.6304 0 1.0461
3.05 1.9206 -0.4128 -0.1244 -1.0363 2.5899 0 1.574
4.5386 0.0747 2.7271 3.8481 -3.2852 1.1269</array>
</property>
</me:FourierSeries>
<me:FourierSeries>
<property dictRef="coeficients">
<array units="kJ/mol">0 0 0 0 0 0 0 0 0
0 0 0 0 0 0 0 0 0 0 0 0
0 0 0 0 0 0 0 0 0 0 0 0
0 0 0 0 0 0 0 0 0 0 0 0
0 0 0 0 0 0 0 0 0 0 0 0
0 0 0 0 0 0 0 0 0 0 0 0
0 0 0 -1.2423 179.6237 139.9817 51.4544
11.3788 3.0079 -1.7012 0.0401 -1.092 33.3519 56.5308
27.0322 -5.7792 -1.9766 -0.4575 1.8232 4.6303 5.2522
12.7124 4.3766 -7.1095 -0.6487 0.3735 -1.4307 1.6458 -
1.1013 6.0294 5.2885 3.1602 7.5148 1.7888 -3.5876 0.2167 -1.73 -4.0217 -
1.6041 -6.5729 -1.9645 -0.4903 -0.8116 -0.1012 0.8072 -
3.2519 -2.5122 -7.6142 -4.0502 -2.252 -2.41 -0.4441 -1.1794
3.0999 6.0569 5.1089 4.4894 -1.2552 -4.4311 0 0 0 0
0 0 0 0 -5.2338 73.6428 103.9539 54.9487
15.1974 0.017 0.931 4.2877 -7.2702 9.9598 53.8235 39.5106
19.1311 3.2991 -1.1337 2.1361 -0.674 1.3819 6.0665 12.8913
10.7561 1.5552 -1.4567 0.2512 1.3037 5.1806 1.8179 -3.0866

4.2322 -0.9205 -0.6175 4.088 -1.6072 2.2812 -0.6965 -
1.9845 3.5525 -1.9317 0.4874 2.0041 -2.5442 -0.5605 -3.4003 -
5.8633 1.8694 -2.5661 -3.3094 0.9664 0.3729 6.4786 2.2127 0.3453 3.2573 -
0.3664 3.0087 2.3545 0 0 0 0 0 0 0 0 -4.3357
9.2071 21.6511 11.9538 0.7822 -4.1036 -2.2529 0.2406 -
3.3473 -0.7432 18.9109 25.8418 10.4088 6.2162 0.2142 -4.7096
1.3529 -2.6632 -1.6578 10.4864 7.986 6.8149 0.9229 -4.4115
2.8719 -1.9896 -5.5646 -5.6966 -7.9427 0.778 0.5312
2.8514 2.3203 -2.3892 -0.645 2.2641 -1.427 2.8206 1.2771 -2.8347
0.733 -4.5761 2.8115 4.1213 2.2267 4.8601 -1.832 -2.5302 0.5507
0.9491 -4.4819 -0.9794 -5.5534 -3.1154 3.6997 0.8926 0
0 0 0 0 0 0 0 -9.3941 -6.5556
5.131 5.4231 4.7129 0.4972 -1.5963 0.3005 0.3785 2.9794 6.6936 11.1868
7.094 0.0396 -0.9577 -0.7654 0.2454 2.7565 1.0282 1.0507 8.1961
1.2362 2.4519 1.0372 -3.4188 -2.2517 -3.4369 -2.0115
1.8467 -0.4907 -1.3171 -0.9738 -0.1174 2.5208 0.3997 -
1.513 2.2088 1.2624 1.5696 1.1606 1.8454 5.9051 4.059 2.6616 1.3622 2.8447 2.8626
4.5658 -3.3875 -6.0376 -3.634 -3.3376 -2.0237 -3.3401
-2.4243 -2.7152 0 0 0 0 0 0 0 0
-5.7146 -3.0962 5.1742 6.0956 3.9077 -0.3286 -1.4679 -
2.9044 0.6278 2.6607 1.513 -0.5403 -0.2392 -1.7768 0.4571 4.0117
1.1281 3.2506 0.9488 -3.0392 1.0563 -1.5874 1.6047 0.6932 -3.1368
-2.6448 -0.591 1.6879 4.2986 1.0593 -1.8455 -4.349 -0.9535
3.6062 -1.1448 -1.9327 1.449 0.5713 0.964 2.7312 0.4654 5.8601
0.2475 -2.1215 -2.4935 -2.6454 1.1084 3.151 -1.1921 -
2.0463 3.418 -0.0131 2.6711 1.1981 -3.8131 -3.5572 0 0 0
0 0 0 0 0 2.3412 5.1658 3.804 2.2657 -2.2389 -
4.2103 1.7353 -0.6129 -2.1082 -7.1388 -1.2411 -0.4827 -
4.0555 0.5949 0.9941 -1.6375 1.0155 -5.3314 -1.4379 2.9264 -1.2114
1.8011 -0.3615 -2.8093 4.7474 3.556 2.9392 1.6886 -5.653 1.0506
0.3984 0.2782 0.4328 -3.0859 -1.3151 3.0998 -3.5228 1.4128 -
0.0052 -3.563 -1.9534 -7.1269 -2.6266 -0.4337 -2.4709 -
0.3721 -1.7934 -4.3924 3.2802 3.962 3.8449 1.9091 -0.0378 2.4142
1.7866 3.2866 0 0 0 0 0 0 0 0 3.7284
1.6014 -5.1606 -5.1502 0.0733 -2.935 2.0649 6.0684 -3.9101 -
6.0423 -6.8166 -5.3573 0.744 -0.583 -0.2289 -1.2468 -4.5107
-2.6466 -1.4064 0.9152 5.3345 0.2418 -1.207 2.9192 0.4433 7.6148
5.8048 -0.9304 2.4165 -3.654 2.1021 9.5794 -1.8444 1.401 0.9181 -
2.1292 1.8445 -3.0866 -1.9604 1.4153 -2.8178 -3.4027 0.1676 -
1.147 5.0718 -1.0358 -0.1501 -1.1855 0.6601 5.0402 -0.9092 -
2.4024 0.077 -2.4068 1.5406 7.1478</array>
</property>
</me:FourierSeries>
<me:FourierSeries>
<property dictRef="coeficients">
<array units="kJ/mol">0 0 0 0 0 0 0 0 0
0 0 0 0 0 0 0 0 0 0 0 0
0 0 0 0 0 0 0 0 0 0 0 0
0 0 0 0 0 0 0 0 0 0 0 0
0 0 0 0 0 0 0 0 0 0 0 0

0 0 0 0 0 0 0 0 0 0 0 0
0 0 0 0 -46.7996 -86.3782 -51.9035 -9.6314
10.3147 11.1339 6.8298 0 -6.5803 -27.1138 -30.8416
-15.5604 -3.5325 -6.6562 -3.2826 0 0.2873 -7.5225
-9.1409 -4.4772 3.1617 -4.7325 -1.2867 0 -2.9063
-5.2786 0.7585 4.3721 12.1644 4.6388 7.6516 0 -0.5668
5.7651 -2.3327 -1.7814 -3.1336 -5.6478 -2.0997 0
0.1096 6.4214 -0.9078 -2.6479 -3.2002 -7.0327 -3.6179
0 -3.9411 -2.6513 -1.4811 1.359 6.3903 6.6232 7.0753 0
0 0 0 0 0 0 0 0 11.3665 -24.4552
-22.849 -10.6532 -1.0768 2.4936 -0.2966 0 6.0393 -
14.1705 -21.8435 -18.8154 -6.563 2.031 -0.5383 0 1.744 -
5.0433 -5.025 -6.4009 -5.7628 1.4648 -0.9394 0 1.9947 -2.9445
2.2881 2.6106 1.9724 7.5637 0.9575 0 2.5931 -4.8891 0.1678 0.582 -
4.2267 3.4448 -0.8869 0 0.2144 -2.3557 0.7626 -1.5951 -3.4711
0.6905 -5.384 0 2.1028 0.1886 0.7281 2.1279 -1.8869 4.2587 0.0401 0
0 0 0 0 0 0 0 0 14.1451 19.006
11.1954 2.3876 0.3029 -7.1094 -6.2173 0 0.4371 7.1343 -
1.5454 -1.9576 3.6821 0.0601 3.9978 0 -5.3548 6.412 -2.5323 -
1.146 0.0226 0.1652 4.4074 0 -2.3181 8.1291 -0.1833 -1.6963 -
5.9099 -8.364 -1.518 0 -0.9448 -1.103 -2.6576 0.6961 3.8413 -1.4499
6.7603 0 -4.3034 0.3357 -3.9219 0.6501 8.0813 3.5018 4.9421 0
1.7504 7.1466 -0.0425 0.4773 -3.1714 -6.7311 -3.217 0 0
0 0 0 0 0 0 0 -13.5144 -3.8662 -
1.7613 0.7659 1.7058 1.8052 -0.7155 0 1.3502 6.647 10.5188 6.2076
2.5827 1.7781 1.559 0 1.0892 3.9634 4.0167 1.2929 -2.8329 0.0151 -
0.3925 0 -0.8493 -2.1742 1.4875 -1.5036 -5.8206 -2.0753
-4.0448 0 1.5024 -0.4997 1.3015 0.8646 -1.6963 -1.8872
-0.9082 0 2.857 1.4067 2.4039 1.0183 4.3865 2.7724 2.17 0 -
0.3708 -3.152 -1.8176 -1.7464 0.8297 -1.9431 -0.6343 0 0
0 0 0 0 0 0 0 -7.2862 -4.8279 -
1.7195 3.9105 3.1477 3.2113 -0.3275 0 0.9285 -1.5632 1.9063 1.8106 -
1.7146 -0.9567 -1.6069 0 2.9051 -0.5651 1.8182 -0.8884 -
2.3604 -0.037 -1.3255 0 -0.3983 -7.6052 -1.7639 0.9653
2.2063 3.3193 0.8335 0 1.3464 2.7157 2.5597 0.7868 0.0628 -0.0376 -
2.0104 0 4.6436 2.3757 5.0202 -0.2767 -4.1436 -2.0716 -2.8695
0 -0.4829 -6.7107 -2.9005 -3.6234 2.1882 1.2603
0.9095 0 0 0 0 0 0 0 0 0 10.2843
9.8285 8.2162 2.7894 -1.462 -2.3858 -0.11 0 -2.1339 -3.2013
-6.4145 -1.8013 0.6379 -0.3051 -1.2125 0 -2.758
2.4933 -5.1791 -4.4232 2.6001 -1.2764 2.5189 0 -0.7114
6.5557 -2.2952 1.1796 2.3174 -3.5872 5.3309 0 -3.1851
2.7534 -1.7328 -0.9174 5.431 -2.3914 1.9367 0 -3.2346
-1.3825 -3.19 -0.6713 -2.0102 -1.7986 -0.0616 0
2.1046 3.3733 1.3424 0.0279 -0.4574 -0.7726 -0.038 0 0 0
0 0 0 0 0 0 2.6958 1.7953 4.0025 -0.3713 -
4.5875 0.6439 0.2647 0 1.5971 -6.3356 -1.3432 -1.1158 -3.2716
4.403 -2.9861 0 1.5218 -1.7078 1.3428 -1.8416 -0.3675
2.937 -3.4818 0 5.1895 4.7263 4.9223 2.8663 -6.2939 1.5117
0.8971 0 -0.3204 -5.424 -0.6755 -2.1932 -1.7737

2.1888 -3.6427 0 -0.663 -8.5812 -4.2181 1.2323 -2.3898
7.3094 -2.0189 0 3.2599 2.9676 7.1008 3.7834 -1.854 4.1016 -
1.3332</array>
</property>
</me:FourierSeries>
</me:DOSCMethod>
</molecule>
<molecule spinMultiplicity="2" id="[O]COOC=O">
<atomArray>
<atom id="a1" elementType="C" x3="0.954980" y3="-0.022450" z3="0.299400" />
<atom id="a2" elementType="O" x3="2.105480" y3="-0.012280" z3="-0.381780" />
<atom id="a3" elementType="H" x3="0.557020" y3="1.009540" z3="0.313910" />
<atom id="a4" elementType="H" x3="0.212100" y3="-0.676420" z3="-0.186430" />
<atom id="a5" elementType="C" x3="1.155350" y3="1.335850" z3="2.927910" />
<atom id="a6" elementType="O" x3="0.018600" y3="1.616690" z3="2.691670" />
<atom id="a7" elementType="H" x3="1.839870" y3="1.873850" z3="3.599580" />
<atom id="a8" elementType="O" x3="1.022200" y3="-0.541040" z3="1.619660" />
<atom id="a9" elementType="O" x3="1.855810" y3="0.305230" z3="2.393780" />
</atomArray>
<bondArray>
<bond atomRefs2="a2 a1" order="1" />
<bond atomRefs2="a4 a1" order="1" />
<bond atomRefs2="a1 a3" order="1" />
<bond atomRefs2="a1 a8" order="1" id="bond18" />
<bond atomRefs2="a8 a9" order="1" id="bond89" />
<bond atomRefs2="a9 a5" order="1" id="bond95" />
<bond atomRefs2="a6 a5" order="2" />
<bond atomRefs2="a5 a7" order="1" />
</bondArray>
<propertyList>
<property dictRef="me:ZPE">
<scalar units="kJ/mol">-265.3280773</scalar>
</property>
<property dictRef="me:vibFreqs">
<array units="cm-1">64.1612 148.089 256.3099 335.2107 437.4186 635.8246 815.6395
863.8643 919.6432 1030.255 1043.5068 1132.0953 1162.5421 1231.5233 1345.7974
1369.8066 1391.0231 1886.0615 2995.5547 3066.6843 3144.1323</array>
</property>
<property dictRef="me:spinMultiplicity">
<scalar units="cm-1">2</scalar>
</property>
<property dictRef="me:hessian">
<matrix matrixType="squareSymmetricLT" rows="27"
units="Hartree/Bohr2">0.49203686 0.04050488 0.52020963 0.02582662 0.01634467
0.44685339 -0.22200276 -0.00514240 0.09341380 0.29932308 0.00257831 -0.02972727
0.01161412 0.00505489 0.03223828 0.08809152 0.00952056 -0.11245463 -0.15365233 -
0.01509753 0.15268933 -0.05783334 0.08886827 -0.00105089 -0.03317502 0.02112250
0.01380877 0.08123956 0.06937363 -0.25155072 0.00367911 0.01513902 -0.00477710 -
0.01112642 -0.09529260 0.28501033 0.00086128 -0.02221123 -0.04216801 0.01153015 -
0.01028413 0.00160698 -0.00555056 0.00433929 0.05447308 -0.14048274 -0.11115038 -

0.08338629 -0.04147261 -0.01256985 0.00470712 0.00851077 0.00764528 0.00204421
0.17510694 -0.10488781 -0.13627610 -0.07432460 -0.00342978 0.00042867 0.00264626 -
0.01790143 -0.01554073 -0.01079196 0.11722782 0.14411298 -0.06396565 -0.05335682 -
0.09314994 0.00995895 0.00345302 0.00856250 -0.00394155 -0.00242655 0.00310191
0.08384274 0.07171256 0.11120875 -0.00070182 -0.01130644 0.01547541 0.00202104
0.00112159 -0.00453778 0.00212676 0.00041035 -0.00441091 0.00055913 0.00114297 -
0.00432044 1.05414629 0.00201973 0.00586984 -0.01350950 -0.00232584 -0.00054091
0.00401193 -0.00076518 0.00054365 0.00199350 -0.00055574 -0.00054273 0.00316390 -
0.10344461 0.46105994 0.00209262 -0.00575593 -0.00564171 -0.00047896 0.00038248
0.00037674 -0.00093030 0.00013495 -0.00151758 -0.00035064 0.00056301 0.00043598
0.25737268 0.16775802 0.41866950 0.00107697 0.00404951 -0.00788307 -0.00083849 -
0.00048978 0.00235140 -0.00132215 0.00038693 0.00264003 -0.00050337 -0.00022375
0.00234022 -0.77482048 0.13566693 -0.17473073 0.87068760 -0.00024457 -0.00171319
0.00599609 0.00095394 0.00039790 -0.00153160 0.00010362 -0.00037130 -0.00084258
0.00003193 0.00020985 -0.00039723 0.14534385 -0.12349973 0.01798808 -0.20818893
0.12378713 0.00029544 0.00132216 -0.00510938 -0.00061314 -0.00014164 0.00153031
0.00013513 -0.00109052 0.00055165 -0.00043202 -0.00023713 0.00079816 -0.16264316
0.01217121 -0.12692705 0.17001404 -0.00968994 0.08904846 0.00015635 0.00096474 -
0.00153557 -0.00022630 -0.00018339 0.00031026 -0.00008735 -0.00026913 0.00033931 -
0.00004487 -0.00016771 0.00028921 -0.14626472 -0.08516233 -0.09622685 -0.03194076 -
0.00858631 -0.02749208 0.16716671 -0.00052616 -0.00201562 0.00057414 0.00010612
0.00016876 -0.00032283 0.00016191 0.00005550 -0.00012127 0.00000092 0.00020726 -
0.00058670 -0.07979836 -0.12174594 -0.07790432 0.00995273 0.01751108 0.00226010
0.07943989 0.12392224 0.00018120 0.00019437 0.00197448 0.00029722 0.00019036 -
0.00025296 0.00001309 0.00027781 -0.00025010 -0.00000259 0.00012279 0.00014201 -
0.09253473 -0.07987529 -0.15769085 -0.01336007 -0.00488330 0.01134915 0.11497634
0.10526857 0.15011355 -0.05820411 -0.00684364 -0.02254831 -0.00587791 -0.01671816
0.04397431 0.00191486 0.00415720 -0.00983529 -0.00447227 0.00675651 -0.02283895
0.00943686 0.00251480 0.00227543 0.00420914 0.00608881 0.00665461 0.00084012
0.00411322 0.00156783 0.18363981 -0.01263213 -0.10369192 0.04652711 -0.01146770 -
0.00002603 0.01226470 0.00131806 -0.01635000 0.03886799 -0.00180220 0.00456690 -
0.02216055 -0.02141090 -0.04371739 -0.03427094 -0.00819282 0.00385532 0.00419175
0.00560326 0.00090905 -0.00423628 0.10287934 0.27601265 -0.01566925 0.08852682 -
0.15971123 0.03920899 0.01243895 -0.05544246 -0.00361900 0.00776320 -0.01973557 -
0.00792644 0.00588406 -0.02098465 -0.02462300 -0.02450952 -0.02233669 0.00251547
0.00504273 0.00107268 0.00332222 -0.00480022 0.00286344 0.06803412 -0.01397264
0.37284446 -0.01404541 0.00005546 -0.01831170 0.00224897 0.00008390 0.00494674 -
0.00137411 -0.00155069 0.00238178 0.00279902 0.00148317 -0.00136452 -0.14650306
0.05205225 0.01097676 -0.06654847 0.06449765 0.01408118 0.01040082 -0.01345027 -
0.01113829 -0.13148649 -0.05429490 -0.06124311 0.34450873 0.00381412 -0.00110466
0.00309886 0.00111176 0.00183772 -0.00036506 0.00238484 0.00298036 -0.00094960
0.00117221 0.00283390 0.00059838 0.06794155 -0.17742673 -0.06889536 0.06703917 -
0.02017706 -0.00878598 0.00836099 -0.01901233 -0.01705903 -0.10294808 -0.12155859 -
0.07637338 -0.04887657 0.33162741 -0.03771378 -0.03458462 -0.03059298 0.00033532 -
0.00255562 0.00338419 0.00113531 -0.00155086 0.00393764 0.00150393 0.00442502 -
0.01011473 0.02022195 -0.07120425 -0.10536835 0.01611270 -0.01168225 0.02768602
0.00601715 -0.02436746 -0.00824872 -0.06728375 -0.02721113 -0.09856997 0.05967116
0.16873117 0.21788689</matrix>
</property>
<property dictRef="me:sigma" default="true">

<scalar>5.0</scalar>
</property>
<property dictRef="me:epsilon" default="true">
<scalar>50.0</scalar>
</property>
<property dictRef="me:vibFreqsFromHessian">
<scalar calculated="20220120_074051" units="cm-1">64.1609 148.088 256.309 335.209 437.417 635.822 815.636 863.861 919.64 1030.25 1043.5 1132.09 1162.54 1231.52 1345.79 1369.8 1391.02 1886.05 2995.54 3066.67 3144.12 </scalar>
</property>
<property dictRef="me:frequenciesScaleFactor" default="true">
<scalar>0.9522</scalar>
</property>
<property dictRef="me:symmetryNumber" default="true">
<scalar>1</scalar>
</property>
</propertyList>
<me:DOSCMethod xsi:type="me:ClassicalCoupledRotors">
<me:MCPoints>10000</me:MCPoints>
<me:RotorArray>
<me:Rotor>
<bondRef>bond18</bondRef>
<me:HinderedRotorPotential format="numerical" units="kJ/mol" expansionSize="10" UseSineTerms="yes">
<me:PotentialPoint angle="0" potential="0.0" />
<me:PotentialPoint angle="5.0" potential="0.2761580012141134" />
<me:PotentialPoint angle="10.0" potential="0.6924434209198528" />
<me:PotentialPoint angle="15.0" potential="1.2730527179159226" />
<me:PotentialPoint angle="20.0" potential="1.8671693568371694" />
<me:PotentialPoint angle="25.0" potential="2.6188077331921886" />
<me:PotentialPoint angle="30.0" potential="3.450568774421475" />
<me:PotentialPoint angle="35.0" potential="4.214989708680938" />
<me:PotentialPoint angle="40.0" potential="5.045526368457495" />
<me:PotentialPoint angle="45.0" potential="5.857802986296422" />
<me:PotentialPoint angle="50.0" potential="6.621586456020039" />
<me:PotentialPoint angle="55.0" potential="7.3134740936404405" />
<me:PotentialPoint angle="60.0" potential="7.918118978397356" />
<me:PotentialPoint angle="65.0" potential="8.42943539648047" />
<me:PotentialPoint angle="70.0" potential="8.745159636360679" />
<me:PotentialPoint angle="75.0" potential="9.015933229175435" />
<me:PotentialPoint angle="80.0" potential="9.25258209270073" />
<me:PotentialPoint angle="85.0" potential="9.402136429830179" />
<me:PotentialPoint angle="90.0" potential="9.694178510140539" />
<me:PotentialPoint angle="95.0" potential="10.095755632548244" />
<me:PotentialPoint angle="100.0" potential="10.656264349264273" />
<me:PotentialPoint angle="105.0" potential="11.349655301655103" />
<me:PotentialPoint angle="110.0" potential="12.172381351058673" />
<me:PotentialPoint angle="115.0" potential="13.098250264898551" />
<me:PotentialPoint angle="120.0" potential="13.864971253449141" />
<me:PotentialPoint angle="125.0" potential="14.641546136568241" />

<me:PotentialPoint angle="130.0" potential="15.024993803847817" />
<me:PotentialPoint angle="135.0" potential="15.265842252527362" />
<me:PotentialPoint angle="140.0" potential="14.976328631078724" />
<me:PotentialPoint angle="145.0" potential="14.627313650189373" />
<me:PotentialPoint angle="150.0" potential="14.149446036254849" />
<me:PotentialPoint angle="155.0" potential="13.248008277660396" />
<me:PotentialPoint angle="160.0" potential="12.381307966832196" />
<me:PotentialPoint angle="165.0" potential="11.437216766046928" />
<me:PotentialPoint angle="170.0" potential="10.286414524294395" />
<me:PotentialPoint angle="175.0" potential="9.195519777005009" />
<me:PotentialPoint angle="180.0" potential="8.123725821193572" />
<me:PotentialPoint angle="185.0" potential="7.101879007825264" />
<me:PotentialPoint angle="190.0" potential="6.161911365665716" />
<me:PotentialPoint angle="195.0" potential="5.333618265437" />
<me:PotentialPoint angle="200.0" potential="4.6476324879004745" />
<me:PotentialPoint angle="205.0" potential="4.129752890318114" />
<me:PotentialPoint angle="210.0" potential="3.800340575101435" />
<me:PotentialPoint angle="215.0" potential="3.6727769247836286" />
<me:PotentialPoint angle="220.0" potential="3.7525939887762005" />
<me:PotentialPoint angle="225.0" potential="4.035976993775664" />
<me:PotentialPoint angle="230.0" potential="4.462220888471799" />
<me:PotentialPoint angle="235.0" potential="5.001869591345938" />
<me:PotentialPoint angle="240.0" potential="5.682182057768046" />
<me:PotentialPoint angle="245.0" potential="6.403826564065236" />
<me:PotentialPoint angle="250.0" potential="7.224311566406766" />
<me:PotentialPoint angle="255.0" potential="7.988401180132365" />
<me:PotentialPoint angle="260.0" potential="8.76119649089743" />
<me:PotentialPoint angle="265.0" potential="9.46989776798268" />
<me:PotentialPoint angle="270.0" potential="10.070291077845447" />
<me:PotentialPoint angle="275.0" potential="10.562796241765808" />
<me:PotentialPoint angle="280.0" potential="10.758060235158768" />
<me:PotentialPoint angle="285.0" potential="10.746525237429449" />
<me:PotentialPoint angle="290.0" potential="10.50151858101109" />
<me:PotentialPoint angle="295.0" potential="9.9793441776307" />
<me:PotentialPoint angle="300.0" potential="9.263347216491622" />
<me:PotentialPoint angle="305.0" potential="8.212527898505758" />
<me:PotentialPoint angle="310.0" potential="7.05098497586548" />
<me:PotentialPoint angle="315.0" potential="5.777662371604118" />
<me:PotentialPoint angle="320.0" potential="4.501083435079434" />
<me:PotentialPoint angle="325.0" potential="3.3346253819900356" />
<me:PotentialPoint angle="330.0" potential="2.2463199956812443" />
<me:PotentialPoint angle="335.0" potential="1.385517489122569" />
<me:PotentialPoint angle="340.0" potential="0.7161690033028294" />
<me:PotentialPoint angle="345.0" potential="0.25659225733160285" />
<me:PotentialPoint angle="350.0" potential="-0.018833385905815316" />
<me:PotentialPoint angle="355.0" potential="-0.09551772711156829" />
<me:PotentialPoint angle="360.0" potential="0.007655353380957006" />
</me:HinderedRotorPotential>

</me:Rotor>

<me:Rotor>
<bondRef>bond89</bondRef>
<me:HinderedRotorPotential format="numerical" units="kJ/mol" expansionSize="10" UseSineTerms="yes">
<me:PotentialPoint angle="0" potential="0.0" />
<me:PotentialPoint angle="5.0" potential="0.5585118118801405" />
<me:PotentialPoint angle="10.0" potential="1.3380073447339804" />
<me:PotentialPoint angle="15.0" potential="2.052997187415686" />
<me:PotentialPoint angle="20.0" potential="2.869841262760882" />
<me:PotentialPoint angle="25.0" potential="3.6834805823010446" />
<me:PotentialPoint angle="30.0" potential="4.463391775938697" />
<me:PotentialPoint angle="35.0" potential="5.156791873403421" />
<me:PotentialPoint angle="40.0" potential="5.9080303933674045" />
<me:PotentialPoint angle="45.0" potential="6.623903903211694" />
<me:PotentialPoint angle="50.0" potential="7.310067607063853" />
<me:PotentialPoint angle="55.0" potential="7.815304539461602" />
<me:PotentialPoint angle="60.0" potential="8.33038324784308" />
<me:PotentialPoint angle="65.0" potential="8.770232141306941" />
<me:PotentialPoint angle="70.0" potential="9.11751850380165" />
<me:PotentialPoint angle="75.0" potential="9.399592724543053" />
<me:PotentialPoint angle="80.0" potential="9.521785640240143" />
<me:PotentialPoint angle="85.0" potential="9.61147367345506" />
<me:PotentialPoint angle="90.0" potential="9.624721969422074" />
<me:PotentialPoint angle="95.0" potential="9.557000047956372" />
<me:PotentialPoint angle="100.0" potential="9.415100910342993" />
<me:PotentialPoint angle="105.0" potential="9.18725747194686" />
<me:PotentialPoint angle="110.0" potential="8.878998893093316" />
<me:PotentialPoint angle="115.0" potential="8.502715117851672" />
<me:PotentialPoint angle="120.0" potential="8.053520963371525" />
<me:PotentialPoint angle="125.0" potential="7.557513368929732" />
<me:PotentialPoint angle="130.0" potential="7.010707880515047" />
<me:PotentialPoint angle="135.0" potential="6.464267246053225" />
<me:PotentialPoint angle="140.0" potential="5.90086602828926" />
<me:PotentialPoint angle="145.0" potential="5.421397885760669" />
<me:PotentialPoint angle="150.0" potential="5.047855915154377" />
<me:PotentialPoint angle="155.0" potential="4.8796660998609225" />
<me:PotentialPoint angle="160.0" potential="5.034494132840412" />
<me:PotentialPoint angle="165.0" potential="5.63018507970309" />
<me:PotentialPoint angle="170.0" potential="6.619220263431349" />
<me:PotentialPoint angle="175.0" potential="8.443405435591982" />
<me:PotentialPoint angle="180.0" potential="11.068462213891467" />
<me:PotentialPoint angle="185.0" potential="14.489102022608211" />
<me:PotentialPoint angle="190.0" potential="18.608094488760592" />
<me:PotentialPoint angle="195.0" potential="23.19594738921881" />
<me:PotentialPoint angle="200.0" potential="27.98411444152327" />
<me:PotentialPoint angle="205.0" potential="27.86507161990799" />
<me:PotentialPoint angle="210.0" potential="14.385669695548678" />
<me:PotentialPoint angle="215.0" potential="16.21414019505145" />
<me:PotentialPoint angle="220.0" potential="18.607070432157965" />
<me:PotentialPoint angle="225.0" potential="21.470399072284117" />

```
    <me:PotentialPoint angle="230.0" potential="24.679547185104497" />
    <me:PotentialPoint angle="235.0" potential="27.77946866499353" />
    <me:PotentialPoint angle="240.0" potential="31.120030842109117" />
    <me:PotentialPoint angle="245.0" potential="34.27497821711651" />
    <me:PotentialPoint angle="250.0" potential="37.29566477183546" />
    <me:PotentialPoint angle="255.0" potential="39.86813266879474" />
    <me:PotentialPoint angle="260.0" potential="42.14295124995817" />
    <me:PotentialPoint angle="265.0" potential="43.802858427992994" />
    <me:PotentialPoint angle="270.0" potential="44.9604865880815" />
    <me:PotentialPoint angle="275.0" potential="45.52881487621926" />
    <me:PotentialPoint angle="280.0" potential="45.460325570065194" />
    <me:PotentialPoint angle="285.0" potential="44.766231786864374" />
    <me:PotentialPoint angle="290.0" potential="43.47984622300775" />
    <me:PotentialPoint angle="295.0" potential="41.72113067854406" />
    <me:PotentialPoint angle="300.0" potential="39.35670460124135" />
    <me:PotentialPoint angle="305.0" potential="36.70750231213834" />
    <me:PotentialPoint angle="310.0" potential="33.62201638022527" />
    <me:PotentialPoint angle="315.0" potential="30.43751762795453" />
    <me:PotentialPoint angle="320.0" potential="27.20795777641082" />
    <me:PotentialPoint angle="325.0" potential="23.826448110538838" />
    <me:PotentialPoint angle="330.0" potential="20.781930820970484" />
    <me:PotentialPoint angle="335.0" potential="18.095687100086614" />
    <me:PotentialPoint angle="340.0" potential="15.876401376011849" />
    <me:PotentialPoint angle="345.0" potential="14.21175370540037" />
    <me:PotentialPoint angle="350.0" potential="13.146595789287256" />
    <me:PotentialPoint angle="355.0" potential="3.8814839011998843" />
    <me:PotentialPoint angle="360.0" potential="-0.02539268302596788" />
   </me:HinderedRotorPotential>

  </me:Rotor>
  <me:Rotor>
   <bondRef>bond95</bondRef>
   <me:HinderedRotorPotential format="numerical" units="kJ/mol" expansionSize="10"
UseSineTerms="yes">
    <me:PotentialPoint angle="0" potential="0.0" />
    <me:PotentialPoint angle="5.0" potential="0.39108211697479633" />
    <me:PotentialPoint angle="10.0" potential="1.6248826960935443" />
    <me:PotentialPoint angle="15.0" potential="3.7045776089533633" />
    <me:PotentialPoint angle="20.0" potential="6.5535063008644805" />
    <me:PotentialPoint angle="25.0" potential="10.13295730605312" />
    <me:PotentialPoint angle="30.0" potential="14.324740926499892" />
    <me:PotentialPoint angle="35.0" potential="17.0965794128406" />
    <me:PotentialPoint angle="40.0" potential="14.951351595265894" />
    <me:PotentialPoint angle="45.0" potential="16.37485988368472" />
    <me:PotentialPoint angle="50.0" potential="20.39232892260245" />
    <me:PotentialPoint angle="55.0" potential="24.615208579963998" />
    <me:PotentialPoint angle="60.0" potential="28.919782157524086" />
    <me:PotentialPoint angle="65.0" potential="33.34596959789889" />
    <me:PotentialPoint angle="70.0" potential="37.39255945204994" />
    <me:PotentialPoint angle="75.0" potential="41.358422171721834" />
```

<me:PotentialPoint angle="80.0" potential="44.965709065689204" />
<me:PotentialPoint angle="85.0" potential="48.06212995286176" />
<me:PotentialPoint angle="90.0" potential="50.52797867273736" />
<me:PotentialPoint angle="95.0" potential="52.19134909524589" />
<me:PotentialPoint angle="100.0" potential="53.05184242406038" />
<me:PotentialPoint angle="105.0" potential="53.00253970676374" />
<me:PotentialPoint angle="110.0" potential="52.14970272945837" />
<me:PotentialPoint angle="115.0" potential="50.425301218594626" />
<me:PotentialPoint angle="120.0" potential="47.96835032454164" />
<me:PotentialPoint angle="125.0" potential="44.894582740416965" />
<me:PotentialPoint angle="130.0" potential="41.224662827174086" />
<me:PotentialPoint angle="135.0" potential="37.18602782501941" />
<me:PotentialPoint angle="140.0" potential="32.806944212298994" />
<me:PotentialPoint angle="145.0" potential="28.324977729892627" />
<me:PotentialPoint angle="150.0" potential="23.840364666583415" />
<me:PotentialPoint angle="155.0" potential="19.403533342627313" />
<me:PotentialPoint angle="160.0" potential="15.251587044496182" />
<me:PotentialPoint angle="165.0" potential="11.460202305633782" />
<me:PotentialPoint angle="170.0" potential="8.052365329836949" />
<me:PotentialPoint angle="175.0" potential="5.216253086309122" />
<me:PotentialPoint angle="180.0" potential="2.9868557270158633" />
<me:PotentialPoint angle="185.0" potential="1.4267739819799683" />
<me:PotentialPoint angle="190.0" potential="0.43996361074818596" />
<me:PotentialPoint angle="195.0" potential="0.24532999816470152" />
<me:PotentialPoint angle="200.0" potential="0.778397052867437" />
<me:PotentialPoint angle="205.0" potential="2.0219296896366075" />
<me:PotentialPoint angle="210.0" potential="3.9025368922897963" />
<me:PotentialPoint angle="215.0" potential="6.446556382601747" />
<me:PotentialPoint angle="220.0" potential="9.567301624832506" />
<me:PotentialPoint angle="225.0" potential="13.19246627192237" />
<me:PotentialPoint angle="230.0" potential="17.188370075480396" />
<me:PotentialPoint angle="235.0" potential="21.52483376725961" />
<me:PotentialPoint angle="240.0" potential="26.07035098953783" />
<me:PotentialPoint angle="245.0" potential="30.636811119720154" />
<me:PotentialPoint angle="250.0" potential="35.2051861326849" />
<me:PotentialPoint angle="255.0" potential="39.616811728874" />
<me:PotentialPoint angle="260.0" potential="43.745041665519295" />
<me:PotentialPoint angle="265.0" potential="47.37412613950161" />
<me:PotentialPoint angle="270.0" potential="50.51768263776395" />
<me:PotentialPoint angle="275.0" potential="52.990939080524704" />
<me:PotentialPoint angle="280.0" potential="54.666694237473735" />
<me:PotentialPoint angle="285.0" potential="55.43800857500173" />
<me:PotentialPoint angle="290.0" potential="55.22322937603081" />
<me:PotentialPoint angle="295.0" potential="53.992066960026676" />
<me:PotentialPoint angle="300.0" potential="51.78005337427865" />
<me:PotentialPoint angle="305.0" potential="48.689545626196946" />
<me:PotentialPoint angle="310.0" potential="44.79891022458479" />
<me:PotentialPoint angle="315.0" potential="40.452194544305236" />
<me:PotentialPoint angle="320.0" potential="35.792039009745075" />
<me:PotentialPoint angle="325.0" potential="31.017259934574927" />

```
<me:PotentialPoint angle="330.0" potential="26.192159249218562" />
<me:PotentialPoint angle="335.0" potential="21.64405369380712" />
<me:PotentialPoint angle="340.0" potential="17.43613630405114" />
<me:PotentialPoint angle="345.0" potential="13.664091745102574" />
<me:PotentialPoint angle="350.0" potential="10.3896531989045" />
<me:PotentialPoint angle="355.0" potential="7.701736835549704" />
<me:PotentialPoint angle="360.0" potential="5.56127762733325" />
</me:HinderedRotorPotential>

</me:Rotor>
</me:RotorArray>
</me:DOSCMethod>
<me:DistributionCalcMethod default="true" name="Boltzmann" />
<me:energyTransferModel name="ExponentialDown" default="true" />
<me:deltaEDown default="NEEDS TO BE CHECKED**">130.0</me:deltaEDown>
</molecule>
<molecule spinMultiplicity="2" id="TS_[O]OCOC=O_OOCO[C]=O">
<atomArray>
<atom id="a1" elementType="C" x3="0.628546" y3="0.715130" z3="-0.485169"/>
<atom id="a2" elementType="O" x3="-0.542153" y3="0.857921" z3="0.344263"/>
<atom id="a3" elementType="H" x3="0.307374" y3="0.585391" z3="-1.521735"/>
<atom id="a4" elementType="H" x3="1.220060" y3="1.614274" z3="-0.322409"/>
<atom id="a5" elementType="C" x3="-1.133431" y3="-0.275211"
z3="0.732105"/>
<atom id="a6" elementType="O" x3="-2.132152" y3="-0.375653"
z3="1.360883"/>
<atom id="a7" elementType="H" x3="-0.389053" y3="-1.246867"
z3="0.305901"/>
<atom id="a8" elementType="O" x3="1.386788" y3="-0.363836" z3="-
0.057109"/>
<atom id="a9" elementType="O" x3="0.654022" y3="-1.511140" z3="-
0.356731"/>
</atomArray>
<bondArray>
<bond atomRefs2="a3 a1" order="1"/>
<bond atomRefs2="a1 a4" order="1"/>
<bond atomRefs2="a1 a8" order="1"/>
<bond atomRefs2="a1 a2" order="1"/>
<bond atomRefs2="a9 a8" order="1"/>
<bond atomRefs2="a9 a7" order="1"/>
<bond atomRefs2="a2 a5" order="1"/>
<bond atomRefs2="a5 a6" order="2"/>
</bondArray>
<propertyList>
<property dictRef="me:ZPE">
<scalar units="kJ/mol" >-118.424</scalar>
</property>
<property dictRef="me:vibFreqs">
```

<array units="cm-1">161.6899 321.7878 376.8557 442.6456 531.4604 559.6671 747.9046 992.809 1017.4146 1055.5696 1105.0799 1157.9299 1195.5406 1302.4587 1430.9187 1497.9747 1709.7137 1960.5988 3135.6295 3236.2682</array>
</property>
<property dictRef="me:spinMultiplicity">
<scalar units="cm-1">2</scalar>
</property>
<!-- <property dictRef="me:hessian">
<matrix matrixType="squareSymmetricLT" rows="42" units="Hartree/Bohr2">0.46665261 0.13375680 0.62913590 0.09936036 0.0161322 2 0.59639833 -0.09956577 -0.00431390 0.06326299 0.32109292 0.02023892 -0.10441026 - 0.00381544 0.06544715 0.37542691 0.04412736 -0.00326363 - 0.12140942 -0.18266876 - 0.05551294 0.19201401 -0.07019412 -0.01929126 -0.08555129 -0.00842758 -0.00164168 0.03989916 0.08929326 -0.02228596 -0.06283085 -0.04545656 0.00197539 0.00610689 - 0.00044929 0.00728966 0 .06004818 -0.08088858 -0.03208608 -0.29424000 0.00980045 - 0.00150190 - 0.02165206 0.08217677 0.03451248 0.32214111 -0.13467560 -0.13275839 -0 .03193227 -0.02373140 -0.02484764 0.00715481 -0.00122593 -0.00681582 - 0.00081104 0.15025502 -0.12955540 -0.24546910 -0.03571479 0.00301070 0 .00689342 -0.00070148 - 0.00053974 0.00171595 -0.00014855 0.13608848 0. 26904589 -0.03841568 -0.05007206 - 0.06441592 0.02002302 0.01980193 -0. 00047870 -0.01392176 -0.02241420 0.00084520 0.02296297 0.04039367 0.06 190789 -0.03571184 -0.03240491 0.01565509 -0.09298618 - 0.02986194 0.04 185401 0.00344991 -0.00063996 -0.00223242 -0.00366900 0.00173408 0.004 25966 0.82396115 -0.07905096 0.04273286 0.01350915 -0.03756377 -0.2482 9187 0.03164971 0.01124045 -0.00178986 -0.00509584 0.00666076 -0.00147 321 -0.00177462 0.09218100 0.34454240 0.01538611 0.01992422 -0.0190870 8 0.04405463 0.02311450 - 0.06095867 0.00133059 0.00035164 -0.00064056 -0.00012005 -0.00101378 -0.00056856 - 0.43989605 -0.06108498 0.41104320 0.01299375 -0.00154936 -0.00505740 -0.05060779 - 0.05620227 0.03810233 -0.00239975 0.00032773 0.00151099 -0.00056980 -0.00009747 0.00046634 -0.65552625 -0.02647504 0.38196311 0.71472191 0.01712771 -0.01478141 - 0.00319845 -0.06738091 -0.00507148 0.04258521 -0.00256791 0.00027217 0 .00121837 - 0.00213473 0.00076515 0.00122149 -0.03103342 -0.07325754 0. 02044782 0.07758396 0.08097200 -0.00393241 0.00267785 0.00944960 0.037 64619 0.03543047 -0.01715147 0.00028791 -0.00026904 -0.00026642 0.0010 3190 -0.00014644 -0.00104209 0.38244424 0.01665246 -0.29018342 -0.4368 2599 -0.05060035 0.29759211 -0.02330695 0.01278471 0.00021085 0.018506 28 0.00690002 -0.00019649 0.00241661 -0.00061374 -0.00036812 0.0011449 4 -0.00059103 -0.00045391 0.02072335 -0.07495306 -0.04021412 -0.017632 97 0.01668266 0.01872004 -0.04744774 0.00989811 -0.01096688 0.00181126 -0.00258646 - 0.03474453 0.00087161 -0.00146742 0.00018782 0.00040990 -0.00045176 0.00062985 0.00027047 -0.01934227 -0.01199667 0.00837731 0 .00312842 0.00425081 -0.00064580 0.07986856 0.06568651 0.01545243 -0.0 0826963 0.00063059 -0.00327370 -0.00133085 0.01469248 -0.00154626 0.00 044971 -0.00033003 -0.00076482 0.00035090 0.00003629 - 0.04648960 0.046 32307 -0.02517020 0.02075826 -0.00969272 0.00329221 0.05688894 - 0.0373 6924 0.00198220 -0.14004588 0.05386226 -0.06625053 -0.05032397 0.03276 319 0.00773541 -0.01130119 0.01826739 -0.01089686 0.01255732 -0.013716 24 0.00484511 0.00952256 -0.00168027 -0.00712361 -0.00769933 -0.000954 01 0.00481745 -0.03428268 - 0.01091820 0.00555002 0.32948640 0.06502832 -0.15288164 0.06483918 0.05804739 0.01135088 -0.02213392 0.01108159 - 0.01011321 0.00455516 0.01864585 -0.02830295 0.01118506 0.01084840 -0. 07471657 -0.00374650 -0.00815785 0.01610281 0.00339425 - 0.05594745 0.0 2190597 0.03157604 -0.00898373 0.40643869 -0.05996201 0.04297212 - 0.11 397550 0.01461098 -0.01380285 0.01455427 -0.02713204 0.03281483 -0.008 15195

0.00434344 -0.00627341 0.00198683 0.00051344 -0.00250064 0.00312 819 -0.00119073 -
0.00023578 0.00038789 0.00312154 0.01123679 0.0076350 2 0.10455036 -0.02178841
0.14619138 0.02385380 -0.01008595 0.01030220 -0.01395650 -0.01279575 0.00399216 -
0.00161120 0.00249531 0.00170881 - 0.00008556 0.00366663 0.00023425 -0.06976370
0.10964089 0.04461940 0.0 0672023 -0.00732335 -0.00418933 0.07987916 -0.05812899 -
0.04657527 -0. 10791324 -0.09056252 -0.03885499 0.08287701 -0.01515753 -0.08052861 -
0 .00810657 -0.01663559 -0.00725997 0.00695473 -0.00410368 0.00640291 -0 .00186354
0.00561326 -0.00380499 0.00138826 0.00851902 0.02425045 -0.0 0637023 0.01144187 -
0.00925251 -0.00649340 0.01586934 -0.03495289 -0.0 2203728 -0.06864041 -0.18978399 -
0.04242266 0.06309373 0.29492961 0.00 887242 0.01198500 0.00664940 -0.00345581 -
0.00238294 0.00038955 0.0044 5692 0.00046043 0.00229470 -0.00186493 0.00325388
0.00172905 0.0438916 3 -0.03767831 -0.01756288 0.00027309 -0.00174559 -0.00207842 -
0.037708 73 0.01503769 -0.00276857 -0.04322736 -0.06788086 -0.05175612 0.028762 77
0.07895069 0.06310329
</matrix>
</property>-->
<property dictRef="me:imFreqs">
<scalar units="cm-1">1907.1332</scalar>
</property>
<property dictRef="me:frequenciesScaleFactor" default="true">
<scalar>0.75</scalar>
</property>
<property dictRef="me:symmetryNumber" default="true">
<scalar>1</scalar>
</property>
</propertyList>
<me:DOSCMethod default="true" name="ClassicalRotors" />
</molecule>
<molecule id="O=O">
<propertyList>
<property title="Energy" dictRef="me:ZPE">
<scalar units="kJ/mol">0</scalar>
</property>
<property dictRef="me:rotConsts">
<array units="cm-1">1.44914</array>
</property>
<property dictRef="me:symmetryNumber">
<scalar>2</scalar>
</property>
<!--><property dictRef="me:frequenciesScaleFactor">
<scalar>0.9854</scalar>
</property>-->
<property dictRef="me:vibFreqs">
<array units="cm-1">1777.2564</array>
</property>
<property dictRef="me:MW">
<scalar units="amu">32</scalar>
</property>
<property dictRef="me:spinMultiplicity">
<scalar>3</scalar>

</property>
<property dictRef="me:frequenciesScaleFactor" default="true">
<scalar>0.9522</scalar>
</property>
</propertyList>
<me:DOSCMethod default="true" name="ClassicalRotors" />
</molecule>
<molecule spinMultiplicity="2" id="TS_COC(=O)O[O]_[CH2]OC(=O)OO">
<atomArray>
<atom id="a1" elementType="C" x3="0.868023" y3="0.590300" z3="0.411834"/>
<atom id="a2" elementType="O" x3="0.336819" y3="-0.670140" z3="0.666824"/>
<atom id="a3" elementType="H" x3="1.596275" y3="0.601745" z3="-0.402084"/>
<atom id="a4" elementType="H" x3="1.167313" y3="1.064211" z3="1.342823"/>
<atom id="a5" elementType="H" x3="-0.179453" y3="1.218315" z3="-0.050957"/>
<atom id="a6" elementType="C" x3="-0.548245" y3="-1.188454" z3="-0.213124"/>
<atom id="a7" elementType="O" x3="-0.750234" y3="-2.349772" z3="-0.345902"/>
<atom id="a8" elementType="O" x3="-1.172558" y3="-0.257931" z3="-1.006198"/>
<atom id="a9" elementType="O" x3="-1.317939" y3="0.991726" z3="-0.403215"/>
</atomArray>
<bondArray>
<bond atomRefs2="a8 a9" order="1"/>
<bond atomRefs2="a8 a6" order="1"/>
<bond atomRefs2="a9 a5" order="1"/>
<bond atomRefs2="a3 a1" order="1"/>
<bond atomRefs2="a7 a6" order="2"/>
<bond atomRefs2="a6 a2" order="1"/>
<bond atomRefs2="a1 a2" order="1"/>
<bond atomRefs2="a1 a4" order="1"/>
</bondArray>
<propertyList>
<property dictRef="me:ZPE">
<scalar units="kJ/mol">-114.38</scalar>
</property>
<property dictRef="me:vibFreqs">
<array units="cm-1">146.3927 195.5254 369.2157 512.2759 572.198 599.3038 734.3464 777.606 868.3832 1070.1871 1101.3889 1162.2998 1199.9525 1241.5667 1272.5407 1484.7267 1671.4518 1963.1952 3119.4011 3249.7738</array>
</property>
<property dictRef="me:spinMultiplicity">
<scalar units="cm-1">2</scalar>
</property>
<!--<property dictRef="me:hessian">

<matrix matrixType="squareSymmetricLT" rows="27"
units="Hartree/Bohr2">0.38243612 0.15313639 0.39162260 -0.07843826 0.1137 0763
0.60241486 -0.09381013 -0.06891587 0.00971672 0.27156216 -0.09770 745 -0.21177012
0.04259783 0.17686449 0.43413414 0.00582076 0.04937180 -0.09365675 0.14220023 -
0.01105827 0.26118878 -0.16492564 -0.00903254 0.15100518 -0.00518844 -0.00499865
0.00736470 0.17339889 0.00436361 - 0.04289397 -0.02200792 -0.03410122 -0.01391903
0.02187603 0.00942474 0 .05329518 0.14077693 -0.01463552 -0.20658452 0.00722039
0.00858083 0.0 0561324 -0.15696346 0.00452374 0.21292104 -0.06150310 -0.04427693 -
0.0 6591979 -0.00431957 -0.00639931 -0.01072674 0.01019559 0.00860979 0.01 850483
0.06237443 -0.03603784 -0.08808275 -0.09782692 -0.02093322 -0.0 2314760 -0.02759705 -
0.00012097 0.00372679 -0.00130816 0.05009793 0.10 789057 -0.06529034 -0.11672150 -
0.28184186 0.00308848 0.00576717 0.012 20900 -0.00909646 -0.00610162 -0.01062354
0.07012377 0.11634111 0.2832 5138 0.06243534 -0.06906647 0.04828347 0.00741177
0.03382699 0.0059886 6 -0.01896116 0.01180545 -0.01289434 -0.00854624 0.00639993
0.00066656 -0.08546687 0.01602916 -0.03492247 0.00434720 0.00954618 -0.02606750
0.00881192 0.00216938 0.00020067 0.00025614 -0.00499668 0.00426023 -0. 00256013
0.00321483 0.10080020 0.02632035 -0.02634754 -0.01039348 -0.0 0039573 0.02126122 -
0.00033293 0.00616129 -0.00439253 -0.00071809 -0.0 1229153 0.00882443 -0.00458131 -
0.04858614 0.00886617 0.02602945 -0.00 796409 -0.05239941 0.00740933 -0.13596878 -
0.03247213 -0.08031619 -0.0 0080566 0.00459255 -0.00510879 -0.00051105 0.00640005
0.00139561 -0.01 549686 -0.00127784 -0.00603470 0.36612927 -0.03033664 -0.02507793 -
0.0 2706504 -0.02473132 -0.11460662 -0.01520468 0.00209084 0.00074605 0.00 186280 -
0.00243442 -0.00445936 0.00247867 0.00444885 -0.00311712 0.002 06909 0.14025670
1.14850080 0.01680579 -0.06017722 0.01757725 -0.08046 894 -0.00957535 -0.15331427
0.00040286 0.01089313 -0.00527467 -0.00157 334 0.00601481 0.00111061 -0.02955640
0.00075024 -0.00854183 0.1449520 0 0.08290794 0.37519427 0.00094120 0.00865525 -
0.00968526 -0.01151629 -0.05726711 -0.02233570 0.00034911 -0.00058063 0.00148750 -
0.00070810 -0.00242797 0.00056913 0.00364935 0.00051296 0.00161174 -0.10703364 -0
.12536292 -0.01464053 0.09782746 0.00303173 0.00472901 -0.00346886 -0. 04261978 -
0.04870207 -0.03288500 -0.00127029 -0.00133105 -0.00040008 - 0.00111922 -0.00151592
0.00061320 -0.00237577 0.00060045 -0.00077722 - 0.12673253 -0.82984209 -0.08442931
0.15221302 0.96032109 -0.00428775 0 .00988249 -0.00536097 -0.02728643 -0.05357755
0.00235324 -0.00018536 - 0.00215898 0.00080198 -0.00018468 -0.00185695 0.00017059
0.00335752 0. 00003233 0.00115262 -0.01496714 -0.08108514 -0.09524426 0.05009305 0.0
9469829 0.07850538 -0.00330386 0.01926176 -0.01319503 -0.03001633 0.00 080265 -
0.04684138 0.00193312 -0.00192260 0.00333772 0.00033342 -0.000 69112 -0.00018112
0.01340170 -0.01955972 -0.00865707 -0.09339479 0.022 78472 -0.05571434 0.01333496
0.01543541 -0.00395748 0.14605359 0.01623 112 -0.03907220 0.01172154 0.01746029
0.02181290 0.01772184 -0.0040089 1 0.00440676 -0.00417349 -0.00229519 0.00391625
0.00044792 -0.04674200 -0.01548642 -0.02490376 0.04695526 -0.11839369 0.04233022
0.02345779 -0.08145439 0.03288566 -0.08452695 0.42504822 -0.00146574 0.01265676 -
0.00485282 -0.04724540 -0.00132732 -0.03199141 -0.00127468 -0.00071006 0.00045053 -
0.00003429 -0.00084638 0.00039779 -0.00269016 -0.00951062 -0.00770414 -0.04796695
0.03042113 -0.15013298 -0.00566085 0.02181663 0.02060698 0.12158351 0.03963148
0.25983771 -0.11430585 0.06263782 -0 .04917635 0.00184560 -0.01264948 -0.00115433
0.00400418 -0.00219170 0. 00363922 0.00268461 -0.00268680 -0.00127562 0.04157296 -
0.00563828 0.0 4187179 -0.00495440 0.01328419 0.01979290 0.00315596 0.00343743 -
0.002 58172 -0.04834182 0.03346860 -0.01524544 0.11433874 -0.02871008 0.0454 6784 -
0.02200545 -0.01256956 -0.01773410 -0.01103659 0.00574640 -0.004 23141 0.00529374
0.00281402 -0.00258819 -0.00026482 0.05848819 -0.0262 6804 0.01540014 0.01467734 -

0.05375004 0.01128554 0.00079961 -0.002805 02 0.00117985 0.04841585 -0.20077743 -0.09213163 -0.08966178 0.2626864 0 -0.04024174 0.03226310 -0.01730171 -0.00682933 -0.00266856 -0.002068 89 0.00258594 -0.00192179 0.00341403 0.00210178 -0.00174489 -0.0000926 7 0.03543083 -0.01099324 0.00508970 0.00063683 0.00361524 0.01862588 -0.00143906 0.00483234 -0.00298557 0.00362520 -0.11566142 -0.08661167 0 .00412955 0.09227922 0.08193090</matrix>
</property>-->
<property dictRef="me:imFreqs">
<scalar units="cm-1">1787.8458</scalar>
</property>
<property dictRef="me:frequenciesScaleFactor" default="true">
<scalar>0.75</scalar>
</property>
</propertyList>
</molecule>
<molecule id="O">
<atomArray>
<atom id="a1" elementType="O" x3="1.056552" y3="0.077968" z3="0.039285" />
<atom id="a2" elementType="H" x3="2.024439" y3="0.038981" z3="0.014068" />
<atom id="a3" elementType="H" x3="0.777703" y3="-0.701244" z3="-0.464731" />
</atomArray>
<bondArray>
<bond atomRefs2="a1 a3" order="1" />
<bond atomRefs2="a1 a2" order="1" />
</bondArray>
<propertyList>
<property dictRef="me:ZPE">
<scalar units="kJ/mol">0</scalar>
</property>
<property dictRef="me:vibFreqs">
<array units="cm-1">1597.1544 3888.4559 4014.3888</array>
</property>
<property dictRef="me:spinMultiplicity">
<scalar units="cm-1">1</scalar>
</property>
</propertyList>
</molecule>
<molecule spinMultiplicity="2" id="COC(=O)O[O]">
<atomArray>
<atom id="a1" elementType="C" x3="0.932820" y3="-0.066430" z3="0.060280"/>
<atom id="a2" elementType="O" x3="0.488990" y3="-1.427460" z3="-0.092380"/>
<atom id="a3" elementType="H" x3="2.018370" y3="-0.132990" z3="0.069770"/>
<atom id="a4" elementType="H" x3="0.567980" y3="0.351710" z3="0.999030"/>
<atom id="a5" elementType="H" x3="0.595830" y3="0.541120" z3="-0.780580"/>
<atom id="a6" elementType="C" x3="-0.793140" y3="-1.733770" z3="-0.117310"/>
<atom id="a7" elementType="O" x3="-1.262120" y3="-2.813710" z3="-0.232530"/>

<atom id="a8" elementType="O" x3="-1.565260" y3="-0.542600" z3="0.018530"/>
<atom id="a9" elementType="O" x3="-2.852970" y3="-0.777950" z3="0.000780"/>
</atomArray>
<bondArray>
<bond atomRefs2="a5 a1" order="1" />
<bond atomRefs2="a7 a6" order="2" />
<bond atomRefs2="a6 a2" order="1" id="bond62" />
<bond atomRefs2="a6 a8" order="1" id="bond68" />
<bond atomRefs2="a2 a1" order="1" id="bond21" />
<bond atomRefs2="a9 a8" order="1" />
<bond atomRefs2="a1 a3" order="1" />
<bond atomRefs2="a1 a4" order="1" />
</bondArray>
<propertyList>
<property dictRef="me:ZPE">
<scalar units="kJ/mol">-229.9</scalar>
</property>
<property dictRef="me:vibFreqs">
<array units="cm-1">106.5935 145.3134 199.9123 265.2836 376.8631 562.3774 660.9103 730.4077 858.0242 1083.7241 1184.3073 1197.4676 1250.8514 1316.7485 1499.6391 1504.755 1510.897 1989.8072 3104.5272 3199.2145 3215.8126</array>
</property>
<property dictRef="me:spinMultiplicity">
<scalar units="cm-1">2</scalar>
</property>

<property dictRef="me:hessian">
<matrix matrixType="squareSymmetricLT" rows="27" units="Hartree/Bohr2">
0.62422814 -0.05732978 0.47409784 -0.00714180 -0.01482582 0.60948651 -0.08276285 -0.04103723 -0.00477239 0.53822431 -0.01574765 -0.21316052 -0.01810140 0.15007820 0.43303303 -0.00190462 -0.01749297 -0.05896896 0.01431181 0.04077391 0.06806409 -0.33527703 0.02284274 -0.00188669 -0.01136072 -0.00561133 -0.00068519 0.35847375 0.03810428 -0.04797452 0.00108682 -0.04535830 -0.01154764 -0.00169824 -0.01935950 0.06307598 -0.00030190 0.00081784 -0.05335092 -0.00502264 -0.00130252 0.00071541 0.00267295 0.00124358 0.04739318 -0.08810752 0.03854262 0.08933222 0.00215291 -0.01009418 -0.01306301 -0.00707657 0.01075829 0.02801925 0.08764763 0.03250291 -0.08340929 -0.07984978 0.00109704 -0.02798902 -0.02931923 0.00149448 0.00102185 -0.00118546 -0.03379507 0.10077953 0.08393455 -0.08666612 -0.26615182 -0.00402229 -0.00353729 0.00363200 -0.00107078 0.00218599 0.00302001 -0.09174243 0.09691148 0.27744263 -0.08149066 0.05166897 -0.07350364 0.00170578 -0.01265048 0.01055583 -0.00633606 0.01647918 -0.02545226 0.00423985 -0.00624027 0.01003873 0.07996270 0.04518039 -0.12501692 0.11150815 -0.00078945 -0.03353553 0.02136084 0.00126355 0.00110696 0.00105629 -0.00368944 0.00936987 -0.00891714 -0.04737779 0.14825223 -0.06971069 0.11824043 -0.22675384 0.00382129 -0.00462323 0.00948190 0.00111753 -0.00200973 0.00193299 -0.01035686 0.01472333 -0.01837832 0.07665366 -0.12618764 0.23306752 -0.02771223 -0.03408127 -0.00419797 -0.33578915 -0.06759972 -0.00579444 0.00282315 0.00177526 0.00030148 0.00393881 0.00808302 0.00325574 0.00388891 0.00852081 -0.00121478 0.71941814 -0.03829017 -0.00604529 0.00064817 -0.03009969 -

0.12724694 -0.00817848 0.00037573 -0.00563055 -0.00053840 -0.00078865 0.00029073 -
0.00134444 -0.00133671 -0.00058921 0.00112165 0.33442918 1.01257978 -0.00448384
0.00059641 -0.00929535 -0.00137719 -0.00773441 -0.05534926 -0.00003758 -0.00079334 -
0.00078457 -0.00238302 -0.00260225 0.00002738 0.00219089 0.00242042 0.00087506
0.03248829 0.08763025 0.20526484 -0.00093964 0.01019826 0.00132508 -0.04790498 -
0.07439934 -0.00781156 -0.00081653 -0.00260748 -0.00029521 -0.00091507 -0.00177966 -
0.00069066 -0.00094357 -0.00192309 0.00022335 -0.21690368 -0.28294187 -0.02944029
0.25208905 0.00167133 0.00709967 0.00013372 -0.05550250 -0.02666491 -0.00446245 -
0.00083386 -0.00122121 -0.00017060 -0.00053958 -0.00111566 0.00027489 -0.00036778 -
0.00084684 -0.00042948 -0.27528148 -0.73857778 -0.06959949 0.32681886 0.85124698
0.00030225 -0.00005554 0.00656432 -0.00581804 -0.00429642 0.01720655 -0.00008120 -
0.00008380 0.00026813 0.00075693 0.00099909 -0.00015625 -0.00086710 -0.00115614 -
0.00050173 -0.02857029 -0.06954195 -0.08753984 0.03365715 0.08652365 0.04490980 -
0.00882268 0.00783082 0.00071729 -0.06097992 0.03104072 0.00382243 -0.00051768
0.00037890 0.00009810 -0.00135068 -0.00089121 0.00043448 -0.00054091 -0.00070681 -
0.00056781 -0.07689002 -0.00976426 -0.00076839 0.01125997 0.01759902 0.00207927
0.54556909 -0.00396993 -0.00310621 -0.00032246 0.01239186 0.01159411 0.00005598
0.00005131 0.00064315 0.00000901 -0.00033786 0.00083799 0.00048090 -0.00024245
0.00091526 -0.00032352 -0.01190601 -0.14825388 -0.01179760 0.01796567 -0.09323821 -
0.01260946 0.05646452 0.30442906 -0.00044545 -0.00042174 -0.00091782 0.00181597 -
0.00018892 0.01145414 0.00000215 0.00001871 0.00069495 -0.00064832 0.00019980
0.00046806 0.00045344 -0.00003607 0.00032594 -0.00069803 -0.01141068 -0.04983260
0.00195121 -0.01272352 0.01944690 0.00253127 0.03214175 0.02235629 0.00088446
0.00136485 0.00012789 -0.00328537 0.00498378 0.00056875 0.00008769 -0.00017063 -
0.00001977 -0.00052936 -0.00047125 -0.00013732 -0.00048605 -0.00047817 0.00003429 -
0.07277393 0.02841644 0.00381111 0.00507445 -0.01356401 -0.00145896 -0.40772715 -
0.07041711 -0.00496224 0.47875526 -0.00212137 -0.00248476 -0.00027740 0.00922007 -
0.00448258 -0.00103935 -0.00022312 0.00052598 0.00007027 -0.00005614 0.00021398
0.00061173 0.00006733 0.00034418 -0.00051181 0.03606020 0.01347315 0.00188001
0.00866865 0.00331798 0.00022058 -0.10195170 -0.07382127 -0.00757933 0.05033609
0.06291335 -0.00024850 -0.00019250 -0.00061212 0.00106347 -0.00098972 0.00376413 -
0.00003120 0.00005003 0.00011081 0.00008524 0.00012303 0.00009631 -0.00006955 -
0.00004871 -0.00004951 0.00442998 0.00161387 -0.00336565 0.00108094 0.00045329 -
0.00019788 -0.00834664 -0.00763459 -0.00399586 0.00203626 0.00662530 0.00424976
</matrix>
</property>
<property dictRef="me:sigma" default="true">
<scalar>5.0</scalar>
</property>
<property dictRef="me:epsilon" default="true">
<scalar>50.0</scalar>
</property>
</propertyList>
<me:energyTransferModel xsi:type="me:ExponentialDown">
<me:deltaEDown >57</me:deltaEDown>
<me:deltaEDownTExponent referenceTemperature="298"
>0.0</me:deltaEDownTExponent>
</me:energyTransferModel>
<me:DOSCMethod xsi:type="me:ClassicalCoupledRotors">
<me:MCPoints>10000</me:MCPoints>

```
    <me:RotorArray>
   <me:Rotor>
    <bondRef>bond68</bondRef>
    <me:HinderedRotorPotential format="numerical" units="kJ/mol" expansionSize="10"
UseSineTerms="yes">
        <me:PotentialPoint  angle="    0    " potential="   0    "/>
        <me:PotentialPoint  angle="    10    " potential="   0.656375    "/>
        <me:PotentialPoint  angle="    20    " potential="   2.546735    "/>
        <me:PotentialPoint  angle="    30    " potential="   5.749845    "/>
        <me:PotentialPoint  angle="    40    " potential="   9.60933    "/>
        <me:PotentialPoint  angle="    50    " potential="   13.783875    "/>
        <me:PotentialPoint  angle="    60    " potential="   17.249535    "/>
        <me:PotentialPoint  angle="    70    " potential="   19.69125    "/>
        <me:PotentialPoint  angle="    80    " potential="   20.74145    "/>
        <me:PotentialPoint  angle="    90    " potential="   20.032565    "/>
        <me:PotentialPoint  angle="    100    " potential="   18.48352    "/>
        <me:PotentialPoint  angle="    110    " potential="   16.8032    "/>
        <me:PotentialPoint  angle="    120    " potential="   15.38543    "/>
        <me:PotentialPoint  angle="    130    " potential="   14.519015    "/>
        <me:PotentialPoint  angle="    140    " potential="   14.49276    "/>
        <me:PotentialPoint  angle="    150    " potential="   14.939095    "/>
        <me:PotentialPoint  angle="    160    " potential="   16.251845    "/>
        <me:PotentialPoint  angle="    170    " potential="   18.247225    "/>
        <me:PotentialPoint  angle="    180    " potential="   20.715195    "/>
        <me:PotentialPoint  angle="    190    " potential="   23.68201    "/>
        <me:PotentialPoint  angle="    200    " potential="   26.858865    "/>
        <me:PotentialPoint  angle="    210    " potential="   14.991605    "/>
        <me:PotentialPoint  angle="    220    " potential="   14.361485    "/>
        <me:PotentialPoint  angle="    230    " potential="   14.571525    "/>
        <me:PotentialPoint  angle="    240    " potential="   15.28041    "/>
        <me:PotentialPoint  angle="    250    " potential="   16.75069    "/>
        <me:PotentialPoint  angle="    260    " potential="   18.457265    "/>
        <me:PotentialPoint  angle="    270    " potential="   20.16384    "/>
        <me:PotentialPoint  angle="    280    " potential="   20.715195    "/>
        <me:PotentialPoint  angle="    290    " potential="   19.770015    "/>
        <me:PotentialPoint  angle="    300    " potential="   17.3283    "/>
        <me:PotentialPoint  angle="    310    " potential="   13.70511    "/>
        <me:PotentialPoint  angle="    320    " potential="   9.530565    "/>
        <me:PotentialPoint  angle="    330    " potential="   5.72359    "/>
        <me:PotentialPoint  angle="    340    " potential="   2.599245    "/>
        <me:PotentialPoint  angle="    350    " potential="   0.68263    "/>
        <me:PotentialPoint  angle="    360    " potential="   0    "/>
    </me:HinderedRotorPotential>

   </me:Rotor>
   <me:Rotor>
    <bondRef>bond62</bondRef>
    <me:HinderedRotorPotential format="numerical" units="kJ/mol" expansionSize="10"
UseSineTerms="yes">
        <me:PotentialPoint  angle="    0    " potential="   0    "/>
```

<me:PotentialPoint angle=" 10 " potential=" 1.36526 "/>
<me:PotentialPoint angle=" 20 " potential=" 5.172235 "/>
<me:PotentialPoint angle=" 30 " potential=" 11.000845 "/>
<me:PotentialPoint angle=" 40 " potential=" 18.247225 "/>
<me:PotentialPoint angle=" 50 " potential=" 25.966195 "/>
<me:PotentialPoint angle=" 60 " potential=" 33.055045 "/>
<me:PotentialPoint angle=" 70 " potential=" 38.8574 "/>
<me:PotentialPoint angle=" 80 " potential=" 42.58561 "/>
<me:PotentialPoint angle=" 90 " potential=" 43.924615 "/>
<me:PotentialPoint angle=" 100 " potential=" 42.874415 "/>
<me:PotentialPoint angle=" 110 " potential=" 40.06513 "/>
<me:PotentialPoint angle=" 120 " potential=" 36.28441 "/>
<me:PotentialPoint angle=" 130 " potential=" 32.057355 "/>
<me:PotentialPoint angle=" 140 " potential=" 27.804045 "/>
<me:PotentialPoint angle=" 150 " potential=" 24.180855 "/>
<me:PotentialPoint angle=" 160 " potential=" 21.47659 "/>
<me:PotentialPoint angle=" 170 " potential=" 19.875035 "/>
<me:PotentialPoint angle=" 180 " potential=" 19.37619 "/>
<me:PotentialPoint angle=" 190 " potential=" 19.402445 "/>
<me:PotentialPoint angle=" 200 " potential=" 19.927545 "/>
<me:PotentialPoint angle=" 210 " potential=" 21.5291 "/>
<me:PotentialPoint angle=" 220 " potential=" 24.1546 "/>
<me:PotentialPoint angle=" 230 " potential=" 27.804045 "/>
<me:PotentialPoint angle=" 240 " potential=" 31.97859 "/>
<me:PotentialPoint angle=" 250 " potential=" 36.2319 "/>
<me:PotentialPoint angle=" 260 " potential=" 40.01262 "/>
<me:PotentialPoint angle=" 270 " potential=" 42.821905 "/>
<me:PotentialPoint angle=" 280 " potential=" 43.95087 "/>
<me:PotentialPoint angle=" 290 " potential=" 42.63812 "/>
<me:PotentialPoint angle=" 300 " potential=" 38.8574 "/>
<me:PotentialPoint angle=" 310 " potential=" 33.055045 "/>
<me:PotentialPoint angle=" 320 " potential=" 25.808665 "/>
<me:PotentialPoint angle=" 330 " potential=" 18.194715 "/>
<me:PotentialPoint angle=" 340 " potential=" 10.97459 "/>
<me:PotentialPoint angle=" 350 " potential=" 5.19849 "/>
<me:PotentialPoint angle=" 360 " potential=" 0 "/>
</me:HinderedRotorPotential>

</me:Rotor>
<me:Rotor>
<bondRef>bond21</bondRef>
<me:HinderedRotorPotential format="numerical" units="kJ/mol" expansionSize="10" UseSineTerms="yes">
<me:PotentialPoint angle=" 0 " potential=" 0 "/>
<me:PotentialPoint angle=" 10 " potential=" 0.5251 "/>
<me:PotentialPoint angle=" 20 " potential=" 1.83785 "/>
<me:PotentialPoint angle=" 30 " potential=" 3.62319 "/>
<me:PotentialPoint angle=" 40 " potential=" 5.382275 "/>
<me:PotentialPoint angle=" 50 " potential=" 6.72128 "/>
<me:PotentialPoint angle=" 60 " potential=" 7.272635 "/>

<me:PotentialPoint angle=" 70 " potential=" 6.51124 "/>
<me:PotentialPoint angle=" 80 " potential=" 5.067215 "/>
<me:PotentialPoint angle=" 90 " potential=" 3.25562 "/>
<me:PotentialPoint angle=" 100 " potential=" 1.5753 "/>
<me:PotentialPoint angle=" 110 " potential=" 0.31506 "/>
<me:PotentialPoint angle=" 120 " potential=" -0.078765 "/>
<me:PotentialPoint angle=" 130 " potential=" 0.47259 "/>
<me:PotentialPoint angle=" 140 " potential=" 1.811595 "/>
<me:PotentialPoint angle=" 150 " potential=" 3.62319 "/>
<me:PotentialPoint angle=" 160 " potential=" 5.35602 "/>
<me:PotentialPoint angle=" 170 " potential=" 6.61626 "/>
<me:PotentialPoint angle=" 180 " potential=" 7.220125 "/>
<me:PotentialPoint angle=" 190 " potential=" 6.72128 "/>
<me:PotentialPoint angle=" 200 " potential=" 5.329765 "/>
<me:PotentialPoint angle=" 210 " potential=" 3.62319 "/>
<me:PotentialPoint angle=" 220 " potential=" 1.811595 "/>
<me:PotentialPoint angle=" 230 " potential=" 0.47259 "/>
<me:PotentialPoint angle=" 240 " potential=" -0.05251 "/>
<me:PotentialPoint angle=" 250 " potential=" 0.26255 "/>
<me:PotentialPoint angle=" 260 " potential=" 1.444025 "/>
<me:PotentialPoint angle=" 270 " potential=" 3.229365 "/>
<me:PotentialPoint angle=" 280 " potential=" 5.119725 "/>
<me:PotentialPoint angle=" 290 " potential=" 6.61626 "/>
<me:PotentialPoint angle=" 300 " potential=" 7.325145 "/>
<me:PotentialPoint angle=" 310 " potential=" 6.852555 "/>
<me:PotentialPoint angle=" 320 " potential=" 5.40853 "/>
<me:PotentialPoint angle=" 330 " potential=" 3.596935 "/>
<me:PotentialPoint angle=" 340 " potential=" 1.78534 "/>
<me:PotentialPoint angle=" 350 " potential=" 0.47259 "/>
<me:PotentialPoint angle=" 360 " potential=" 0 "/>
</me:HinderedRotorPotential>

</me:Rotor>
</me:RotorArray>
</me:DOSCMethod>
</molecule>
<molecule spinMultiplicity="2" id="TS_[O]OCOC=O_[O]COOC=O">
<atomArray>
<atom id="a1" elementType="C" x3="-1.108290" y3="-0.113830" z3="0.624610" />
<atom id="a2" elementType="O" x3="0.007500" y3="-0.069450" z3="1.154810" />
<atom id="a3" elementType="H" x3="-1.834380" y3="-0.865660" z3="0.952060" />
<atom id="a4" elementType="H" x3="-1.445270" y3="0.690500" z3="-0.041390" />
<atom id="a5" elementType="C" x3="1.131260" y3="0.523330" z3="-0.223060" />
<atom id="a6" elementType="O" x3="0.711310" y3="1.506810" z3="-0.736760" />
<atom id="a7" elementType="H" x3="2.056590" y3="0.338530" z3="0.328330" />
<atom id="a8" elementType="O" x3="-0.388240" y3="-1.157370" z3="-1.068390" />
<atom id="a9" elementType="O" x3="0.849920" y3="-0.833990" z3="-0.979010" />
</atomArray>
<bondArray>
<bond atomRefs2="a9 a8" order="1" />

<bond atomRefs2="a9 a5" order="1" id="bond95" />
<bond atomRefs2="a6 a5" order="2" />
<bond atomRefs2="a5 a7" order="1" />
<bond atomRefs2="a4 a1" order="1" />
<bond atomRefs2="a1 a3" order="1" />
<bond atomRefs2="a1 a2" order="2" id="bond12" />
<bond atomRefs2="a5 a2" order="1" />
<bond atomRefs2="a9 a5" order="1" />
</bondArray>
<propertyList>
<property dictRef="me:ZPE">
<scalar units="kJ/mol" >-187.5214172</scalar>
</property>
<property dictRef="me:vibFreqs">
<array units="cm-1">186.7032 225.7017 330.3951 426.5634 454.5216 520.4625 574.3158 764.7289 788.1861 1064.2054 1197.0614 1248.467 1277.6991 1369.2927 1481.7497 1667.1965 1863.9251 3101.1708 3194.8489 3209.1309</array>
</property>
<property dictRef="me:spinMultiplicity">
<scalar units="cm-1">2</scalar>
</property>
<property dictRef="me:hessian">
<matrix matrixType="squareSymmetricLT" rows="27" units="Hartree/Bohr2">0.85255403 0.10351310 0.49924974 0.17507927 -0.25360387 0.35557693 -0.53451737 -0.00254079 -0.16536923 0.57610710 -0.02595494 -0.08419788 0.02986514 0.02160487 0.10565791 -0.22355744 0.02208373 -0.18652460 0.26370192 -0.01042765 0.24646391 -0.15666691 -0.11606107 0.05650515 -0.03559883 -0.02363863 0.00503899 0.18296165 -0.11803903 -0.19394909 0.08046072 -0.01133806 0.00775791 -0.00120344 0.13574512 0.18644363 0.06175288 0.07590861 -0.08919553 -0.02140738 -0.01430976 0.01809402 -0.05556601 -0.07970854 0.07508667 -0.07972411 0.04288169 -0.04649277 -0.01810454 0.02455975 -0.02616178 0.00938296 -0.01502882 0.00763404 0.09046094 0.05549698 -0.21146340 0.12893059 0.00083215 0.01750700 0.00104994 0.00451832 -0.00502372 0.01199238 -0.06231051 0.20926860 -0.04213724 0.12881534 -0.14028412 -0.02642967 0.01444835 -0.00580898 -0.00491445 0.00875669 0.00429415 0.06240893 -0.13725306 0.15037152 -0.04970021 -0.02165246 -0.01070517 0.00121933 0.01836714 -0.03381208 0.00128175 0.00266531 0.00156064 -0.00052871 -0.00145359 0.00243993 0.61815471 -0.01698827 0.01499174 0.00532578 0.01393360 -0.03568971 -0.00544296 0.00099597 0.00030703 0.00506764 0.00811303 -0.00236591 -0.00440749 -0.28886010 0.63883392 0.04672670 0.02062494 0.07271606 -0.09546812 -0.01538672 0.00695190 -0.00682322 -0.00644911 -0.00950508 -0.00596049 0.00094406 -0.00240370 0.33414805 -0.32135429 0.33808856 -0.00309146 0.00433605 0.00109477 0.01169685 -0.01623309 0.00810724 0.00132143 0.00009170 0.00246572 0.00261233 -0.00177818 0.00044285 -0.21959860 0.22431989 -0.14925934 0.17955160 0.01183421 -0.00959986 0.00420061 -0.02306735 -0.01024152 0.02811478 -0.00205954 0.00084356 -0.00227036 -0.00394766 -0.00050062 0.00035030 0.26193073 -0.57351130 0.30235732 -0.24900945 0.67720487 -0.01228524 -0.00203072 -0.01614141 0.02814845 0.02889497 -0.02327085 0.00202000 0.00045283 0.00222545 0.00224893 -0.00010065 -0.00046326 -0.16472901 0.28129412 -0.23821726 0.14566455 -0.33330401 0.24888173 0.00120272 -0.00128635 0.00405762 -0.01594722 -0.00353323 0.00296249 -0.00085094 0.00011004 -0.00049603 0.00011593 0.00012504 -0.00047974 -0.25909377 0.04548112 -0.12730538 0.00401237

0.00493798 -0.00736447 0.27173306 -0.00137080 -0.00083573 -0.00239118 -0.00168209 0.00273327 -0.00048356 -0.00011649 0.00005314 -0.00005479 -0.00046654 -0.00020791 0.00073073 0.04234406 -0.05248018 0.02193345 0.02931374 -0.00648405 0.02228132 -0.04482531 0.06645643 -0.00137320 0.00027171 -0.00837282 0.02423281 0.00279322 -0.00177057 0.00098979 0.00047098 0.00115060 0.00019360 -0.00005454 0.00043369 -0.12272219 0.02372484 -0.10699726 -0.01616834 0.01079121 0.00327944 0.13271693 -0.02876924 0.11560138 -0.00327340 0.00180652 -0.00240383 -0.00330557 -0.00275619 0.00421818 -0.00257222 0.00400270 0.00440488 -0.00155064 0.00188520 0.00349118 -0.02440689 -0.01039445 -0.01849194 0.00730180 -0.00334834 0.00561535 -0.00300344 -0.00569324 -0.00108692 0.54213367 0.00013216 -0.01050242 0.03281720 -0.02879715 -0.00822966 -0.01636656 0.00010200 0.00306808 0.00295102 0.00772850 -0.00777901 -0.01470699 -0.03303653 -0.00323553 -0.02488398 0.00570268 0.00284394 0.00527353 -0.00092938 -0.00520062 -0.00221377 0.13736789 0.09512433 -0.00868261 0.02204655 0.03541925 -0.04096981 -0.02906052 -0.03361520 0.00333177 -0.00433551 -0.00401581 0.00599506 -0.00506547 -0.00480535 -0.01279381 -0.00510169 -0.03003593 0.00223437 0.00033645 0.00735264 -0.00113968 -0.00371404 -0.00077985 0.03918061 0.04403484 0.05936718 -0.02678328 -0.01099669 -0.01176582 0.01845025 0.00758431 -0.00049752 0.00074110 0.00179104 -0.00034873 -0.00266417 0.00268460 0.00517821 -0.06732761 0.02339922 0.02243373 0.01619367 0.00272941 0.00068144 0.00183130 -0.01750333 -0.01678248 -0.51132330 -0.08827018 0.01284409 0.57088203 -0.00862343 -0.00369311 -0.02560499 0.03105482 0.00470268 -0.01732427 0.00051432 0.00049946 0.00042379 -0.00152943 0.00056497 0.00326612 0.01969544 0.01314993 0.02221432 0.00325665 -0.08055502 -0.00276139 -0.00007990 -0.00403435 -0.00701440 -0.12287009 -0.06608911 -0.01914060 0.07858161 0.13545455 0.00447687 -0.01411629 -0.02319376 0.03356103 -0.00681702 -0.02051963 -0.00058202 0.00155538 0.00186553 0.00013447 -0.00044325 -0.00133396 0.00661363 0.02089404 -0.03059730 0.00541819 -0.01057630 0.01635352 -0.00295173 -0.00953269 -0.00254461 -0.03492751 -0.02690528 -0.02888693 -0.01174292 0.04594142 0.08885714</matrix>
</property>
<property dictRef="me:imFreq">
<scalar units="cm-1">383.5768</scalar>
</property>
<property dictRef="me:vibFreqsFromHessian">
<scalar calculated="20220120_074051" units="cm-1">186.711 225.7 330.392 426.548 454.508 520.472 574.311 764.723 788.145 1064.2 1197.06 1248.46 1277.69 1369.26 1481.74 1667.15 1863.87 3101.15 3194.82 3209.11 </scalar>
</property>
<property dictRef="me:frequenciesScaleFactor" default="true">
<scalar>0.9522</scalar>
</property>
<property dictRef="me:symmetryNumber" default="true">
<scalar>1</scalar>
</property>
</propertyList>
<me:DOSCMethod default="true" name="ClassicalRotors" />
</molecule>
<molecule spinMultiplicity="2" id="[CH]=O">
<atomArray>
<atom id="a1" elementType="C" spinMultiplicity="2" x3="1.259096" y3="-0.298049" z3="0.025187" />

<atom id="a2" elementType="H" x3="0.312085" y3="-0.754420" z3="0.194861" />
<atom id="a3" elementType="O" x3="2.308569" y3="0.199797" z3="-0.159908" />
</atomArray>
<bondArray>
<bond atomRefs2="a2 a1" order="1" />
<bond atomRefs2="a1 a3" order="2" />
</bondArray>
<propertyList>
<property dictRef="me:ZPE">
<scalar units="kJ/mol">6.679130035</scalar>
</property>
<property dictRef="me:vibFreqs">
<array units="cm-1">1102.4018 2010.5681 2753.0615</array>
</property>
<property dictRef="me:spinMultiplicity">
<scalar units="cm-1">2</scalar>
</property>
<property dictRef="me:hessian">
<matrix matrixType="squareSymmetricLT" rows="42" units="Hartree/Bohr2">1.08805815 -0.15453323 0.26876927 -0.00 220894 0.00338558 0.00305102 -0.06409677 0.06154072 0.00091656 0.09476 530 0.06777099 -0.21506451 - 0.00273627 -0.10113992 0.21130020 0.001021 48 -0.00273897 -0.00149090 -0.00143315 0.00272380 0.00086893 -1.023961 37 0.09299251 0.00129238 -0.03066853 0.03336893 0.00041167 1.05462990 0.08676223 -0.05370476 -0.00064931 0.03959920 0.00376430 0.00001517 -0 .12636143 0.04994046 0.00118746 -0.00064661 -0.00156012 0.00051659 0.0 0001247 0.00062197 -0.00170405 0.00063414 0.00093815</matrix>
</property>
</propertyList>
</molecule>
<molecule spinMultiplicity="2" id="CH_post_comp">
<atomArray>
<atom id="a1" elementType="C" x3="0.552683" y3="0.263940" z3="0.434212" />
<atom id="a2" elementType="O" x3="-0.044633" y3="1.480057" z3="0.066978" />
<atom id="a3" elementType="H" x3="0.152033" y3="-0.277587" z3="1.298392" />
<atom id="a4" elementType="H" x3="1.618697" y3="0.425009" z3="0.623860" />
<atom id="a5" elementType="H" x3="0.339592" y3="-0.399117" z3="-0.412354" />
<atom id="a6" elementType="C" spinMultiplicity="2" x3="0.356285" y3="2.503839" z3="0.823160" />
<atom id="a7" elementType="H" x3="2.049784" y3="-0.634206" z3="-1.071990" />
<atom id="a8" elementType="O" x3="0.644968" y3="3.432490" z3="1.461920" />
<atom id="a9" elementType="O" x3="2.764438" y3="-1.143382" z3="-0.659535" />
<atom id="a10" elementType="H" x3="2.573077" y3="-1.170777" z3="0.291758" />
</atomArray>
<bondArray>
<bond atomRefs2="a7 a9" order="1" />
<bond atomRefs2="a9 a10" order="1" />
<bond atomRefs2="a3 a1" order="1" />
<bond atomRefs2="a2 a6" order="1" />
<bond atomRefs2="a2 a1" order="1" id="bond21" />
<bond atomRefs2="a5 a1" order="1" />

<bond atomRefs2="a6 a8" order="2" />
<bond atomRefs2="a1 a4" order="1" />
<bond atomRefs2="a8 a2" order="1" />
</bondArray>
<propertyList>
<property dictRef="me:ZPE">
<scalar units="kJ/mol">-90.80641228</scalar>
</property>
<property dictRef="me:vibFreqs">
<array units="cm-1">37.8172 113.8875 133.6182 163.4778 231.0168 296.4605 321.0005 338.4072 382.117 772.2143 940.1757 1168.0863 1191.6258 1228.5626 1468.9676 1499.6666 1503.6724 1606.0184 1922.8759 3096.3451 3190.4416 3235.3533 3866.2348 4000.8341</array>
</property>
<property dictRef="me:spinMultiplicity">
<scalar units="cm-1">2</scalar>
</property>
<!--><property dictRef="me:hessian">
<matrix matrixType="squareSymmetricLT" rows="42" units="Hartree/Bohr2"></matrix>
</property>-->
<property dictRef="me:sigma" default="true">
<scalar>5.0</scalar>
</property>
<property dictRef="me:epsilon" default="true">
<scalar>50.0</scalar>
</property>
<property dictRef="me:frequenciesScaleFactor" default="true">
<scalar>0.9522</scalar>
</property>
<property dictRef="me:symmetryNumber" default="true">
<scalar>1</scalar>
</property>
</propertyList>
<me:DOSCMethod default="true" name="ClassicalRotors" />
<me:DistributionCalcMethod default="true" name="Boltzmann" />
<me:energyTransferModel name="ExponentialDown" default="true" />
<me:deltaEDown default="NEEDS TO BE CHECKED**">130.0</me:deltaEDown>
</molecule>
<molecule spinMultiplicity="2" id="TS2">
<atomArray>
<atom id="a1" elementType="C" x3="0.740880" y3="0.322330" z3="-0.036170" />
<atom id="a2" elementType="O" x3="1.391840" y3="-0.405740" z3="0.625810" />
<atom id="a3" elementType="O" x3="-0.344550" y3="0.629080" z3="-0.499910" />
<atom id="a4" elementType="C" x3="-1.790850" y3="-0.468350" z3="-0.055240" />
<atom id="a5" elementType="H" x3="-1.335160" y3="-1.220120" z3="0.579420" />
<atom id="a6" elementType="H" x3="-2.086910" y3="-0.782450" z3="-1.047370" />
<atom id="a7" elementType="H" x3="-2.424300" y3="0.261430" z3="0.431380" />
</atomArray>
<bondArray>
<bond atomRefs2="a6 a4" order="1" />

<bond atomRefs2="a3 a4" order="1" id="bond34" />
<bond atomRefs2="a3 a1" order="1" id="bond31" />
<bond atomRefs2="a4 a7" order="1" />
<bond atomRefs2="a4 a5" order="1" />
<bond atomRefs2="a1 a2" order="2" />
<bond atomRefs2="a5 a4" order="1" />
</bondArray>
<propertyList>
<property dictRef="me:ZPE">
<scalar units="kJ/mol">-21.95375197</scalar>
</property>
<property dictRef="me:vibFreqs">
<array units="cm-1">36.437 175.9266 515.5667 645.7669 750.9992 754.8561
1048.4003 1209.1695 1442.7415 1447.1205 2115.6487 3144.8593 3307.2109
3321.0552</array>
</property>
<property dictRef="me:spinMultiplicity">
<scalar units="cm-1">2</scalar>
</property>
<property dictRef="me:hessian">
<matrix matrixType="squareSymmetricLT" rows="21"
units="Hartree/Bohr2">0.67250227 -0.46944386 0.46955115 0.46556772 -0.37983777
0.45683543 -0.28036316 0.27854064 -0.25126023 0.34825881 0.35212236 -0.35615731
0.30322397 -0.33522808 0.35503363 -0.30317197 0.28645747 -0.31126076 0.31143664 -
0.31317356 0.31130870 -0.23049298 0.26493463 -0.22591809 -0.09549647 -0.06932456
0.02050636 0.16358988 0.22668185 -0.08278831 0.06821879 0.03986964 -0.03334285
0.05317357 -0.32604796 0.10869517 -0.19893029 0.07693584 -0.15891252 -0.05652954
0.02829167 -0.00369064 0.25567496 -0.11498171 0.19040103 -0.16283253 -0.06492393
0.00512146 0.02732555 0.04817741 -0.02598112 0.18605331 0.07258230 -0.00320258
0.23680905 -0.11074998 -0.02343072 0.00237618 0.01664761 0.03090856 -0.02352753
0.15047259 0.01399476 0.00704545 -0.28401473 0.42679887 0.03745256 0.01281628
0.01520118 -0.00373708 -0.01634160 0.00285969 -0.05815362 -0.01070204 -0.03031996
0.11075598 0.07871160 0.59980025 0.00176221 -0.00420827 0.00311071 -0.00044260
0.00355412 -0.00245672 -0.00156467 -0.01114690 0.00762336 -0.08889396 0.09872995 -
0.07732100 0.09159790 0.00109786 -0.00126168 0.00194217 -0.00048078 0.00136966 -
0.00154426 -0.00210895 -0.00733033 0.00434948 0.10408557 -0.19234778 0.12037551 -
0.10384585 0.19670178 -0.00063201 0.00073437 0.00047477 0.00038955 -0.00062564 -
0.00013416 0.00124249 0.00228649 -0.00181764 -0.08110171 0.11915346 -0.15808151
0.08358940 -0.12970338 0.15715868 -0.00070230 -0.00163448 0.00306039 0.00053575 -
0.00022260 -0.00117036 -0.00980920 -0.00528095 -0.00852277 -0.05761176 -0.01063136 -
0.08617374 0.00005587 -0.00428653 -0.00949643 0.05825590 0.00020239 -0.00167676
0.00318513 0.00062955 0.00022509 -0.00133188 -0.00722558 -0.00346979 -0.00597882 -
0.00995590 -0.07128600 -0.08854788 0.00645089 0.01054187 0.01943373 0.01651871
0.06730342 0.00050336 0.00150281 -0.00203331 -0.00008193 -0.00079209 0.00115029
0.00351916 0.00254969 0.00479064 -0.08111035 -0.08486766 -0.31302713 -0.00278256 -
0.00750078 -0.00919316 0.08542844 0.09004410 0.32477906 0.00012649 -0.00326473
0.00031804 0.00018212 0.00092135 0.00083716 -0.01227986 0.00334204 0.00388686 -
0.14084966 0.13954592 0.07717690 -0.00251474 0.00553867 0.00600871 0.00927574 -
0.00662007 -0.00547612 0.14605991 0.00008938 -0.00423637 0.00089152 0.00002142
0.00196324 -0.00005380 -0.01070019 0.00424134 0.00433807 0.13404928 -0.18463769 -

0.09631186 0.01046606 -0.00767353 -0.01127903 0.00553722 -0.00163783 -0.00093608 -
0.13946317 0.19198085 -0.00078938 0.00139099 -0.00030477 -0.00021740 -0.00058276 -
0.00023312 0.00312874 -0.00054480 -0.00045091 0.07551831 -0.09889149 -0.11643251 -
0.01176320 0.01208126 0.01159302 0.01687447 -0.01680438 -0.00646638 -0.08275155
0.10335117 0.11229466</matrix>
</property>
<property dictRef="me:imFreqs">
<scalar units="cm-1">937.967</scalar>
</property>
<property dictRef="me:vibFreqsFromHessian">
<scalar calculated="20220120_074051" units="cm-1">36.4378 175.926 515.565
645.764 750.997 754.853 1048.4 1209.17 1442.74 1447.12 2115.64 3144.85 3307.2 3321.04
</scalar>
</property>
<property dictRef="me:frequenciesScaleFactor" default="true">
<scalar>0.9522</scalar>
</property>
<property dictRef="me:symmetryNumber" default="true">
<scalar>1</scalar>
</property>
</propertyList>
<me:ExtraDOSCMethod xsi:type="me:HinderedRotorQM1D">
<bondRef>bond34</bondRef>
<me:periodicity>3</me:periodicity>
<me:HinderedRotorPotential format="analytical" units="kJ/mol">
<me:PotentialPoint index="0" coefficient="0.1"/>
<me:PotentialPoint index="3" coefficient="0.1"/>
</me:HinderedRotorPotential>

</me:ExtraDOSCMethod>
<me:DOSCMethod default="true" name="ClassicalRotors" />
</molecule>
<molecule spinMultiplicity="4" id="TS3">
<atomArray>
<atom id="a1" elementType="C" x3="0.969220" y3="-0.446350" z3="0.090890" />
<atom id="a2" elementType="O" x3="1.285760" y3="0.697670" z3="-0.038950" />
<atom id="a3" elementType="O" x3="-0.828080" y3="-0.667130" z3="-0.030090" />
<atom id="a4" elementType="C" x3="-1.456020" y3="0.393800" z3="0.014380" />
<atom id="a5" elementType="H" x3="-0.930720" y3="1.343260" z3="-0.146570" />
<atom id="a6" elementType="H" x3="-2.524030" y3="0.376930" z3="0.244490" />
<atom id="a7" elementType="H" x3="1.240460" y3="-1.319840" z3="-0.526070" />
</atomArray>
<bondArray>
<bond atomRefs2="a7 a1" order="1" />
<bond atomRefs2="a5 a4" order="1" />
<bond atomRefs2="a2 a1" order="1" />
<bond atomRefs2="a3 a4" order="1" id="bond34" />
<bond atomRefs2="a3 a1" order="1" id="bond31" />
<bond atomRefs2="a4 a6" order="1" />
</bondArray>

<propertyList>
<property dictRef="me:ZPE">
<scalar units="kJ/mol">54.88091895</scalar>
</property>
<property dictRef="me:vibFreqs">
<array units="cm-1">240.1971 273.4347 381.6285 541.1119 853.3173 1085.8078
1114.8184 1250.4759 1454.5405 1640.034 1822.8385 2990.0565 3068.0646
3193.1603</array>
</property>
<property dictRef="me:spinMultiplicity">
<scalar units="cm-1">2</scalar>
</property>
<property dictRef="me:hessian">
<matrix matrixType="squareSymmetricLT" rows="21"
units="Hartree/Bohr2">0.06701958 0.12847487 0.99808788 -0.13986013 0.05116134
0.16500328 -0.10625852 -0.19055589 0.02467146 0.12203608 -0.20787935 -0.77520858
0.05410964 0.22410628 0.82139980 0.05849314 0.08055436 -0.03919359 -0.02592338 -
0.06033468 0.02765313 0.15293654 -0.04797561 0.11518508 -0.02765527 0.02364436 -
0.04607865 0.08946234 -0.07303758 0.01211084 -0.06814782 -0.02911599 -0.05513960
0.02891702 -0.16217723 0.57530652 0.00044646 0.00149493 -0.02719525 0.00778083 -
0.00524789 0.01066378 -0.04567577 0.03452034 0.04706436 -0.07845344 0.05913035 -
0.03564721 0.00346634 -0.03104052 0.01777725 -0.19166638 0.20808327 0.02062514
0.71586589 0.12283277 -0.04293707 0.07353319 -0.00428069 0.04219524 -0.03184647
0.12428647 -0.47881566 -0.02901354 -0.09077680 0.76710145 0.00005157 0.00129836
0.00845964 -0.00016053 0.00126463 -0.00443340 0.01988923 -0.01745514 -0.05741155 -
0.10132398 -0.02662348 0.16477102 -0.00422056 0.01023941 -0.00574154 -0.00149470 -
0.00717699 0.00326667 0.02563500 0.01455564 -0.00180027 -0.13867560 -0.11131576
0.02467122 0.12306248 -0.00820073 -0.00309267 -0.00238089 -0.00088163 0.00219877
0.00025006 0.00420747 -0.04521700 0.00576942 -0.11228568 -0.23471957 0.03290208
0.11388762 0.27626269 -0.00026561 0.00551946 -0.00486078 0.00140578 -0.00453176 -
0.00031259 -0.00266706 -0.00220786 0.02023715 0.02218194 0.03492727 -0.05569833 -
0.02788327 -0.03767834 0.02842885 0.01068280 -0.00266032 0.00663379 -0.00035838
0.00305422 -0.00214070 -0.02921380 0.02302095 0.00307069 -0.31050210 -0.03120349
0.05712933 -0.00529887 0.00265653 0.00443521 0.33626051 -0.00611043 -0.00768038 -
0.00034724 -0.00028059 0.00641222 -0.00089483 0.04752562 -0.00324887 -0.00754177 -
0.02736228 -0.06113105 0.00912789 -0.01902689 0.00491452 0.00488929 0.00464438
0.06053228 0.00273086 -0.00667899 0.00820633 -0.00206445 0.00645642 -0.00194663
0.00120056 0.00111064 0.00721826 0.06367540 0.00389713 -0.05724457 0.00594939
0.00016847 0.01167417 -0.06726373 -0.00553711 0.03331727 -0.04170641 0.04334719
0.03475855 0.01026444 -0.00470801 -0.00539433 -0.01949843 0.01867094 0.01555292 -
0.00003471 -0.00954250 -0.00025684 0.00099225 0.00061642 0.00279301 -0.00157017
0.00061020 -0.00422804 0.05155303 0.04392046 -0.18128002 -0.10792822 0.00100850 -
0.04185785 -0.01664546 0.01048893 -0.00499625 0.00001851 -0.00574834 0.00830666 -
0.00051434 -0.00116302 -0.00034674 -0.00091807 0.00048772 0.00020129 0.00058343 -
0.04899424 0.21997291 0.07840371 -0.13334946 -0.11041965 -0.00570971 0.00828364
0.00756930 -0.04185341 0.02326281 -0.00057675 0.01271146 -0.02487412 0.00155719
0.00153780 0.00096920 0.00053153 -0.00186458 0.00030378 -0.00122484 -0.04322527
0.12540414 0.10256322</matrix>
</property>
<property dictRef="me:imFreqs">

<scalar units="cm-1">737.1306</scalar>
</property>
<property dictRef="me:vibFreqsFromHessian">
<scalar calculated="20220120_074051" units="cm-1">240.196 273.434 381.627 541.11 853.314 1085.8 1114.81 1250.47 1454.54 1640.03 1822.83 2990.05 3068.05 3193.15 </scalar>
</property>
<property dictRef="me:frequenciesScaleFactor" default="true">
<scalar>0.9522</scalar>
</property>
<property dictRef="me:symmetryNumber" default="true">
<scalar>1</scalar>
</property>
</propertyList>
<me:ExtraDOSCMethod xsi:type="me:HinderedRotorQM1D">
<bondRef>bond31</bondRef>
<me:HinderedRotorPotential format="numerical" units="kJ/mol" expansionSize="10" UseSineTerms="yes">
<me:PotentialPoint angle="0" potential="0.0" />
<me:PotentialPoint angle="5.0" potential="0.6571120122332232" />
<me:PotentialPoint angle="10.0" potential="1.51485180491361" />
<me:PotentialPoint angle="15.0" potential="2.542757723569915" />
<me:PotentialPoint angle="20.0" potential="3.728916553539967" />
<me:PotentialPoint angle="25.0" potential="5.090699750019351" />
<me:PotentialPoint angle="30.0" potential="6.561288703727224" />
<me:PotentialPoint angle="35.0" potential="8.207640795188647" />
<me:PotentialPoint angle="40.0" potential="9.981809406053785" />
<me:PotentialPoint angle="45.0" potential="11.902527072698529" />
<me:PotentialPoint angle="50.0" potential="13.941103812989851" />
<me:PotentialPoint angle="55.0" potential="16.0517088355657" />
<me:PotentialPoint angle="60.0" potential="18.25816369171549" />
<me:PotentialPoint angle="65.0" potential="20.494101562261353" />
<me:PotentialPoint angle="70.0" potential="22.704795309312274" />
<me:PotentialPoint angle="75.0" potential="24.84935166228289" />
<me:PotentialPoint angle="80.0" potential="26.826157368470714" />
<me:PotentialPoint angle="85.0" potential="28.650048294921156" />
<me:PotentialPoint angle="90.0" potential="30.206411456974582" />
<me:PotentialPoint angle="95.0" potential="31.58202026258916" />
<me:PotentialPoint angle="100.0" potential="32.75804786234216" />
<me:PotentialPoint angle="105.0" potential="33.65696701551386" />
<me:PotentialPoint angle="110.0" potential="34.409673380735185" />
<me:PotentialPoint angle="115.0" potential="34.99141579889494" />
<me:PotentialPoint angle="120.0" potential="35.39765497084052" />
<me:PotentialPoint angle="125.0" potential="35.62324323495585" />
<me:PotentialPoint angle="130.0" potential="35.72113052906934" />
<me:PotentialPoint angle="135.0" potential="35.70119080352609" />
<me:PotentialPoint angle="140.0" potential="35.48081952400013" />
<me:PotentialPoint angle="145.0" potential="35.19984243261252" />
<me:PotentialPoint angle="150.0" potential="34.84120305338746" />
<me:PotentialPoint angle="155.0" potential="34.320905802436215" />

```
<me:PotentialPoint angle="160.0" potential="33.77676544736264" />
<me:PotentialPoint angle="165.0" potential="33.189713995367654" />
<me:PotentialPoint angle="170.0" potential="32.48424773665101" />
<me:PotentialPoint angle="175.0" potential="31.787982144736386" />
<me:PotentialPoint angle="180.0" potential="31.055157671892" />
<me:PotentialPoint angle="185.0" potential="30.311073439901246" />
<me:PotentialPoint angle="190.0" potential="29.52865494804535" />
<me:PotentialPoint angle="195.0" potential="28.75321043199359" />
<me:PotentialPoint angle="200.0" potential="27.956753482918405" />
<me:PotentialPoint angle="205.0" potential="27.160624788117246" />
<me:PotentialPoint angle="210.0" potential="26.333055104474056" />
<me:PotentialPoint angle="215.0" potential="25.51277132970083" />
<me:PotentialPoint angle="220.0" potential="24.635669270343108" />
<me:PotentialPoint angle="225.0" potential="23.768325931357968" />
<me:PotentialPoint angle="230.0" potential="22.823935139357424" />
<me:PotentialPoint angle="235.0" potential="21.89522713572852" />
<me:PotentialPoint angle="240.0" potential="20.887737171118008" />
<me:PotentialPoint angle="245.0" potential="19.898694545074928" />
<me:PotentialPoint angle="250.0" potential="18.918143195197658" />
<me:PotentialPoint angle="255.0" potential="17.782772413358117" />
<me:PotentialPoint angle="260.0" potential="16.734224222513333" />
<me:PotentialPoint angle="265.0" potential="15.609784494839156" />
<me:PotentialPoint angle="270.0" potential="14.525694793141732" />
<me:PotentialPoint angle="275.0" potential="13.382699149635702" />
<me:PotentialPoint angle="280.0" potential="12.26078877156331" />
<me:PotentialPoint angle="285.0" potential="11.051215567602126" />
<me:PotentialPoint angle="290.0" potential="9.860418758340959" />
<me:PotentialPoint angle="295.0" potential="8.580608086480648" />
<me:PotentialPoint angle="300.0" potential="7.31201513482457" />
<me:PotentialPoint angle="305.0" potential="5.977984642989554" />
<me:PotentialPoint angle="310.0" potential="4.687025966396566" />
<me:PotentialPoint angle="315.0" potential="3.458265493053738" />
<me:PotentialPoint angle="320.0" potential="2.2840281500321735" />
<me:PotentialPoint angle="325.0" potential="1.272547651672434" />
<me:PotentialPoint angle="330.0" potential="0.4370865018276809" />
<me:PotentialPoint angle="335.0" potential="-0.19750485622136607" />
<me:PotentialPoint angle="340.0" potential="-0.6101018919705556" />
<me:PotentialPoint angle="345.0" potential="-0.7920627908131447" />
<me:PotentialPoint angle="350.0" potential="-0.7464162029648939" />
<me:PotentialPoint angle="355.0" potential="-0.4794143723686256" />
<me:PotentialPoint angle="360.0" potential="-0.007382153122163883" />
</me:HinderedRotorPotential>
</me:ExtraDOSCMethod>
<me:ExtraDOSCMethod xsi:type="me:HinderedRotorQM1D">
<bondRef>bond34</bondRef>
<me:HinderedRotorPotential format="numerical" units="kJ/mol" expansionSize="10"
UseSineTerms="yes">
<me:PotentialPoint angle="0" potential="0.0" />
<me:PotentialPoint angle="5.0" potential="0.2146279307339461" />
<me:PotentialPoint angle="10.0" potential="1.0780174932777677" />
```

<me:PotentialPoint angle="15.0" potential="2.5765560920032384" />
<me:PotentialPoint angle="20.0" potential="4.660328968445228" />
<me:PotentialPoint angle="25.0" potential="7.178740293093182" />
<me:PotentialPoint angle="30.0" potential="10.055994998934061" />
<me:PotentialPoint angle="35.0" potential="13.213097112322036" />
<me:PotentialPoint angle="40.0" potential="16.406821402277497" />
<me:PotentialPoint angle="45.0" potential="19.343328009253153" />
<me:PotentialPoint angle="50.0" potential="22.20854457541851" />
<me:PotentialPoint angle="55.0" potential="24.4220232796992" />
<me:PotentialPoint angle="60.0" potential="25.707727491510653" />
<me:PotentialPoint angle="65.0" potential="25.32043899940468" />
<me:PotentialPoint angle="70.0" potential="24.948666616795766" />
<me:PotentialPoint angle="75.0" potential="24.582923745448664" />
<me:PotentialPoint angle="80.0" potential="24.33955857194614" />
<me:PotentialPoint angle="85.0" potential="24.22366602707283" />
<me:PotentialPoint angle="90.0" potential="24.187102018461367" />
<me:PotentialPoint angle="95.0" potential="24.11141765446661" />
<me:PotentialPoint angle="100.0" potential="24.050951250766765" />
<me:PotentialPoint angle="105.0" potential="23.573550969423906" />
<me:PotentialPoint angle="110.0" potential="22.20058933147729" />
<me:PotentialPoint angle="115.0" potential="19.641996130050085" />
<me:PotentialPoint angle="120.0" potential="16.89276978990041" />
<me:PotentialPoint angle="125.0" potential="14.425271008054013" />
<me:PotentialPoint angle="130.0" potential="11.601172032732077" />
<me:PotentialPoint angle="135.0" potential="8.792933784856807" />
<me:PotentialPoint angle="140.0" potential="6.3471879236797015" />
<me:PotentialPoint angle="145.0" potential="3.7242954791091463" />
<me:PotentialPoint angle="150.0" potential="1.3646481197523754" />
<me:PotentialPoint angle="155.0" potential="0.09778649661856577" />
<me:PotentialPoint angle="160.0" potential="-0.5131506098552393" />
<me:PotentialPoint angle="165.0" potential="-0.49523745957484616" />
<me:PotentialPoint angle="170.0" potential="0.15908474084551505" />
<me:PotentialPoint angle="175.0" potential="1.396772797842435" />
<me:PotentialPoint angle="180.0" potential="3.36378381466941" />
<me:PotentialPoint angle="185.0" potential="5.946780424728397" />
<me:PotentialPoint angle="190.0" potential="9.080538100691546" />
<me:PotentialPoint angle="195.0" potential="12.6736931283538" />
<me:PotentialPoint angle="200.0" potential="16.647737641759814" />
<me:PotentialPoint angle="205.0" potential="20.868138513308793" />
<me:PotentialPoint angle="210.0" potential="25.227809605491466" />
<me:PotentialPoint angle="215.0" potential="29.54978700775397" />
<me:PotentialPoint angle="220.0" potential="33.69359292510492" />
<me:PotentialPoint angle="225.0" potential="37.427891628179935" />
<me:PotentialPoint angle="230.0" potential="40.33777118334896" />
<me:PotentialPoint angle="235.0" potential="41.93420874630327" />
<me:PotentialPoint angle="240.0" potential="42.379050146904774" />
<me:PotentialPoint angle="245.0" potential="41.55497477314243" />
<me:PotentialPoint angle="250.0" potential="38.604511173340654" />
<me:PotentialPoint angle="255.0" potential="27.350243846210244" />
<me:PotentialPoint angle="260.0" potential="26.16417954825491" />

```
        <me:PotentialPoint angle="265.0" potential="24.438438551911702" />
        <me:PotentialPoint angle="270.0" potential="24.34654032303179" />
        <me:PotentialPoint angle="275.0" potential="24.51574012637032" />
        <me:PotentialPoint angle="280.0" potential="24.630131154076626" />
        <me:PotentialPoint angle="285.0" potential="24.741969309303332" />
        <me:PotentialPoint angle="290.0" potential="24.700934347916412" />
        <me:PotentialPoint angle="295.0" potential="24.084869452720906" />
        <me:PotentialPoint angle="300.0" potential="20.537304053533287" />
        <me:PotentialPoint angle="305.0" potential="17.161224894017458" />
        <me:PotentialPoint angle="310.0" potential="14.699451963257195" />
        <me:PotentialPoint angle="315.0" potential="12.132820939559258" />
        <me:PotentialPoint angle="320.0" potential="9.368514892525036" />
        <me:PotentialPoint angle="325.0" potential="6.740684868748846" />
        <me:PotentialPoint angle="330.0" potential="4.5753371519769805" />
        <me:PotentialPoint angle="335.0" potential="2.4533417883696553" />
        <me:PotentialPoint angle="340.0" potential="0.9920260481366876" />
        <me:PotentialPoint angle="345.0" potential="0.6128418912774743" />
        <me:PotentialPoint angle="350.0" potential="0.06285110423903853" />
        <me:PotentialPoint angle="355.0" potential="1.439731558165943" />
        <me:PotentialPoint angle="360.0" potential="3.469801014683794" />
      </me:HinderedRotorPotential>
    </me:ExtraDOSCMethod>
  </molecule>
  <molecule id="O=C=O">
    <atomArray>
      <atom id="a1" elementType="C" x3="0.948086" y3="0.026156" z3="-0.081368" />
      <atom id="a2" elementType="O" x3="2.145086" y3="0.026156" z3="-0.081368" />
      <atom id="a3" elementType="O" x3="-0.248914" y3="0.026156" z3="-0.081368" />
    </atomArray>
    <bondArray>
      <bond atomRefs2="a3 a1" order="2" />
      <bond atomRefs2="a1 a2" order="2" />
    </bondArray>
    <propertyList>
      <property dictRef="me:ZPE">
        <scalar units="kJ/mol">0</scalar>
      </property>
      <property dictRef="me:vibFreqs">
        <array units="cm-1">669.8606 669.8606 1411.2872 2460.5554</array>
      </property>
      <property dictRef="me:spinMultiplicity">
        <scalar units="cm-1">1</scalar>
      </property>
      <property dictRef="me:hessian">
        <matrix matrixType="squareSymmetricLT" rows="18"
units="Hartree/Bohr2">1.20396732 -0.60297369 0.49255125 0.69008851 -0. 39411988
0.59924443 -0.60198491 0.30148770 -0.34504524 0.64476706 0.30 148770 -0.24627587
0.19706050 -0.34709981 0.23524275 -0.34504524 0.197 06050 -0.29962261 0.39724717 -
0.22687381 0.29666033 -0.60198241 0.3014 8599 -0.34504328 -0.04278215 0.04561211 -
0.05220194 0.64476456 0.30148 599 -0.24627538 0.19705938 0.04561211 0.01103311
```

0.02981331 -0.3470981 0 0.23524227 -0.34504328 0.19705938 -0.29962182 -0.05220194 0.02981331 0.00296228 0.39724521 -0.22687269 0.29665954</matrix>
</property>
</propertyList>
</molecule>
<molecule spinMultiplicity="2" id="C[O]">
<atomArray>
<atom id="a1" elementType="O" spinMultiplicity="2" x3="0.933229" y3="-0.053412" z3="0.065184" />
<atom id="a2" elementType="C" x3="2.257786" y3="-0.053412" z3="0.065186" />
<atom id="a3" elementType="H" x3="2.763272" y3="0.050345" z3="1.040333" />
<atom id="a4" elementType="H" x3="2.763275" y3="-0.949791" z3="-0.332531" />
<atom id="a5" elementType="H" x3="2.763274" y3="0.739212" z3="-0.512241" />
</atomArray>
<bondArray>
<bond atomRefs2="a1 a2" order="1" />
<bond atomRefs2="a4 a2" order="1" />
<bond atomRefs2="a5 a2" order="1" />
<bond atomRefs2="a2 a3" order="1" />
</bondArray>
<propertyList>
<property dictRef="me:ZPE">
<scalar units="kJ/mol">0</scalar>
</property>
<property dictRef="me:vibFreqs">
<array units="cm-1">699.0453 975.4912 1145.1094 1394.2496 1395.2175 1523.6191 2974.6913 3053.5053 3095.113</array>
</property>
<property dictRef="me:spinMultiplicity">
<scalar units="cm-1">2</scalar>
</property>
<property dictRef="me:hessian">
<matrix matrixType="squareSymmetricLT" rows="30" units="Hartree/Bohr2">0.19553513 -0.117460 08 0.11536650 0.12507178 -0.08864868 0.12415264 -0.15570200 0.08672470 -0.10265411 0.50252918 0.06528965 -0.08015286 0.04931789 0.02720698 0 .43585746 -0.10259910 0.06545915 -0.09701143 0.03344988 0.02048908 0.4 8307803 -0.00540957 0.00037718 -0.02080253 -0.04242549 0.00475722 0.00 218889 0.04921867 0.00699845 -0.00257007 0.03029769 0.00321631 -0.0483 6732 -0.05479381 -0.01031279 0.05435054 -0.01562328 0.01037182 -0.0216 7400 0.03140673 -0.05069149 -0.29108998 -0.01884340 0.04470837 0.32534 514 -0.03001650 0.01010373 -0.00855620 -0.26340304 -0.04666446 0.09504 178 0.00266324 -0.00108620 0.00110316 0.29443781 0.03107557 -0.0026683 7 -0.00161890 -0.05113296 -0.04805553 0.01798167 0.00795092 0.00194889 -0.00341581 0.03934606 0.05405472 -0.01380417 -0.00246621 0.00302852 0.06584953 0.01839145 -0.07041926 0.03590896 0.00847497 -0.00799169 -0 .08944538 -0.02200317 0.08042560 -0.00440706 0.02025448 0.00694106 -0. 04099865 -0.05058939 -0.02808145 -0.00404686 0.00118423 0.00195679 -0. 00368151 -0.02723959 0.00149105 0.05313408 0.01409642 -0.02997520 0.01 065200 -0.06601503 -0.25928175 -0.04913610 -0.00277253 -0.00536203 -0. 00097289 -0.00169913 -0.00527972 -0.00239703 0.05639026 0.29989871 0.0 0695477 0.01528392 -0.00849573 -0.02805203 -0.03750693 -

0.02455737 0.0 0154808 -0.02868722 -0.00458948 0.00185664 0.00905620 -0.00504318 0.01 769255 0.04185403 0.04268575</matrix>
</property>
</propertyList>
</molecule>
<molecule spinMultiplicity="3" id="[C]=O">
<atomArray>
<atom id="a1" elementType="C" spinMultiplicity="3" x3="0.996032" y3="-0.076595" z3="-0.049588" />
<atom id="a2" elementType="O" x3="2.193032" y3="-0.076595" z3="-0.049588" />
</atomArray>
<bondArray>
<bond atomRefs2="a2 a1" order="2" />
</bondArray>
<propertyList>
<property dictRef="me:ZPE">
<scalar units="kJ/mol">2.26292883</scalar>
</property>
<property dictRef="me:vibFreqs">
<array units="cm-1">2277.8678</array>
</property>
<property dictRef="me:spinMultiplicity">
<scalar units="cm-1">1</scalar>
</property>
<property dictRef="me:hessian">
<matrix matrixType="squareSymmetricLT" rows="12" units="Hartree/Bohr2">0.55589341 -0.50570552 0.45991628 0.42858481 -0.389 83992 0.33031637 -0.55589341 0.50570552 -0.42858481 0.55589341 0.50570 552 -0.45991628 0.38983992 -0.50570552 0.45991628 -0.42858481 0.389839 92 -0.33031637 0.42858481 -0.38983992 0.33031637</matrix>
</property>
</propertyList>
</molecule>
<molecule spinMultiplicity="4" id="TS1">
<atomArray>
<atom id="a1" elementType="C" x3="0.224440" y3="1.104170" z3="0.217040" />
<atom id="a2" elementType="O" x3="-0.493530" y3="1.790890" z3="-0.335770" />
<atom id="a3" elementType="O" x3="-0.017080" y3="-0.787590" z3="0.058320" />
<atom id="a4" elementType="C" x3="0.935480" y3="-1.500340" z3="0.789280" />
<atom id="a5" elementType="H" x3="0.866390" y3="-1.308620" z3="1.865150" />
<atom id="a6" elementType="H" x3="1.954820" y3="-1.327020" z3="0.428800" />
<atom id="a7" elementType="H" x3="0.681830" y3="-2.554990" z3="0.610570" />
</atomArray>
<bondArray>
<bond atomRefs2="a2 a1" order="2" />
<bond atomRefs2="a3 a4" order="1" id="bond34" />
<bond atomRefs2="a6 a4" order="1" />
<bond atomRefs2="a7 a4" order="1" />
<bond atomRefs2="a4 a5" order="1" />
<bond atomRefs2="a3 a1" order="1" id="bond31" />

</bondArray>
<propertyList>
<property dictRef="me:ZPE">
<scalar units="kJ/mol">10.05705609</scalar>
</property>
<property dictRef="me:vibFreqs">
<array units="cm-1">98.1058 168.9676 214.6586 333.0632 1027.7224 1152.7269 1163.7027 1430.6388 1444.0681 1520.5029 2199.3797 3030.1505 3104.1379 3131.1415</array>
</property>
<property dictRef="me:spinMultiplicity">
<scalar units="cm-1">2</scalar>
</property>
<property dictRef="me:hessian">
<matrix matrixType="squareSymmetricLT" rows="21" units="Hartree/Bohr2">0.53532046 -0.46584194 0.35454895 0.40881640 -0.35746296 0.31799043 -0.51816910 0.45839599 -0.39692255 0.51123630 0.47338143 -0.45190287 0.36444526 -0.48048495 0.48487419 -0.39711483 0.35309096 -0.30748760 0.39226083 -0.37025592 0.30362381 -0.02272350 -0.01071160 -0.01532397 0.00776061 0.01013939 0.00581807 0.21075423 -0.01792701 0.10979053 -0.01502193 0.02195532 -0.03060689 0.01702689 -0.09406673 0.04001158 -0.01523166 -0.00955553 -0.01373608 0.00566651 0.00807446 0.00410003 0.12475765 -0.07118140 0.14315498 0.00305643 0.01561618 -0.00038357 0.00175542 -0.00442276 0.00179032 -0.16809316 0.06240199 -0.08007047 0.54851260 0.00790652 -0.01754788 0.00625790 0.00095053 -0.00141378 0.00073177 0.04984271 -0.08721076 0.03807418 0.04382429 0.49415239 -0.00028447 0.01209955 0.00292644 0.00172167 -0.00334027 0.00081514 -0.07991097 0.04759186 -0.12464601 -0.02441683 0.03430923 0.56105707 0.00054567 0.00249190 0.00251048 -0.00075073 0.00022604 -0.00136073 -0.00030387 -0.00278928 -0.03162874 -0.05762341 0.00513447 0.02777042 0.05732234 -0.00005085 0.00014563 0.00110243 -0.00021711 0.00012688 -0.00034637 0.00756340 -0.00049397 0.02765543 0.00141336 -0.05658385 -0.05846079 -0.00961743 0.05923155 0.00061181 -0.00097939 0.00117525 -0.00039416 0.00030397 -0.00056122 -0.01303193 0.00766530 -0.01984829 0.02896031 -0.05443942 -0.29867420 -0.01895177 0.05228429 0.32953798 0.00195188 -0.00028727 0.00021949 -0.00103241 0.00036281 -0.00032225 -0.02961100 0.00690650 -0.00478662 -0.26753597 -0.04698299 0.08857657 0.00010839 -0.00166771 0.00113579 0.30087624 0.00106008 0.00017312 -0.00035610 -0.00039870 0.00012778 -0.00011813 0.02864600 -0.00100615 0.00003382 -0.05187953 -0.05474084 0.01552199 0.00697122 0.00233927 -0.00373916 0.04332463 0.05737157 0.00213380 0.00263155 -0.00025892 -0.00127365 0.00014456 -0.00028018 -0.02379412 -0.00429487 0.00997081 0.08749655 0.01803099 -0.09060247 0.02839967 0.00720040 -0.00825638 -0.08808132 -0.02184534 0.08784225 0.00001816 0.00033674 0.00108373 -0.00080009 0.00079804 -0.00107142 0.00221668 0.02351922 0.00129333 -0.06007189 -0.06067553 -0.01345638 0.00070162 0.00257633 0.00166995 -0.00475714 -0.02772369 -0.00488093 0.06269266 0.00147177 0.00479252 0.00103541 -0.00020109 -0.00120532 -0.00012920 0.00858684 -0.03048434 0.00689905 -0.06695353 -0.27665529 -0.04772158 -0.00241691 -0.00476552 -0.00109558 -0.00165597 -0.00426475 -0.00186729 0.06116889 0.31258269 0.00106895 0.00017582 -0.00060952 -0.00105866 0.00062795 -0.00020998 0.00148528 0.01821415 0.00100455 -0.01337631 -0.04296463 -0.05087597 -0.00673933 -0.02943539 -0.00337315 0.00325834 0.01050291 0.00158488 0.01536172 0.04287919 0.05247919</matrix>
</property>

<property dictRef="me:imFreqs">
<scalar units="cm-1">489.8933</scalar>
</property>
<property dictRef="me:vibFreqsFromHessian">
<scalar calculated="20220120_074051" units="cm-1">98.1055 168.967 214.658 333.062 1027.72 1152.72 1163.7 1430.63 1444.06 1520.5 2199.37 3030.14 3104.13 3131.13 </scalar>
</property>
<property dictRef="me:frequenciesScaleFactor" default="true">
<scalar>0.9522</scalar>
</property>
<property dictRef="me:symmetryNumber" default="true">
<scalar>1</scalar>
</property>
</propertyList>
<me:ExtraDOSCMethod xsi:type="me:HinderedRotorQM1D">
<bondRef>bond31</bondRef>
<me:HinderedRotorPotential format="numerical" units="kJ/mol" expansionSize="10" UseSineTerms="yes">
<me:PotentialPoint angle="0" potential="0.0" />
<me:PotentialPoint angle="5.0" potential="0.016117150212008956" />
<me:PotentialPoint angle="10.0" potential="0.06948195103822233" />
<me:PotentialPoint angle="15.0" potential="0.1677170050808831" />
<me:PotentialPoint angle="20.0" potential="0.32169888846655753" />
<me:PotentialPoint angle="25.0" potential="0.5439898334989649" />
<me:PotentialPoint angle="30.0" potential="0.8014054540822224" />
<me:PotentialPoint angle="35.0" potential="1.1824768751282353" />
<me:PotentialPoint angle="40.0" potential="1.6939392021353739" />
<me:PotentialPoint angle="45.0" potential="2.2572782231472273" />
<me:PotentialPoint angle="50.0" potential="3.035348373510545" />
<me:PotentialPoint angle="55.0" potential="3.9213862494759617" />
<me:PotentialPoint angle="60.0" potential="5.051064871242922" />
<me:PotentialPoint angle="65.0" potential="6.416318079049647" />
<me:PotentialPoint angle="70.0" potential="7.861866372514429" />
<me:PotentialPoint angle="75.0" potential="9.60589573632138" />
<me:PotentialPoint angle="80.0" potential="11.576379125955711" />
<me:PotentialPoint angle="85.0" potential="12.89016751126117" />
<me:PotentialPoint angle="90.0" potential="15.017459491509175" />
<me:PotentialPoint angle="95.0" potential="17.20308440316359" />
<me:PotentialPoint angle="100.0" potential="19.501964971959552" />
<me:PotentialPoint angle="105.0" potential="21.565563753017557" />
<me:PotentialPoint angle="110.0" potential="23.403142763880133" />
<me:PotentialPoint angle="115.0" potential="24.965196133300996" />
<me:PotentialPoint angle="120.0" potential="26.309492212751117" />
<me:PotentialPoint angle="125.0" potential="27.490438986689565" />
<me:PotentialPoint angle="130.0" potential="28.583210553566456" />
<me:PotentialPoint angle="135.0" potential="29.633260235091697" />
<me:PotentialPoint angle="140.0" potential="30.723152982467" />
<me:PotentialPoint angle="145.0" potential="30.970602155300774" />
<me:PotentialPoint angle="150.0" potential="31.83174283726364" />

```
<me:PotentialPoint angle="155.0" potential="32.67846559316943" />
<me:PotentialPoint angle="160.0" potential="33.572550017550896" />
<me:PotentialPoint angle="165.0" potential="34.465166257875836" />
<me:PotentialPoint angle="170.0" potential="34.33746910049392" />
<me:PotentialPoint angle="175.0" potential="34.77154469764189" />
<me:PotentialPoint angle="180.0" potential="35.154903231926674" />
<me:PotentialPoint angle="185.0" potential="35.464592016784756" />
<me:PotentialPoint angle="190.0" potential="35.693661223049276" />
<me:PotentialPoint angle="195.0" potential="33.93288923957357" />
<me:PotentialPoint angle="200.0" potential="33.407979518900774" />
<me:PotentialPoint angle="205.0" potential="32.88714710256945" />
<me:PotentialPoint angle="210.0" potential="31.900954185858808" />
<me:PotentialPoint angle="215.0" potential="31.161209773158983" />
<me:PotentialPoint angle="220.0" potential="30.431260776458853" />
<me:PotentialPoint angle="225.0" potential="29.61382311428517" />
<me:PotentialPoint angle="230.0" potential="28.797863844630154" />
<me:PotentialPoint angle="235.0" potential="27.876602813071067" />
<me:PotentialPoint angle="240.0" potential="26.77089564543782" />
<me:PotentialPoint angle="245.0" potential="25.39674676505159" />
<me:PotentialPoint angle="250.0" potential="23.73412368639874" />
<me:PotentialPoint angle="255.0" potential="21.843946227059412" />
<me:PotentialPoint angle="260.0" potential="19.839489194539734" />
<me:PotentialPoint angle="265.0" potential="17.850858053461586" />
<me:PotentialPoint angle="270.0" potential="15.833024159539674" />
<me:PotentialPoint angle="275.0" potential="13.965568948686292" />
<me:PotentialPoint angle="280.0" potential="11.234657452096325" />
<me:PotentialPoint angle="285.0" potential="9.414969017432828" />
<me:PotentialPoint angle="290.0" potential="7.802181917681005" />
<me:PotentialPoint angle="295.0" potential="6.352473249652558" />
<me:PotentialPoint angle="300.0" potential="5.1375531811993636" />
<me:PotentialPoint angle="305.0" potential="4.0613960084335785" />
<me:PotentialPoint angle="310.0" potential="3.2089577999358547" />
<me:PotentialPoint angle="315.0" potential="2.548979628040774" />
<me:PotentialPoint angle="320.0" potential="1.6079969779616121" />
<me:PotentialPoint angle="325.0" potential="1.1455994191185712" />
<me:PotentialPoint angle="330.0" potential="0.7766757078733877" />
<me:PotentialPoint angle="335.0" potential="0.5181136894173684" />
<me:PotentialPoint angle="340.0" potential="0.34565100214440514" />
<me:PotentialPoint angle="345.0" potential="0.18884991154968728" />
<me:PotentialPoint angle="350.0" potential="0.11211774097072387" />
<me:PotentialPoint angle="355.0" potential="0.08387083606324944" />
<me:PotentialPoint angle="360.0" potential="0.08323519496560605" />
</me:HinderedRotorPotential>
</me:ExtraDOSCMethod>
<me:ExtraDOSCMethod xsi:type="me:HinderedRotorQM1D">
<bondRef>bond34</bondRef>
<me:periodicity>3</me:periodicity>
<me:HinderedRotorPotential format="numerical" units="kJ/mol" expansionSize="10"
UseSineTerms="yes">
<me:PotentialPoint angle="0" potential="0.0" />
```

<me:PotentialPoint angle="5.0" potential="0.15357551429212069" />
<me:PotentialPoint angle="10.0" potential="0.5212677555020743" />
<me:PotentialPoint angle="15.0" potential="1.0459314323355746" />
<me:PotentialPoint angle="20.0" potential="1.6861522596460223" />
<me:PotentialPoint angle="25.0" potential="2.3564609275889143" />
<me:PotentialPoint angle="30.0" potential="2.991337704540303" />
<me:PotentialPoint angle="35.0" potential="3.5255425002586795" />
<me:PotentialPoint angle="40.0" potential="3.921428679659782" />
<me:PotentialPoint angle="45.0" potential="4.162378279290841" />
<me:PotentialPoint angle="50.0" potential="4.272729520544559" />
<me:PotentialPoint angle="55.0" potential="4.346121717864237" />
<me:PotentialPoint angle="60.0" potential="4.371858395781603" />
<me:PotentialPoint angle="65.0" potential="4.344750245470085" />
<me:PotentialPoint angle="70.0" potential="4.267869154183783" />
<me:PotentialPoint angle="75.0" potential="4.131197787291358" />
<me:PotentialPoint angle="80.0" potential="3.9145585546757906" />
<me:PotentialPoint angle="85.0" potential="3.596644720917575" />
<me:PotentialPoint angle="90.0" potential="3.163728593739747" />
<me:PotentialPoint angle="95.0" potential="2.6207545511903616" />
<me:PotentialPoint angle="100.0" potential="2.0030970222550093" />
<me:PotentialPoint angle="105.0" potential="1.3686137084199683" />
<me:PotentialPoint angle="110.0" potential="0.7952423433141325" />
<me:PotentialPoint angle="115.0" potential="0.3470224594218352" />
<me:PotentialPoint angle="120.0" potential="0.08856504258429257" />
<me:PotentialPoint angle="125.0" potential="0.04486002182636086" />
<me:PotentialPoint angle="130.0" potential="0.24757669977016097" />
<me:PotentialPoint angle="135.0" potential="0.6703371739160244" />
<me:PotentialPoint angle="140.0" potential="1.2855395667989669" />
<me:PotentialPoint angle="145.0" potential="1.9961423129738558" />
<me:PotentialPoint angle="150.0" potential="2.7980079850123034" />
<me:PotentialPoint angle="155.0" potential="3.486522928141583" />
<me:PotentialPoint angle="160.0" potential="4.0216723294597605" />
<me:PotentialPoint angle="165.0" potential="4.320487039135592" />
<me:PotentialPoint angle="170.0" potential="4.530129774965973" />
<me:PotentialPoint angle="175.0" potential="4.525639944195728" />
<me:PotentialPoint angle="180.0" potential="4.528831147873699" />
<me:PotentialPoint angle="185.0" potential="4.386007406641231" />
<me:PotentialPoint angle="190.0" potential="4.275139601103938" />
<me:PotentialPoint angle="195.0" potential="4.016129683772819" />
<me:PotentialPoint angle="200.0" potential="3.7418355161670145" />
<me:PotentialPoint angle="205.0" potential="3.3336121738337203" />
<me:PotentialPoint angle="210.0" potential="2.8492541291315248" />
<me:PotentialPoint angle="215.0" potential="2.2750819063776233" />
<me:PotentialPoint angle="220.0" potential="1.6640423369830044" />
<me:PotentialPoint angle="225.0" potential="1.0726230745890433" />
<me:PotentialPoint angle="230.0" potential="0.5619845238628782" />
<me:PotentialPoint angle="235.0" potential="0.1855959868188346" />
<me:PotentialPoint angle="240.0" potential="0.015268383312405799" />
<me:PotentialPoint angle="245.0" potential="0.05066214925230796" />
<me:PotentialPoint angle="250.0" potential="0.2813543090381429" />

<me:PotentialPoint angle="255.0" potential="0.7058432734057436" />
<me:PotentialPoint angle="260.0" potential="1.2799740467449006" />
<me:PotentialPoint angle="265.0" potential="1.9552583513957602" />
<me:PotentialPoint angle="270.0" potential="2.654849390845307" />
<me:PotentialPoint angle="275.0" potential="3.2970885124573432" />
<me:PotentialPoint angle="280.0" potential="3.8671027798145112" />
<me:PotentialPoint angle="285.0" potential="4.230538452673686" />
<me:PotentialPoint angle="290.0" potential="4.48469869855072" />
<me:PotentialPoint angle="295.0" potential="4.567107918636899" />
<me:PotentialPoint angle="300.0" potential="4.573368989746086" />
<me:PotentialPoint angle="305.0" potential="4.473681060559928" />
<me:PotentialPoint angle="310.0" potential="4.346026621007667" />
<me:PotentialPoint angle="315.0" potential="4.149954714967705" />
<me:PotentialPoint angle="320.0" potential="3.9022756324364387" />
<me:PotentialPoint angle="325.0" potential="3.5263012679945773" />
<me:PotentialPoint angle="330.0" potential="3.082694992477639" />
<me:PotentialPoint angle="335.0" potential="2.4308577160741525" />
<me:PotentialPoint angle="340.0" potential="1.7122733693548955" />
<me:PotentialPoint angle="345.0" potential="1.086358714012821" />
<me:PotentialPoint angle="350.0" potential="0.5163421254100121" />
<me:PotentialPoint angle="355.0" potential="0.1953114028412892" />
<me:PotentialPoint angle="360.0" potential="0.05058753705919749" />
</me:HinderedRotorPotential>
</me:ExtraDOSCMethod>
<me:DOSCMethod default="true" name="ClassicalRotors" />
</molecule>
<molecule id="[CH3]">
<atomArray>
<atom id="a1" elementType="C" x3="0.948086" y3="0.026156" z3="-0.081368" />
<atom id="a2" elementType="O" x3="2.145086" y3="0.026156" z3="-0.081368" />
<atom id="a3" elementType="O" x3="-0.248914" y3="0.026156" z3="-0.081368" />
</atomArray>
<bondArray>
<bond atomRefs2="a3 a1" order="2" />
<bond atomRefs2="a1 a2" order="2" />
</bondArray>
<propertyList>
<property dictRef="me:ZPE">
<scalar units="kJ/mol">-160</scalar>
</property>
<property dictRef="me:vibFreqs">
<array units="cm-1">669.8606 669.8606 1411.2872 2460.5554</array>
</property>
<property dictRef="me:spinMultiplicity">
<scalar units="cm-1">1</scalar>
</property>
<property dictRef="me:hessian">
<matrix matrixType="squareSymmetricLT" rows="18"
units="Hartree/Bohr2">1.20396732 -0.60297369 0.49255125 0.69008851 -0. 39411988
0.59924443 -0.60198491 0.30148770 -0.34504524 0.64476706 0.30 148770 -0.24627587

0.19706050 -0.34709981 0.23524275 -0.34504524 0.197 06050 -0.29962261 0.39724717 -0.22687381 0.29666033 -0.60198241 0.3014 8599 -0.34504328 -0.04278215 0.04561211 -0.05220194 0.64476456 0.30148 599 -0.24627538 0.19705938 0.04561211 0.01103311 0.02981331 -0.3470981 0 0.23524227 -0.34504328 0.19705938 -0.29962182 -0.05220194 0.02981331 0.00296228 0.39724521 -0.22687269 0.29665954</matrix>
</property>
</propertyList>
</molecule>
</moleculeList>
<reactionList>
<reaction active="true" id="R1">
<reactant>
<molecule role="modelled" ref="COC=O"/>
</reactant>
<reactant>
<molecule role="excessReactant" ref="[OH]"/>
</reactant>
<product>
<molecule role="modelled" ref="CH_pre_comp"/>
</product>
<me:MCRCMethod xsi:type="me:MesmerILT">
<me:preExponential units="cm3molecule-1s-1">3.e-10</me:preExponential>
<me:activationEnergy units="cm-1">0.0</me:activationEnergy>
<me:nInfinity>0</me:nInfinity>
</me:MCRCMethod>
<me:excessReactantConc>1E16</me:excessReactantConc>
</reaction>
<reaction active="true" id="R2">
<reactant>
<molecule role="modelled" ref="CH_pre_comp"/>
</reactant>
<product>
<molecule role="sink" ref="CH_post_comp"/>
</product>
<me:MCRCMethod name="RRKM"/>
<me:transitionState>
<molecule role="transitionState" ref="TS_COC=O_CO[C]=O"/>
</me:transitionState>
<me:tunneling xsi:type="me:WKB">
<me:IRCPotential units="kJ/mol" ReducedMass="1">
<me:PotentialPoint ReacCoord= " -3.87079E-10 " potential= " 0 " />
<me:PotentialPoint ReacCoord= " -3.80837E-10 " potential= " 0.8060285 " />
<me:PotentialPoint ReacCoord= " -3.74595E-10 " potential= " 1.3783875 " />
<me:PotentialPoint ReacCoord= " -3.68353E-10 " potential= " 1.428272 " />
<me:PotentialPoint ReacCoord= " -3.62111E-10 " potential= " 3.208361 " />
<me:PotentialPoint ReacCoord= " -3.5587E-10 " potential= " 4.5342385 " />
<me:PotentialPoint ReacCoord= " -3.49629E-10 " potential= " 5.749845 " />
<me:PotentialPoint ReacCoord= " -3.43387E-10 " potential= " 6.6661445 " />
<me:PotentialPoint ReacCoord= " -3.37144E-10 " potential= " 7.4012845 " />
<me:PotentialPoint ReacCoord= " -3.30901E-10 " potential= " 7.9316355 " />

<me:PotentialPoint ReacCoord= " -3.24657E-10 " potential= " 8.328086 " />
<me:PotentialPoint ReacCoord= " -3.18414E-10 " potential= " 8.6195165 " />
<me:PotentialPoint ReacCoord= " -3.1217E-10 " potential= " 8.863688 " />
<me:PotentialPoint ReacCoord= " -3.05927E-10 " potential= " 9.0763535 " />
<me:PotentialPoint ReacCoord= " -2.99683E-10 " potential= " 9.268015 " />
<me:PotentialPoint ReacCoord= " -2.93439E-10 " potential= " 9.4518 " />
<me:PotentialPoint ReacCoord= " -2.87196E-10 " potential= " 9.635585 " />
<me:PotentialPoint ReacCoord= " -2.80951E-10 " potential= " 9.81937 " />
<me:PotentialPoint ReacCoord= " -2.74708E-10 " potential= " 9.9795255 " />
<me:PotentialPoint ReacCoord= " -2.68464E-10 " potential= " 10.2525775 " />
<me:PotentialPoint ReacCoord= " -2.62221E-10 " potential= " 10.202693 " />
<me:PotentialPoint ReacCoord= " -2.55977E-10 " potential= " 10.2315735 " />
<me:PotentialPoint ReacCoord= " -2.49733E-10 " potential= " 10.407482 " />
<me:PotentialPoint ReacCoord= " -2.43489E-10 " potential= " 10.60702 " />
<me:PotentialPoint ReacCoord= " -2.37246E-10 " potential= " 10.806558 " />
<me:PotentialPoint ReacCoord= " -2.31002E-10 " potential= " 11.0192235 " />
<me:PotentialPoint ReacCoord= " -2.24759E-10 " potential= " 11.258144 " />
<me:PotentialPoint ReacCoord= " -2.18515E-10 " potential= " 11.541698 " />
<me:PotentialPoint ReacCoord= " -2.12272E-10 " potential= " 11.8593835 " />
<me:PotentialPoint ReacCoord= " -2.06029E-10 " potential= " 12.219077 " />
<me:PotentialPoint ReacCoord= " -1.99785E-10 " potential= " 12.6102765 " />
<me:PotentialPoint ReacCoord= " -1.93542E-10 " potential= " 13.006727 " />
<me:PotentialPoint ReacCoord= " -1.873E-10 " potential= " 13.384799 " />
<me:PotentialPoint ReacCoord= " -1.81057E-10 " potential= " 13.741867 " />
<me:PotentialPoint ReacCoord= " -1.74814E-10 " potential= " 14.0753055 " />
<me:PotentialPoint ReacCoord= " -1.68571E-10 " potential= " 14.403493 " />
<me:PotentialPoint ReacCoord= " -1.62328E-10 " potential= " 14.75531 " />
<me:PotentialPoint ReacCoord= " -1.56085E-10 " potential= " 15.149135 " />
<me:PotentialPoint ReacCoord= " -1.49843E-10 " potential= " 15.579717 " />
<me:PotentialPoint ReacCoord= " -1.436E-10 " potential= " 16.036554 " />
<me:PotentialPoint ReacCoord= " -1.37357E-10 " potential= " 16.498642 " />
<me:PotentialPoint ReacCoord= " -1.31113E-10 " potential= " 16.9528535 " />
<me:PotentialPoint ReacCoord= " -1.2487E-10 " potential= " 17.3939375 " />
<me:PotentialPoint ReacCoord= " -1.18627E-10 " potential= " 17.8350215 " />
<me:PotentialPoint ReacCoord= " -1.12384E-10 " potential= " 18.2813565 " />
<me:PotentialPoint ReacCoord= " -1.06141E-10 " potential= " 18.7486955 " />
<me:PotentialPoint ReacCoord= " -9.98972E-11 " potential= " 19.2422895 " />
<me:PotentialPoint ReacCoord= " -9.36541E-11 " potential= " 19.764764 " />
<me:PotentialPoint ReacCoord= " -8.74106E-11 " potential= " 20.3239955 " />
<me:PotentialPoint ReacCoord= " -8.1167E-11 " potential= " 20.914733 " />
<me:PotentialPoint ReacCoord= " -7.49234E-11 " potential= " 21.5291 " />
<me:PotentialPoint ReacCoord= " -6.86798E-11 " potential= " 22.1618455 " />
<me:PotentialPoint ReacCoord= " -6.24363E-11 " potential= " 22.799842 " />
<me:PotentialPoint ReacCoord= " -5.61927E-11 " potential= " 23.440464 " />
<me:PotentialPoint ReacCoord= " -4.99486E-11 " potential= " 24.086337 " />
<me:PotentialPoint ReacCoord= " -4.3705E-11 " potential= " 24.737461 " />
<me:PotentialPoint ReacCoord= " -3.74614E-11 " potential= " 25.399087 " />
<me:PotentialPoint ReacCoord= " -3.12179E-11 " potential= " 26.0633385 " />
<me:PotentialPoint ReacCoord= " -2.49743E-11 " potential= " 26.7197135 " />
<me:PotentialPoint ReacCoord= " -1.87307E-11 " potential= " 27.341957 " />

<me:PotentialPoint ReacCoord= " -1.24871E-11 " potential= " 27.898563 " />
<me:PotentialPoint ReacCoord= " -6.24357E-12 " potential= " 28.3554 " />
<me:PotentialPoint ReacCoord= " 0 " potential= " 28.680962 " />
<me:PotentialPoint ReacCoord= " 6.24253E-12 " potential= " 28.675711 " />
<me:PotentialPoint ReacCoord= " 1.24803E-11 " potential= " 27.693774 " />
<me:PotentialPoint ReacCoord= " 1.87161E-11 " potential= " 27.546746 " />
<me:PotentialPoint ReacCoord= " 2.4695E-11 " potential= " 23.482472 " />
<me:PotentialPoint ReacCoord= " 3.08805E-11 " potential= " 23.3958305 " />
<me:PotentialPoint ReacCoord= " 3.71184E-11 " potential= " 18.3443685 " />
<me:PotentialPoint ReacCoord= " 4.33604E-11 " potential= " 7.8948785 " />
<me:PotentialPoint ReacCoord= " 4.96034E-11 " potential= " -6.973328 " />
<me:PotentialPoint ReacCoord= " 5.58465E-11 " potential= " -23.9104285 " />
<me:PotentialPoint ReacCoord= " 6.20838E-11 " potential= " -38.253535 " />
<me:PotentialPoint ReacCoord= " 6.80674E-11 " potential= " -38.5029575 " />
<me:PotentialPoint ReacCoord= " 7.42064E-11 " potential= " -39.8997235 " />
<me:PotentialPoint ReacCoord= " 8.04201E-11 " potential= " -41.288613 " />
<me:PotentialPoint ReacCoord= " 8.66533E-11 " potential= " -42.79565 " />
<me:PotentialPoint ReacCoord= " 9.28921E-11 " potential= " -44.2790575 " />
<me:PotentialPoint ReacCoord= " 9.91331E-11 " potential= " -45.715206 " />
<me:PotentialPoint ReacCoord= " 1.05376E-10 " potential= " -47.096219 " />
<me:PotentialPoint ReacCoord= " 1.11619E-10 " potential= " -48.3092 " />
<me:PotentialPoint ReacCoord= " 1.17863E-10 " potential= " -49.642954 " />
<me:PotentialPoint ReacCoord= " 1.24106E-10 " potential= " -51.102732 " />
<me:PotentialPoint ReacCoord= " 1.3035E-10 " potential= " -52.7069125 " />
<me:PotentialPoint ReacCoord= " 1.36594E-10 " potential= " -54.342599 " />
<me:PotentialPoint ReacCoord= " 1.42838E-10 " potential= " -55.886393 " />
<me:PotentialPoint ReacCoord= " 1.49082E-10 " potential= " -57.2280235 " />
<me:PotentialPoint ReacCoord= " 1.55326E-10 " potential= " -58.270347 " />
<me:PotentialPoint ReacCoord= " 1.61569E-10 " potential= " -59.252284 " />
<me:PotentialPoint ReacCoord= " 1.67813E-10 " potential= " -60.160707 " />
<me:PotentialPoint ReacCoord= " 1.74057E-10 " potential= " -60.974612 " />
<me:PotentialPoint ReacCoord= " 1.80301E-10 " potential= " -61.688748 " />
<me:PotentialPoint ReacCoord= " 1.86545E-10 " potential= " -62.334621 " />
<me:PotentialPoint ReacCoord= " 1.92788E-10 " potential= " -62.6680595 " />
<me:PotentialPoint ReacCoord= " 1.99032E-10 " potential= " -62.9043545 " />
<me:PotentialPoint ReacCoord= " 2.05276E-10 " potential= " -63.180032 " />
<me:PotentialPoint ReacCoord= " 2.1152E-10 " potential= " -63.453084 " />
<me:PotentialPoint ReacCoord= " 2.17763E-10 " potential= " -63.7077575 " />
<me:PotentialPoint ReacCoord= " 2.24007E-10 " potential= " -63.9230485 " />
<me:PotentialPoint ReacCoord= " 2.3025E-10 " potential= " -64.051698 " />
<me:PotentialPoint ReacCoord= " 2.36493E-10 " potential= " -64.088455 " />
<me:PotentialPoint ReacCoord= " 2.42736E-10 " potential= " -64.1120845 " />
<me:PotentialPoint ReacCoord= " 2.48979E-10 " potential= " -64.1750965 " />
<me:PotentialPoint ReacCoord= " 2.55222E-10 " potential= " -64.298495 " />
<me:PotentialPoint ReacCoord= " 2.61465E-10 " potential= " -64.487531 " />
<me:PotentialPoint ReacCoord= " 2.67709E-10 " potential= " -64.7317025 " />
<me:PotentialPoint ReacCoord= " 2.73952E-10 " potential= " -65.0100055 " />
<me:PotentialPoint ReacCoord= " 2.80194E-10 " potential= " -65.222671 " />
<me:PotentialPoint ReacCoord= " 2.86437E-10 " potential= " -65.5088505 " />
<me:PotentialPoint ReacCoord= " 2.9268E-10 " potential= " -65.8029065 " />

```
<me:PotentialPoint ReacCoord= "  2.98922E-10  "  potential= "  -66.1022135  "  />
<me:PotentialPoint ReacCoord= "  3.05165E-10  "  potential= "  -66.4067715  "  />
<me:PotentialPoint ReacCoord= "  3.11408E-10  "  potential= "  -66.729708  "  />
<me:PotentialPoint ReacCoord= "  3.1765E-10  "  potential= "  -67.0368915  "  />
<me:PotentialPoint ReacCoord= "  3.23893E-10  "  potential= "  -67.3782065  "  />
<me:PotentialPoint ReacCoord= "  3.30136E-10  "  potential= "  -67.716896  "  />
<me:PotentialPoint ReacCoord= "  3.36378E-10  "  potential= "  -68.068713  "  />
<me:PotentialPoint ReacCoord= "  3.42621E-10  "  potential= "  -68.457287  "  />
<me:PotentialPoint ReacCoord= "  3.48865E-10  "  potential= "  -68.851112  "  />
<me:PotentialPoint ReacCoord= "  3.55108E-10  "  potential= "  -69.2475625  "  />
<me:PotentialPoint ReacCoord= "  3.61351E-10  "  potential= "  -69.5626225  "  />
<me:PotentialPoint ReacCoord= "  3.67594E-10  "  potential= "  -69.817296  "  />
<me:PotentialPoint ReacCoord= "  3.73838E-10  "  potential= "  -69.8146705  "  />
<me:PotentialPoint ReacCoord= "  3.80081E-10  "  potential= "  -69.7254035  "  />
<me:PotentialPoint ReacCoord= "  3.86325E-10  "  potential= "  -69.5626225  "  />
<me:PotentialPoint ReacCoord= "  3.92569E-10  "  potential= "  -69.4628535  "  />
<me:PotentialPoint ReacCoord= "  3.98813E-10  "  potential= "  -69.4313475  "  />
<me:PotentialPoint ReacCoord= "  4.05056E-10  "  potential= "  -69.4628535  "  />
<me:PotentialPoint ReacCoord= "  4.113E-10  "  potential= "  -69.559997  "  />
<me:PotentialPoint ReacCoord= "  4.17544E-10  "  potential= "  -69.670268  "  />
<me:PotentialPoint ReacCoord= "  4.23788E-10  "  potential= "  -69.817296  "  />
<me:PotentialPoint ReacCoord= "  4.30032E-10  "  potential= "  -69.948571  "  />
<me:PotentialPoint ReacCoord= "  4.36276E-10  "  potential= "  -70.0772205  "  />
<me:PotentialPoint ReacCoord= "  4.42519E-10  "  potential= "  -70.1454835  "  />
<me:PotentialPoint ReacCoord= "  4.48763E-10  "  potential= "  -70.2505035  "  />
<me:PotentialPoint ReacCoord= "  4.55007E-10  "  potential= "  -70.3607745  "  />
<me:PotentialPoint ReacCoord= "  4.61251E-10  "  potential= "  -70.5340575  "  />
<me:PotentialPoint ReacCoord= "  4.67495E-10  "  potential= "  -70.662707  "  />
<me:PotentialPoint ReacCoord= "  4.73739E-10  "  potential= "  -70.8386155  "  />
<me:PotentialPoint ReacCoord= "  4.79983E-10  "  potential= "  -71.0434045  "  />
<me:PotentialPoint ReacCoord= "  4.86226E-10  "  potential= "  -71.287576  "  />
<me:PotentialPoint ReacCoord= "  4.9247E-10  "  potential= "  -71.597385  "  />
     </me:IRCPotential>
     </me:tunneling>
     </reaction>

     <reaction active="true" id="R4">
     <reactant>
     <molecule role="modelled" ref="COC=O"/>
     </reactant>
     <reactant>
     <molecule role="excessReactant" ref="[OH]"/>
     </reactant>
     <product>
     <molecule role="modelled" ref="CH3_pre_comp"/>
     </product>
       <me:MCRCMethod xsi:type="me:MesmerILT">
         <me:preExponential units="cm3molecule-1s-1">3.e-10</me:preExponential>
         <me:activationEnergy units="cm-1">0.0</me:activationEnergy>
         <me:nInfinity>0</me:nInfinity>
```

</me:MCRCMethod>
<me:excessReactantConc>1E16</me:excessReactantConc>
</reaction>
<reaction active="true" id="R5">
<reactant>
<molecule role="modelled" ref="CH3_pre_comp"/>
</reactant>
<product>
<molecule role="sink" ref="CH3_post_comp"/>
</product>
<me:MCRCMethod name="RRKM"/>
<me:tunneling xsi:type="me:WKB">
<me:IRCPotential units="kJ/mol" ReducedMass="1">
<me:PotentialPoint ReacCoord= " -2.8774E-10 " potential= " 0 " />
<me:PotentialPoint ReacCoord= " -2.82E-10 " potential= " 0.4384585 " />
<me:PotentialPoint ReacCoord= " -2.75495E-10 " potential= " 0.514598 " />
<me:PotentialPoint ReacCoord= " -2.69372E-10 " potential= " 0.304558 " />
<me:PotentialPoint ReacCoord= " -2.63249E-10 " potential= " 0.204789 " />
<me:PotentialPoint ReacCoord= " -2.57126E-10 " potential= " 0.0708885 " />
<me:PotentialPoint ReacCoord= " -2.51003E-10 " potential= " 0.703634 " />
<me:PotentialPoint ReacCoord= " -2.44881E-10 " potential= " 1.5516705 " />
<me:PotentialPoint ReacCoord= " -2.38758E-10 " potential= " 2.032137 " />
<me:PotentialPoint ReacCoord= " -2.32635E-10 " potential= " 2.1030255 " />
<me:PotentialPoint ReacCoord= " -2.26512E-10 " potential= " 2.2343005 " />
<me:PotentialPoint ReacCoord= " -2.2039E-10 " potential= " 1.916615 " />
<me:PotentialPoint ReacCoord= " -2.14267E-10 " potential= " 1.8667305 " />
<me:PotentialPoint ReacCoord= " -2.08E-10 " potential= " 1.937619 " />
<me:PotentialPoint ReacCoord= " -2.02E-10 " potential= " 1.9454955 " />
<me:PotentialPoint ReacCoord= " -1.959E-10 " potential= " 1.9612485 " />
<me:PotentialPoint ReacCoord= " -1.89777E-10 " potential= " 2.5388585 " />
<me:PotentialPoint ReacCoord= " -1.84E-10 " potential= " 3.985509 " />
<me:PotentialPoint ReacCoord= " -1.78E-10 " potential= " 4.63E+00 " />
<me:PotentialPoint ReacCoord= " -1.71411E-10 " potential= " 4.7862865 " />
<me:PotentialPoint ReacCoord= " -1.65289E-10 " potential= " 4.5552425 " />
<me:PotentialPoint ReacCoord= " -1.59167E-10 " potential= " 4.3662065 " />
<me:PotentialPoint ReacCoord= " -1.53045E-10 " potential= " 4.400338 " />
<me:PotentialPoint ReacCoord= " -1.46924E-10 " potential= " 4.6550115 " />
<me:PotentialPoint ReacCoord= " -1.40803E-10 " potential= " 5.0908445 " />
<me:PotentialPoint ReacCoord= " -1.34682E-10 " potential= " 5.692084 " />
<me:PotentialPoint ReacCoord= " -1.28561E-10 " potential= " 6.400969 " />
<me:PotentialPoint ReacCoord= " -1.22441E-10 " potential= " 7.052093 " />
<me:PotentialPoint ReacCoord= " -1.1632E-10 " potential= " 7.6585835 " />
<me:PotentialPoint ReacCoord= " -1.10199E-10 " potential= " 8.527624 " />
<me:PotentialPoint ReacCoord= " -1.04079E-10 " potential= " 9.205003 " />
<me:PotentialPoint ReacCoord= " -9.79574E-11 " potential= " 9.7694855 " />
<me:PotentialPoint ReacCoord= " -9.18362E-11 " potential= " 10.2893345 " />
<me:PotentialPoint ReacCoord= " -8.57145E-11 " potential= " 10.785554 " />
<me:PotentialPoint ReacCoord= " -7.95922E-11 " potential= " 11.23714 " />
<me:PotentialPoint ReacCoord= " -7.347E-11 " potential= " 11.9118935 " />
<me:PotentialPoint ReacCoord= " -6.73478E-11 " potential= " 12.6942925 " />

<me:PotentialPoint ReacCoord= " -6.1225E-11 " potential= " 13.573835 " />
<me:PotentialPoint ReacCoord= " -5.51028E-11 " potential= " 14.5321425 " />
<me:PotentialPoint ReacCoord= " -4.898E-11 " potential= " 15.553462 " />
<me:PotentialPoint ReacCoord= " -4.29E-11 " potential= " 16.6482955 " />
<me:PotentialPoint ReacCoord= " -3.67345E-11 " potential= " 17.8350215 " />
<me:PotentialPoint ReacCoord= " -3.06117E-11 " potential= " 19.0532535 " />
<me:PotentialPoint ReacCoord= " -2.4489E-11 " potential= " 20.237354 " />
<me:PotentialPoint ReacCoord= " -1.83662E-11 " potential= " 21.4004505 " />
<me:PotentialPoint ReacCoord= " -1.22434E-11 " potential= " 22.537292 " />
<me:PotentialPoint ReacCoord= " -6.12119E-12 " potential= " 23.4588425 " />
<me:PotentialPoint ReacCoord= " 0 " potential= " 23.928807 " />
<me:PotentialPoint ReacCoord= " 6.11858E-12 " potential= " 23.7581495 " />
<me:PotentialPoint ReacCoord= " 1.22324E-11 " potential= " 23.5481095 " />
<me:PotentialPoint ReacCoord= " 1.82804E-11 " potential= " 2.19E+01 " />
<me:PotentialPoint ReacCoord= " 2.43566E-11 " potential= " 2.07E+01 " />
<me:PotentialPoint ReacCoord= " 3.04679E-11 " potential= " 17.028993 " />
<me:PotentialPoint ReacCoord= " 3.6587E-11 " potential= " 9.6119555 " />
<me:PotentialPoint ReacCoord= " 4.27087E-11 " potential= " -1.848352 " />
<me:PotentialPoint ReacCoord= " 4.88299E-11 " potential= " -16.1336975 " />
<me:PotentialPoint ReacCoord= " 5.49469E-11 " potential= " -29.3872215 " />
<me:PotentialPoint ReacCoord= " 6.09703E-11 " potential= " -34.3336635 " />
<me:PotentialPoint ReacCoord= " 6.69592E-11 " potential= " -37.219088 " />
<me:PotentialPoint ReacCoord= " 7.30657E-11 " potential= " -4.02E+01 " />
<me:PotentialPoint ReacCoord= " 7.9178E-11 " potential= " -42.7405145 " />
<me:PotentialPoint ReacCoord= " 8.5294E-11 " potential= " -45.1087155 " />
<me:PotentialPoint ReacCoord= " 9.14126E-11 " potential= " -47.3036335 " />
<me:PotentialPoint ReacCoord= " 9.75332E-11 " potential= " -49.4040335 " />
<me:PotentialPoint ReacCoord= " 1.03654E-10 " potential= " -51.4598 " />
<me:PotentialPoint ReacCoord= " 1.09777E-10 " potential= " -53.376415 " />
<me:PotentialPoint ReacCoord= " 1.15898E-10 " potential= " -55.177508 " />
<me:PotentialPoint ReacCoord= " 1.22021E-10 " potential= " -56.889334 " />
<me:PotentialPoint ReacCoord= " 1.28143E-10 " potential= " -58.401622 " />
<me:PotentialPoint ReacCoord= " 1.34266E-10 " potential= " -59.556842 " />
<me:PotentialPoint ReacCoord= " 1.40388E-10 " potential= " -60.643799 " />
<me:PotentialPoint ReacCoord= " 1.4651E-10 " potential= " -61.6336125 " />
<me:PotentialPoint ReacCoord= " 1.52632E-10 " potential= " -62.59192 " />
<me:PotentialPoint ReacCoord= " 1.58754E-10 " potential= " -63.5082195 " />
<me:PotentialPoint ReacCoord= " 1.64876E-10 " potential= " -64.5689215 " />
<me:PotentialPoint ReacCoord= " 1.70997E-10 " potential= " -65.579739 " />
<me:PotentialPoint ReacCoord= " 1.77118E-10 " potential= " -66.2702455 " />
<me:PotentialPoint ReacCoord= " 1.83238E-10 " potential= " -66.7375845 " />
<me:PotentialPoint ReacCoord= " 1.89357E-10 " potential= " -67.086776 " />
<me:PotentialPoint ReacCoord= " 1.95476E-10 " potential= " -67.4622225 " />
<me:PotentialPoint ReacCoord= " 2.01595E-10 " potential= " -67.8928045 " />
<me:PotentialPoint ReacCoord= " 2.07714E-10 " potential= " -67.9873225 " />
<me:PotentialPoint ReacCoord= " 2.13834E-10 " potential= " -68.4914185 " />
<me:PotentialPoint ReacCoord= " 2.19954E-10 " potential= " -69.481232 " />
<me:PotentialPoint ReacCoord= " 2.26075E-10 " potential= " -70.631201 " />
<me:PotentialPoint ReacCoord= " 2.32196E-10 " potential= " -7.17E+01 " />
<me:PotentialPoint ReacCoord= " 2.38316E-10 " potential= " -72.0358435 " />

<me:PotentialPoint ReacCoord= " 2.44438E-10 " potential= " -72.1933735 " />
<me:PotentialPoint ReacCoord= " 2.50559E-10 " potential= " -72.2143775 " />
<me:PotentialPoint ReacCoord= " 2.56681E-10 " potential= " -72.379784 " />
<me:PotentialPoint ReacCoord= " 2.62802E-10 " potential= " -72.9600195 " />
<me:PotentialPoint ReacCoord= " 2.68924E-10 " potential= " -74.044351 " />
<me:PotentialPoint ReacCoord= " 2.75046E-10 " potential= " -74.868758 " />
<me:PotentialPoint ReacCoord= " 2.81168E-10 " potential= " -74.6403395 " />
<me:PotentialPoint ReacCoord= " 2.8729E-10 " potential= " -73.7161635 " />
<me:PotentialPoint ReacCoord= " 2.93412E-10 " potential= " -73.057163 " />
<me:PotentialPoint ReacCoord= " 2.99535E-10 " potential= " -73.177936 " />
<me:PotentialPoint ReacCoord= " 3.05657E-10 " potential= " -74.086359 " />
<me:PotentialPoint ReacCoord= " 3.1178E-10 " potential= " -75.168065 " />
<me:PotentialPoint ReacCoord= " 3.17902E-10 " potential= " -75.9425875 " />
<me:PotentialPoint ReacCoord= " 3.24025E-10 " potential= " -75.924209 " />
</me:IRCPotential>
</me:tunneling>
<me:transitionState>
<molecule role="transitionState" ref="TS_COC=O_[CH2]OC=O"/>
</me:transitionState>
</reaction>

</reactionList>
<me:conditions>
<me:bathGas>He</me:bathGas>
<me:PTs>

<me:PTpair units="PPCC" P=" 5E+16 " T=" 213 "
precision="d"><me:experimentalEigenvalue EigenvalueID="1"
error="185">1.26e3</me:experimentalEigenvalue></me:PTpair>
<me:PTpair units="PPCC" P=" 5E+16 " T=" 233 "
precision="d"><me:experimentalEigenvalue EigenvalueID="1"
error="185">1.19e3</me:experimentalEigenvalue></me:PTpair>
<me:PTpair units="PPCC" P=" 5E+16 " T=" 261 "
precision="d"><me:experimentalEigenvalue EigenvalueID="1"
error="185">1.24e3</me:experimentalEigenvalue></me:PTpair>
<me:PTpair units="PPCC" P=" 5E+16 " T=" 294 "
precision="d"><me:experimentalEigenvalue EigenvalueID="1"
error="185">1.61e3</me:experimentalEigenvalue></me:PTpair>
<me:PTpair units="PPCC" P=" 5E+16 " T=" 294 "
precision="d"><me:experimentalEigenvalue EigenvalueID="1"
error="185">1.81e3</me:experimentalEigenvalue></me:PTpair>
<me:PTpair units="PPCC" P=" 5E+16 " T=" 380 "
precision="d"><me:experimentalEigenvalue EigenvalueID="1"
error="385">3.85e3</me:experimentalEigenvalue></me:PTpair>
<me:PTpair units="PPCC" P=" 5E+16 " T=" 418 "
precision="d"><me:experimentalEigenvalue EigenvalueID="1"
error="513">5.13e3</me:experimentalEigenvalue></me:PTpair>
<me:PTpair units="PPCC" P=" 5E+16 " T=" 454 "
precision="d"><me:experimentalEigenvalue EigenvalueID="1"
error="588">5.88e3</me:experimentalEigenvalue></me:PTpair>

<me:PTpair units="PPCC" P=" 5E+16 " T=" 495 "
precision="d"><me:experimentalEigenvalue EigenvalueID="1"
error="675">6.75e3</me:experimentalEigenvalue></me:PTpair>
<me:PTpair units="PPCC" P=" 5E+16 " T=" 541 "
precision="d"><me:experimentalEigenvalue EigenvalueID="1"
error="959">9.59e3</me:experimentalEigenvalue></me:PTpair>
<me:PTpair units="PPCC" P=" 5E+16 " T=" 573 "
precision="d"><me:experimentalEigenvalue EigenvalueID="1"
error="1080">10800</me:experimentalEigenvalue></me:PTpair>

</me:PTs>
<me:InitialPopulation>
<me:molecule grain="1.0" population="1.0" ref="COC=O"/>
</me:InitialPopulation>
</me:conditions>
<me:modelParameters>
<me:grainSize units="cm-1">50</me:grainSize>
<me:energyAboveTheTopHill>25.0</me:energyAboveTheTopHill>
</me:modelParameters>
<me:control>
    <me:printSpeciesProfile/>
<me:ForceMacroDetailedBalance/>
<me:eigenvalues>6</me:eigenvalues>
</me:control>
</me:mesmer>
